# X-Ray Crystal and Cryo-Electron Microscopy Structure Analysis Unravels How the Unique Thylakoid Lipid Composition Is Utilized by Cytochrome *b*_6_*f* for Driving Reversible Proteins’ Reorganization During State Transitions

**DOI:** 10.3390/membranes15050143

**Published:** 2025-05-08

**Authors:** Radka Vladkova

**Affiliations:** Institute of Biophysics and Biomedical Engineering, Bulgarian Academy of Sciences, Acad. G. Bonchev Str., Bl. 21, 1113 Sofia, Bulgaria; rvladkova@bio21.bas.bg

**Keywords:** carotenoid, chlorophyll *a*, cytochrome *b*_6_*f* conformational dynamics, dynamics of lipid nanoenvironment, hydrophobic mismatch, signaling, thylakoid lipid raft-like nanodomains

## Abstract

The rapid regulatory mechanism of light-induced state transitions (STs) in oxygenic photosynthesis is particularly appealing for membrane-based applications. This interest stems from the unique ability of the thylakoid membrane protein cytochrome *b*_6_*f* (cyt*b*_6_*f*) to increase or decrease its hydrophobic thickness (d_P_) in parallel with the reduction or oxidation of the PQ pool induced by changes in light quality. This property appears to be the long-sought biophysical driver behind the reorganizations of membrane proteins during STs. This study decisively advances the hydrophobic mismatch (HMM) model for cyt*b*_6_*f*-driven STs by thoroughly analyzing thirteen X-ray crystal and eight cryo-electron microscopy cyt*b*_6_*f* structures. It uncovers the lipid nanoenvironments that cyt*b*_6_*f*, with different hydrophobic thicknesses, selectively attracts. Under optimal, stationary conditions for photosynthesis in low light, when there is hydrophobic matching between the hydrophobic thicknesses of cyt*b*_6_*f* d_P_ and that of the bulk thylakoid lipid phase d_L_, d_P_ = d_L_, cyt*b*_6_*f* predominantly binds to anionic lipids—several phosphatidylglycerol (PG) molecules and one sulfoquinovosyldiacylglycerol (SQDG) molecule. Upon the induction of the transition to State 2, when d_P_ increases and induces a positive HMM (d_P_ > d_L_), the neutral, non-bilayer-forming lipid monogalactosyldiacylglycerol (MGDG) replaces some of the bound PGs. Upon the induction of the transition to State 1, when d_P_ decreases and induces a negative HMM (d_P_ < d_L_), PGs and SQDG detach from their binding sites, and two neutral, bilayer-forming lipids such as digalactosyldiacylglycerol (DGDG) occupy two sites. Additionally, this research uncovers two lipid-mediated signaling pathways from Chl*a* to the center of flexibility, the Phe/Tyr124*^fg^*^-loop-suIV^ residue—one of which involves β-carotene. This study identifies two novel types of lipid raft-like nanodomains that are devoid of typical components, such as sphingomyelin and cholesterol. These findings firmly validate the HMM model and underscore the STs as the first recognized functional process that fully utilizes the unique and evolutionarily conserved composition of just four thylakoid lipid classes. This research contributes to our understanding of membrane dynamics in general and STs in particular. It introduces a novel and simple approach for reversible protein reorganization driven purely by biophysical mechanisms, with promising implications for various membrane-based applications.

## 1. Introduction

A classical question regarding most biological membranes is why there is such a huge lipid diversity [1,2]. In contrast, for oxygen-evolving photosynthetic (thylakoid) membranes of cyanobacteria, algae, and plants, the question is why nature evolutionarily conserved a unique lipid composition of just four lipid classes [3,4]. These include two galactolipids—monogalactosyldiacylglycerol (MGDG), the only non-bilayer-forming lipid, at ~50 mol%, and digalactosyldiacylglycerol (DGDG) at 20–30 mol%—one sulpholipid, sulfoquinovosyldiacylglycerol (SQDG) at 10–15 mol%, and only one phospholipid—phosphatidylglycerol (PG) at 10–15 mol%; MGDG and DGDG are uncharged lipids, while SQDG and PG are anionic at neutral pH [5,6,7,8]. This lipid profile is distinct from other biomembranes as it lacks the domination of phospholipids. The thylakoid membrane is an internal membrane in cyanobacterial cells (prokaryotes) and the chloroplasts of algae and plants (eukaryotes). These organisms perform the Earth’s most important light-induced biological process—oxygenic photosynthesis—converting sunlight into biologically useful chemical energy and supplying oxygen in the air that we breathe, food to eat, raw materials for building, fuel for heating, and plentiful green nature for relaxation [9,10]. The thylakoid membrane is where the primary light reactions (light-harvesting, excitation energy transfer, charge separation, proton, and electron transfer) of oxygenic photosynthesis and their adaptive regulations occur (e.g., [11]). Proteins dominate the thylakoid membrane, comprising about 70–80% of its surface area [12]. The three specific integral membrane proteins involved in these events are two multi-subunit photochemically active photosystems—photosystem II (PSII) [13] and PSI [14] (with their integral or peripheral light-harvesting antenna complexes)—working in a series thanks to the third one, the cytochrome *b*_6_*f* complex (cyt*b*_6_*f)*, between them. The core molecular machinery of thylakoids that mediates photosynthetic electron transport is from PSII to cyt*b*_6_*f* via the reduced mobile lipophilic electron carrier plastoquinone (PQ), as a part of the mobile PQ pool (5–10 PQ per PSII [15]), and from cyt*b*_6_*f* to PSI via the reduced hydrophilic mobile electron carrier plastocyanin (or cytochrome *c*_6_). Remarkably, from cyanobacteria to higher plants, this core molecular machinery has remained almost unchanged [16]. In contrast, nature designed substantially different light-harvesting antenna complexes (LHCs) to PSII and PSI during evolution to adapt to different ecological niches [17,18]. In cyanobacterial thylakoids, the principal antenna to PSII is the electronegative-/cytoplasmic-/n-side peripherally bound phycobilisome (PBS) [19,20]; in algae and plants, the respective integral membrane antenna complexes to PSII and PSI are light-harvesting complex II (LHCII) and light-harvesting complex I (LHCI) [18,21].

Another unique characteristic of the thylakoid membrane is that under optimal conditions for photosynthesis, the lipid bilayer phase [22,23] consists of homogeneously distributed lipid classes [24], and the bulk lipids do not exhibit lateral heterogeneity [25]. Nevertheless, as in other biomembranes [26], there is an asymmetric distribution of lipid classes in the bulk thylakoid lipid phase (and in the photosystems) [4]. The thylakoid membrane does not contain lipids such as sphingomyelin and cholesterol [7], which are essential components for lipid raft formation in other biomembranes [27,28,29,30]. Consequently, no evidence suggests the presence of thylakoid lipid rafts, a form of liquid–liquid phase separation, i.e., liquid-ordered and liquid-disordered phases coexist [31,32]. Unlike the homogeneous lateral membrane distribution of thylakoid lipids and cyt*b*_6_*f* [33,34], the two photosystems, along with their antennas, are heterogeneously distributed in both cyanobacterial and chloroplast thylakoids. They are organized into three main microdomains. In cyanobacteria, there are PSII-PBS supercomplex-dominant microdomains, PSI-dominant microdomains, and mixed PSI and PSII-PBS microdomains [35,36]. In chloroplasts, the thylakoid membrane forms a 3D network of granal and stromal lamellae with a preferential distribution of PSII-LHCII supercomplexes in the stacked regions of the grana, PSI-LHCI in the stromal lamellae, and both PSII-LHCII and PSI-LHCI in the grana margins [37,38]. Please refer to the schematic representation of the specific photosynthetic complexes in the third mixed zone in cyanobacteria and chloroplasts in Section 4.7.

However, photosynthetic organisms are constantly subjected to variable environmental conditions, such as light quality (spectral composition) and quantity (intensity), which immediately modify the efficiency of primary light reactions and can even destroy the photosynthetic apparatus [39,40]. To optimize efficiency and protect against stress, these organisms have evolved short-term (on a timescale of seconds and minutes) and long-term (involving hours and gene expression) adaptive mechanisms. The short-term adaptive regulatory mechanisms include state transitions, non-photochemical quenching, cyclic electron transport, etc. These mechanisms involve specific dynamic structural reorganizations of the photosynthetic protein complexes, transient protein–protein interactions, and the remodeling of the thylakoid 3D membrane structure of chloroplast thylakoids, which is an area of intensive multidisciplinary research [41,42,43,44]. Remarkably, despite its simplicity and the absence of lipid rafts, the thylakoid membrane can also self-regulate the organization of membrane proteins, like other complex biomembranes [29].

However, it is still unclear why and which process regulating the primary light reactions requires and depends on the evolutionarily conserved thylakoid lipid composition [7]. Understanding the regulation of primary light reactions in oxygenic photosynthesis, particularly with regard to thylakoid lipid composition, is crucial for advancing plant biology and membrane biophysics. Furthermore, identifying this process will benefit the long-term search for biophysical drivers regulating protein organization in diverse membrane-based materials, such as engineered and artificial cells [45]. Moreover, this process will provide an alternative approach for regulating protein organization, which is distinct from that of other biomembranes, i.e., a non-raft-based approach. For instance, very recent elegant work [45] has demonstrated the power of hydrophobic mismatch, a term used to describe the difference in the length of the hydrophobic part of a protein and the hydrophobic thickness of the lipid bilayer, to drive the self-organization of designer proteins into synthetic membranes. Earlier, Milovanovic et al. [46] showed the strength of the hydrophobic mismatch as a driving force, even when it is only 1 Å. Most importantly, the vital significance of oxygenic photosynthesis [9,10] and its potential to provide bioinspired innovative solutions in diverse membrane-related fields has led to an exponentially rising number of reports in this field [47,48,49,50]. However, there is no report on replicating any fast regulatory process because the molecular mechanisms underlying it have not yet been clarified. Implementing fast regulatory mechanisms in hybrid or artificial photosynthetic cells, photovoltaic cells, and similar is of utmost importance for their applicability in various dynamic environmental conditions [48].

In this context, from a membrane biophysical viewpoint, among regulatory mechanisms, the most attractive are the light-induced state transitions, discovered in 1969 [51,52]. This is thanks to the discovery in 2016 [53] of a unique ability in the main player of state transitions—the integral membrane protein cyt*b*_6_*f*—to increase or decrease its hydrophobic thickness relative to the optimal one for its primary function: electron transport from PSII to PSI. This occurs within a few seconds and correlates with the changes in the redox state of the PQ pool [53]. This ability of cyt*b*_6_*f* raised the idea of the hydrophobic mismatch model (HMM) for cyt*b*_6_*f*-driven state transitions [53]. This study is the only one that explains how cyt*b*_6_*f* induces hydrophobic mismatch. This is possible thanks to the Chl*a* molecule inside cyt*b*_6_*f*. Based on the (i) highly significant linear correlations found between the calculated molecular (van der Waals) volume of Chl*a* (mainly of its chlorophyllide part) and several interatomic distances, representing transmembrane signal transmission, and (ii) a polynomial correlation of the Chl*a* volume with the hydrophobic thickness of the complex, as well as (iii) the absence of transmembrane signal transmission and systematic hydrophobic thickness changes in the analogous 47 cyt*bc*_1_ structures, it is proposed that Chl*a* is the long-sought crucial redox sensor and transmembrane signal transmitter in cyt*b*_6_*f* for the changes in the redox state of the PQ pool upon light-quality changes. According to the HMM model [53], cyt*b*_6_*f* can be envisioned as a perpetual motion machine for reversible two-membrane self-reorganization types whenever the spectral composition of the light changes the redox state of the PQ pool. Cyt*b*_6_*f* induces a positive (when the Chl*a* volume increases) or negative (when the Chl*a* volume decreases) hydrophobic mismatch, which leads to opposite dynamic lipid redistributions. Two different lipid types sorting around cyt*b*_6_*f* and the lipids’ automatic depletion from other areas have been proposed [53] but not yet identified. This lipid exchange changes the interaction and organization between nearby photosynthetic complexes (antennas–PSs), a behavior that has no analog in other biomembranes [53,54]. Moreover, the light-induced state transitions are a unique biomembrane regulatory mechanism as they are specific only to oxygenic photosynthesis, while non-photochemical quenching—activated upon high-intensity light illumination—also occurs in anoxygenic (bacterial) photosynthesis [55]. Cyclic electron transport around one photosystem may also happen in anoxygenic (bacterial) photosynthesis [56]. However, anoxygenic photosynthetic membranes possess a very variable lipid composition [57]. State transitions are the sole short-term adaptive mechanism that optimizes photosynthetic light reactions in low light [51,58] and is practically a non-energy-consuming process since the stimulus is just a change in the quality of low-intensity light [51,52]. Thus, state transitions seem to be the searched-for process that may rely on the unique thylakoid lipid composition.

Discovered in 1969, state transitions refer to the low-intensity light-controlled redistribution of excitation energy between PSII and PSI to optimize electron transport efficiency from PSII to PSI [51,52]. PSII is optimized for red light, while PSI is optimized for far-red light (e.g., [11]). Under low light intensity and when both photosystems work at an equal rate, their reaction centers are open, and the PQ pool redox state is more oxidized (i.e., PQ > PQH_2_) than neutral (i.e., PQ = PQH_2_). When PSII is preferentially excited (by PSII light, inducing State 2), it works faster than PSI, causing the PQ pool to become increasingly reduced (PQH_2_ ≫ PQ) to over-reduced (PQH_2_). Conversely, when PSI is preferentially excited (PSI light, inducing State 1), it operates faster than PSII, and the PQ pool becomes progressively more oxidized (PQ ≫ PQH_2_) to over-oxidized (PQ). While both scenarios may suggest decreased linear electron transport efficiency and an overall decline in photosynthesis, this is not observed in vivo because the state transition mechanism is activated by the gradual reduction or oxidation of the PQ pool induced by changes in light quality [53,59,60].

The induction phases of state transitions occur on a timescale of a few seconds [53,61] and are consistent across cyanobacteria and chloroplasts [53]. The state transition mechanism rapidly equilibrates the activities of both photosystems by redistributing the excitation energy transfer between them on a timescale of minutes. In this way, the optimal redox state of the PQ pool and the electron transport efficiency are restored [62,63]. In chloroplasts, this is achieved by relocating a fraction of the antenna complexes of PSII—specifically the loosely bound LHCII. It is noteworthy that in chloroplasts, the binding of LHCII to PSI occurs after the phosphorylation of LHCII by STN7/Stt7 kinases (in the plant *Arabidopsis thaliana* [64] and the alga *Chlamydomonas reinhardtii* [65]). The State 2 supercomplex, PSI-LHCI-LHCII, has been structurally characterized using cryo-electron microscopy (cryo-EM) methods [66]. The reverse transition to State 1 occurs through the dephosphorylation of LHCII by a constitutively active phosphatase [67,68]. In cyanobacteria, state transitions are not mediated by an antenna kinase/phosphatase pair as for chloroplasts [69,70]. The exact relocation of PBS antenna complexes in State 2 is not structurally characterized as in chloroplasts and has been debated for many years [71]. Recently, for red algae, which use PBS as the PSII antenna and LHC as the PSI antenna, it has been demonstrated by cryo-electron tomography and in situ that the PBS interactions with the photosystems are modified in State 2 [72]. In stationary State 1, the excitation energy is redistributed in favor of PSII, and in stationary State 2, it is redistributed in favor of PSI. Irrespective of the fact that the state transitions in chloroplasts are mediated by the activity of LHCII kinase/phosphatase pairs [73], while in cyanobacteria they are not [70], another shared characteristic of chloroplasts and cyanobacterial thylakoid membranes is that stationary State 1 possesses more ordered antenna–PSII megacomplexes than their stationary State 2 [19,34,74,75]. A schematic representation of the organization of stationary State 1 and stationary State 2 in cyanobacteria and chloroplast thylakoid membranes is given in Section 4.7.

The PQ pool redox state changes are sensed and transduced by the cyt*b*_6_*f* complex [53,59,76,77,78,79,80]. As noted above, the primary function of cyt*b*_6_*f* is to catalyze the electron transport from PSII to PSI, coupled with transmembrane proton transfer for ATP synthesis, just as cyt*bc*_1_ (complex III) does in anoxygenic photosynthetic bacteria and mitochondria for the respiratory electron transport chain [69,81,82]. In contrast to cyt*bc*_1_, however, cyt*b*_6_*f* additionally harbors three specific cofactors—chlorophyll *a* (Chl*a*), a carotenoid (β-, α-carotene, or echinenone, see Refs in [53]) (Car), and the heme *c*_n_ [83,84]. Additionally, the eight-helix cyt*b*_6_ subunit of cyt*bc*_1_ is split into two in cyt*b*_6_*f*—cyt*b*6 (helices A–D) and subunit IV (E–G)—and the position of helix H is substituted by Chl*a* [83,84]. Cyt*b*_6_*f* is central to regulating the primary light reactions of oxygenic photosynthesis [81,85].

Despite extensive multidisciplinary research on light-induced state transitions at various levels of system complexity over the years (for recent reviews, see [71,81,86,87]), a comprehensive understanding of this mechanism remains elusive. There is no accepted, single, generalized model for state transitions in cyanobacteria and chloroplasts that explains the spatiotemporal sequence of molecular events from induction to the onset and completion of state transitions. However, the HMM model is a unified model [53] but has received little attention.

While the induction phase and completion of state transitions are very similar in cyanobacteria and chloroplasts (see above), the exact events that occur at the onset of their state transitions remain unclear. In other words, who is the recipient of the signal outside cyt*b*_6_*f*, i.e., the primary effector of the sensed and transduced signal by cyt*b*_6_*f*? From one side, for chloroplasts, it is thought that this process involves the binding to and activation by cyt*b*_6_*f* of the LHCII kinase, which phosphorylates LHCII in grana and leads to the 3D remodeling of the grana (e.g., a decrease in grana diameter) [34,88,89]. However, most of the kinase is localized to the stroma lamella, with only a minor fraction bound to grana margins or grana stacks [90]. How the kinase reaches cyt*b*_6_*f* in grana margins and grana stacks is unknown; that is, what is the driving force behind this movement? The recent insights into the process of LHCII kinase activation by cyt*b*_6_*f*, particularly the experimentally disfavored thiol redox regulatory activation model of STN7/Stt7 LHCII kinases [91], highlight the necessity for comprehensive testing of the non-redox-based conformational change model [53], i.e., the HMM model [53]. Therefore, other models based on the redox activation of the kinase are unlikely. According to the HMM model [53], the lipid sorting around cyt*b*_6_*f* during the induction phase of the transition to State 2 may facilitate the segregation of the LHCII kinase around cyt*b*_6_*f* and the binding of the Phe/Tyr124*^fg^*^-loop-suIV^ residue to the kinase transmembrane helix residue Phe116-STN7 of the plant *Arabidopsis* or Tyr121-Stt7 of the alga *Chlamydomonas* [53]. Note that this prediction also means that the transmembrane helix of the kinase will bind not between the F and G helices of subunit IV as suggested before [92,93,94] but between the G helix of subunit IV and the small PetG single helix because therein buried in the membrane is Phe/Tyr124*^fg^*^-loop-suIV^. In line with this prediction, Arg125 *^fg^*^-loop-suIV^ is bound to the TSP9 fragment and is shielded from interaction with the kinase stroma domain [95]. The last of the models from Cramer’s group [92] is also discarded, thanks to the power of the original approach [53] to characterize each X-ray structure with its respective redox state of the PQ pool. The 4OGQ structure [96] corresponds to the oxidized state of the PQ pool, as is seen by using its hydrophobic thickness of 30.6 Å calculated with the OPM (Orientation of Proteins in Membranes) database ([97] and Figure 6C in [53]), which precludes its use as a model for interaction with the kinase [59]. The HMM model also predicts that the higher hydrophobic thickness of cyt*b*_6_*f* and the sorted lipids around cyt*b*_6_*f* may activate the kinase [53]. Therefore, it is necessary to experimentally understand the dependence of LHCII kinase activation on the material properties of the thylakoid bilayer, such as hydrophobic thickness and intrinsic lipid curvature, as already noted in [53]. The importance of such future study is demonstrated by the fact that the STN7 kinase can be activated by a light-independent pathway under low-temperature stress [98].

On the other hand, in cyanobacteria, there is still no accepted model to describe how the signal from cyt*b*_6_*f* is further processed to give rise to PBS–photosystem reorganizations during the progression of state transitions. According to the HMM model [53], the driving force for these reorganizations is created again during the induction phases, when the two types of lipid sorting—one type to reduce the positive hydrophobic mismatch and the other to minimize the negative hydrophobic mismatch created by cyt*b*_6_*f* increasing or decreasing the hydrophobic thickness during the induction of the transition to State 2 and State 1, respectively. The sorting of these two lipid types leads to opposite reorganizations and interactions at the neighboring PBS–photosystem complexes [53].

However, this model for cyanobacterial state transitions was put under question. Specifically, until 2019, it was widely accepted that cyt*b*_6_*f* functions similarly in cyanobacterial and chloroplast state transitions (e.g., [53,69,79,80]). Based on indirect evidence (experiment with the chemical TMPD), however, Calzadilla et al. [70] concluded that cyt*b*_6_*f* and its Chl*a* molecule were not involved in cyanobacterial state transitions. However, their evidence was insufficient, lacking a demonstration that point mutations [93] or modification [99] of cyt*b*_6_*f* affecting algal state transitions do not influence cyanobacterial ones. Additionally, the discussion in [70] overlooked the fact that key findings from the analysis of six cyanobacterial structures and only one algal chloroplast cyt*b*_6_*f* structure [53] have been confirmed in vivo [93], but not citing [53], through studies on the alga *Chlamydomonas* and its cyt*b*_6_*f* mutants, particularly regarding the importance of Phe/Tyr124*^fg^*^-loop-suIV^ for state transitions. Very recently, the consensus on the role of cyt*b*_6_*f* in cyanobacteria and chloroplasts was restored by Wei et al. [100], who found that the same chemical TMPD used in [70] enhances oxygen evolution (i.e., photochemical activity) in a cyanobacterial mutant with a significant loss of cyt*b*_6_*f* but does not induce state transitions, thus supporting the idea that cyt*b*_6_*f* is essential for state transitions in cyanobacteria, as it is for plants. Overall, the validation of the results from [53] supports the usefulness of the original structure-analyzing approach applied, based on correlations between Chl*a* volume changes and interatomic distances in cyt*b*_6_*f*, as well as the identified role of Chl*a* in cyanobacterial and chloroplast state transitions.

It can be seen from any review of state transitions that a role for lipids in state transitions is missing, except in [53]. Little is known beyond the principal lipid bilayer role in maintaining membrane fluidity [101,102,103,104,105,106]. This is despite membrane biophysics indicating that there are other lipid bilayer characteristics beyond fluidity [107]. X-ray crystallography and cryo-EM imaging have provided significant insights into the structural and functional roles of lipids at a molecular level. Yoshihara and Kobayashi [108] have reviewed recent advances in lipid molecules identified in photosynthetic complexes, including three cyt*b*_6_*f* structures. They have discussed the conserved and differentiated roles of lipids in the assembly and functioning of such complexes among plants, algae, and cyanobacteria but not in the context of state transitions. Currently, there are thirteen dimeric cyt*b*_6_*f* X-ray crystal structures [83,84,96,109,110,111,112,113,114], comprising twelve from cyanobacterial species and one from algae [83]. Eight cyanobacterial and plant cyt*b*_6_*f* cryo-EM structures have been determined [95,115,116,117]. Purified protein complexes used for X-ray and cryo-EM often lack full complements of lipids due to detergent solubilization. However, the different complexes regarding lipid binding can be compared. A comparison of lipid- and detergent-binding sites in three cyt*b*_6_*f* structures (PDB IDs 1Q90 [83], 2E74 [109], and 2ZT9 [110]) with a structure of yeast cyt*bc*_1_ (3CX5, [118]) has shown significant conservation of lipid positions [119]. These authors also suggested similar functions for the respective lipids and β-Car in the cyt*b*_6_*f* complex based on overlapping structures. Hasan and Cramer [120] inferred that a lipid plus Chl*a* replacement in the cyt*b*_6_*f* of helix H in the cyt*bc*_1_ structure could mediate conformational changes associated with transmembrane signaling in cyt*b*_6_*f*. However, for the same lipid and Chl*a*, later on, Hasan et al. [121] proposed a lipidic mechanism of cyt*b*_6_*f*–LHCII kinase supercomplex formation, inferring that the lipid detaches to allow the transmembrane helix of the LHCII kinase to attach in its place. Finally, the same authors proposed that several lipid sites on the surface of cyt*b*_6_*f*, located around Chl*a*, could form an adaptable surface for interaction with STN7/Stt7 kinase through lipid-mediated contacts [96]. However, as noted above, the 4OGQ structure of cyt*b*_6_*f* corresponds to the oxidized state of the PQ pool. This fact renders the suggested lipid role invalid, as the kinase is activated when the PQ pool is reduced [59]. It is thus evident that these proposals, made without taking into account that the cyt*b*_6_*f* structures represent different conformational states of cyt*b*_6_*f*, corresponding to different redox states of the PQ pool, as has already been demonstrated in [53], could not provide robust propositions for a role of lipids in state transitions.

A systematic study on the dynamics of interactions between cyt*b*_6_*f* and thylakoid lipids during state transitions has not yet been conducted. The initial work on the HMM model for cyt*b*_6_*f*-driven state transitions [53] identified the lipid phase and the lipid composition as the third main component of state transitions, alongside the major Chl*a*, which serves as the crucial redox sensor and transmembrane signal transmitter for the changes in the redox state of the PQ pool, and several amino acid residues identified as an important part of the signal transduction pathway from Chl*a* to the stroma side of the complex. Three main distances characterize the various conformations of cyt*b*_6_*f*, which correlate with the molecular volume of Chl*a* (primarily its chlorophyllide part) and the redox state of the PQ pool: the p-side gate width (p-gate), the n-side flexibility distance (dn), and the hydrophobic thickness of cyt*b*_6_*f*. It was suggested that two different lipid types are sorted around cyt*b*_6_*f* and restructured in the other regions of the thylakoid membrane during the induction phases (to State 2 and State 1) of state transitions. However, the nature of these two different lipid types remains to be clarified.

Understanding the changes in the lipid occupants of the various structures, which are ordered to mimic the conformational changes in cyt*b*_6_*f* during the two induction phases of state transitions, is crucial for identifying the two different lipid types that are believed to reduce both the positive and negative hydrophobic mismatches created by cyt*b*_6_*f* [53]. This understanding will help determine how changes in the bulk lipid phase, such as the depletion of one type of lipid and the enrichment of others, affect nearby protein–protein interactions (antennas and photosystems) during state transitions. Ultimately, this understanding will facilitate the acquisition of structural evidence that the function of cyt*b*_6_*f* in state transitions relies on the thylakoid lipid composition. Hopefully, the original approach in [53] has been experimentally validated, as noted above. One key advantage of this structure analysis is the ability to track lipid-binding dynamics to cyt*b*_6_*f* over seconds, which molecular dynamics simulations in photosynthesis cannot currently achieve [122].

The present work aimed to comprehensively test the HMM model of cyt*b*_6_*f*-driven light state transitions, focusing on the role of lipid–cyt*b*_6_*f* interactions and dynamics as the third main component of this mechanism in cyanobacterial and chloroplast state transitions [53]. A systematic analysis of 21 available X-ray and cryo-EM dimeric cyt*b*_6_*f* structures was conducted. The findings indicate that lipid binding to cyt*b*_6_*f* is variable and dependent on the PQ pool’s redox state, specifically, the conformational state of cyt*b*_6_*f*. Through visual inspection of the superimposition of these structures over a control one under hydrophobic matching conditions, it was determined whether the co-crystallized lipids and detergents retained their positions or released them and whether other lipids occupied the vacated places. It was possible to distinguish the movement of lipids and follow a lipid flip-flop from the cyt*b*_6_*f*-binding site to the bulk phase. The hydrophobic mismatch model was confirmed by identifying two lipid classes: MGDG to minimize the positive and DGDG to minimize the negative hydrophobic mismatches caused by cyt*b*_6_*f*. The HMM model was further expanded by deciphering the effects on the bulk phase and which antenna–photosystem supercomplexes can be destabilized at the onset of state transitions. Furthermore, the structural analysis provided new evidence for the major role of Chl*a* in state transitions and, for the first time, for the role of Car in cyt*b*_6_*f*. The increase in the reduction level of the PQ pool is accompanied by a shift in the Chl*a* macrocycle from a more tilted to a more parallel orientation with respect to the membrane normal. It was also identified that the Chl*a* molecule signals to the n-side center of flexibility—the Phe/Tyr124*^fg^*^-loop-suIV^ residue [53]—through a non-protein pathway. It was also demonstrated that state transitions are indeed the mechanism that requires and depends on the unique and evolutionarily conserved four-lipid-class composition of the thylakoid membrane. This work provides a novel understanding of the roles of lipids in signaling between Chl*a* and Phe/Tyr124*^fg^*^-loop-suIV^, including β-Car, and introduces two types of thylakoid lipid raft-like nanodomains. Overall, the insights into dynamic lipid binding during state transitions highlight the lipid compartment’s active, primary effector role in the thylakoid membrane, paving the way for multidisciplinary research on this topic with expected results.

## 2. Materials and Methods

A total of 21 X-ray crystal [83,84,96,109,110,111,112,113,114] and cryo-EM [95,115,116,117] structures of dimeric cyt*b*_6_*f* complexes in the Protein Data Bank (http://www.rsbs.org (accessed on 7 March 2025)) (Table S1) were analyzed. As in previous work [53], the cyt*b*_6_*f* structure files were downloaded from the OPM database (http://opm.phar.umich.edu (accessed on 7 March 2025)), [97]), except the three newest structures, 9ES7, 9ES8, and 9ES9 [95], since their OPM files have not yet been calculated. The OPM database provides the topologies (optimal positioning) of various cyt*b*_6_*f* structures in the model lipid bilayer and their hydrophobic thickness d_P_ values. The d_P_ values in this database are calculated using a method based on the evaluation of the free energy of transfer of molecules from water to an anisotropic solvent model of the fluid lipid bilayer environment under hydrophobic matching conditions (for an explanation of the method, see [123,124]). The downloaded OPM structure files provide the atomic coordinates of a protein with lipid bilayer hydrophobic boundaries located at the level of lipid carbonyls of the modeled lipid bilayer. The distance between the two boundary planes is the hydrophobic protein thickness, d_P_, which is equal to the hydrophobic thickness of the modeled fluid lipid bilayer, d_L_, under hydrophobic matching conditions (d_P_ = d_L_). The visualization, overlapping, relative rotation of the structures, and interatomic distance determinations were conducted using ViewerLite 4.2.

The analysis also includes 15 X-ray crystal structures of cyt*bc*_1_, selected from Table 1 in [53], and 9 dimeric (11 monomeric) cryo-EM structures of cyt*bc*_1_ (complex III_2_). See Table S5 for details.

For the assignment of the lipid-binding sites in the different structures, each structure was first aligned with the 4OGQ structure [96]. This was performed by manually overlapping the respective OPM structure over that of 4OGQ. This was made possible thanks to the transformed original PDB coordinates provided by the OPM database, which ensured that the calculated membrane normal coincided with the Z axis and the origin of the coordinates corresponded to the center of the membrane [97]. Only a slight rotation of each dimer structure around the Z axis was needed for its two heme-*b*_n_–heme-*b*_p_ pairs to maximally coincide with the same two heme pairs of the 4OGQ dimer structure.

To follow the changes in the occupation (exchange or escape) of the different lipid-binding sites, all available crystal and cryo-EM structures were ordered as a sequence of time-resolved snapshots of intermediate conformational states by using the width of the Chl*a*-identified redox-sensor gate in cyt*bc* complexes—the p-gate (Ala147(CA)*^cd^*^1-helix-cyt*b*6^−Leu76(N)*^ef^*^-loop-suIV^)—the n-side flexibility distance d_n_ (Phe40(CZ)^E-helix-suIV^−Phe/Tyr124(CA)*^fg^*^-loop-suIV^) (see in Section 3.2), and the cyt*b*_6_*f* hydrophobic thickness d_P_, available from the OPM database [97] and calculated as described above. These three characteristic distances can be used together as a sum or as an estimated volume of a truncated cone, with the n-distance and p-gate width representing its larger and smaller radii, respectively, and d_P_ representing the height, which serves as an empirical indicator characterizing the changes in cyt*b*_6_*f* dimer volume.

To follow the dynamic conformational changes in each lipid-binding site occupant, Chl*a*, and the Car related to state transitions, a color-blind-friendly color scheme of 23 colors was used [125] for the color coding of each structure. The increase in the wavelength of the structure color corresponds to a rise in the value for the p-side lateral p-gate width [53]. Thus, if any lipid, Chl*a*, or Car displacement is correlated with changes in the redox state of the substrate pool, then coherently rainbow-ordered colors for the respective molecule will be observed. If the displacement reflects an inherent mobility of the site, then an incoherent, dispersed picture of colors will be seen.

## 3. Results

Previous work [53] has established that the ordered nine monomeric cyt*b*_6_*f* crystal structures (the first seven dimer structures in Table S1) in the direction of increasing Chl*a* molecular volume represent a time sequence of snapshots that mimics the in vivo conformational changes in cyt*b*_6_*f* in parallel with the redox state changes in the PQ pool from an over-oxidized to an over-reduced state. The monomeric cyt*b*_6_*f* structures’ sequence is 2D2C(CLA1201) < 1VF5(CLA201) < 1VF5(CLA1201) < 2ZT9(CLA201) < 2E74(CLA201) < 2E75(CLA201) < 2D2C(CLA201) < 1Q90(CL1910) < 2E76(CLA201), wherein in brackets is the ID of the Chl*a* molecule in the respective monomer structure [53]. The stationary, oxidized state of the PQ pool in low light, from which the induction of the two transitions to State 2 and to State 1 begins, respectively, corresponds to a structure between the 1VF5 and 2ZT9 structures (see Figure 6C in [53]) (see Section 3.2 below). The two sequences of structures starting from this state represent time sequences of dynamic conformational changes in cyt*b*_6_*f* during the induction phase of the transition to State 2—when the PQ pool continuously changes its redox state from oxidized to over-reduced and the hydrophobic thickness of cyt*b*_6_*f* reaches its maximum (i.e., the structure 2E76)—and during the induction phase of the transition to State 1—when the redox state of the PQ pool changes from oxidized to fully oxidized and the hydrophobic thickness of cyt*b*_6_*f* reaches its minimum (i.e., the structure 2D2C(CLA1201)). This is possible for the X-ray crystal structures because the substrate/inhibitor pool in the crystallization solution, as the PQ pool in vivo, is in excess relative to cyt*b*_6_*f,* and the substrate/inhibitor pool and the substrate/inhibitor bound to cyt*b*_6_*f* are in equilibrium [53]. Based on the comparative analysis of the nine monomeric cyt*b*_6_*f* structures with the forty-seven cyt*bc*_1_ structures, it was also predicted that during the Q-cycle (ms time range), Chl*a* cannot sense the redox state of the PQ pool in vivo because the operation of the Q-cycle in cyt*bc*_1_ does not involve transmembrane signal transmission from the p- to n-side [53]. In the present work, these two predictions and others in [53] are tested by comparing X-ray and cryo-EM structures of cyt*b*_6_*f*. To clarify some differences between the cyt*b*_6_*f* X-ray and cryo-EM structures, a comparison was also made with the cyt*bc*_1_ X-ray and cryo-EM structures.

### 3.1. Comparison of the X-Ray Crystal and Cryo-EM Structures of Cytochrome b_6_f

#### 3.1.1. Characteristic Distances for the Conformational Dynamics of Cyt*b*_6_*f*

Table S1 shows all twenty-one of the currently available dimeric cyt*b*_6_*f* structures—thirteen obtained by X-ray crystallography [83,84,96,109,110,111,112,113,114] and eight by the cryo-EM method [95,115,116,117]. As seen from the overview of all these structures (Text S1), they represent a valuable database for assessing the response of the lipid compartment of cyt*b*_6_*f* to changes in the protein’s conformational state. The conformational changes in cyt*b*_6_*f* have been previously characterized by identifying several characteristic distances that are strongly correlated with the molecular volume of Chl*a* [53]. The exact mechanism is not clear. It is probably due to the established ability of Chl*a* to significantly stimulate volume-controlled lipid phase transformations such as the lamellar liquid crystalline to non-lamellar inverted hexagonal (Lα→H_II_) phase transformation [126]. Chl*a* does not stabilize the H_II_ phase by filling the interstitial “voids” as alkanes (see discussion in [126]). Rather, it reduces the radius of the spontaneous monolayer curvature [126]. Because the Lα→H_II_ phase transition represents a significant topological change with low enthalpy and Chl*a* has the ability to strongly favor such transformation [126], it is not unreasonable to expect that the change in Chl*a* volume expressed as the induction of negative curvature can promote the revealed conformational changes in cyt*b*_6_*f*. The identified distances are also mutually connected because they correlate with the same parameter—the volume of Chl*a* [53]. As demonstrated in [53], these distances also directly correlate with each other. These characteristic distances are the distance of the [2Fe-2S] center (i) to several Chl*a* atoms, including its metal center—the Mg atom, (ii) to the C34 of the Car, (iii) to several amino acid residues, (iv) the p-side lateral gate width (Ala147(CA)*^cd^*^1-helix-cyt*b*6^−Leu76(N)*^ef^*^-loop-suIV^, p-gate), (v) the n-side flexibility distance (Phe40(CZ)^E-helix-suIV^−Phe/Tyr124(CA)*^fg^*^-loop-suIV^, d_n_) (see in Section 3.2, see also Figure 2 and Table 2 in [53] for all distance correlations with the Chl*a* volume), and (vi) the hydrophobic thickness of cyt*b*_6_*f* d_P_ (OPM database [97]), which increases in a polynomial manner (first a steep linear increase and then a slower increase) with the rise in Chl*a* volume (see Figure 6C in [53]). In addition, with the increase in Chl*a* volume, the identified most important flexibility center at the n-side of cyt*b*_6_*f*—Phe/Tyr124*^fg^*^-loop-suIV^—rotates from the n-side directed towards the membrane, becoming buried. The other residue is Phe40 at the n-side end of helix E of subunit IV (Phe40^E-helix-suIV^), closest to the Fe of heme *c*_n_. Its mobility is smaller; this residue shifts slightly by TDS at the *c*_n_ position. The d_n_-distance (Phe40^E-helix-suIV^-Phe/Tyr124*^fg^*^-loop-suIV^) characterizes the flexibility region between the n-side end of helix E (Phe40) and the *fg*-loop between helices F and G of subunit IV (see [53] for more details). Note that recently, the peripheral subunit PetP in cyanobacterial cyt*b*_6_*f* cryo-EM structure 7R0W [116] and the TDS9 fragment in plant cyt*b*_6_*f* cryo-EM structures 7ZYV [117], 9ES7, 9ES8, and 9ES9 [95] were demonstrated exactly in this region of flexibility.

Special attention is required for the Chl*a*-identified universal p-side lateral gate for both cyt*bc* complexes (cyt*b*_6_*f* and cyt*bc*_1_). The width of the p-gate is the atomic distance (Ala147*^cd^*^1-helix-cyt*b*6^–Leu76*^ef^*^-loop-suVI^) between Ala147 of the small *cd*_1_-helix of cyt*b*_6_ and Leu76 of the *ef*-loop of the subunit IV of cyt*b*_6_*f* (see in Section 3.2). The p-gate is not at the entrance of the Qp-site from the intermonomer cavity. It is the lateral gate for the access of the Qp-site-bound inhibitor, mimicking the native quinol substrate, to the [2Fe-2S] cluster ligand residue His of loop 2 in the Rieske ISP extra-membrane domain (ISP-ED). This Chl*a*-identified p-side lateral gate exhibits excellent Qp-site inhibitor-type sensitivity, as documented for 47 [53] and later for 56 [127] cyt*bc*_1_ complexes. Four ranges of the width of this gate define the different positions and mobility of the [2Fe-2S] cluster relative to the three cyt*bc*_1_ (cyt*b*_6_*f*) hemes *b*_L_ (*b*_p_), *b*_H_ (*b*_n_), and *c*_1_ (*f*) [53]. Range 1 (9.8–8.1 Å) defines [2Fe-2S] fixed at the *b*-position, observed with SMA-like inhibitors (H-bond between the inhibitor/substrate occupant of the Qo(p)-site and the His residue from loop 2 of the ISP-ED, which is a ligand to the [2Fe-2S] cluster); Range 2 (7.8–7.4 Å) defines [2Fe-2S] fixed at the *b*-position, observed with famoxadone-like inhibitors (no H-bond as in Range 1). It is interesting to note that this state of [2Fe-2S] has not yet been observed in cyt*b*_6_*f* structures; Range 3 (7.4–6.8 Å) defines [2Fe-2S] mobile at *b*-released and intermediate *I*_2_-positions, observed with MOA-like inhibitors, and Range 4 (6.7–6.0 Å) defines an empty Qo-site, with [2Fe-2S] mobile at all positions—*b*-, intermediate *I*_1_-, *I*_2_-, and *c*_1_-positions [53]. In contrast to other distances identified in the Qp-site cyt*bc*_1_ literature (e.g., [128], see [53] for more details), the Chl*a*-identified p-gate can distinguish between the two *b*-fixed positions, the two mobile positions, the *b*-fixed and *b*-mobile positions. The rule is the smaller the p-gate, the higher the [2Fe-2S] mobility, and the intermediate states and *c*_1_(*f*) are resolved in X-ray crystal structures (see [53] and Table 1 therein for details of sources).

Previous work [53] analyzed seven of thirteen X-ray cyt*b*_6_*f* dimeric structures. The remaining six were excluded from the correlation analysis with the volume of Chl*a* because it has a modified molecular structure, with three He and seven B atoms added to it. For unclear reasons, the Chl*a* volume seems to be fixed [53]. However, thanks to the significant correlations of the above-noted interatomic distances with the volume of Chl*a* [53] found, in the present work, all the X-ray crystal structures and all the cryo-EM structures are ordered and analyzed using three characteristic distances, the width of the p-side lateral gate p-gate (Ala147*^cd^*^1-helix-cyt*b*6^–Leu76*^ef^*^-loop-suVI^), the n-side flexibility distance d_n_ (Phe40^E-helix-suIV^−Phe/Tyr124*^fg^*^-loop-suIV^), and the cyt*b*_6_*f* hydrophobic thickness d_P_ [53], the last taken from the OPM database [97] (see the first two columns in Tables S2 and S3). The three distances can be used together as a sum or as a calculated volume of a truncated cone, with the n- and p-distances representing its larger and smaller radii, respectively, and d_P_ as the height, which represents an empirical indicator characterizing the changes in the cyt*b*_6_*f* dimer volume.

#### 3.1.2. The Cytochrome *b*_6_*f* Cryo-EM Structures Are More Swollen at the n-Side than the Cyt*bc*_1_ Cryo-EM Structures

The first notable aspect when comparing these three characteristic distances in the X-ray (Table S2) and cryo-EM (Table S3) structures of cyt*b*_6_*f* is that at equally small p-gate widths, i.e., p-gate ≤ 6.6 Å, the hydrophobic thickness d_P_ for cryo-EM structures is larger by 1–2 Å than that for X-ray structures of cyt*b*_6_*f*. At a p-gate of around 6 Å, both d_P_ and the flexibility distance d_n_ are larger in cryo-EM structures by 2–3 Å. To understand why this is observed, the mean distances between the metal centers of the hemes for cyt*b*_6_*f* and cyt*bc*_1_ and the two Chl*a* in the cyt*b*_6_*f* dimer in cryo-EM structures were compared with those in X-ray structures (Table S4). The data show that the cryo-EM cyt*b*_6_*f* structures are more swollen in the n-side membrane half of the protein than the X-ray structures and cryo-EM structures of cyt*bc*_1_. This may reflect the more plastic structure of cyt*b*_6_*f* relative to cyt*bc*_1_ in the n-side leaflet and the more distant heme *f* from the membrane hemes in cyt*b*_6_*f* relative to heme *c*_1_ in cyt*bc*_1_ (see Figure S1, Table S5, and Text S4). Table S5 shows that the structure method has an effect on the [2Fe-2S]-Fe-heme distances for cyt*bc* complexes.

#### 3.1.3. The Chlorophyll *a* Redox Sensor and Transmembrane Signal Transmission Role Is Not Active During the Q-Cycle

The present availability of cryo-EM structures provides an excellent opportunity first to directly and independently check the main prediction of the previous work [53]; this is that Chl*a* acts as a redox sensor and signal transducer during the induction phases of state transitions but not during the regular function of cyt*b*_6_*f* to catalyze the proton-coupled electron transport from PSII to PSI via the Q-cycle. The time component of the above-noted sequences is established to be a few seconds, corresponding to the experimentally determined duration of the induction phases of the state transitions [53]. This is consistent with the experimentally determined duration of continuous changes in the redox state of the PQ pool upon changes in light quality [61]. In contrast, in native membranes, only the sensor role of the p-gate width for the substrate redox state within the Qp-pocket is valid [53]. The timescale of the Q-cycle operation during the regular function of cyt*b*_6_*f* to catalyze proton-coupled electron transport is in the range of a few milliseconds (~2.5–4 ms [129,130]). The cryo-EM structures are snapshots of different transient states of the catalytic Q-cycle of cyt*b*_6_*f* [95]. Compared to the substrate pool, these structures are obtained at a non-equilibrium, transient state, where the substrate has already undergone a change in its redox state during cryo-EM sample vitrification [131].

**Figure 1:**
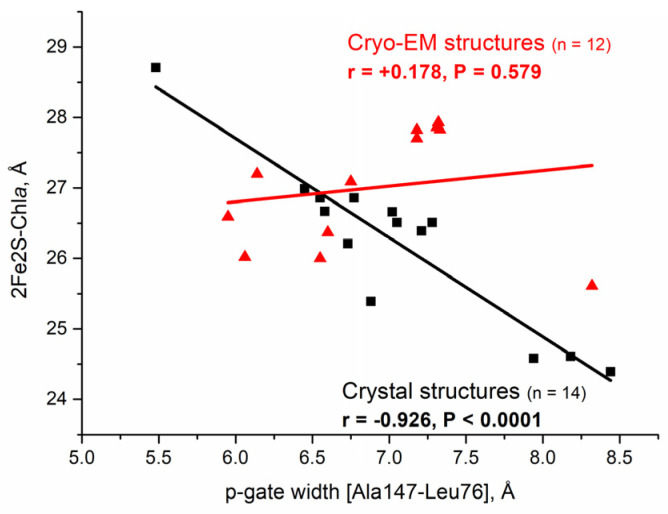
Linear regression analysis (solid lines) and correlation coefficients (Pearson correlation, r) of the relationship between the p-gate width and the [2Fe-2S]–Chl*a* distance in the X-ray crystal (black squares) and cryo-EM (red triangles) monomeric cyt*b*_6_*f* structures. The width of the p-side lateral gate is the distance (Ala147*^cd^*^1-helix-cyt*b*6^–Leu76*^ef^*^-loop-suVI^); [2Fe-2S]–Chl*a* is the distance between the center of the [2Fe-2S] center and the Mg of Chl*a*. There are 14 crystal structures of monomeric cyt*b*_6_*f* included, not 15, because [2Fe-2S] is missing in the structure 4I7Z.

According to previous work [53], the Chl*a* volume (mainly its chlorophyllide part) can sense the redox state of the PQ pool by the position of the [2Fe-2S] center to the Mg of Chl*a*. This is because, among the metal centers of cyt*b*_6_*f*, the Mg is the closest to the [2Fe-2S] and senses the full amplitude of [2Fe-2S] movement [53]. To determine whether Chl*a* can detect the [2Fe-2S] position in the cryo-EM structures, the relationship between the redox state of the substrate/inhibitor at the Qp-site and the position of the [2Fe-2S] cluster relative to the Mg of Chl*a* has been investigated (Figure 1).

As seen from the Pearson’s correlation coefficient, r, and the associated *p* value, indicated in Figure 1, there is a very strong (according to [132]) negative linear correlation between the p-gate width and the distance of the [2Fe-2S] center to the Mg of Chl*a* for the X-ray crystal structures. In contrast, the very low r value and the very high *p* value in the case of the cryo-EM structures undoubtedly indicate closeness to the null hypothesis for a relationship between the two variables.

The fact that the cryo-EM structures do not exhibit the same very strong, negative linear correlation as the X-ray structures is convincing structural evidence that during the operation of the catalytic Q-cycle in the ms time range, there is no signal sensing of the PQ pool redox state by Chl*a* related to state transition induction. This result confirms the indirect structural evidence from previous work, indicating that signal sensing and transduction occur on a much longer timescale [53]. This result is important not only in that it confirms a conclusion from previous work [53] based on indirect evidence (comparison of the p-gate-ordered sequences of cyt*b*_6_*f* and cyt*bc*_1_ structures) but also because it provides strong evidence for the time ranges of the lipid-binding dynamics in the two sets of ordered X-ray and cryo-EM structures in the present work: a few seconds for the X-ray crystal structures and a few milliseconds for the cryo-EM structures. Therefore, it is evident that the X-ray crystal and cryo-EM changes in the three characteristic distances of cyt*b*_6_*f* cannot be combined into a single table to characterize state-transition-related changes.

### 3.2. Lipid-Binding Sites of Cytb_6_f Under Optimal Conditions Under Low-Intensity Light

**Figure 2:**
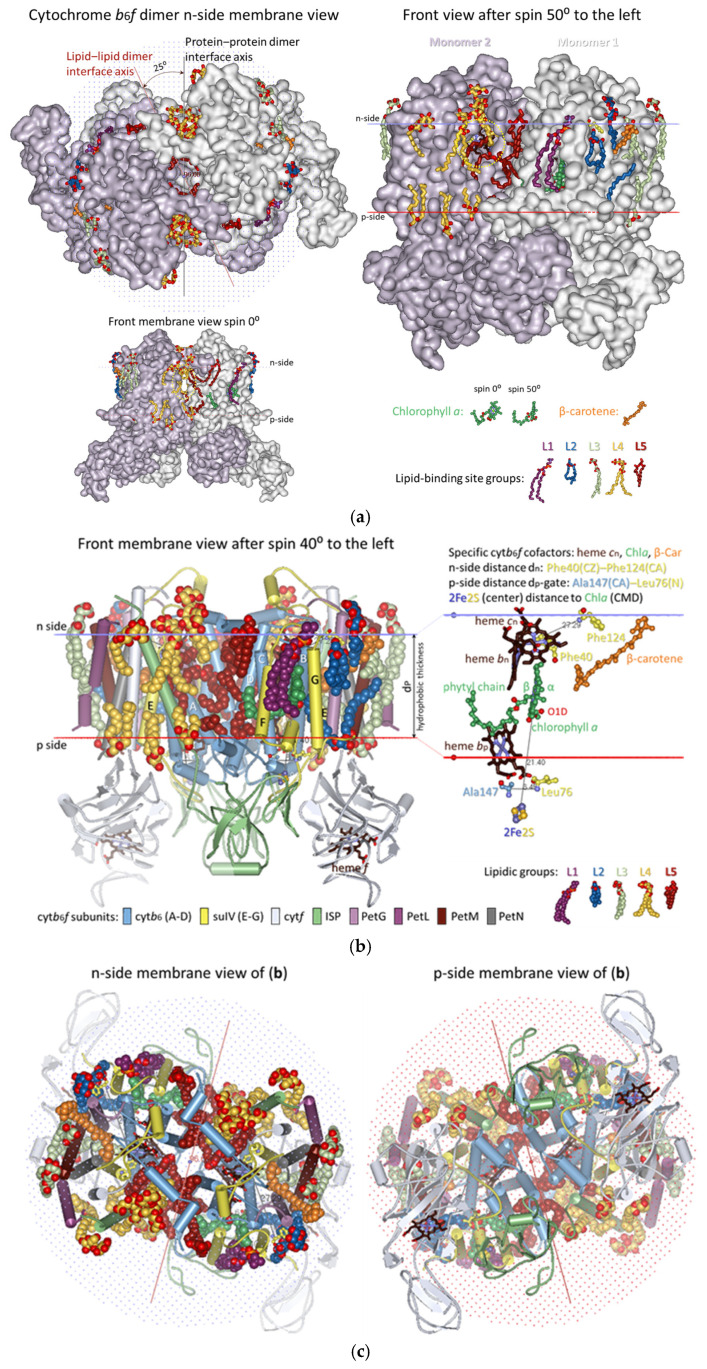
Overview of the selected reference X-ray crystal structure of the cytochrome *b*_6_*f* dimer for comparative analysis of changes in the cyt*b*_6_*f*–lipid interactions in other structures. This structure file represents the cyanobacterium *Nostoc* sp. PCC 7120, with PDB ID 4OGQ [96], features a maximum of 22 lipid-binding sites per monomer, and is sourced from the OPM database [97]. The OPM structure file provides the topology of cyt*b*_6_*f* in a modeled lipid bilayer membrane with calculated electronegative n-side (blue) and electropositive p-side (red) lipid hydrophobic boundary planes at the level of lipid carbonyls. The distance between the two planes of the modeled lipid bilayer is the calculated hydrophobic thickness of both the cyt*b*_6_*f* (d_P_) and the modeled lipid bilayer (d_L_), which are in hydrophobic matching conditions (see also Section 2). (**a**) n-side (electronegative, cytoplasmic in cyanobacteria, stromal in the chloroplasts of algae and plants) and front-side membrane view of the surface-covered protein compartment of the cyt*b*_6_*f* dimer with five color-coded groups of lipid-binding sites, chlorophyll *a* (Chl*a*) and β-carotene (β-Car) in stick-and-ball style, and the hemes in stick style. The black line is the protein–protein axis of symmetry. The red line, 25° to the left of the black one, represents the estimated lipid–lipid axis of symmetry based on the distribution of lipids between monomer 1 and monomer 2. The Supplementary Material S2 (Suppl_Images-1.pdf) provides a scrolling 3D visualization of (**a**) with an indicated Qp-site (the site of plastoquinol (PQH_2_) oxidation) and Qn-site (the site of plastoquinone (PQ) reduction), as well as other details related to the present study. Additionally, the assignment of bound lipids to three types is also visualized: annular, partially shielded annular, and core–annular. (**b**) Front membrane view of the cyt*b*_6_*f* dimer after a 40° rotation to the left with color codes for the eight cyt*b*_6_*f* subunits, the five groups of lipid-binding sites, and an enlarged view of the specific-to-cyt*b*_6_*f* Chl*a*, β-Car, and heme *c*_n_, the key structural components, and their n- and p-side essential residue pair distances used to follow the cyt*b*_6_*f* conformational changes related to the induction of state transitions [53]. The α- and β-axial ligand sites to the Mg^2+^ ion in the center of the chlorin ring of Chl*a*, as well as the ester carbonyl oxygen O1D of the carbomethoxy group bound to the fifth cyclopentanone ring of the chlorin macrocycle of Chl*a*, are also shown. In the enlarged view in the (**b**) panel, the cyt*b*_6_*f*-specific Chl*a*, β-Car, and amino acid residues Phe40, Leu76, Phe124 (suIV), and Ala147 (cyt*b*_6_) are displayed in ball-and-stick style. A stick style is used for the common *b*_p_ and *b*_n_ hemes with cyt*bc*_1_, heme *f*, equivalent to heme *c*_1_ in cyt*bc*_1_, and heme *c*_n_ specific to cyt*b*_6_*f*. (**c**) Electronegative (cytoplasmic in cyanobacteria/stromal in chloroplasts, n-side) and electropositive (lumen, p-side) membrane view of (**b**). The structure is presented in a depth cue view in panels (**b**,**c**). A schematic display style is used for cyt*b*_6_*f* subunits, while a CPK (space-filling) style is employed for lipids and other non-protein molecules, except for the hemes, which are depicted in stick style, and the [2Fe-2S] cluster, which is shown in ball-and-stick style. The Supplementary Video S1 Material (SupplVideo1.mp4) provides a 3D visualization of Figure 2b.

The analysis presented in this work utilizes all 13 crystal structures of dimeric cyt*b*_6_*f* available, providing a choice of the most suitable structure that represents the conformational state of cyt*b*_6_*f* under optimal, stationary conditions for photosynthesis in low-intensity light conditions. As noted above (Section 3.1), such a reference structure should be between the 1VF5 and 2ZT9 structures. The structure 4OGQ was chosen (Figure 2) because the three key parameters characterizing the conformational state of cyt*b*_6_*f*—the p-gate width, the n-side flexibility distance d_n_, and the hydrophobic thickness d_P_—fall most closely between the parameters of the 1VF5 and 2ZT9 structures (Table S2). Moreover, the 4OGQ hydrophobic thickness of 30.6 Å is the closest to the estimated average hydrophobic thickness for thylakoid proteins of 30.7 ± 2.1 Å reported in [133]. Notably, the hydrophobic thickness of the modeled spinach thylakoid membrane at the optimal temperature of 23 °C is precisely that of 4OGQ [134]. Fortunately, this 4OGQ structure possesses a maximum of 22 lipid-binding sites per cyt*b*_6_*f* monomer that have been identified so far [96]. The lipid-binding sites in the 4OGQ structure have been previously described, including all amino acid residues and their subunits at contact distances of less than 4 Å, depending on their location—boundary, boundary/cavity, and cavity [96]. In the present work, the lipid-binding sites are analyzed from a different viewpoint concerning (i) the dynamics of their occupation and interactions with the already identified main components of cyt*b*_6_*f* involved in state transitions (Chl*a* and the central, key residues Phe/Tyr124*^fg^*^-loop-suIV^ and Phe40^E-helix-suIV^ [53]) and (ii) their contacts with each other and with the bulk lipid phase (Figure 2 and Supplementary Material S2 (Suppl_Images-1.pdf)).

#### 3.2.1. Five Groups of Lipid-Binding Sites

**Figure 3:**
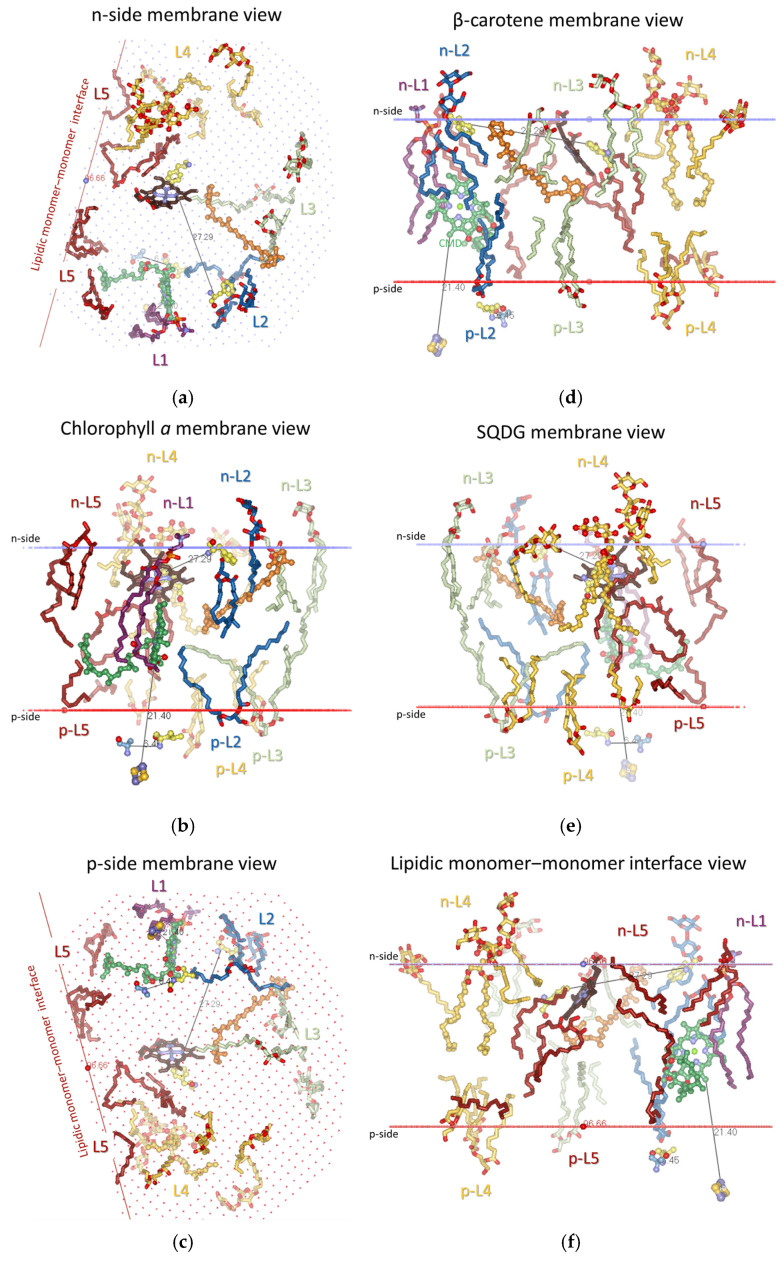
Five lipid groups form ellipse-shaped bilayer shells around each cyt*b*_6_*f* monomer in Figure 2. (**a**) n-side membrane view as in Figure 2c, but the protein chains are hidden. (**b**) Chl*a* membrane view (90° rotation of (**a**) upward); Chl*a*-governed n-/p-L1 and n-/p-L2 lipid-binding sites are in front; (**c**) p-side membrane view (90° rotation of (**b**) upward). (**d**) The β-Car membrane view (90° rotation of (**b**) to the left) and the β-Car-related n-/p-L3 sites are in front. (**e**) SQDG dominated the group of the n-/p-L4 lipid site region (next 90° rotation of (**d**) to the left). (**f**) cyt*b*_6_*f* lipid–lipid intermonomer interface region of lipid-binding sites n-/p-L5 (last 90° rotation of (**e**) to the left). Chl*a*, β-Car, SQDG, [2Fe-2S] cluster, and the two residue pairs Ala147-Leu76 and Phe40-Phe124 are displayed in ball-and-stick style; all others are in stick style. The structures are presented in a depth cue view. Supplementary Video S2 Material (SupplVideo2.mp4) provides a 3D visualization of Figure 3.

To follow the changes in the lipid occupation and interactions with Chl*a*, Phe/Tyr124*^fg^*^-loop-suIV^, Car, SQDG, and Phe40^E-helix-suIV^ during the conformational changes in cyt*b*_6_*f* related to the induction of state transitions [53], the 22 lipids/detergents/hydrocarbon chains of the 4OGQ structure were divided into five groups of binding sites (L1–L5, see Figure 2 and Figure 3 and Table S2, first 4OGQ row). They form a lipid bilayer shell covering the cyt*b*_6_*f* monomer, and each group of sites has n- and/or p-components (Figure 3, Supplementary Video S2 Material (SupplVideo2.mp4)).

The groups are numbered consecutively (Figure 3a,c), starting with Chl*a* (L1 and L2 sites, Figure 3b) and continuing to β-Car (L3 sites, Figure 3d), SQDG (L4 sites, Figure 3e), and the intermonomer region (L5 sites, Figure 3f), wherein Phe40 is accessible until the lipid bilayer shell is closed near the β-side of Chl*a* in a tic-tack manner (Figure 2 and Figure 3, Table S2). In this way, the present analysis does not duplicate lipid descriptions belonging to more than one group of sites [96]. In addition, such grouping is convenient for the rapid visualization of the respective lipid bilayer elements (n- and p-sides of each Li group) and valuable for tracking any communication between different Li sites in both transmembrane and lateral directions.

As seen in Table S2, the first 4OGQ row, each group has a different number of sites (numbered in brackets), and the lipid bilayer shell has n- and p-side elements and is one-molecule thick, covering the whole cyt*b*_6_*f* monomer (Figure 2 and Figure 3 and their Supplementary Materials S2 (Suppl_Images-1.pdf), Supplementary Video S1 Material (SupplVideo1.mp4), and Supplementary Video S2 Material (SupplVideo2.mp4). A detailed description of the five groups of lipid-binding sites in X-ray crystal structures is provided in Text S2 in the Supporting Information File S1 (Supp_Data.pdf). The lipid-binding site group numbering begins with the lipids surrounding Chl*a*, as the single Chl*a* molecule is the most essential player in cyt*b*_6_*f* function during state transitions [53]. It is the crucial redox sensor and transmembrane signal transmitter [53].

One may ask why two groups of lipid-binding sites are located in the vicinity of Chl*a*. This is because Chl*a* is not a planar molecule, and it has six chiral centers. The six chiral centers are C2A and C3A at the pyrrole ring A (PDB numbering); C8 and C13 at the phytyl chain; CBD at the cyclopentanone ring; and the Mg^2+^ ion at the center of the tetrapyrrole macrocycle, which has one or two axial ligands (see, e.g., [135] and refs therein). Lipid binding to the β-side and α-side of the Chl*a* macrocycle provides utterly different information to the lipids from the L1 and L2 groups. The n-L1 lipid (DOPC in 4OGQ) is bound to the β-side of the Chl*a* macrocycle plane (see Figure 2b and Figure 3b). The β-side refers to the side of the Chl*a* macrocycle, wherein the phytyl chain of Chl*a* binds to the propionic acid at the C2A chiral center of the pyrrole ring A. The *sn*-1 chain (i.e., the chain bound to the non-chiral glycerol backbone carbon of the lipid) interacts only with non-chiral atoms of the Chl*a* macrocycle (Figure 3b), mainly of the pyrrole ring C (CHC, C1C, C4C, and CHD). The n-L1 lipid head connects the residues Asn118 *^fg^*^-loop-suIV^, Val128 *^fg^*^-loop-suIV^, and Ala129^G-helix-suIV^ via H-bonds at the n-side of cyt*b*_6_*f* with the contacted Chl*a* macrocycle atoms. Phe/Tyr124*^fg^*^-loop-suIV^ is situated between these residues, which serve as communication contacts between Chl*a* and Phe/Tyr124*^fg^*^-loop-suIV^. The α-side is the opposite of the β-side and is the side where the carbomethoxy group is located at the CBD chiral center (see Figure 2b and Figure 3b). In contrast to n-L1, the p-L2(1) DAG lipid (2WA101) chain contacts a chiral Chl*a* atomic group, the ester carbonyl oxygen O1D, which is part of the carbomethoxy group bound to the CBD chiral center. As shown in Table S2, the p-L2(1) occupants in several structures come into contact with another chiral center of Chl*a*—the Mg^2+^ ion. Thus, the critical difference between the L1 and L2 sites is that the chain of the p-L2(1) occupant contacts exclusively chiral Chl*a* atoms, while the chain of n-L1 contacts non-chiral Chl*a* atoms.

One may also ask why SQDG was chosen as a group L4 dominator. This is because SQDG is a specific occupant, most strongly bound to its specific binding site, n-L4(1). The SQDG lipid occupies this site in almost all structures except those assigned to the induction phase of the transition to State 1 (detailed below). Moreover, this is a specific SQDG site that the other anionic lipid, PG, cannot occupy; when PG is in excess during crystallization [113], it cannot substitute for only the SQDG lipid (structure 4I7Z).

#### 3.2.2. All Lipid-Binding Sites of Cytochrome *b*_6_*f* Contact the Bulk Lipid Phase

As seen in Figure 2 and Supplementary Material S2 (Suppl_Images-1.pdf), almost all (19 of a total of 22 per monomer) lipids, detergents, and hydrocarbon chains are fully visible on the protein dimer surface, including those on the surface of the protein that is part of the intermonomer cavity. As also seen from the lipids of the cyt*b*_6_*f* monomer structure (Figure 3, Supplementary Video S2 Material (SupplVideo2)), these 19 annular lipids [136,137] form an ellipse-shaped bilayer shell around the monomer. The remaining three lipids in cyt*b*_6_*f* could be considered as “partially shielded” since only one part of the two lipid chains is shielded from the bulk lipid phase by protein residues or subunits. This characteristic of the cyt*b*_6_*f* lipids contrasts with PSII and PSI, which possess several lipid-binding sites that are fully shielded by protein subunits from contact with the bulk lipids [138]. The three partially shielded lipid-binding sites are (1) p-L2(1)—a lipid (2WA101) chain end contacts (<4 Å) the α-side of the Chl*a* macrocycle (best seen in Slides 17–22 in the Supplementary Material S2 (Suppl_Images-1.pdf)); (2) p-L3(1)—a lipid (3WM101) chain end contacts the side carbon of the ionone ring of β-Car that is buried in the protein core (best seen in Slides 17–22 from the Supplementary Material S2 (Suppl_Images-1.pdf)); and (3) n-L5(4)—an 18-C-atom-long hydrocarbon (8K6307). The 8K6307 on the dimer protein surface (Slides 8 and 25 from Supplementary Material S2 (Suppl_Images-1.pdf)) is not fully visible, whereas it is fully visible on the monomer protein surface (Slides 36–46 in Supplementary Material S2 (Suppl_Images-1.pdf)). Behind the shielding residues, the three partially visible chains of p-L2(1), p-L3(1), and n-L5(4) interact with another molecule of non-protein origin—Chl*a*, β-Car, and a detergent, respectively.

The annular n-L5(2) site is occupied by the DAG lipid 2WM309 in 4OGQ (Slide 11 in Supplementary Material S2 (Suppl_Images-1.pdf)). It has an unusual lipid position—the head group and the glycerol backbone are entirely inside the hydrophobic bilayer core. The chains are not more or less parallel to the bilayer membrane normal but are partly parallel to the membrane plane. This lipid-binding mode resembles an integral [139] or deep [140] lipid-binding site. However, the lipid binding is neither fully integral, as it does not reside within a membrane protein (as defined in [139]), nor deep, as it does not involve H-bonding (as defined in [140]). An appropriate name could be a core–annular lipid-binding site. This name indicates that this site is entirely within the hydrophobic membrane core (the lipid bilayer region characterized by the hydrophobic thickness of the bilayer), on the surface of the transmembrane protein, and is not involved in H-bonding interactions.

The above-documented observations show that in a stationary, optimal state for photosynthesis under low light intensity when the hydrophobic thickness of cyt*b*_6_*f* is the same as that of the host lipid bilayer (i.e., under hydrophobic matching conditions), practically all the available lipid-binding sites on cyt*b*_6_*f* are exposed to contact with the bulk lipid phase. Therefore, one can conclude that they can be exchanged with lipids from the bulk phase, or the occupant can quickly release/escape its binding site (i) during the induction phase of the transition to State 2 when the hydrophobic thickness of cyt*b*_6_*f* reaches its maximum and maximum positive hydrophobic mismatch is reached and (ii) during the induction phase of the transition to State 1 when the hydrophobic thickness of cyt*b*_6_*f* reaches its minimum and maximum negative hydrophobic mismatch is reached.

### 3.3. Variable Occupation of the Lipid-Binding Sites in the Diverse Cytochrome b_6_f Crystal Structures During Induction of the Transition to State 2 and State 1

Thanks to the ordering of the X-ray crystal structures in a time sequence corresponding to different redox states of the PQ pool [53], it was possible to follow the interplay between the conformational states of cyt*b*_6_*f* and the bound lipids throughout both state transition induction processes. Through the visual inspection of the superimposition of each of the structures over the structure 4OGQ, i.e., the optimal stationary state cyt*b*_6_*f* structure, which also has the maximal number of binding sites [96], it was determined for each structure which lipid-binding sites are occupied and whether there is an exchange or escape of the bound lipids. The fact that the bilayer and hydrophobic thicknesses around a protein can be measured from the crystallographic structures using the resolved annular lipids [30,141,142] is the rationale behind the belief that the mobile lipids that are not resolved by these structures can be excluded from the HMM model.

Table S2 lists the n-side and p-side lipid, detergent, and hydrocarbon occupants of the corresponding binding site groups for X-ray crystal structures. The ordering of the structures begins with the reference structure 4OGQ. It continues in the direction of increasing values for the three characteristic distances: the width of the p-side lateral gate, the n-side flexibility distance d_n_, and the cyt*b*_6_*f* hydrophobic thickness d_P_ [53]. Such ordering corresponds to consecutive snapshots of cyt*b*_6_*f* structures during the induction of the transition to State 2 because the increase in the p-gate width, n-side distance d_n_, and the hydrophobic thickness d_P_ in the crystal structures of cyt*b*_6_*f* reflects the increase in the reduction level of the PQ pool [53]. Then, the last two structures, which are asymmetric dimers, are presented in a direction that mimics the sequence of structural changes and dynamics of lipid-binding site occupation in cyt*b*_6_*f* during the induction of the transition to State 1.

Text S2 provides a detailed description of the occupation dynamics of each lipid-binding site. Following the analogous description of the occupation changes in the cryo-EM structures (Table S3), a systematic summary of the results related to the exchange and/or escape of the lipid-binding sites is provided below (in Section 3.5).

#### 3.3.1. Lipid-Binding Changes During the Induction of Transition to State 2

During the induction phase of the transition to State 2, the three characteristic distances increase as follows: p-gate (6.45–8.4 Å), dn-distance (27.3–28.5 Å), and d_P_ (30.6–32 Å). During the induction phase to State 2, the n-L1 occupant OPC does not escape its position. The native lipid at this site should be PG, as evidenced by the structure with PG used for crystallization (12-4I7Z) instead of OPC in the other structures. Cryo-EM structures resolve the same PG at this site (Table S3, Text S3, see below). Next, the occupants of p-L2(1) and p-L3(1) (the two partially shielded annular lipid-binding sites) are exchanged by MGDG at an over-reduced state structure without artificially added lipids (14-1Q90 structure). Observation of the cryo-EM structures has shown that the exchange of PG with MGDG occurs at a smaller p-gate but when the hydrophobic thickness increases from 31.4 Å to 32.6 Å—the maximal d_P_ for the available cryo-EM structures. n-L3(1) is occupied only at the p-gate up to 6.6 Å; then, its occupant escapes. n-L4(1) is always occupied by SQDG, even when PG is used for crystallization. For p-L4(1), although the occupant is changeable (Text S2), the site is always occupied, most probably by PG, as evidenced by the 12-4I7Z and the cryo-EM structures (Table S3, Text S3, see below). n-L4(2) and n-L4(3) are always occupied by a detergent, as is the third partially shielded n-L5(4) site. Note that in cryo-EM structures, n-L4(3) is occupied by MGDG when the p-gate is ≥7.2 Å. To sum up, the lipid type expected to sort around and bind to cyt*b*_6_*f* upon the over-reduced state of the PQ pool being reached and the hydrophobic thickness of cyt*b*_6_*f* reaching its maximum is MGDG. At least three MGDG molecules exchange the PG occupants at sites p-L2(1), p-L3(1), and n-L4(3).

**Figure 4:**
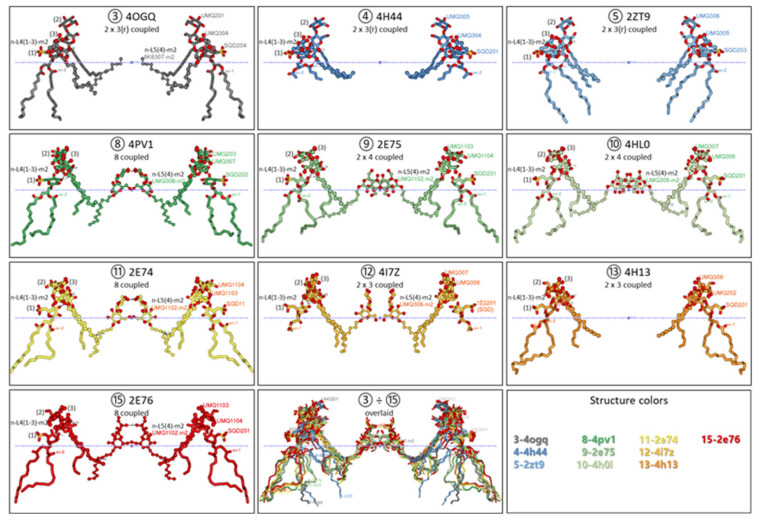
Dynamic changes in lipid conformations and lipid–lipid contacts at the n-side of the dimer interface in cyt*b*_6_*f* X-ray structures ordered in the direction of increasing p-gate width. The ordering of structures mimics time-ordered snapshots during the induction phase of the transition to State 2, corresponding to a progression from a more oxidized (3-4OGQ, p-gate = 6.45 Å) to an over-reduced state (15-2E76, p-gate = 8.4 Å) of the PQ pool. Shown are the occupants of n-L4(1) site (thick stick)—SQDG; n-L4(2) site (thick stick)—a detergent which is always bound to SQDG; n-L4(3) site (thick stick and ball)—a detergent that is not always bound to SQDG; and n-L5(4) site (thin stick and ball)—a detergent that is closer to the center of the dimer interface. The numbering of structures is as in Table S2. The last two slides show the ten structures overlaid (3–15) and their rainbow color coding, respectively. The details can be viewed by zooming (up to 500%).

Figure 4 represents the dynamic behavior of the n-side lipid–lipid dimer interface in a sequence of cyt*b*_6_*f* dimer structures that mimic consecutive conformational changes upon going from the optimal stationary state (3-4OGQ, p-gate = 6.45 Å) to the over-reduced state (15-2E76, p-gate = 8.4 Å) of the PQ pool. Included are the three n-L4(1–3) sites and the n-L5(4) site. The first row represents structures with a p-gate from 6.45 Å to 6.9 Å. The three n-L4(1–3) occupants in each cyt*b*_6_*f* monomer form ring-coupled trimers (2 × 3(r) coupled), wherein each molecule contacts the other two. The most characteristic is the continuous movement of n-L4(1) and n-L4(3) chains in a direction closer to the membrane interface and its center. The second row includes structures with a p-gate width increase from 7.0 to 7.2 Å. The most characteristic is the break of the n-L4(1)–n-L4(3) contact and formation of eight coupled lipid–detergent molecules (8-4PV1, p-gate 7 Å, d_P_ 31.8 Å). The eight molecules are coupled due to the sufficient movement of the n-L4(3) chain, which allows it to contact the chain of the n-L5(4) occupant from the other monomer, as well as the head–head (O4-O4) contact between n-L5(4) from one monomer and that from the other monomer. After that, the eight-lipid-composed nanodomain is converted into two separate three-membered lipid clusters, and then this sequence is repeated twice. The fact that the two n-L5(4) detergents contact only when each of them comes into contact with n-L4(3) detergent indicates that their contact is stabilized by contact with the neighboring n-L4(3) occupant.

The observed different directions of movement of lipids in each of the three groups explain the oscillating formation of eight coupled molecules at the inflection point of the chain’s movements. The almost identical d_P_ hydrophobic thickness above the p-gate width of 7.0 Å (see Table S2) is consistent with the polynomial dependence of the hydrophobic thickness d_P_ on the reduction level of the PQ pool (Figure 6C in [53]). It also aligns with the curvilinear dependence between the redox state of the PQ pool and the extent of state transitions [61]. As demonstrated in [61], the transition to State 2 can start even at the moderately reduced PQ pool redox state. This is in contrast to the transition to State 1, which can begin after the over-oxidation of the PQ pool [61].

#### 3.3.2. Lipid-Binding Changes During the Induction of Transition to State 1

During the induction phase of the transition to State 1, the three characteristic distances decrease as follows: p-gate (6.45 to 5.5 Å), dn-distance (27.3 to 21.8 Å), and d_P_ (30.6 to 28.6 Å). In contrast to the induction phase of the transition to State 2, the induction phase of the transition to State 1 is characterized by more significant changes in the cyt*b*_6_*f* conformation and the occupation of the lipid-binding sites (Table S2, last four rows). This is the region of substantial change in the position and orientation of the aromatic ring of the key *fg*-loop Phe124 residue from membrane-buried to n-side-exposed. It has been deduced from X-ray structure analysis [53] that the two dimer structures (1VF5 and 2D2C) and their monomers are snapshots of cyt*b*_6_*f* conformational changes during the induction of the transition to State 1. As can be seen from Table S2, starting from the structure 4OGQ with d_P_ = 30.6 Å, d_P_ decreases to 29.8 Å (1VF5) and then to 28.6 Å (2D2C). This decrease in d_P_ corresponds to the steeper, linear part of the relation between the volume of Chl*a* (a measure of the redox state of the PQ pool) and the cyt*b*_6_*f* hydrophobic thickness d_P_ (Figure 6C in [53]). A substantial decrease in the n-side distance parallels it. Their monomers have different p-gate widths and n-side distances, but their average values follow the same direction of decline relative to the values of the p-gate width and d_n_-distance of the 4OGQ structure. There are only two resolved lipids in these structures. The first OPC lipid occupies an intermediate pose in the n-L4(1–3) sites of SQDG + 2UMQ in all other structures. The second OPC lipid occurs only at n-L4 sites, and the core–annular lipid occurs at n-/p-L5(2).

**Figure 5:**
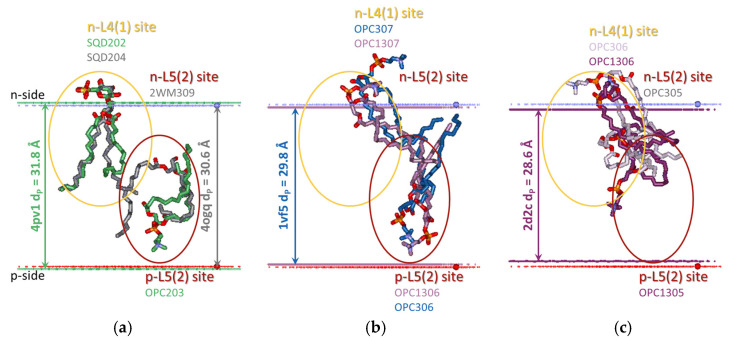
Dynamic changes in the position and conformation of lipid molecules in n-L4(1) and n/p-L5(2) sites were identified as important upon the induction of the transition to State 1 (see Section 3.5 below). The OPM-estimated cyt*b*_6_*f* hydrophobic thicknesses d_P_ [97] are shown. (**a**) SQDG and DAG in structure 4PV1 (Table S2) and SQDG and DOPC in the reference structure 4OGQ for the stationary state, (**b**) the two overlaid DOPC from the two monomers of the asymmetric dimer 1VF5, and (**c**) the two overlaid DOPC from the two monomers of the asymmetric dimer 2D2C. Note the strongly shifted pose of DOPC at n-L4(1–3) sites in (**b**,**c**) relative to SQDG at n-L4(1) sites in (**a**). Notably, the distance between the two DOPC lipids in the 2D2C structure (**c**) is also diminished. Supplementary Material is provided to show their location relative to the other occupants in 11 structures (see Slides 62–95 in Supplementary Material S3 (Suppl_Images-2.pdf)).

Figure 5 compares the lipid-binding sites n-L4(1) and n-L5(2) of four dimer structures with different hydrophobic thicknesses. Note that these lipid sites are also surface-bound and are accessible from the intermonomer cavity. More extensive changes in these two lipid occupants are observed during the induction phase of the transition to State 1 than during the induction phase of the transition to State 2. The orientation of the chains of DOPC in n-L4(1–3) towards closer to the n-side membrane plane, as well as the movement of the core–annular n/p-L5(2) DOPC lipid closer to the n-side leaflet, is the lipid dynamic response to the diminished hydrophobic thickness of cyt*b*_6_*f* during the induction phase of the transition to State 1.

The emptying of most of the lipid-binding sites is characteristic of the induction phase of the transition to State 1. However, it is impossible to distinguish which emptied sites accompanied the decreased hydrophobic thickness and contributed to the temporal minimization of the negative hydrophobic mismatch induced by cyt*b*_6_*f*. As shown in Table S3 with the cryo-EM structures below (Section 3.4.3), lipids from the n-L4(1) and p-L3(1) sites are the first to escape their binding sites, followed by those from n-L1(1), n-L2(1–2), and p-L2(1).

### 3.4. Comparison of the Lipid-Binding Sites in Cryo-EM and X-Ray Crystal Structures

As noted above, the cryo-EM structures (Table S3, Text S3) are indispensable for identifying the native lipid occupants in most of the lipid-binding sites of the X-ray crystal structures. Moreover, the cryo-EM structures can reveal several new characteristics of the cyt*b*_6_*f*–lipid interplay, which are detailed below.

#### 3.4.1. Lipid-Binding Sites in X-Ray Crystal Structures Are Not Affected by Crystal Packing

Similar to Table S2, Table S3 presents lipid-binding sites in the different structures resolved by the cryo-EM method. Here, the dimer structures are ordered toward increasing p-gate width, corresponding to an increase in the reduction level of the substrate/inhibitor of cyt*b*_6_*f*. Text S3 provides a detailed description of the dynamics of site occupation during the Q-cycle in inverse order. Overall, the lipids in the cryo-EM structures exhibit more pronounced movements from the binding sites defined by the 3-4OGQ crystal structure than the lipids in the X-ray structures (see comments in brackets in Table S3). Nevertheless, the occupants are well resolved as different binding sites, at least because the distance between the C2 atoms of their glycerol backbone is more than 10 Å. For instance, this distance is 12–14 Å for the p-L4(1, 3, 4) sites. It is also important to note that the lipids in both the X-ray and cryo-EM structures are bound at almost the same sites on cyt*b*_6_*f*, indicating that they are discrete binding sites and, therefore, the lipid-binding sites in the X-ray structures are not affected by crystal packing. In addition, the cryo-EM structures possess intermediate poses between two lipid-binding sites in the groups L4 and L5 (n-L4(1) + n-L4(3) and p-L5(2) + p-L4(3), Table S3), just as the X-ray crystal structures 1VF5 and 2D2C (n-L4(1–3) and n-L5(1–2), Table S2). These intermediate poses undoubtedly represent the lipid translocation pathways for escape/exchange, captured on both millisecond ([95,129,130] and Section 3.1.3) and second ([53,61] and Section 3.1.3) timescales (see below, Section 3.4.4). Notably, some cryo-EM structures exhibit lipid, detergent, and hydrocarbon poses that are absent from crystal structures. These are p-L1(1), n-L2(1–2), p-L3(3), and p-L4(5) (Table S3), and they likely represent transient states accessible only in the millisecond time range.

#### 3.4.2. Cytoplasmic/Stromal Surface-Bound Peripheral Proteins Do Not Affect the Hydrophobic Thickness of Cytochrome *b*_6_*f*

There are two pairs of cryo-EM structures without and with peripheral bound proteins—the cyanobacterial structures 7ZXY(-PetP) and 7R0W(+PetP) without and with the PetP cyt*b*_6_*f* subunit [116] and the spinach structures 7QRM(-TSP9) and 7ZYV(+TSP9) without and with the TSP9 fragment [117]. Both subunits bind at the same cytoplasmic/stromal part of cyt*b*_6_*f* [95]. The difference between the cyanobacterial 7ZXY(-PetP) and 7R0W(+PetP) structures lies in the values of the hydrophobic thickness dP (31.4 Å, the minimal value, vs. 32.6 Å, the maximal value). However, the difference in the hydrophobic thickness between them should not be due to the bound peripheral protein because the spinach structures 7QRM(-TSP9) and 7ZYV(+TSP9) have practically equal hydrophobic thicknesses (32.2 Å and 32.4 Å) to that of the thicker (32.6 Å) cyanobacterial structure 7R0W(+PetP). One may conclude that the binding of peripheral subunits is not a reason for a change in the hydrophobic thickness of cyt*b*_6_*f* in cryo-EM structures. Hence, it appears that cyt*b*_6_*f* can alter its hydrophobic thickness during the operation of the Q-cycle on the ms timescale. In this regard, cyt*b*_6_*f* behaves similarly to the G-protein-coupled receptor rhodopsin [143].

#### 3.4.3. The Emptying of the SQDG n-L4(1) Site and PC p-L3(1) Site Is the First Lipid Response to the Decreased Hydrophobic Thickness of Cryo-EM Cytochrome *b*_6_*f* Structures

The thicker structure 7R0W(+PetP) has two more lipids than the thinnest structure 7ZXY(-PetP) (Table S3). These are SQDG at n-L4(1) and PC at p-L3(1), and PQ occupies its n-L5 sites. Both plant structures 7qrm(-TSP9) and 7zyv(+TSP9) also have SQDG at n-L4(1) and UMQ at p-L3(1). They differ only by the presence of PG at the n-L5(5) site in 7QRM (-TSP9). However, since there is no difference between the two structures in the three characteristic distances (Table S3), it is clear that the n-L4(1) and p-L3(1) sites are the sites whose occupation by SQDG and PC accompanies the thicker cyt*b*_6_*f* structure. Thus, the missing SQDG at the n-L4(1) site and PC at the p-L3(1) site characterize the thinnest cyt*b*_6_*f* structure 7ZXY(-PetP). In the above subsection (Section 3.3.2), it was found that for X-ray crystal structures, the emptying of most lipid-binding sites is characteristic of the induction phase of the transition to State 1. Thanks to the cryo-EM structures, it is possible to distinguish that the first sites to empty responded to the decreased hydrophobic thickness, p-gate, and dn-distance, with SQDG from site n-L4(1) and PC from site p-L3(1) (Table S3). The next are the lipids from n-L1, n-L2(1–2), p-L2(1), and p-L4(1).

Based on the above comparisons, one can deduce that the emptying of the n-L4(1) and p-L3(1) sites is the lipid response to cyt*b*_6_*f* structures with a smaller hydrophobic thickness d_P_. The empty n-L4(1) site breaks the H-bonding and salt-bridge interactions characteristic of the head group of SQDG.

#### 3.4.4. SQDG Translocation Escape Pathway to the Bulk Lipid Phase: Scramblase Function of Cytochrome *b*_6_*f*?

Another interesting result from Table S3 is that one can follow the trajectory of SQDG release from the cyt*b*_6_*f* structure. Upon decreasing the p-gate from 7.2 Å (7-7QRM) to 6.8 Å (6-6RQF), SQDG releases its n-L4(1) binding site and occupies an intermediate pose n-L4(1) + n-L4(3), with a head and *sn*-1 chain almost coinciding with n-L4(1), while the *sn*-2 chain overlaps with the chain of n-L4(3). Then, at a p-gate of 6.1 Å (5-6RQF), SQDG occupies an intermediate pose p-L5(2) + p-L4(3), with the head overlapped with the p-L5(2) and P-group and the *sn*-2 chain with p-L4(3), and the chains are parallel to the p-side plane and contact the phytyl chain of Chl*a* of the second monomer. At a p-gate of 6.0–6.1 Å (1, 2-7ZXY), n-L4(1) is empty. Hence, the translocation pathway of SQDG is from the n-side bound to the p-side, escaping cyt*b*_6_*f*. It is reasonable to propose that SQDG will go to the p-leaflet of the bulk lipid bilayer phase. It is also possible, via Chl*a*, for cyt*b*_6_*f* to play a scramblase role in the flip-flop of SQDG. After escape from cyt*b*_6_*f*, the result that SQDG will go to the p-side (luminal) monolayer of the bulk bilayer is consistent with the preferential p-side leaflet distribution of SQDG in the bulk lipid phase of the thylakoid membrane [4]. The interleaflet translocation of SQDG raises the question of whether the cyt*b*_6_*f* intermonomer cavity with the phytyl chain of Chl*a* therein may scramble SQDG.

### 3.5. Predicted Native Lipid Exchange at/Escape from Cytochrome b_6_f Complex upon Induction of State Transitions

Table 1 summarizes the exchange and escape of lipid occupants during the induction phase of the transition to State 2 and State 1, where the native lipid was deduced from X-ray crystal or cryo-EM structures at the same p-gate width (see above and Text S3). At the optimal stationary state of cyt*b*_6_*f* for electron transport under low-light conditions, when there is hydrophobic matching, there is a strict dominance of PG at almost all lipid-binding sites, as observed by X-ray crystallography and cryo-EM methods. Upon the induction of the transition to State 2, the escape of PG at n-L2(1) and the exchange of PG at the p-L2(1) and p-L3(1) sites with MGDG and the n-L4(3) detergent with MGDG are most important for the transient minimization of the positive hydrophobic mismatch induced by the increasing hydrophobic thickness of cyt*b*_6_*f* during the induction of the transition to State 2. The bulk lipid phase becomes enriched with PG and depleted of MGDG. Upon the induction of the transition to State 1, first, the escape of SQDG from n-L4(1) and PG from p-L3(1) and then that of the other lipids and the binding of DGDG in place of n-L4(1–3) occupants and of n-/p-L5(2) core–annular lipid with DGDG are most important for the transient minimization of the negative hydrophobic mismatch induced by the decreasing hydrophobic thickness of cyt*b*_6_*f* during the induction of the transition to State 1. In this case, the bulk lipid phase becomes enriched with anionic SQDG and PG and depleted of DGDG.

Table 1 also shows that each of the three cyt*b*_6_*f* conformations selects its lipid environment, each distinctly different. The number of bound lipids detectable by X-ray and cryo-EM at optimal (5 PG, 1 SQDG, 1 DAG/PQ), extreme State 2 (2 PG, 3 MGDG, 1 SQDG), and extreme State 1 (2 DGDG) conditions differs, as do the lipid classes. Under hydrophobic matching conditions, the cyt*b*_6_*f* lipid nanoenvironment is enriched with PG. At maximal positive hydrophobic mismatch, it is enriched with MGDG, whereas at maximal negative hydrophobic mismatch, it is enriched with DGDG. Notably, the bound lipid composition in these three states is distinctly different from that of the bulk lipid phase, which is typical of the thylakoid lipid phase, characterized by roughly 5 MGDG, 2.5 DGDG, 1.5 SQDG, and 1.5 PG [7,8].

Altogether, the documented changes provide direct structural evidence supporting the proposed hydrophobic mismatch model for cyt*b*_6_*f*-driven state transitions [53]. They also identify the two lipid classes (MGDG and DGDG) that are important in temporarily minimizing the cyt*b*_6_*f*-induced positive (during the induction phase of the transition to State 2) and negative (during the induction phase of the transition to State 1) hydrophobic mismatch, respectively.

### 3.6. Continuous Chlorophyll a Movement and Membrane Normal Alignment upon Increasing the Reduction Level of the Plastoquinone Pool

Color coding of the different structures was applied to track the dynamics of each binding site during the induction of the transition to State 2. The increasing spectral wavelength of the structures’ colors (4–15) codes the growing value of the p-gate width (see Table S2). In this way, a direct visualization of the effect of the redox state of the cyt*b*_6_*f* substrate pool on the conformational changes and movements of the non-protein components can be followed. If there are dispersed colors, this could be ascribed to the inherent mobility of the lipids.

**Figure 6:**
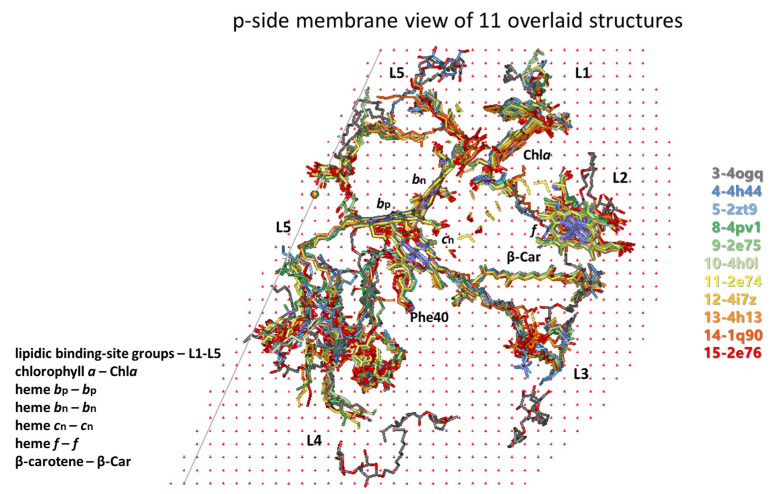
Representation of eleven overlaid monomeric cyt*b*_6_*f* crystal structures, with only the non-protein compartment shown. These structures are color-coded—the monotonic increase in the spectral wavelength of the color corresponds to the monotonic rise in the p-gate width from 6.45 Å to 8.4 Å (see Table S2). The structures were prior aligned, as described in Section 2. In this way, the dynamics of the lipid binding in the five groups L1–L5 can be followed and analyzed. Supplementary Video S3 Material (SupplVideo3.mp4) provides a 3D visualization of Figure 6. Supplementary Material S3 (Suppl_Images-2.pdf) provides a scrolling 3D detailed visualization of each of the 22 binding sites resolved in 3-4OGQ during the induction of the transition to State 2 and the induction of the transition to State 1. The latter involves the four other monomeric cyt*b*_6_*f* structures of 1VF5 and 2D2C, superimposed over the former ones.

Figure 6 shows eleven overlaid snapshots with an increasing p-gate width from 6.45 Å to 8.4 Å. Supplementary Video S3 Material (SupplVideo3.mp4) provides a 3D visualization of Figure 6. Supplementary Material S3 (Suppl_Images-2.pdf) provides a scrolling 3D detailed visualization and descriptions of the mobility of each occupant in the respective site, as also listed after Table S2.

The careful inspection of the overlaid structures clearly shows that the most significant systematic difference upon the induction of the transition to State 2 is the rotation of the Chl*a* macrocycle around a vertical axis, increasing the angle between the protein dimer axis and Chl*a* from 45° (see Slide 3 in Supplementary Material S3 (Suppl_Images-2.pdf) to 50° (see Section 3.7.1 below). Slides 5–6 in Supplementary Material S3 (Suppl_Images-2.pdf) clearly show that the Chl*a* macrocycle becomes more parallel to the membrane normal at a larger hydrophobic thickness. Note that the n-L1 lipid strictly follows the movement of the Chl*a* macrocycle (Slides 5–8 in Supplementary Material S3 (Suppl_Images-2.pdf)). During the induction phase of the transition to State 2, the n-L1 lipid is always bound to Chl*a* and follows its movement and alignment to the membrane normal. Thus, during the induction phase of the transition to State 2, the *fg*-loop is controlled by Chl*a* (volume, position, untilting) mediated by the n-L1 lipid. This provides additional evidence for the signal transmission from the Chl*a* macrocycle to the *fg*-loop, which contains the key flexibility center of cyt*b*_6_*f*—the Phe/Tyr124*^fg^*^-loop-suIV^ residue.

### 3.7. Communications and Signaling Between the Three Main Players in State Transitions

Given the significant impact of Chl*a* on the lipid bilayer, with a propensity for non-lamellar phase transformations [126], the question arises as to whether Chl*a* may communicate and signal to the center of flexibility, Phe/Tyr124*^fg^*^-loop-suIV^, through the bound lipids.

#### 3.7.1. Chl*a* Signaling to Phe/Tyr124*^fg^*^-loop-suIV^ Under Optimal and Reducing Conditions: Role for β-Car

**Figure 7:**
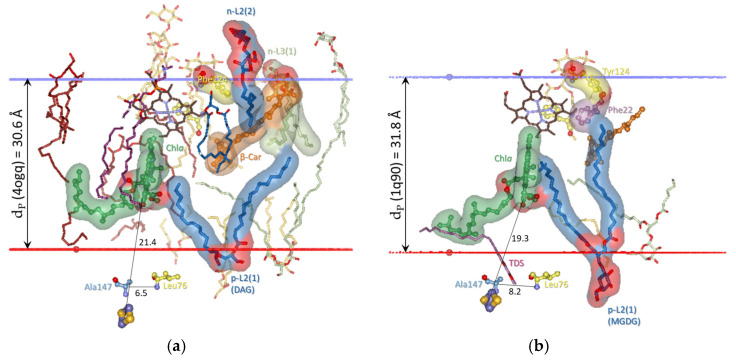
Lipid-mediated signaling pathways from chlorophyll *a* to the key residue Phe/Tyr124*^fg^*^-loop-suIV^. (**a**) The first pathway is identified in the 3-4OGQ structure of cyt*b*_6_*f*, which corresponds to an optimal oxidized state of the plastoquinone (PQ) pool (Section 3.2). (**b**) The second pathway is identified in the 14-1Q90 structure of cyt*b*_6_*f* that corresponds to the over-reduced redox state of the PQ pool [53]. Surface-covered participants mark the signaling pathways, and their names are indicated. The structures are presented in perspective projection, a depth cue, and with a 50° rotation of the front view, as shown in Figure 2a.

The first lipid-mediated molecular sequence of crystal contacts (below 4 Å) from Chl*a* to Phe124 (Figure 7a) is identified in the 3-4OGQ structure of cyt*b*_6_*f* (Table S2). This structure mimics cyt*b*_6_*f* in situ when the PQ pool is oxidized (PQ > PQH_2_), as in the stationary State 1 or State 2 ([53] and Figure 6C therein).

The signaling pathway under optimal conditions for electron transport, i.e., under hydrophobic matching conditions (Figure 7a), also involves β-Car as follows: Chl*a* (O1D) → p-L2 *sn*-1 chain of 2WA101(15:0/17:1) and then the *sn*-2 chain of 2WA101 → n-L3(1) *sn*-2 chain of 7PH104 (3 C atoms in contact) → n-side C39-ring carbon and three-chain carbons of β-Car (BCR102) and then n-side C38-ring carbon and C37-chain carbon of β-Car → n-L2(2) chain of UMQ101 → CE1-phenyl ring carbon of Phe124. This sequence of crystal contacts occurs between the participants’ hydrocarbon chains, which can be linear or cyclic. It originates from a Chl*a* atomic group bound to one (CBD atom) of the six chiral centers of Chl*a* ([135] and refs therein). Moreover, the following two DAG lipids—2WA101 and 7PH104—also have a chiral center (the C2 atom of the glycerol backbone). The transmembrane contact between them is via the terminal methyl carbons of their *sn*-2 chains and obeys a selection rule for the same chain stereochemistry (*sn*-2). The next β-Car and the detergent have no chiral centers. However, it is known that Car can even induce chirality in a non-chiral sequence of molecules [144]. Therefore, the identified molecular sequence of contacts may exhibit common, collective low-frequency vibrations (phonons) distinct from the low-frequency vibrations of the other hydrocarbon chains in the cyt*b*_6_*f* complex. Below, it will be shown that this signaling pathway is part of a more extensive network of chain–chain lipid contacts, which have common, collective low-frequency vibrations (phonons). The signaling originates from a chiral center in Chl*a*, ensuring unidirectional signal propagation. This molecular sequence of signal transfer also deciphers a role for the Car molecule for the first time. It mediates the signaling from Chl*a* to the center of flexibility Phe/Tyr124*^fg^*^-loop-suIV^.

Figure 7b shows a second lipid-mediated molecular sequence of crystal contacts (below 4 Å) from Chl*a* to Tyr124 identified in the 14-1Q90 structure of algal cyt*b*_6_*f*. The algal 1Q90 X-ray crystal structure [83] with the inhibitor TDS bound at the Qp-site for plastoquinol oxidation is known to mimic the cyt*b*_6_*f* conformation when the PQ pool is over-reduced [53]. This connection between Chl*a* and Tyr124 is shorter than that when the PQ pool is oxidized (Figure 7a) and involves Chl*a* (Mg, O1D) → *sn*-2 chain of p-L2(1)-LMG953(18:0/18:0) and then the *sn*-1 lipid chain of p-L2(1)-LMG953 → aromatic ring of PetG-Phe22 → aromatic ring of suIV-Tyr124. The PetG residue Phe22 is involved in this signaling pathway instead of β-Car.

The conformation of the lipid chains in Figure 7b, compared to Figure 7a, is similar to the typical lipid response to relax positive hydrophobic mismatch—the chains are closer to one another and stretched. This is additional evidence that the lipid at the p-L2(1) site is the one that responds most clearly to dynamic hydrophobic mismatching during the continuous increase in the PQ pool reduction level.

#### 3.7.2. Under Low-Light and Optimal Stationary Conditions for Photosynthesis—Two Thylakoid Lipid Raft-like Nanodomains Around Cytochrome *b*_6_*f* Dimer

**Figure 8:**
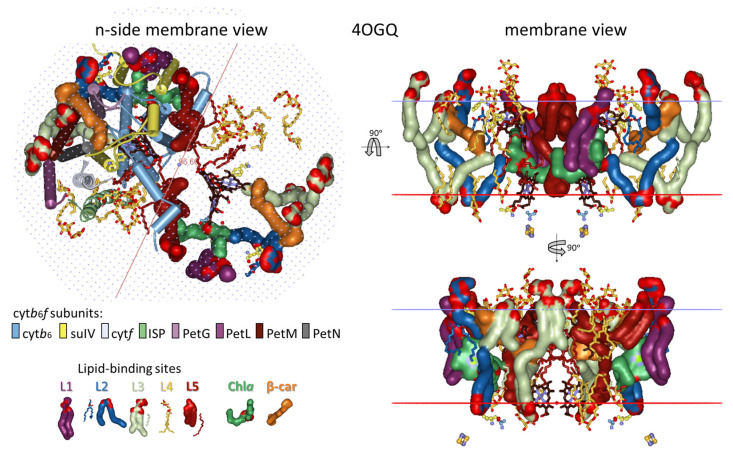
The n-side and membrane views of the location of the identified bilayer raft-like lipid nanodomain around the cyt*b*_6_*f* dimer crystal structure 4OGQ. The n-side view is obtained by rotating the front view in Figure 2 by 50° to the left. The transmembrane helices of cyt*b*_6_*f* are presented in a schematic style, except for the transmembrane helices of Rieske ISP and cytochrome *f* subunits. They are depicted in a solid ribbon style to emphasize that the lipids around them are not involved in the raft-like nanodomain. The lipids, detergents, and hydrocarbon chains, which are part of chlorophyll *a* and β-carotene-mediated hydrophobic contacts (below 4 Å), are shown in a surface-covered style. In contrast, those not involved are shown in stick style. The Supplementary Video S4 Material (SupplVideo4.mp4), which includes a 3D visualization of the raft, is provided. It features the values of the contact distances and the estimated dimensions of the identified raft-like structure.

Figure 8 shows the existence and location of two sequences of a transbilayer lipid network of contacts around the cyt*b*_6_*f* crystal dimer structure 4OGQ. Starting from the α-side of Chl*a* in the right direction, these contacts are as follows: Chl*a* (α-side, O1D) → p-L2 *sn*-1 chain (2WA101(15:0/17:1)) → p-L2 *sn*-2 chain (2WA101) → n-L3(1) *sn*-2 chain (7PH104(12:0/14:0)) → β-Car C21 at n-side (β-Car n-side C37 and ionone ring C31 in contact with the chain of the n-L2(2)-UMQ101) → β-Car buried ionone ring C31 → p-L3(1) *sn*-1 chain (3WM101(18:1/18:1)) → p-L3(1) *sn*-2 chain (3WM101) → n-L3(2) *sn*-2 chain (1O2103, MGDG(16:0/18:1)). Together with Chl*a*, the total number of molecules involved is seven. Starting from the β-side of Chl*a* in the left direction, the Chl*a* macrocycle contacts with the four atoms (C7, C8, C10, and C12) of the *sn*-1 chain of n-L1 lipid OPC205(18:1/18:1). The Chl*a*-phytyl terminal C19 contacts → n-L5(3) chain of 8K6306 (in contact with p-L5(4)-8K6308) → n-L5(4)-chain of 8K6307 → n-L5(5) *sn*-1 chain of 7PH305 (of the other monomer). A total of 12 molecules are coupled, forming a nanosized domain (nanodomain) of interacting molecules with a hydrophobic core perimeter of approx. 8 nm (78 Å). p-L5(2)-MYS202 and p-L4(3)-2WD206 can also be added. The numerous hydrocarbon chain contacts (Supplementary Video S4 Material (SupplVideo4.mp4)) form a matrix—a one-lipid-molecule-thick bilayer sheet. Since 12 molecules are connected, it is obvious that the frequency of the collective vibrations (phonons) of this matrix of connected hydrocarbons will differ from that of the unconnected hydrocarbon chains. The fact that the transmembrane contacts of lipids occur between their *sn*-2 chains suggests that, by adhering to this stereoselective rule, they are more ordered than other chain–chain-bound lipids. Based on the size of this nanodomain (within 2–20 nm range) [145] and its crystal contacts, indicative of common, collective low-frequency vibrations (phonons) [146], one may conclude that these 12 molecules form a phonon-assisted raft-like nanodomain. Moreover, the existence of ordered lipid chains obeying a chirality-selective rule (that is, *sn*-2 chain from one leaflet contacts with the *sn*-2 chain of the other leaflet) allows us to name them chiral-phonon-assisted raft-like nanodomains. Notably, chiral phonons have been registered in nano- and microcrystals of biomolecules [147].

Figure 8 also shows that the lipids around the transmembrane helices of Rieske ISP and cyt*f*—those from the L4 lipid-binding site group—are not part of these bilayer raft-like nanodomains. Notably, this nanodomain contains the p-L2 and p-L3(1) lipid-binding sites, which are partially shielded annular lipid-binding sites.

#### 3.7.3. Communication Under Reducing Conditions: The n-Side Monolayer Thylakoid Lipid Raft-Like Nanodomain

**Figure 9:**
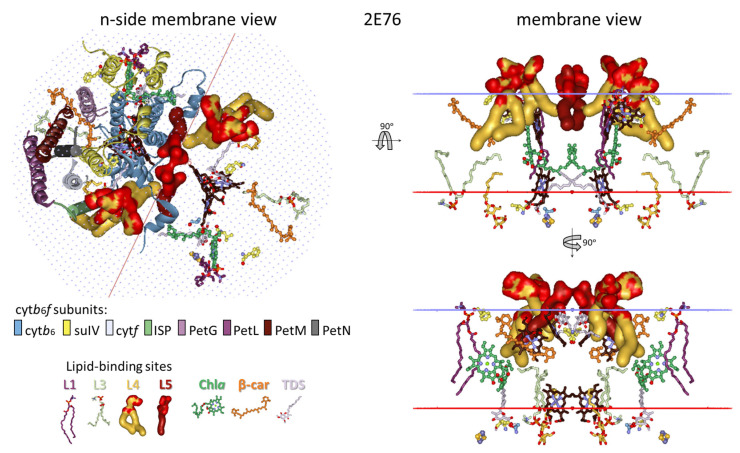
Monolayer raft-like lipid nanodomain in cyt*b*_6_*f* crystal structure 2E76. These are the eight coupled molecules in Figure 5. The transmembrane helices of cyt*b*_6_*f* are presented as solid ribbons, except for the transmembrane helix of Rieske ISP. It is depicted in a schematic style to emphasize that SQDG and the two detergent molecules around it are involved in the raft-like nanodomain. SQDG and detergents which are involved in the raft-like nanodomain are shown in a surface-covered style, while those not involved are shown in stick style. The Supplementary Video S5 Material (SupplVideo5.mp4) provides a 3D visualization of the raft-like lipid nanodomain, including the values of the contact distances and the estimated dimensions of the identified raft-like structure.

Three of the dimer structures (8-4PV1, 12-2E74, and 15-2E76) with a larger p-gate width (>7 Å) feature a cluster of eight lipid/detergent molecules with crystal contacts, designated as “8 coupled” in Table S2. These eight coupled molecules are the three n-L4(1-3) molecules (SQDG and the two detergents) and the n-L5(4) detergent from the two monomeric units of cyt*b*_6_*f*. Figure 9 shows the location of this cluster. The hydrophobic core length of the intermonomer cluster, composed of eight contacting lipid–detergent molecules, is approximately 7 nm (69.7 Å) (Supplementary Video S5 Material (SupplVideo5.mp4)). Thus, based on the size of this nanodomain [145] and its crystal contacts, indicative of common, collective low-frequency vibrations (phonons) [146], one may conclude that these eight molecules form a phonon-assisted raft-like nanodomain.

Interestingly, the lipid-binding sites involved in the bilayer raft-like nanodomain (Figure 8) are not engaged in this monolayer raft-like nanodomain at the n-side (stroma side) (Figure 9). Notably, as in the case of the bilayer raft-like nanodomain (Figure 8), this monolayer raft-like nanodomain also features a partially shielded binding site, the n-L5(4) sites of the two monomers.

## 4. Discussion

### 4.1. Cytochrome b_6_f-Induced Positive and Negative Hydrophobic Mismatch Operates in a Time Range of a Few Seconds

In previous work [53], it was proposed that the sensor and transmembrane signal transduction role of Chl*a* in cyt*b*_6_*f*, which reflects changes in the redox state of the PQ pool during the induction phases of state transitions, is active on longer timescales but not on the millisecond timescale of the Q-cycle operation. This was proposed based on the absence of signal transmission from the Qo(p)-site to the n-side of cyt*bc*_1_. The present work (Section 3.1.3) provides direct structural evidence that signal sensing and transduction occur on a much longer timescale of seconds. This is a very strong, negative linear correlation between the p-gate width and the position of the [2Fe-2S] cluster relative to the Mg^2+^ of Chl*a*, as observed in X-ray crystal structures but absent in cryo-EM structures (see Figure 1). The presented direct structural evidence (Figure 1) is significant because it convincingly establishes that the cyt*b*_6_*f*-induced hydrophobic mismatch during the induction of state transitions occurs on a timescale of seconds. This is the first direct structural demonstration that the hydrophobic mismatch can be a driving force for protein reorganization on a timescale of seconds, providing information for the first time on the contribution of hydrophobic mismatch to dynamic protein signaling within a time window of seconds.

### 4.2. An Idea of How the Volume of the Chlorophyllide Part of Chlorophyll a Could Sense the Position of the [2Fe-2S] Cluster Relative to the Mg of Chlorophyll a

Previous work [53] did not explain how the Chl*a* volume could sense the position of the [2Fe-2S] center relative to Chl*a*. The (i) direct structural evidence that the distance of [2Fe-2S] to Chl*a* senses the redox state of the PQ pool only in the time range of a few seconds (Figure 1) and (ii) the shortest Mg–[2Fe-2S] distance among the metal centers [53], as well as (iii) the sensitivity of the chlorophyllide *a* molecular volume to this distance [53], borrows the following speculative explanation of how the changes in the Mg–[2Fe-2S] distance can lead to changes in the volume of the Chl*a* macrocycle: A single Chl*a* molecule (i.e., as is the monomeric Chl*a* in cyt*b*_6_*f* [126]) can absorb only ~10 photons per second with maximum sunlight [148] (p. 67). At lower sunlight intensity, such as during light state transitions, the number of absorbed photons per second will be lower. The absorbed photon, in addition to fluorescence and internal conversion events, also populates the paramagnetic triplet state of Chl*a*. The triplet lifetime of Chl*a* in cyt*b*_6_*f* and various solvents at room temperature ranges from 2 to 12 μs, with an average value of approximately 7 μs [149,150]. This paramagnetic triplet state of Chl*a* can test the distance to [2Fe-2S] by magnetic interactions with the [2Fe-2S] magnetic moment (see [151] for the [2Fe-2S] magnetic properties) at, e.g., 0.2–0.3 s. This stroboscope-like manner of Chl*a* sensing changes in the distance of [2Fe-2S] to Chl*a* would result in the altered strength of their magnetic interactions and triplet state characteristics. Since triplet state formation, decay, and quenching in an aqueous solution are accompanied by volume changes [152], changes in the Mg–[2Fe-2S] distance would be observed as a change in the Chl*a* volume in cyt*b*_6_*f*.

In this context, the observed monotonous, unidirectional rotation of the Chl*a* macrocycle (Figure 6), which occurs in synchrony with the decreasing Mg–[2Fe-2S] distance (Figure 1), is particularly noteworthy. The axis of rotation is normal to the membrane plane and is located at the point (C2A) where the phytyl chain ester binds to the Chl*a* macrocycle (Figure 6). Looking from the n-side of the membrane plane, the angle between the Chl*a* macrocycle plane and the protein interface axis increases from ~45° (see Slide 3 in Supplementary Material S2 (Suppl_Images-1.pdf)) to ~50° (Figure 7) while heme *b*_n_, heme *b*_p_, and heme *c*_n_ remain static (Figure 6). This selective rotation of the Chl*a* macrocycle could be interpreted as an alignment effect of the magnetic field of the [2Fe-2S] cluster. Looking from the membrane side (n-side above, p-side below) and along the Chl*a* macrocycle, it is seen that the slightly tilted Chl*a* macrocycle orientation relative to the membrane normal at shorter cyt*b*_6_*f* hydrophobic thickness d_P_ becomes more parallel to it at maximal cyt*b*_6_*f* d_P_ (Figure 7). As will be seen in Section 4.7 below, the same is observed when comparing the tilting angle of the Chl*a* plane relative to the membrane normal among the three structures with different hydrophobic thicknesses. At minimal d_P_ (2D2C structure), Chl*a* is most tilted relative to the membrane normal. Upon increasing d_P_ to optimal (4OGQ) and maximal (2E76) values, the plane of the Chl*a* macrocycle becomes more parallel to the membrane normal. This more perpendicular orientation of the Chl*a* macrocycle relative to the membrane plane may also contribute to the increase in the hydrophobic thickness of the cyt*b*_6_*f* complex. It is thus evident that this observation and the suggested idea for a stroboscope-like manner of Chl*a* testing changes in the position of [2Fe-2S] relative to the Mg of Chl*a* in the time range of seconds widely open the door to an untouched area for future research—the possible magnetic interactions between Chl*a* and the [2Fe-2S] cluster.

### 4.3. Lipid- and Carotenoid-Mediated Signaling Pathways from Chla to the Center of Flexibility Phe/Tyr124^fg^^-loop-suIV^ Residue

Previously, unreported crystal contacts were found between the already established [53] main players in signal transduction during the induction of state transitions—Chl*a* and Phe/Tyr124*^fg^*^-loop-suIV^. The present work demonstrates that Chl*a* may communicate and signal to the flexibility center—the Phe/Tyr124*^fg^*^-loop-suIV^ residue at the stromal/cytoplasmic side of cyt*b*_6_*f* (Figure 7)—through lipid-mediated pathways. Their crystal contact is mediated by three lipid/detergent molecules and β-Car under optimal conditions for electron transport (Figure 7a) and by a single lipid molecule and Phe22^PetG^ at a maximally reduced state of the PQ pool (Figure 7b). The communication from Chl*a* to the Phe/Tyr124*^fg^*^-loop-suIV^ residue involves chiral Chl*a* atoms, ensuring a unidirectional signaling pathway from Chl*a* to Phe/Tyr124*^fg^*^-loop-suIV^. This signaling pathway is essential because it again demonstrates the role of Chl*a* as a signal transducer to the center of flexibility. Under optimal (Figure 7a) and over-reduced (Figure 7b) PQ pool conditions, Phe/Tyr124*^fg^*^-loop-suIV^ is controlled by Chl*a*. Under optimal conditions for the electron transport function of cyt*b*_6_*f*, Phe/Tyr124*^fg^*^-loop-suIV^ is shielded from interaction with kinases by lipids. Note that the same lipid shielding of Phe/Tyr124*^fg^*^-loop-suIV^ is observed in the cryo-EM structures at the smallest p-gate width of 6 Å (Table S3, structure 1-7ZXY). At a more oxidized state of the PQ pool (i.e., during the induction of the transition to State 1), the flexibility center Phe/Tyr124*^fg^*^-loop-suIV^ rotates from a membrane-buried to an n-side-oriented position. It is distant from interaction with the kinase transmembrane helix residue Phe116 of STN7 in the plant *Arabidopsis* or Tyr121 of Stt7 of alga *Chlamydomonas*, as predicted in [53]. The lipid shielding of Phe/Tyr124*^fg^*^-loop-suIV^ under oxidized conditions allows for interaction with a kinase to occur immediately after a signal from the Chl*a* volume change, making Phe/Tyr124*^fg^*^-loop-suIV^ available for interaction with the above kinases’ transmembrane helix residues. Moreover, the position of Arg125*^fg^*^-loop-suIV^—the only tested residue for interaction with the kinase stromal fragment [93]—is a consequence of the conformational change in Phe/Tyr124*^fg^*^-loop-suIV^. When Phe/Tyr124*^fg^*^-loop-suIV^ is buried in the membrane and not lipid-shielded, it can interact with the above transmembrane helix residue of the kinases. In contrast, Arg125 *^fg^*^-loop-suIV^ is located on the n-side and interacts with the C-terminus of cyt*b*_6_-(Leu215) and the peripheral PetP and TSP9 (see Section 1). When the Phe124*^fg^*^-loop-suIV^ aromatic ring is turned to the n-side under oxidizing PQ pool conditions, Arg125 *^fg^*^-loop-suIV^ remains up but distant from the C-terminal part of cyt*b*_6_. However, Arg125 *^fg^*^-loop-suIV^ cannot interact with a kinase stromal fragment because the kinase–cyt*b*_6_*f* interactions occur under reducing conditions for the PQ pool [59].

Figure 7a presents the first structural evidence for the previously enigmatic role of the Car molecule in cyt*b*_6_*f*, marking a significant breakthrough. The role of β-Car is to mediate signaling from Chl*a* to the center of flexibility—the Phe/Tyr124*^fg^*^-loop-suIV^ residue—under optimal conditions for electron transport (see also Section 4.4 below).

### 4.4. Raft-like Nanodomains and Different Timescales of Cytb_6_f Lipid Nanoenvironment Dynamics

Two types of raft-like nanodomains were identified in the X-ray crystal structures of cyt*b*_6_*f*—a bilayer nanodomain of 12 molecules under optimal conditions for electron transport under hydrophobic matching conditions (Figure 8) and a monolayer nanodomain of eight molecules under reducing conditions for the PQ pool when there is positive hydrophobic mismatching (Figure 9). The identified lipid nanodomains are considered lipid raft-like, based on their sizes of ~9 nm and 7 nm, respectively [145], and their crystal contacts, indicative of common, collective low-frequency vibrations (phonons) [146]. These are different from the vibrations of the uncontacted lipids.

Notably, the lipids around the transmembrane helices of Rieske ISP and cyt*f* —those from the L4 lipid-binding site group—are not involved in the bilayer raft-like nanodomain (Figure 8). This is entirely in line with the fact that the operation of cyt*b*_6_*f* as a charge transfer catalyzer requires higher mobility, especially of Rieske ISP. Since it is known that the timescale of the Q-cycle is in the range of a few milliseconds [129,130], it is an important result that the dynamics of the lipids involved in the Q-cycle are significantly faster than those of the lipids involved in raft-like nanodomain formation (Figure 8). The dynamics of lipids resolved in the X-ray crystal structures can be divided into three timescale groups: (i) The dynamics of annular lipids not involved in the lipid nanodomains under optimal conditions for electron transport. Their dynamics are slower than those of the bulk lipids but faster than those of the nanodomain-forming lipids, as shown in Figure 8 and Figure 9. The upper limit for the slower dynamics of these lipids could be a few milliseconds—the turnover time for PQH_2_ processing at the Qp-site. (ii) The dynamics of the lipids involved in the monolayer raft-like lipid nanodomains under reducing conditions, as shown in Figure 9. Their dynamics are slower than those of the annular lipids not engaged in the nanodomains but faster than those of lipids forming a bilayer-shell nanodomain (Figure 8). They are faster because the raft-like nanodomain under optimal conditions (Figure 8) is more ordered, has transbilayer contacts, and includes more molecules (12 vs. 8) than the raft-like nanodomain under reducing conditions (Figure 9). Therefore, the upper limit for the slower dynamics of these lipids (Figure 9) could be well above a few milliseconds. Hence, the role of the monolayer raft-like nanodomain is likely to delay the operation of the Q-cycle under reducing conditions. (iii) The dynamics of the bilayer raft-like nanodomains (Figure 8) will be the slowest relative to the above two cases. Moreover, the bilayer raft-like nanodomain (Figure 8) forms under optimal conditions for the primary function of cyt*b*_6_*f*—electron transport—when there is a hydrophobic match between cyt*b*_6_*f* and the hydrophobic thickness of the bulk lipid bilayer (Section 3.2). Therefore, the assigned slowest dynamics of the bilayer raft-like nanodomain align with the fact that the exchange rate values for the lipid–protein interaction are lowest with optimal hydrophobic matching [153]. Thus, the role of the two bilayer raft-like nanodomains surrounding the cyt*b*_6_*f* dimer under optimal conditions (Figure 8) is to provide a lipid bilayer scaffold, ensuring the maximal efficiency of cyt*b*_6_*f* function in electron transport. At the same time, this bilayer shell scaffold serves as a perfect signaling platform for Chl*a* volume-change propagation along the chain–chain contacts at different destinations. This will disrupt the shell and prepare the system to respond through various lipid sorting and redistribution processes.

Notably, these chiral-phonon-assisted raft-like nanodomains are formed thanks to Chl*a* and β-Car. They support two partially shielded annular lipid-binding sites: Chl*a*, the p-L2(1) site, and β-Car, the p-L3(1) site. Thus, Chl*a* and β-Car also play a role as a pair of perfect chiral-phonon-assisted raft-makers.

Interestingly, the third partially shielded lipid-binding site, n-L5(4), is also involved in the formation of a raft-like nanodomain, this time in the monolayer one under reducing conditions (Figure 9). Thus, all the partially shielded annular lipid-binding sites are key players in the formation of two types of thylakoid lipid nanodomains: (i) bilayer-shell lipid nanodomains (Figure 8) and (ii) the n-side monolayer nanodomain of eight lipid/detergent molecules (Figure 9).

It is interesting to note that in the cryo-EM structure of cyt*bc*_1_ (complex III_2_) in a supercomplex with complex I (8BEL, [154]), a similar nanodomain of transmembrane lipid contacts is revealed (see Figure S2). However, unlike the cyt*b*_6_*f* nanodomain formed by Chl*a* and β-Car, the transmembrane lipid contacts do not obey the chirality-selective rule. Thus, the Chl*a*-formed nanodomain is for directed signaling, while that in cyt*bc*_1_ is for supercomplex formation.

The chiral-phonon-assisted raft-like nanodomain formation (Figure 8) is ascribed to the optimal, stationary state of cyt*b*_6_*f*. For this state, the native lipid shell of cyt*b*_6_*f* is expected to be dominated by PG (Table 1). This raises the question of whether such symmetric lipid distribution may form an ordered domain, known as a raft. The answer is yes because a loss of asymmetry can induce domain formation in vivo [155]. The domination of negative PG and one negative SQDG indicates a loss of lipid asymmetry in an optimal stationary state, which is reasonable, given the formation of an ordered nanodomain bilayer shell.

### 4.5. Strong Lipid Selectivity of the Different Conformational States of Cytochrome b_6_f

The primary message of this work is that cyt*b*_6_*f* binds various numbers and classes of lipid molecules, as determined by X-ray crystallography and cryo-EM imaging methods, in its three key conformational states: optimal, over-reduced, and over-oxidized states (Table 1). These three conformational states reflect the redox state of the PQ pool under optimal conditions for electron transport when there is hydrophobic matching (d_P_ = d_L_), during the induction of the transition to State 2 when the positive hydrophobic mismatch (d_P_ > d_L_) reaches its maximum, and during the induction of the transition to State 1 when the negative hydrophobic mismatch (d_P_ < d_L_) reaches its maximum, respectively. The relative contributions of the four lipid classes in the three main conformational states differ from their relative contribution to the total lipid composition of the thylakoid membrane (see Section 3.5). The different local lipid compositions around cyt*b*_6_*f* in the three states and their deviation from the bulk lipid composition are indicative of hydrophobic mismatch-induced lipid sorting [156,157]. Therefore, this ability of cyt*b*_6_*f* to attract different classes of lipids depending on its hydrophobic thickness is not only evidence that the hydrophobic mismatch-induced lipid sorting concept [156] is active but also validates the proposed hydrophobic mismatch model for cyt*b*_6_*f*-driven state transitions [53].

From a photosynthetic viewpoint, these results show that cyt*b*_6_*f* is the first integral membrane protein of the thylakoid membrane to possess such strong lipid selectivity in its conformational states. Cyt*b*_6_*f* appears to be unique in dynamically tuning its lipid nanoenvironment, thanks to its ability to induce both positive and negative hydrophobic mismatches. In contrast, as reviewed in [4], PSI and PSII do not show different conformational states. PSII and PSI possess an asymmetric lipid distribution—anionic lipids, such as SQDG and PG, are distributed at the stromal (n-side) leaflet. In contrast, non-charged lipids, including MGDG and DGDG, are located at the luminal leaflet of these complexes [4]. For cyt*b*_6_*f*, there is no such asymmetric lipid distribution (Table 1). The anionic lipids, as well as the neutral lipids, can occupy both membrane leaflets of cyt*b*_6_*f*.

These selective bindings of the lipid classes are particularly interesting because they also adhere to the hydrophobic matching principle. Lipids, such as MGDG (18:0/18:0) with a conical shape, negative intrinsic curvature, and saturated chains, compensate for the positive hydrophobic mismatch (d_P_ > d_L_). Lipids, such as OPC (18:1/18:1), i.e., DGDG [108], with a cylindrical shape, zero curvature, and unsaturated chains, compensate for the negative hydrophobic mismatch (d_P_ < d_L_) [158]. Therefore, these results contribute to understanding the induction mechanism of state transitions by validating the proposed hydrophobic mismatch model for cyt*b*_6_*f*-driven state transitions [53]. Moreover, these results are also important for understanding the spatiotemporal sequence of events leading to the onset and progression of state transitions.

The present work does not deal with any influence on the thickness of the bulk lipid bilayer phase because there is no report of changes in the bilayer or hydrophobic thickness of the bulk lipid phase during state transitions. This is in contrast to the regulatory mechanism of high-energy non-photochemical quenching, which is accompanied by a decrease in membrane thickness [159,160,161]. Moreover, the thylakoid membrane lacks cholesterol, which can change the bilayer thickness in other membranes [30,162,163]. Finally, according to the proposed HMM in [53] and validated here, the lipid response to the increasing or decreasing hydrophobic thickness of cyt*b*_6_*f* is lipid sorting and redistribution in order to compensate locally for the cyt*b*_6_*f*-induced hydrophobic mismatch. The hydrophobic thickness of the bulk lipid bilayer remains unchanged. This is illustrated in Section 4.7 below.

It is known that identifying lipids in crystal and cryo-EM structures is challenging due to their structural flexibility and potential non-specificity of site occupancy (e.g., [108]). However, since there is logic in the changes in lipid occupancy across the different cyt*b*_6_*f* structures, in accordance with the hydrophobic matching concept (Table 1), these occupant changes cannot be attributed to the non-specificity of lipid site occupancy or the uncertainty in lipid class modeling.

The fact that the two searched lipid types [53] are actually two distinct lipid classes (Table 1) is an interesting finding. Since the two identified lipid classes differ by their molecular geometry, phase behavior, and intrinsic curvature, it is evident that the two major physical stimuli, the hydrophobic mismatch and the local membrane curvature under hydrophobic mismatching conditions, are the origin of the driving forces for lipid sorting and restructuring around cyt*b*_6_*f* in the thylakoid membrane during the induction phases of the state transitions.

Together, these observations provide strong dynamic–structural evidence that the signal generated by cyt*b*_6_*f* during the induction of state transitions is primarily transmitted to other destinations via the lipid components of the thylakoid membrane. Therefore, the lipids, through their redistribution during the induction of state transitions, serve as the primary effectors of signals from cyt*b*_6_*f* in response to light-quality-induced changes in the redox state of the PQ pool.

### 4.6. State Transitions Are the Regulatory Mechanism That Relies on the Evolutionarily Conserved Thylakoid Lipid Composition

Thylakoid lipid composition has remained highly conserved throughout evolution [3,4,7]. The clarified role of PG substitution by MGDG (during the induction of the transition to State 2) and SQDG substitution by DGDG (during the induction phase of the transition to State 1) exemplifies the full utilization of the evolutionarily conserved thylakoid lipid composition. Therefore, cyt*b*_6_*f* appears to be the first recognized and most active user of the thylakoid membrane’s conserved four-lipid-class composition.

The present finding is that MGDG and DGDG binding to cyt*b*_6_*f* plays a role in the conformational dynamics of cyt*b*_6_*f*, which is connected to the function of cyt*b*_6_*f* in state transitions. In contrast, PG and SQDG bindings are associated with the primary function of cyt*b*_6_*f* in electron transport (Table 1), which explains the experimental results from a study investigating the effect of individual lipids on the structural and functional integrity of cyt*b*_6_*f* from spinach [164]. The authors found that adding the native (DLPG and SQDG) and synthetic (DOPG and DOPC) lipids stabilized the cyt*b*_6_*f* structure. In contrast, adding the native galactolipids MGDG and DGDG did not cause any significant stabilization. The ability of cyt*b*_6_*f* to undergo conformational changes has never been studied by these authors.

The ability of cyt*b*_6_*f* to attract different classes of lipids depending on its hydrophobic thickness is evidence that the lipid sorting concept [156] is active (see Section 4.5 above). According to this concept [156], hydrophobic mismatch-induced lipid sorting around proteins is observed in a homogeneous lipid mixture (just like the lipid phase of the thylakoid membranes under optimal conditions for photosynthesis; see the Introduction) as an alternative to phase preference when the lipids undergo lateral phase separation (as in other biomembranes able to form lipid rafts). Nature probably selected the unique and conserved four-lipid-class composition for the thylakoid membranes to keep their homogeneous distribution, just to operate the hydrophobic mismatch-induced lipid sorting and not the hydrophobic mismatch-induced phase preference (including lipid rafts) as in other biological membranes. The cyt*b*_6_*f*-induced lipid bilayer raft-like formation with hydrophobic matching, under optimal conditions for its electron transport function, first reported here (Figure 8), is obviously needed to maximize the photosynthetic electron transport efficiency and for perfect signaling for a change in the PQ pool redox state. The cyt*b*_6_*f*-induced monolayer lipid raft-like formation under PQ pool reducing conditions is most probably needed for dimer and Rieske ISP stabilization at a reduced protein state (Figure 9).

Cyt*b*_6_*f* has some similarity to rhodopsin (GPCR) and mechanosensitive ion channels, such as MscL, but is not identical. Similarities with rhodopsin exist in the same role of non-bilayer lipids (e.g., [165]), the local curvature [166], similar volume changes [167], and a similar ability to change their hydrophobic thickness during the operation of the Q-cycle, i.e., in the ms time range, as during the activation of rhodopsin [143]. The similarity with the mechanosensitive ion channel MscL [168] is in the combined effect of hydrophobic mismatch and bilayer local bending, underlying the important role of both physical stimuli—hydrophobic mismatch and the local bilayer curvature (see Section 4.5 above). However, in the time range of seconds, only cyt*b*_6_*f* can induce hydrophobic mismatch, obey the protein-induced lipid sorting concept [156], change its volume, and induce lipid redistribution, which triggers the reorganization of other proteins. This ability of cyt*b*_6_*f* may stimulate research in the field of rhodopsin and mechanosensitive channels regarding the role of hydrophobic mismatching in the time range of a few seconds.

### 4.7. The Hydrophobic Mismatch Model for Cytochrome b_6_f-Driven State Transitions—Bulk and Bound Lipid Dynamics

**Figure 10:**
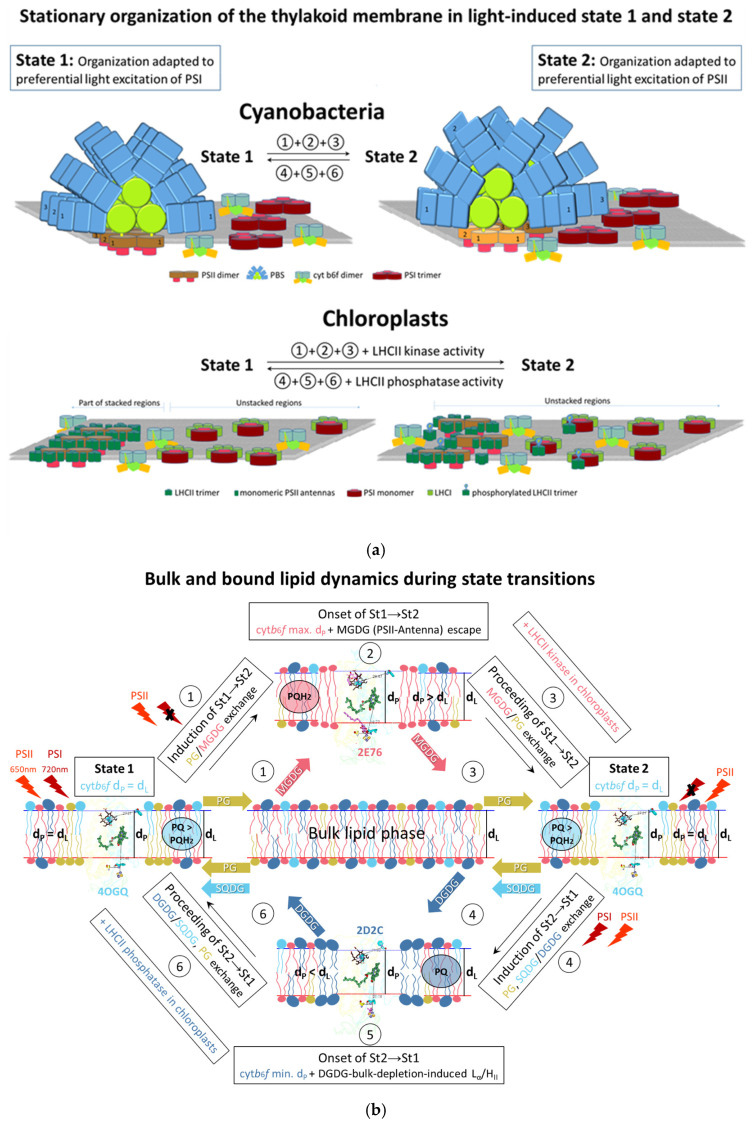
Schematic representation of state transitions in cyanobacteria and chloroplasts. (**a**) Stationary State 1 and State 2 organization of the antenna–photosystem complexes in the cyanobacterial and chloroplast thylakoid membranes’ third (mixed) domains. With low-intensity light, the PSII-PBS megacomplexes in cyanobacteria and PSII-LHCII in chloroplasts are more ordered, with fewer contacts (if any) between the PSII antenna and PSI and between the PSII antenna and PSI in State 1 than in State 2. The relative dimensions of the presented complexes mimic those of the native ones; (**b**) dynamic lipid exchange between cyt*b*_6_*f*-bound and bulk bilayer lipid classes identified in the present work and predicted to occur during state transitions in vivo. Presented in the center is the bulk lipid phase—the source of lipids for sorting around and exchange at cyt*b*_6_*f* and where the escaped lipids from cyt*b*_6_*f* go. The four-lipid-class compositions of the bulk lipid phase are indicated by four colors with lipid class names given on the respective colored arrows. Only part of the cyt*b*_6_*f* monomer structure of 4OGQ, 2E76, and 2D2C is presented with the key residues and Chl*a*. The following processes are indicated: ①—the induction phase of the transition to State 2; ② the onset of the transition to State 2; ③—the proceeding of the transition to State 2 to its completion; ④—the induction phase of the transition to State 1; ⑤—the onset of the transition to State 1; and ⑥—the proceeding of the transition to State 1 to its completion. Lipid exchange at and escape from cyt*b*_6_*f* is indicated by colored arrows with the names of the respective lipids. See the text for details.

Figure 10 shows a schematic representation of (a) the stationary State 1 and State 2 organization of the antenna–photosystems in cyanobacteria and chloroplasts. Panel (b) schematically presents the proposed hydrophobic mismatch molecular mechanism for the transitions between State 1 and State 2. Note that the differentiation of bulk and bound lipids is a new element added to the model described in [53].

Starting from the stationary State 1 (on the left of Figure 10b), cyt*b*_6_*f* is in hydrophobic matching conditions with the bulk lipid phase (d_P_ = d_L_, structure 4OGQ). This stationary State 1, the stationary State 2, and the optimal state for electron transport with low-intensity light are characterized by the domination of PG bound to cyt*b*_6_*f* (see Table 1). Upon turning off the PSI light, ①—the induction phase of the transition to State 2—begins. It begins because the change in light quality induces a change in the PQ pool redox state from an oxidized (PQ > PQH_2_) to an over-reduced (PQH_2_) state. The hydrophobic thickness of cyt*b*_6_*f* starts to increase (d_P_ > d_L_). This is paralleled by the exchange of the cyt*b*_6_*f*-bound PGs at p-L2(1) and p-L3(1) and a detergent at n-L5(4) with MGDGs from the bulk lipid phase, compensating temporally for the increasing hydrophobic thickness of cyt*b*_6_*f* (Table 1). This leads to MGDG depletion in the bulk lipid phase and its enrichment with PGs from the cyt*b*_6_*f*. ②—the onset of the transition to State 2—corresponds to the over-reduced state of the PQ pool, maximal positive hydrophobic mismatch, and the domination of MGDG bound to cyt*b*_6_*f* (d_P_ > d_L,_ structure 2E76, Table 1). This is the maximal fluorescence level reached during the continuous recording of state transition traces using PAM fluorometry (e.g., Figure 6A in [53]). The onset ② occurs when the MGDG depletion in the bulk lipid phase reaches a critical level, and the MGDGs’ escape from the lipid shell of antenna–PSII supercomplexes starts [169]. The destabilization of non-bilayer lipid binding as of MGDG will influence peripheral (PBS) and integral (LHCII) membrane proteins through changes in the lateral pressure profile [158]. Thus, the detachment of MGDGs from the ordered antenna–PSII megacomplexes (Figure 10a, left) will destabilize these megacomplexes, decreasing the energetic connectivity between the antenna complexes of PSII-RC that was stabilized by the lateral pressure exerted by the surrounding MGDG lipids [170,171]. This leads to the start of a decrease in the reduction level of the PQ pool due to the reduced excitation pressure on the PSII reaction centers. Given that state transitions rely on the evolutionarily conserved lipid composition (Section 4.6), together with the evolutionarily conserved core subunits of PSII and PSI [16], and considering that state transitions are also evolutionarily conserved, lipid destabilization likely occurs at the interface of the core PSII subunits. As seen from the recent structure of red algae complexes [72], two MGDGs and two DGDGs mediate hydrophobic interactions between CP43 and CP47 of adjacent PSII dimers. In chloroplasts, identical PSII dimers are also in contact with CP43 and CP47 of the two neighboring dimers [172]. In all cases, the excitation energy transfer destabilized at this CP43-CP47 point of contact will also destabilize the antenna–antenna contacts (e.g., those seen in [173]). Destabilizing the contacts between the neighboring PSII dimers will immediately destabilize the more ordered state of antenna–PSII megacomplexes. This will decrease the efficiency of excitation light energy transfer and the photochemical efficiency of PSII and, hence, will be the first stimulus to decrease the reduction level of the PQ pool. This will be seen as an immediate decrease in PAM-recorded fluorescence changes (e.g., Figure 6 in [53]). Then ③—the proceeding of the transition from State 1 to State 2—occurs. It is PAM-registered as a continuous decrease in the fluorescence signal on a timescale of minutes until it reaches a stationary level. Since the hydrophobic thickness of cyt*b*_6_*f* starts to decrease, MGDGs bound to cyt*b*_6_*f* begin to return to the bulk phase. PGs from the bulk start to return to their previous binding sites on cyt*b*_6_*f* because the inherent lipid property to return to their places of origin after the stimulus for their restructuring is relaxed [174]. In cyanobacteria, ③ proceeds without the mediating involvement of a kinase [69,70]. MGDG-bound PBSs can distance from more disordered PSII rows and contact PSI or both PSI and PSII. In chloroplasts, cyt*b*_6_*f* content is five times smaller than in cyanobacteria relative to Chl*a* (see the estimate made in [136] and references therein), and hence, to the transmembrane protein content, the LHCII kinase—STN7 in plants [64] and Stt7 in algae [65]—is activated, as proposed in [53] (or see the Introduction). During ③, at least two kinetics of fluorescence signal decrease are expected—one very fast, due to the destabilization of antenna–PSII ordered rows, and the second, much slower, due to the reorganization of protein complexes. The protein compartment reorganizes in a way that enhances antenna–PSI interactions. This is stabilized by the PSI enrichment with the MGDG-bound antenna coming from PSII. That the lipid–antenna complexes can move together has also been suggested by [175]. After the transition to State 2, there is again an enrichment of cyt*b*_6_*f* with the bound PGs, and the hydrophobic matching conditions in State 2 (d_P_ = d_L_, structure 4OGQ on the right of Figure 10b) are reached. It is clear from the model that the lower fluorescence quantum yield of PSII in State 2 is due to the decreased MGDG content around PSII–antenna megacomplexes. This destabilizes the highly efficient energy transfer at PSII-PBS or PSII-LHCII megacomplexes due to the decreased lateral pressure exerted by the lower MGDG content surrounding antenna–PSII megacomplexes. Therefore, the present model precludes a search for a quenching center in PSII, according to a so-called PSII quenching model in [71].

Upon turning on the PSI light (on the right side of Figure 10b), ④—the induction phase of the transition to State 1—starts. It starts because the change in light quality induces a change in the PQ pool’s redox state from an oxidized (PQ > PQH_2_) to an over-oxidized (PQ) state. The cyt*b*_6_*f* hydrophobic thickness starts to decrease (d_P_ < d_L_), accompanied by a massive escape of PGs and SQDG from cyt*b*_6_*f* (Table 1). DGDGs from the bulk phase bind to cyt*b*_6_*f* at n-L4(1−3) and n/p-L5(2) lipid-binding sites to temporally compensate for the decreasing hydrophobic thickness of cyt*b*_6_*f*. The bulk phase becomes enriched in PGs and SQDG but is depleted of DGDG. ⑤—the onset of the transition to State 1— corresponds to the over-oxidized state of the PQ pool, maximal negative hydrophobic mismatch, and the domination of DGDG bound to cyt*b*_6_*f* (d_P_ < d_L,_ structure 2D2C-CLA1201). This is the minimal fluorescence level reached during continuous recording of state transition traces using PAM fluorometry (e.g., Figure 6A in [53]). The onset ⑤ occurs when the DGDG depletion in the bulk lipid phase reaches a critical level, at which the decrease in the DGDG content in the bulk lipid phase destabilizes the bilayer phase. The coexistence of the liquid crystalline (Lα) and inverted hexagonal (H_II_) phases may occur [7], resulting in decreased PSI stability. DGDG deficiency, as observed in a DGDG-deficient mutant, primarily affects PSI stability [176]. PSI’s destabilization will undoubtedly destabilize its contacts with the PSII–antenna complexes. This destabilization will immediately decrease the rate of PSI electron withdrawal from the PQ pool. It will start to increase the PQ pool reduction level, and the fluorescence signal will increase. Then ⑥, the proceeding of the State 2 to State 1 transition, occurs, registered as a continuous increase in the fluorescence signal on a timescale of minutes until it reaches a stationary level, similar to that of stationary State 2 [53]. Since the hydrophobic thickness of cyt*b*_6_*f* starts to increase, DGDGs bound to cyt*b*_6_*f* begin to return to the bulk lipid phase. In cyanobacteria, ⑥ proceeds without the mediating involvement of a phosphatase. PGs and SQDG from the bulk start to return to their previous binding sites on cyt*b*_6_*f* because the inherent lipid property returns to its original position after the stimulus for its restructuring is relaxed [174]. MGDG-bound PBSs and MGDG-bound P-LHCIIs will be detached from PSI. In cyanobacteria, the MGDG-bound PBS returns to its prior location at PSII without the involvement of a phosphatase. In chloroplasts, cyt*b*_6_*f* content is five times smaller than in cyanobacteria relative to Chl*a* (see the estimate made in [138] and references therein), and hence, to the transmembrane protein content, the constitutively active LHCII phosphatase in plants [68] and in algae [67], will dephosphorylate them, as proposed in [53]. During ⑥, at least two kinetics of fluorescence signal increase are expected—one very fast, due to the destabilization of antenna–PSII binding to PSI due to the effect of the DGDG-depletion-destabilized PSI, and the second, much slower, due to the protein complexes’ mutual reorganization until the State 1 organization is restored (Figure 10a left). The protein compartment reorganizes in a way that enhances antenna–PSII interactions. This is stabilized by the enrichment of PSII with MGDG-bound antenna proteins (PBSs and LHCII), which are detached from PSI. After the transition to State 1, there is again enrichment of cyt*b*_6_*f* with bound PGs and one SQDG, and the hydrophobic matching conditions are reached in State 1 (d_P_ = d_L_, structure 4OGQ). At the same time, MGDG-PBSs will return to PSII in cyanobacteria. In chloroplasts, the destabilized PSI-LHCI contacts with P-LHCII will facilitate the dephosphorylation of P-LHCII and, together with MGDG, they will return to their prior position, becoming loosely bound to PSII. The return of DGDG to the bulk phase will stabilize the bilayer phase and restore the larger number of grana stacks and their dimensions.

### 4.8. Testable Predictions of the Hydrophobic Mismatch Model for Cytochrome b6f-Driven State Transitions

Structurally confirmed here, the hydrophobic mismatch model for cyt*b*_6_*f*-driven state transitions widely opens the door to experimental and computational confirmation. A perfect test could come from in vivo studies on the state transitions of lipid mutants.

It can be predicted that if an MGDG-deficient mutant is studied, it should be expected to possess a lower (or no) ability to transition to State 2 because MGDG is required during the induction phase of the transition to State 2 (Table 1). There is no study on the ability of MGDG-deficient mutants to perform state transitions. In this case, the low-temperature 77K spectra of the MGDG *Arabidopsis* mutant, relative to the wild type, are encouraging, showing lower energy transfer to PSI in the mutant [177]. That is, the MGDG-deficient mutant is closer to the State 1 organization.

It is expected that DGDG-deficient mutants in vivo will possess a lower ability to perform the transition to State 1, as the induction phase of this transition requires bulk DGDG availability for sorting around cyt*b*_6_*f* (Table 1). Indeed, an in vivo study of a DGDG-deficient mutant of *Arabidopsis* has shown a ~50% reduction in the capacity for state transitions relative to the wild type [178]. The authors associated this lower capacity with a higher reduction state of the PQ pool in the mutant. Although the authors did not specify which transition is suppressed, Figure 5 in their paper indicates that this is indeed the transition to State 1.

There is no study on the ability of SQDG-deficient mutants to perform state transitions. It is expected that SQDG deficiency will lead to a state closer to State 1, as the induction phase of the transition to State 1 requires the release of SQDG for cyt*b*_6_*f* to undergo a more considerable conformational change, reaching the minimal p-gate, d_n_, and d_P_. Since SQDG is required for efficient electron transport under optimal conditions (Table 1), it is also expected that SQDG deficiency will affect the efficiency of electron transport under optimal conditions.

PG deficiency will significantly affect the characteristics of the optimal, stationary state because PG dominates the bound lipid fraction on cyt*b*_6_*f* under optimal conditions for photosynthesis under low light illumination. A PG-deficient mutant is expected to be locked in State 1 because the complete escape of PG from cyt*b*_6_*f* is observed upon the induction of the transition to State 1 (Table 1). There has been no work on the effect of PG deficiency on state transition ability. However, recent work [179] has shown that in an *Arabidopsis* PG mutant, there is a slightly higher PSII/PSI 77K fluorescence ratio. Since a higher ratio usually means diminished energy transfer to PSI, the plant PG mutant antenna–photosystem organization is closer to State 1.

A careful analysis of the fluorescence decrease during ③ and the increase during ⑥ (Section 4.7) in the PAM-recorded fluorescence traces of state transitions in vivo is expected to confirm the suggested at least two-component kinetics here (Section 4.7).

The HMM model predicts that during the proceeding transition to State 2, MGDG-bound antenna complexes (PBS in cyanobacteria and LHCII in chloroplasts) will lead to the enrichment of MGDG around PSI. MGDG enrichment will stabilize the trimer PSI in cyanobacteria and increase the energy transfer to PSI [180,181,182]. In chloroplasts, the attachment of MGDG-bound phosphorylated LHCII to PSI will increase the content of MGDG in its solvation shell. The PSI lipid shell has not yet been studied by molecular dynamics simulations, in contrast to PSII [169] and LHCII [171]. Molecular dynamics simulations on State 2 (PSI-LHCI-LHCII) and State 1 (PSI-LHCI) supercomplexes of PSI can determine whether there is an enrichment of MGDG in the lipid shell surrounding the State 2 relative to State 1 PSI supercomplexes.

## 5. Conclusions

Previous work [53] elucidated how the signal from the PQ pool is sensed by cyt*b*_6_*f* and transduced from its Qp-site on the luminal side to its n-side (stromal/cytoplasmic). The present work answers the question of how the signal from cyt*b*_6_*f* is transmitted to the antenna–photosystem supercomplexes and megacomplexes to induce their movement and reorganization. This paper reveals the unique role of lipids, governed by the active protein cyt*b*_6_*f*, in inducing a balance between the two photosystems in the thylakoid membrane, which occurs on a timescale of a few seconds. Our understanding of hydrophobic mismatch in protein reorganization has been expanded to include its role in dynamic protein signaling that occurs over seconds. The present paper definitively supports the proposed hydrophobic mismatch model for cyt*b*_6_*f*-driven state transitions [53] by providing (1) evidence of cyt*b*_6_*f*-driven lipid exchange that adheres to the hydrophobic matching principle and (2) the identification of two distinct lipid types, previously proposed [53] to interact with cyt*b*_6_*f* during the transition to State 2 and State 1. During the transition to State 2, the sorting of the non-bilayer-forming neutral lipid MGDG (which has a negative intrinsic curvature) and the exchange of anionic PGs with MGDG at specific lipid-binding sites represent the lipid response to the increasing positive hydrophobic mismatch induced by cyt*b*_6_*f*. Conversely, for the transition to State 1, the sorting of the bilayer-forming lipid DGDG and the exchange of anionic SQDG with DGDG, along with the release of various binding sites (except for the n-/p-L5(2) core), reflect the lipid response to the increasing negative hydrophobic mismatch caused by cyt*b*_6_*f*. Cyt*b*_6_*f* appears to be unique in dynamically tuning its lipid nanoenvironment, thanks to its ability to induce both positive and negative hydrophobic mismatches. Together, these observations provide strong dynamic–structural evidence that the signal generated by cyt*b*_6_*f* to the antenna–photosystem complexes during state transitions is primarily transmitted via the lipid components of the thylakoid membrane. The lipids, through their redistribution during the induction of state transitions, serve as the primary effectors of signals from cyt*b*_6_*f* in response to light-quality-induced changes in the redox state of the PQ pool. The clarified roles of MGDG substituting for PG and DGDG substituting for SQDG highlight the effective utilization of the evolutionarily conserved lipid composition in the thylakoid. Cyt*b*_6_*f* appears to be the first and most active user of these four conserved lipid classes in the thylakoid membrane. The hydrophobic mismatch model is further extended by detailing the distinct roles of bound lipids and the bulk lipid phase, alongside a thorough description of lipid-binding dynamics throughout the induction, onset, and completion of transitions to State 2 and State 1, each with its anticipated effects on the antenna–PSII and antenna–PSI megacomplexes. Additionally, for the first time, structural evidence is provided for the carotenoid molecule acting as a mediator in Chl*a*’s unidirectional signaling to both the center of flexibility, Phe/Tyr124*^fg^*^-loop-suIV^, and to other lipids that form a bilayer raft-like nanodomain around each cyt*b*_6_*f* monomer. Hence, Chl*a* and β-Car also play a role as a pair of perfect chiral-phonon-assisted raft-makers. This bilayer shell serves as a scaffold for maximizing electron transport efficiency in low-light conditions and provides a perfect signaling platform for Chl*a* volume-change propagation along the chain–chain contacts at different destinations under light-quality-induced changes in the redox state of the PQ pool. New evidence is also presented for the role of Chl*a* as a crucial redox sensor and transmembrane signal transmitter. This raised the idea of how Chl*a*, via its volume changes, senses the redox state of the PQ pool and opens the door for experimental exploration of Chl*a*-[2Fe-2S] magnetic interactions. Furthermore, this work clarifies the underlying principles that guide the dynamics of lipid binding to cyt*b*_6_*f* during the induction and progression of state transitions in vivo. Based on this, one can confidently predict the effect of each lipid class deficiency on state transition experiments in vivo using lipid mutants.

In summary, this study provides essential new knowledge regarding the role of lipids in state transitions. The data compellingly demonstrate that lipids are primary effectors of signals from cyt*b*_6_*f* to the antenna–photosystem super- and megacomplexes during these transitions. These findings will be of interest to membrane biophysicists, structural biologists, and those focused on the rational design and development of next-generation energy-collecting materials under various environmental conditions. The results could inspire the design of hybrid and artificial membrane materials with beneficial properties.

## Figures and Tables

**Table 1:** Dynamics of predicted native lipid binding on the cytochrome *b*_6_*f* complex upon induction of state transitions.

Specific Lipid-Binding Sites	Optimal (Oxidized) Stationary Conditions (p-Gate = 6.5 Å; d_P_ = 30.6 Å)	From Optimal to Over-Reduced (Induction Phase for State 2) (p-Gate > 7.2 Å, d_P_ > 31.2Å)	From Optimal to Over-Oxidized (Induction Phase for State 1) (p-Gate < 6.5 Å; d_P_ < 30.6 Å)
n-L1(1)	PG ^c^	PG ^c,d^	Escape of PG to bulk
n-L2(1)	PG ^c^	Escape of PG to bulk	Escape of PG to bulk
p-L2(1) ^a^	PG ^c^	Exchange of PG with MGDG	Escape of PG to bulk
p-L3(1) ^a^	PG ^d^	Exchange of PG with MGDG	Escape of PG to bulk
n-L4(1)	SQDG	SQDG	Exchange of SQDG with DGDG ^e^
n-L4(3)	n.i. (detergent)	MGDG ^c^	Empty
p-L4(1)	PG ^c^	PG	Escape of PG to bulk
n/p-L5(2) ^b^	DAG/PQ	PQ	Exchange of DAG/PQ with DGDG ^e^

^a^ Partially shielded annular lipid-binding sites. ^b^ Core–annular lipid-binding site. ^c^ The native lipid is deduced from the cryo-EM structures at the same p-gate width (see Table S3 and Text S3). ^d^ The ability of PG in X-ray structure 4I7Z to substitute PC. The absence of an upper index indicates that the native lipid occupant or its escape is taken from the X-ray structures. ^e^ The synthetic PC lipid may be proposed as a DGDG lipid [108]. Exchange means that one lipid from cyt*b*_6_*f* goes to the bulk lipid phase, and another lipid class from the bulk phase binds to cyt*b*_6_*f*. Escape means the lipid detaches from cyt*b*_6_*f* and goes to the bulk lipid phase.

## Data Availability

Data are contained within this article and the Supplementary Materials.

## Supplementary Materials

The following eight files, which provide supporting information can be downloaded at https://www.mdpi.com/article/10.3390/membranes15050143/s1: Supp_Data.pdf (Tables S1–S5, Texts S1–S4 and Figures S1 and S2); Suppl_Images-1.pdf (Supplementary Package of Images—Part 1 for scrolling 3D visualizations related to Figure 2 and Figure 3); Suppl_Images-2.pdf (Supplementary Package of Images—Part 2 for scrolling 3D visualizations related to Figure 6); SupplVideo1.mp4 (3D visualization of Figure 2b); SupplVideo2.mp4 (3D visualization of Figure 3); SupplVideo3.mp4 (3D visualization of Figure 6); SupplVideo4.mp4 (3D visualization of the size of the raft-like lipid nanodomain in Figure 8); SupplVideo5.mp4 (3D visualization of the size of the raft-like lipid nanodomain in Figure 9).

## Abbreviations

Cryo-EM	Cryo-electron microscopy
DGDG	Digalactosyldiacylglycerol
HMM	Hydrophobic mismatch
ISP	Iron–Sulfur protein
LHC	Light-harvesting complex
MGDG	Monogalactosyldiacylglycerol
PAM	Pulse-Amplitude-Modulated
PBS	Phycobilisome
PG	Phosphatidylglycerol
PQ	Plastoquinone
PQH2	Plastoquinol
PSI	Photosystem I
PSII	Photosystem II
SQDG	Sulfoquinovosyldiacylglycerol
