# Supplementary material for: X-Ray Crystal and Cryo-Electron Microscopy Structure Analysis Unravels How the Unique Thylakoid Lipid Composition Is Utilized by Cytochrome b6f for Driving Reversible Proteins’ Reorganization During State Transitions
Source: Membranes (Basel). 2025 May 8;15(5):143. doi: 10.3390/membranes15050143 (PMC12112970; doi:10.3390/membranes15050143)
Supplement: Supplementary file 1 [file membranes-15-00143-s001.zip › 1-membranes-3572575-supplementary-5.8.pdf]

Supplementary data

X-ray crystal and cryo-electron microscopy structure analysis unravels how the unique thylakoid lipid composition is utilized by cytochrome b_6f for driving reversible proteins' reorganization during state transitions

Radka Vladkova (rvladkova@bio21.bas.bg)

Captions

Table S1 with Text S1. Table S1, along with Text S1, provides details and an overview of the thirteen crystal and eight cryo-EM dimeric cytochrome b_6f structures analyzed.

Table S2 with Text S2. Table S2, along with Text S2, provides a detailed description of the dynamics of lipid-binding sites as resolved in the crystal structures.

Table S3 with Text S3. Table S3, along with Text S3, provides a detailed description of the dynamics of lipid-binding sites resolved in the cryo-EM structures.

Text S4, Table S4, Table S5, and Figure S1. Comparison of cytb6f and cytbc1 X-ray crystal and cryo-EM structures (related to the Subsection 3.1.2 in the main text)

Figure S1. Lipid nanodomain at the interface between cytbc_1 (complex III) and complex I (PDB ID: 8BEL).

Table S1. X-ray crystal and cryo-EM structures of dimeric cytb_6f from different species.

**No.**	**PDB ID**	**Res Å, Rem**	**Year**	**Ref.**	**Species**	**Q_p-site inhibitor or PQ**	**Q_n-site inhibitor or PQ**	**MGDG[l]**	**SQDG[m]**	**PG[n]**	**PC[o]**	PA, DAG	UDM (UMQ)	**Hydro-carbons**	**Total per mono-mer**
1	1Q90	3.10	2003	[1]	C. *reinh.*[a]	TDS[f] head inside	(PQ?)	2 LMG	1 SQD					1 LFA	4

2	1VF5	3.00	2003	[2]	*M. lam.*[b]	TDS[f] head outside	PQ near c_n				2 OPC				2
3	2D2C	3.80	2006	[3]	*M. lam.*[b]	DBMIB[g] outside					2 OPC				2
4	2E74	3.00	2007	[4]	*M. lam.*[b]				1 SQD		2 OPC		4		7
5	2E75	3.55	2007	[4]	*M. lam.*[b]		NQNO[i]		1 SQD		2 OPC		4		7
6	2E76	3.41	2007	[4]	*M. lam.*[b]	TDS[f] head inside	TDS head inside		1 SQD		2 OPC		4		7
7	2ZT9	3.00	2009	[5]	*Nos.* 7120[c]				1 SQD		2 OPC		3		6
8	4H0L	3.25	2013	[6]	*M. lam.*[b]		NQNO[i]		1 SQD		2 OPC		4		7
9	4H13	3.07	2013	[6]	*M. lam.*[b]	TDS[f] head inside	TDS head inside		1 SQD		2 OPC	1 7PH	2	1 MYS<br>1 8K6<br>1 OCT	9
10	4H44	2.70	2013	[6]	*Nos.* 7120[c]				1 SQD		2 OPC	1 7PH	5	1 MYS<br>1 8K6<br>1 OCT	12
11	4I7Z	2.80<br>+*DOPG*	2013	[7]	*M. lam.*[b]				1 1E2	3 OZ2			3	1 MYS<br>1 8K6<br>1 OCT	10
12	4OGQ	2.50	2014	[8]	*Nos.* 7120[c]			1 1O2	1 SQD		1 OPC	5 7PH<br>1 2WA<br>1 2WD<br>1 2WM<br>1 3WM	4	4 8K6<br>1 MYS<br>1 OCT	22

13	4PV1	3.00	2014	[9]	*M. lam.*[b]		SMA[j]		1 SQD		3 OPC	1 7PH	3	1 8K6<br>1 MYS	10
14	6RQF	3.60 EM	2019	[10]	*S. oler.*[d]	0.5 PQ[h]	PQ[h]	1 LMG	1.5 SQD	2 PGV	1.5 6PL				6
15	7ZXY	3.15 EM, *–PetP*	2022	[11]	*Syn. 6803*[e]					3.5 PGV				1 LFA	4.5
16	7R0W	2.80 EM, *+PetP*	2022	[11]	*Syn. 6803*[e]		PQ[h]	0.5 LMG	1 SQD	2 PGV	1 6PL	0.5 2WA		0.5 LFA	5.5
17	7QRM	2.70 EM, *–TSP9*	2023	[12]	*S. oler.*[d]	3 PQ[h]			1 SQD	2 PGT			5		8
18	7ZYV	2.13 EM, *+TSP9*	2023	[12]	*S. oler.*[d]	3 PQ[h]			1 SQD				5		6
19	9ES7	1.94 EM, *+TSP9*	2024	[13]	*S. oler.*[d]			1LMG	1 SQD				2		4
20	9ES8	2.24 EM, *+TSP9*	2024	[13]	*S. oler.*[d]		DPQ[i]	1LMG	1 SQD				2		4
21	9ES9	2.30 EM, *+TSP9*	2024	[13]	*S. oler.*[d]	DBMIB[g]		1LMG	1 SQD				2		4

[a] Green alga C*hlamydomonas reinhardtii;* [b] cyanobacterium *Mastigocladus laminosus;* [c] cyanobacterium *Nostoc* sp. PCC 7120; [d] plant *Spinacia oleracea;* [e] cyanobacterium *Synechocystis sp. PCC 6803;* [f] inhibitor TDS—tridecyl-stigmatellin; [g] inhibitor DBMIB—2,5-dibromo-3-methyl-6-isopropyl-*p*-benzoquinone; [h] natural substrate PQ—plastoquinone; [i] inhibitor NQNO—2*n*-nonyl-4-hydroxy-quinoline-*N*-oxide; [j] inhibitor SMA—stigmateline; [k] analog of PQ—DPQ—decylplastoquinone; [l] nature lipid MGDG—monogalactosyldiacylglycerol; [m] natural lipid SQDG—sulfoquinovosyldiacylglycerol; [n] natural or artificially added PG—phosphatidylglycerol; [o] artificially added synthetic PC—DOPC—1,2-dioleoyl-*sn*-glycero-3-phosphocholine. The chain designation refers to the *sn*-3 headgroup enantiomer. Other abbreviations: OPC—DOPC (18:1/18:1); 6PL—PC (16:0/18:0); SQD—SQDG (16:0/16:0); 1E2—SQDG (no chains); LMG—MGDG (18:0/18:0); 1O2—MGDG (16:0/18:1); OZ2—PG (13:1/18:1); PGV—PG (16:0/18:1); PGT—PG (16:0/18:0); 7PH—phosphatidic acid, PA (12:0/14:0) (it is relevant to note that in the PDB structure files 7PH are DAG, i.e., there is no phosphate group); DAG (diacylglycerols): 2WA (15:0/17:1), 2WD (5:0/6:0), 2WM (18:0/18:1), and 3WM (18:1/18:1); LFA—eicosane, 20-carbon lipid fragment; 8K6—18-carbon chain; MYS—15-carbon chain; OCT—8-carbon chain; UMQ—detergent UDM—*n*-undecyl-*ß*-D-maltopyranoside. PA and DAG are modeled when the lipid headgroup cannot be identified [8].

Text S1. Overview of the X-ray crystal and cryo-EM structures of dimeric cyt*b*$_6$*f* from different species.

As shown in Table S1, until 2014, all structures (from 1Q90 to 4PV1, totaling 13) were obtained using the X-ray crystallography method. They are all cyanobacterial species except for one algal organism, 1Q90. The same detergent, β-D-undecylmaltoside (UDM, PDB name: UMQ), and the synthetic lipid dioleoylphosphatidylcholine (DOPC) (18:1/18:1) were supplemented in the crystallization solutions for all structures except two. The

structure 4I7Z was crystallized using artificially added dioleoylphosphatidylglycerol (DOPG) (18:1/18:1) in place of DOPC [7]. The only structure that has been crystallized without the addition of synthetic lipids is 1Q90; it is stabilized by a His-tag [1]. Eight X-ray structures are co-crystallized with added synthetic quinone analog inhibitors (Table S1). These inhibitors mimic different redox states/positions of the native substrate plastoquinol/plastoquinone at the Q_p-site—the site of PQH_2 oxidation—and at the Q_n-site—the site of PQ reduction to PQH_2. When the lipid headgroup could not be identified, the lipids were modeled as phosphatidic acid and diacylglycerol (both noted as DAG lipids in [8]), along with hydrocarbon chains of varying lengths [8] (see Table S1). In all structures (Table S1), the resolved detergent molecules are markers of lipid-binding sites [14].

Since 2019, all eight structures—two from the cyanobacterium Synechocystis (7ZXY and 7R0W) and the remaining six from the spinach plant—have been obtained using the cryo-EM method. The first five (7ZXY, 7R0W, 6RQF, 7QRM, and 7ZYV) are without added inhibitors; most are with bound native substrate plastoquinone PQ (Table S1). The three very recent structures (9ES7, 9ES8, and 9ES9 [13]) were obtained under specific experimental conditions: 9ES7 and 9ES9—cyt*b*$_6$*f* was equilibrated (5 min) with an excess of DPQ (analog of PQ) and of the inhibitor DBMIB before freezing, respectively; 9ES8—cyt*b*$_6$*f* was exposed to substrates, reduced DPQ ($DPQH_2$) and oxidized plastocyanin and vitrified during catalytic turnover. All lipids in these structures are native and co-purified with cyt*b*$_6$*f*, except the three DOPC molecules in the spinach cyt*b*$_6$*f* dimer structure 6RQF [10] (Table S1). Egg yolk phosphatidylcholine was used during the cyt*b*$_6$*f* purification and is the origin of these three DOPC molecules, which are resolved in the dimer structure [10]. There are also two DOPCs in the cyanobacterium cyt*b*$_6$*f* dimer structure 7R0W [11], which are likely of the same origin (PCs are not discussed in [11]). As in the case of X-ray crystal structures, the same detergent UMD is resolved in the cryo-EM structures 7QRM and 7ZYV of spinach cyt*b*$_6$*f* [12] (Table S1). Since all structures were obtained from detergent solutions of the complex (i.e., the complex is not in nanodisks or in situ), variations in the number of the resolved lipids/detergent/hydrocarbons will depend only on the conformational state of cyt*b*$_6$*f* produced by the added inhibitor (mimicking different position/redox state of the native substrate) and/or the position of the native substrate. The isolation procedure and any specific conditions may also have an effect, but this will ultimately be reflected in the position of the substrate/inhibitor (e.g., [15]). It is thus evident that all these structures (Table S1) are not a database of structures determined using targeted variations in the lipids or detergents to study their effect on the cyt*b*$_6$*f* structure. Just the opposite; it is a database of different trapped conformational states of cyt*b*$_6$*f* (see also Section 3.1. in [16]), with variable lipid compositions. Therefore, the structures listed in Table S1 represent a valuable database that is useful for evaluating the response of the lipid compartment of cyt*b*$_6$*f* to changes in the protein's conformational state.

Two of the crystal structures (1VF5 and 2D2C) and four of the cryo-EM structures (6RQF, 7QRM, 7R0W, and 7ZXY) are asymmetric cyt*b*$_6$*f* dimers [16]. These monomeric cyt*b*$_6$*f* units, together with the symmetric ones, represent a total of 28 monomeric cyt*b*$_6$*f* that have different numbers of lipids and/or PQ in addition to the changed conformations registered by the three characteristic distances (hydrophobic thickness, p-gate width, and the n-side flexibility distance). The other four cryo-EM structures (7ZYV, 9ES7, 9ES8, and 9ES9) have applied C2 symmetry [12,13], and they do not differ in terms of the bound lipids and PQ.

Table S2. Occupation of lipid-binding sites in the various X-ray crystal structures of the cytochrome *b*$_6$*f* complex. In bold are listed the 4OGQ sites also occupied in other crystal structures. In **bold red** are the partially shielded sites (**p-L2(1)**, **p-L3(1)**, and **n-L5(4)**). In **bold green** is the site **p-L1(1)**, which is not occupied in the 4OGQ structure but is occupied in an older version of the 2E74 structure and in some cryo-EM structures, which are also occupied by a detergent (Table S3). In **bold blue**, the core–annular lipid-binding site **n/p-L5(2)** is presented. The attached number in front of the PDB ID for each monomeric structure corresponds to the p-gate value of the structure. The final ordering is based on the three distances together,

which can be used as a sum or as a calculated volume of a truncated cone with n- and p-distances representing its bigger and smaller radiuses, respectively, and the d_P as the height, which represents an empirical indicator characterizing the increase in the cyt*b₆f* dimer volume.

**No.-PDB**<br>**d_P [Å][a]**	**n-/p-side**<br>**d_n/$d_{p\text{-gate}}$**<br>**[Å][b]**	**Qn/Qp occupation**	**L1—β-side of Chl*a***	**L2—α-side of Chl*a* and Phe/Tyr124**	**L3—Carotenoid site**	**L4—SQDG dominated**	**L5—Phe40 and lipid–lipid dimer interface**	**Total n-/p-side**
3-4OGQ<br>30.6	n-side<br>27.3	8K6202	**n-L1(1)—OPC205**	**n-L2(1)—7PH206**;<br>n-L2(2)—UMQ101	**n-L3(1)—7PH104**;<br>n-L3(2)—1O2103 (*MGDG*)	**n-L4(1)—SQD204;**<br>**n-L4(2)—UMQ201;**<br>**n-L4(3)—UMQ304**;<br>n-L4(4)—UMQ201	**n-L5(1)—8K6202**;<br>**n-L5(2)—2WM309**;<br>n-L5(3)—8K6306;<br>**n-L5(4)—8K6307**;<br>**n-L5(5)—7PH305-m2**[c]	14
	p-side<br>6.5	—	—	**p-L2(1)—2WA101**	**p-L3(1)—3WM101**;<br>p-L3(2)—OCT102	**p-L4(1)—7PH203**;<br>p-L4(3)—2WD206;<br>p-L4(4)—7PH303	**p-L5(2)—MYS202**;<br>**p-L5(4)—8K6308**	8
4-4H44<br>31.6	n-side<br>27.5	UMQ307	**n-L1(1)—OPC202**	—	**n-L3(1)—OCT102**<br>(≡*sn-1chain*)	**n-L4(1)—SQD201** (*part of chains*);<br>**n-L4(2)—UMQ305;**<br>**n-L4(3)—UMQ304**	**n-L5(1)—UMQ307;**<br>**n-L5(5)—UMQ309-m2**<br>(≡*head & sn-1 chain*)	7
	p-side<br>6.6		—	**p-L2(1)—UMQ101**<br>(≡*sn-1 chain*)	**p-L3(1)—OPC302**	**p-L4(1)—7PH303**	**p-L5(2)—8K6308**<br>(≡*sn-2 chain of L5(2)*);<br>**p-L5(4)—MYS306**	5
5-2ZT9<br>31.2	n-side<br>27.2		**n-L1(1)—OPC202**	—	—	**n-L4(1)—SQD203;**<br>**n-L4(2)—UMQ306;**<br>**n-L4(3)—UMQ305**	—	4
	p-side<br>6.7		—	—	**p-L3(1)—OPC30**	**p-L4(1)—UMQ304**<br>(≡*sn-2 chain*)	—	2
8-4PV1<br>31.8	n-side<br>27.5<br>8 coupled	SMA308	**n-L1(1)—OPC205**	—	—	**n-L4(1)—SQD202;**<br>**n-L4(2)—UMQ203;**<br>**n-L4(3)—UMQ307**	**n-L5(1)—SMA308**;<br>**n-L5(4)—UMQ306**	5+SMA
	p-side<br>7.0		—	—	**p-L3(1)—OPC101**	**p-L4(1)—7PH309**	p-L5(1-2)—8K6201<br>(*interm. ≡ n-L5(1)-end and n-L5(2)-sn-1 chain end of 4OGQ*);<br>**p-L5(2)—OPC203**<br>**p-L5(4)—MYS301**	5

9-2E75<br>32.0	n-side<br>26.8	QNO501	**n-L1(1)—OPC1001**	—	—	**n-L4(1)—SQD201;<br>n-L4(2)—UMQ1104;<br>n-L4(3)—UMQ1103**	**n-L5(1)—QNO501;<br>n-L5(4)—UMQ1102**	5+NQNO
	p-side<br>7.1		—	—	**p-L3(1)—OPC1002**	**p-L4(1)—UMQ1101**<br>(*≡sn-2 chain*)	—	2
10-4H0L<br>31.4	n-side<br>27.2	QNO308	**n-L1(1)—OPC203**	—	—	**n-L4(1)—SQD201;<br>n-L4(2)—UMQ309;<br>n-L4(3)—UMQ307**	**n-L5(1)—QNO308;<br>n-L5(4)—UMQ306**	5+NQNO
	p-side<br>7.2		—	—	**p-L3(1)—OPC305**	**p-L4(1)—UMQ301**	—	2
11-2E74<br>31.2<br>(*old in OPM*)	n-side<br>27.1<br>8 coupled		**n-L1(1)—OPC202**	—	—	**n-L4(1)—SQD11;<br>n-L4(2)—UMQ1104;<br>n-L4(3)—UMQ1103**	**n-L5(4)—UMQ1102**	5
	p-side<br>7.3		**p-L1(1)—OPC1002**-<br>*sn*-2 (*older in OPM*)	**p-L2(1)—OPC1002**<br>*sn*-1 (*older in OPM*)	**p-L3(1)—OPC1002**<br>(*newer in PDB*)	**p-L4(1)—UMQ1101**<br>(*≡sn-2 chain*)	—	2
12-4I7Z<br>31.8<br>+DOPG, no Fe2S2	n-side<br>27.2		**n-L1(1)—OZ2203**<br>(*PG*)	—	**n-L3(1)—OCT101**<br>(*≡sn-1 chain*)	**n-L4(1)—1E2201**<br>(*SQDG-no chains*);<br>**n-L4(2)—UMQ308;<br>n-L4(3)—UMQ307**	**n-L5(4)—UMQ306**	6
	p-side<br>7.3		—	—	**p-L3(1)—OZ2102**<br>(*PG*)	**p-L4(1)—OZ2303**<br>(*PG*)	**p-L5(2)—8K6305**<br>(*≡sn-2 chain of L5(2)*);<br>**p-L5(4)—MYS304**	4
13-4H13<br>31.6	n-side<br>28.7	TDS201	**n-L1(1)—OPC204**	—	**n-L3(1)—OCT101**<br>(*≡sn-1 chain*)	**n-L4(1)—SQD201;<br>n-L4(2)—UMQ202;<br>n-L4(3)—UMQ308**	**n-L5(1)—TDS201**	5+TDS
	p-side<br>7.9	TDS	—	—	**p-L3(1)—OPC305**	**p-L4(1)—7PH301**	**p-L5(2)—8K6307**<br>(*≡sn-2 chain of L5(2)*);<br>**p-L5(4)—MYS306**	4
14-1Q90<br>31.8	n-side<br>28.1	?PQ	**Residual electron density**	—	—	**n-L4(1)—SQD950**	—	1
	p-side<br>8.2	TDS	—	**p-L2(1)—LMG953**	**p-L3(1)—LMG951**	**p-L4(1)—LFA960**<br>(*≡sn-2 chain*)	—	3
15-2E76<br>31.6	n-side<br>28.5<br>8 coupled	TDS1202	**n-L1—OPC1001**	—	—	**n-L4(1)—SQD201;<br>n-L4(2)—UMQ1104;<br>n-L4(3)—UMQ1103**	**n-L5(1)—TDS1202;<br>n-L5(4)—UMQ1102**	5+TDS

	p-side<br>8.4	TDS	—	—	**p-L3(1)—OPC1002**	**p-L4(1)—UMQ1101**<br>(≡*sn-2 chain*)	—	2
6-1VF5<br>monomer I<br>29.8	n-side<br>24.5	PQ	—	—	—	n-L4(1-3)—OPC1307	n-L5(1-2)—PL9305<br>(≡*sn-1 chain of n-L5(2)*)	1+PQ
	p-side<br>6.8	TDS-head out	—	—	—	—	**p-L5(2)—OPC1306**;<br>**p-L5(4)—TDS1304**-head	1+TDS
2-1VF5<br>monomer<br>A<br>29.8	n-side<br>24.2	PQ (PL9305)	—	—	—	n-L4(1-3)—OPC307	n-L5(1-2)—PL9305<br>(≡*sn-1 chain of n-L5(2)*)	1+PQ
	p-side<br>6.6	TDS-head out	—	—	—	—	**p-L5(2)—OPC306**;<br>**p-L5(4)—TDS304-head**	1+TDS
7-2D2C<br>monomer<br>A<br>28.6	n-side<br>25.8	—	—	—	—	n-L4(1-3)—OPC306	n-L5(1-2)—OPC305	2
	p-side<br>6.9	DBMIB out	—	—	—	—	—	0
1-2D2C1-<br>2D2C<br>monomer I<br>28.6	n-side<br>21.8	—	—	—	—	n-L4(1-3)—OPC1306	—	1
	p-side<br>5.5	DBMIB out	—	—	—	—	**p-L5(2)—OPC1305**	1

[a] Hydrophobic thickness of cytb_6f in Å, taken from the OPM database [17]; [b] d_n/$d_{p\text{-gate}}$ are the n-side distance Phe40-Phe/Tyr124 and the width of the p-side lateral gate (distance Ala147-Leu76), respectively, in Å; see Figure 3**b** in the main text. [c] m2 means that this lipid belongs to the other cytb_6f monomer, not to the monomer to which this binding site is assigned. Symbols used to describe the coincidence with the respective site occupant in 4OGQ X-ray crystal structure: ≡ complete coincidence, shift below 2 Å; ≅ almost but not entirely, shift below 4 Å; ≠ do not coincide, shift > 4 Å. The other abbreviations are as in Table S1.

Text S2. A detailed description of the five groups of lipidic binding sites in X-ray crystal structures of cytb_6f.

L1 group occupies the β-side of the Chl*a* macrocycle plane (the phytyl-binding side; see Figure 1**b** for the β-side of Chl*a* and Slides 2–8 in Suppl_Images-2):

1. **n-L1**—a lipid chain always contacts (distance < 4 Å) several C-atoms of the C pyrrole ring of Chl*a* from its β-side. In eight of eleven crystal dimer structures, this site is occupied by the synthetic lipid DOPC artificially added to the crystallization solution and by the physiological lipid DOPG artificially added to the crystallization solution in 12-4I7Z. In structure 14-1Q90, there is an unassigned electron density.
2. **p-L1(1)**—occupied only in an older 2E74 structure, still available in the OPM database. One of the DOPC lipid chains is on the β-side of Chl*a*, while the other chain with its headgroup is on the α-side (see below p-L2(1)). It is of note that the **p-L1(1)** binding site is occupied by a detergent in some cryo-EM structures (7, 8-7QRM and 9, 10-7ZYV in Table S3).

L2 group occupies the α-side of the Chl*a* macrocycle plane (the side of the carbomethoxy group bound to the fifth cyclopentanone ring of the macrocycle) and *fg*-loop Phe/Tyr124 residue at the n-side (stromal/cytoplasmic side) (Figure 3b, Figure 4b, see Slides 9-14 in Suppl_Images-2). The L2 consists of a maximum of three binding sites:

3. n-L2(1)—occupied only in 4OGQ by a DAG lipid contacting Phe124 and the next n-L2(2) detergent-occupied site. Both occupants shield Phe124 from direct exposure to the bulk lipid phase.
4. n-L2(2)—occupied only in 4OGQ by a detergent bound to Phe124 and to n-L2(1) and serves as a bridge between this *fg*-loop residue and the n-side-directed ring of β-Car (the next L3 group).
5. **p-L2(1)**—the lipid chain always contacts the *α*-side of the Chl*a* macrocycle with the O1D atom of the carbomethoxy group. It can also interact with the Mg^{2+} ion at the center of the tetrapyrrole macrocycle. This site is occupied in four structures: 3-4OGQ, 4-4H44, 11-2E74 (an earlier version in PDB and current in OPM), and 14-1Q90. This site is the first of the three partially shielded annular lipid-binding sites (see Subsection 3.2.2).

L3 group—carotenoid site (the carotenoid is β-carotene in all structures in Table S1, except in the cyanobacterium *Synechocystis* 6803 (7ZXY and 7R0W) wherein it is echinenone (Figure 3**a, b**, Figure 4**d**; details are provided in Slides 15-21 in Suppl_Images-2)). This group has a maximum of four binding sites (in 4OGQ):

6. **n-L3(1)**—the closest one from the left of β-Car. This site is occupied by a DAG (7PH104) in 3-4OGQ, and its *sn*-2 chain has several contacts with the n-side-directed ring of β-Car. In the other three structures (4-4H44, 12-4I7Z, and 13-4H13), an 8-C-atom-long chain occupies the place of the *sn*-1 chain of the DAG in 4OGQ and does not contact the β-Car ring.
7. n-L3(2)—occupied only in 4OGQ by a MGDG lipid. It is the next site on the left from β-Car.
8. **p-L3(1)**—the *sn*-1 lipid chain always contacts the protein core buried ionone ring of β-Car. As the n-L1 site, this site is always occupied in structures with hydrophobic thickness d_P equal to or above 30.6 Å. It is empty at a hydrophobic thickness of less than 30.6 Å. In eight of the eleven total crystal dimer structures, this site is occupied by the synthetic lipid DOPC artificially added to the crystallization solution and by the physiological lipid DOPG artificially added to the crystallization solution in 12-4I7Z. This site is the second one of the three partially shielded annular lipid-binding sites (see Subsection 3.2.2).
9. p-L3(2)—occupied only in 4OGQ by an 8-C-long hydrocarbon.

L4 group—the SQDG-determined group of sites (Figure 3, Figure 4e for 4OGQ; see Slides 22-40 in Suppl_Images-2 for the occupants in all crystal structures with a hydrophobic thickness value equal or bigger than 30.6 Å; for those with d_P < 30.6 Å, see Slides 62-79 in Suppl_Images-2). The L4 group consists of a maximum of seven lipidic binding sites (in 4OGQ):

10. **n-L4(1)**—SQDG. The SQDG head exhibits characteristic interactions with the transmembrane helices of both cyt*f* and Rieske ISP [8] (Figure 1). This site is occupied by the anionic SQDG in all 11 symmetric cytb_6f dimer structures, even when the other anionic lipid, DOPG (8-4I7Z), is added in excess for the crystallization of the cytb_6f complex [7]. SQDG, together with the next n-L4(2) and n-L4(3) detergent occupants, form a trimer either as a ring (r)-shaped cluster or as an open cluster, which is shown in Figure 5 and described in Section 3.3.1.
11. **n-L4(2)**—a detergent that is always bound to both n-L4(1) (SQDG) and n-L4(3) occupants.
12. **n-L4(3)**—a detergent that is always bound to n-L4(2) but not always to n-L4(1) (SQDG). This n-L4(3) site can provide a chain–chain intermonomer contact with the other monomer's n-L5(4) occupant. These three n-L4(1-3) sites are occupied by the same SQDG and two detergents in all the 11 symmetric dimer structures, except in 14-1Q90, wherein only SQDG is resolved at its n-L4(1) site. These n-L4(1-3) sites are occupied by a single neutral lipid, DOPC, in an intermediate position in the two asymmetric dimer structures, with protein hydrophobic thickness d_P < 30.6 Å—1VF5 (d_P = 29.8 Å) and 2D2C (d_P = 28.6 Å) (see Slides 62-79 in Suppl_Images-2). Their monomeric

structures (7-2D2C, 6-1VF5, 2-1VF5, and 1-2D2C, Table S2) with the occupation of the L4-L5 region are shown in Figure 6 and described in Section 3.3.2.

13. n-L4(4)—occupied only in 3-4OGQ by the detergent UMQ201. This detergent is distant from the n-L4(1-3) trimer. In the vertical direction, its chain coincides with the *sn*-2 chain end of the DAG occupant of the p-L4(4) site (see, in particular, Slides 24-29 in Suppl_Images-2 for a scrolling 3D view).
14. **p-L4(1)**—below the n-L4(1) (SQDG). The *sn*-1 lipid chain end coincides in the transmembrane (vertical) direction with the sulfur group of the SQDG in the n-L4(1) site. This site is always occupied when the p-gate ≥ 6.45 Å. This site is occupied by a lipid (DAG or PG) in five structures (3-4OGQ, 4-4H44, 8-4PV1, 12-4I7Z(PG), and 13-4H13), by a detergent in another five structures (5-2ZT9, 9-2E75, 10-4H0L, 11-2E74, and 15-2E76), and by an eicosane (20-C-long hydrocarbon, lipid fragment) in 14-1Q90. The hydrocarbon chain of the latter six occupants coincides with the *sn*-2 chain of the former five lipid occupants. This p-L4(1) site is always occupied for all 11 symmetric dimer structures, as is the SQDG site n-L4(1) in the opposite n-side monolayer. This site is empty in the two asymmetric dimer structures with a d_P value of less than 30.6 Å. These are the monomeric structures 7-2D2C, 6-1VF5, 2-1VF5, and 1-2D2C. Note that in these structures, the SQDG and the two detergents at n-L4(1-3) are substituted by a neutral lipid OPC (see above).
15. p-L4(3)—occupied only in 3-4OGQ by a DAG lipid 2WD206. In the vertical direction, its *sn*-2 chain coincides with the peripheral oxygen (O6) of the first headgroup of the detergent at n-L4(3) site.
16. p-L4(4)—occupied only in 3-4OGQ by a DAG lipid 7PH303. In the vertical direction, its *sn*-2 chain end coincides with the chain end of n-L4(4)-distant.

L5 group—around Phe40 and the lipid–lipid intermonomer (dimer) interface (Figure 4**e**; see the whole L5 group in different structures in Slides 41-45 in Suppl_Images-2). This group consists of a maximum of seven lipidic binding sites (in 3-4OGQ):

17. **n-L5(1)**—contacts both Phe40 and heme c_n. This is, in fact, the Qn-site wherein the Qn-site inhibitors bind and is the site for the reduction of the native substrate PQ. It is the closest site to the n-L4(1-3) group (see Slides 46-47 in Suppl_Images-2).
18. **n-L5(2)**—core–annular lipid (see above) on the core protein surface, which is part of the dimer hydrophobic cavity region and may also contact the heme c_n. This is the DAG lipid 2WM309 in 3-4OGQ. Its *sn*-2 chain coincides with the p-L5(2) occupants (8K6308 in 4-4H44, *sn*-2 chain of OPC203 in 8-4PV1, 8K6305 in 12-4I7Z, and 8K6307 in 13-4H13) (see Slides 48-50 in Suppl_Images-2).
19. n-L5(3)—occupied only in 3-4OGQ by an 18-C-long hydrocarbon chain 8K6306. This site is on the core protein surface of the dimer hydrophobic cavity region. It contacts the phytyl chain of Chl*a* (Figure 4e and see n-L5(3-5) in Slides 56-58 in Suppl_Images-2).
20. **n-L5(4)**—inter-monomer lipid–lipid contact position at the n-side center of the dimer cavity (the center of the entire cytb_6f dimer, represented by a large ball in all structures). This site is occupied by the 18-C-long hydrocarbon chain 8K6307 in 3-4OGQ. In the other structures, a detergent is resolved when the site is occupied. This site occupant can bridge the two monomers via head–head contact with the same occupant from the other monomer and can make intramonomer chain–chain contact with the n-L4(3) occupant (see Slides 51-55 in Suppl_Images-2). In the cytbc_1 X-ray structure 3CX5 [18], the headgroup region at the dimer interface is occupied by cardiolipin; the chain region overlaps with phosphatidylethanolamine (PE) from each monomer in 3CX5.
21. **n-L5(5)**—monomer–monomer connecting site, exposed on the protein surface close to the n-L1 site. It is occupied by a DAG (7PH305) in 3-4OGQ and by a detergent in 4-4H44; the detergent chain coincides with the *sn*-1 chain of the DAG (see Slides 56-58 in Suppl_Images-2). The occupant of the n-L5(5) belongs to the lipids of the second cytb_6f monomer but completes the lipid bilayer shell of the first monomer. The DAG connects the two monomers via chain–chain contact with the n-L5(4) (8K6307) of the other monomer and head contact with its

N-terminal of cytb_6 subunit (Asn7) and the n-side of the C-helix (cytb_6) and F-helix (suIV) of the other monomer. The n-L5(5) lipid-binding site is conserved for the cyt*bc* complexes; in cytbc_1, it is occupied by the PE lipid [19].

22. **p-L5(2)**—in 3-4OGQ, it is occupied by a 15-C-long hydrocarbon chain (MYS202), but it is occupied by a core–annular lipid (OPC203) in the 8-4PV1 structure. Its *sn*-2 chain coincides with the *sn*-2 chain of the **n-L5(2)** lipid in 3-4OGQ and with the 18-C-long hydrocarbon chain in three other structures (8K6308 in 4-4H44, 8K6305 in 12-4I7Z, and 8K6307 in 13-4H13 (see Slide 59 in Suppl_Images-2)).
23. **p-L5(4)**—closest to the p-side membrane interface. It is occupied by an 18-C-long hydrocarbon chain (8K6308) in 3-4OGQ and by a 15-C-long hydrocarbon (MYS) in four other structures (4-4H44, 8-4PV1, 12-4I7Z, and 13-4H44) (see Slide 59 in Suppl_Images-2).

Table S3. Occupation of lipid-binding sites in the various cryo-EM structures of cytochrome b_6f complex. Underlined in bold are the 4OGQ sites also occupied in cryo-EM structures. The **lipid, detergent, and hydrocarbon names are in bold to facilitate distinguishing them from the PQ substrate**. In **bold green** are sites (**p-L1(1)**, **p-L3(3)**, and **p-L4(5)**) not occupied in the 4OGQ structure. In **bold red** are the partially shielded sites (**p-L2(1)**, **p-L3(1)**, and **n-L5(4)**). In **bold blue**, the core–annular lipid-binding site **n/p-L5(2)** is given.

No.-PDB d_P [Å][a]	n-/p-side d_n/$d_{p\text{-gate}}$ [Å][b]	Qn/Qp occupation	L1—β-side of Chl*a*	L2—*α*-side of Chl*a* and Phe/Tyr124	L3—Carotenoid site	L4—SQDG site	L5—Phe40 and lipid–lipid dimer interface	Total n-/p-side
4OGQ 30.6	n-side 27.3	8K6202	**n-L1—OPC205**	**n-L2(1)—7PH206**; n-L2(2)—UMQ101	n-L3(1)—7PH104; n-L3(2)—1O2103 (*MGDG*)	**n-L4(1)—SQD204;** **n-L4(2)—UMQ201;** **n-L4(3)—UMQ304;** n-L4(4)—UMQ201	**n-L5(1)—8K6202;** **n-L5(2)—2WM309**; n-L5(3)—8K6306; n-L5(4)—8K6307; **n-L5(5)—7PH305-m2**[c]	14
	p-side 6.5		—	**p-L2(1)—2WA101**	**p-L3(1)—3WM101**; p-L3(2)—OCT102	**p-L4(1)—7PH203**; **p-L4(3)—2WD206**; p-L4(4)—7PH303	**p-L5(2)—MYS202**; p-L5(4)—8K6308	8
Syn. 6803 1-7ZXY-I **-PetP** 31.4	n-side 27.3		**n-L1—PGV202** (≅ *head + Chla contact of sn-2 chain*)	**n-L2(1-2)—PGV203** (*sn-2 chain b/n 1-2 and contact with Phe124*)	—	—	—	2
	p-side 6.0		—	**p-L2(1)—PGV204** (*open chains, sn-1 contact with O1D, sn-2 chain is 21 Å, very outside*)	—	**p-L4(1)—PGV302** (≅*part of the chains*)	—	2
Syn. 6803 2-7ZXY-A **-PetP** 31.4	n-side 27.4		**n-L1—PGV202** (≅ *head + Chla contact of sn-1 chain*)	—	—	—	—	1
	p-side 6.1		—	**p-L2(1)—PGV203**	**p-L3(3)—LFA101**	**p-L4(1)—PGV303** (≅ *part of the chains*);	—	4

				(open chains, sn-2 contact with O1D, sn-1 chain is 7 Å outside)		**p-L4(5)—LFA302**		
Syn. 6803 3-7R0W-I **+PetP** 32.6	n-side 26.9	PL9201 (PQ)	**n-L1—PGV203** *(closer extended chains, shorter chains—more mobile ends than in mon. A)*	—	—	**n-L4(1)+n-L4(3)—SQD201** *(head and sn-1 chain ≅ n-L4(1); sn-2 chain ≅ n-L4(3) chain)*	n-L5(1)+**n/p-L5(2)**—PL9201 **(***head ≅ n-L5(1), chain ≅ sn-2 chain n/p-L5(2)*)	2 + 1 PQ
	p-side 6.6		—	**p-L2(1)—LMG101** *(closer chains than in 1-2 (-PetP), sn-1 contact with O1D)*	**p-L3(1)—6PL101** **(***PC,* ≅*; contact with echinenone)*	**p-L4(1)—PGV304** *(≅ chains)*	—	3
Syn, 6803 4-7R0W-A **+PetP** 32.6	n-side 27.1	PL9303 (PQ)	**n-L1—PGV304** *(closer, extended chains than in 1-2 (- PetP), full-length chains)*	—	—	**n-L4(1)+n-L4(3)—SQD202** *(head and sn-1 chain ≅ n-L4(1); sn-2 chain ≅ n-L4(3) chain)*	n-L5(1)+**n/p-L5(2)**—PL9303 **(***head ≅ n-L5(1), chain ≅ sn-2 chain n/p-L5(2)*)	2 + 1 PQ
	p-side 6.6		—	**p-L2(1)—2WA303** *(closer, extended chains, sn-1 contact with O1D)*	**p-L3(1)—6PL101** (*PC*, ≅*; no contact with echinenone);* **p-L3(3)—LFA101**	**p-L4(1)—PGV301** *(≅ chains)*	—	**4**
Spinach 5-6RQF-A 31.8	n-side 28.2	PL9306 (PQ3 in [10]) distant	**n-L1—PGV202** *(sn-1 chain–ring C contact, chains closer)*	—	—	**n-L4(1)+n-L4(3)—SQD202** *(head and sn-1 chain ≅ n-L4(1); sn-2 chain ≅ n-L4(3) chain)*	—	**2**
	p-side 6.1	PL9305 (PQ1 in [10]) distant	—	**p-L2(1)—LMG201** *(sn-1 chain end contact with O1D; sn-2 is outside not toward the n-side)*	**p-L3(1)—6PL101** (*PC*, ≅*; contact* with β-*Car*)	**p-L4(1)—PGV308** *(sn-1 chain ≡ sn-2 of p-L4(1); others –moved 4 Å)*	**p-L5(2)+p-L4(3)—SQD307** *(head ≅ p-L5(2) and P-group≡ TDS-end, sn-2 chain ≅ p-L4(3), chains parallel to the p-side plane, contacts phytyl of m2-I)*	**4**
Spinach 6-6RQF-I 31.8	n-side 28.5	PL9306 (PQ2 in [10])	**n-L1—PGV202** *(sn-2 chain – ring C contact, chains more opened)*	—	—	**n-L4(1)+n-L4(3)—SQD202** *(head and sn-1 chain ≅ n-L4(1); sn-2 chain ≅ n-L4(3) chain)*	n-L5(1)+**n/p-L5(2)**—PL9306 *(head ≅ n-L5(1), chain partly ≅ sn-2 chain of n/p-L5(2))*	**2** + 1 PQ
	p-side 6.8		—	**p-L2(1)—LMG201** *(sn-2 chain end contact with Mg and O1D; sn-1 is outside not toward the n-side)*	**p-L3(1)—6PL101** **(***PC*, ≅*; contact with* β-*Car*)	**p-L4(1)—PGV302** *(sn-2 chain ≡ sn-2 of p-L4(1); others–moved 3 Å);* **p-L4(3)—6PL307**	**p-L5(2)**—PL9305 *(head ≅ p-L5(2), directed to the TDS-end and contacts phytyl of m1-A)*	**4** +1 PQ

						(≡ in n/p-view; in front view to below > 4-5 Å)		
	n-side 28.8	—	**n-L1—UMQ302** (contacts Mg, one C-ring atom)	—	—	**n-L4(1)—SQD201; n-L4(2)—UMQ307; n-L4(3)—UMQ306**	**n-L5(5)—PGT308-m2** (≡ in n/p-view; in front view shifted up + more vertical max. 4-5 Å)	**5**
Spinach 7- 7QRM-A **-TSP9** 32.2	p-side 7.2	PL9305 (PQ2 in [12] chain end)	**p-L1(1)—UMQ303** (no contact with Chla); p-L1(2)—PL9305 (PQ2, new for PQ: circles phytyl and contacts it, part chain ≅ p-L5(4); chain end at 3.5 Å to the p-side gate and ≡ TDS-head in Qp-site)	**p-L2(1)**—PL9301 (PQ1, ≅ sn-1 chain of lipid at p-L2(1) and contact with O1D)	**p-L3(1)—UMQ201** (its chain ≡ sn-1 chain of p-L3(1) lipid; no contact with β-Car)	p-L4(1)+**n/p-L5(2)**—PL9202 (PQ3 ≡ head+sn-2 chain of p-L4(1) and end ≡ part of sn-2 of n/p-L5(2)); **p-L4(3)+p-L5(2)—PGT204** (≅ part head+sn-1 of p-L4(3); sn-2 chain ≡ p-L5(2)-MYS202)		**3** + 3 PQ
	n-side 28.8	PL92001 (PQn in [12])	**n-L1—UMQ205** (contacts Mg, two C-ring atoms)	—	—	**n-L4(1)—SQD302; n-L4(2)—UMQ2005; n-L4(3)—UMQ2006**	n-L5(1)—PL92001; **n-L5(5)—PGT2007-m2** (≡ in n/p-view; in front view shifted up + more vertical max. 4-5 Å)	**5** + 1 PQ
Spinach 8- 7QRM-I **-TSP9** 32.2	p-side 7.3	PL9202 (PQ2 in [12] chain end)	**p-L1(1)—UMQ204** (no contact with Chla); p-L1(2)—PL9202 (PQ2, new for PQ: circles phytyl and contacts it, part chain ≅ p-L5(4); chain end at 1.7 Å to the p-side gate and ≡ TDS-head in Qp-site)	**p-L2(1)**—PL9203 (PQ1, ≅ sn-1 chain of lipid at p-L2(1) and contact with O1D)	**p-L3(1)—UMQ102** (its chain ≡ sn-1 chain of p-L3(1) lipid; no contact with β-Car)	p-L4(1)+**p-L5(2)**—PL92008 (PQ3, ≡ head+sn-2 chain of p-L4(1) and end ≡ part of sn-2 of n/p-L5(2)); **p-L4(3)+p-L5(2)—PGT801** (≅ part head+sn-1 of p-L4(3); sn-2 chain ≡ p-L5(2)-MYS202)	—	**3** + 3 PQ
Spinach 9, 10-	n-side 28.8		**n-L1—UMQ404/403** (contact with Mg only)	—	—	**n-L4(1)–SQD401/201; n-L4(2)–UMQ305/306; n-L4(3)–UMQ306/305**	—	**4**

7ZYV-I/A<br>**+TSP9**<br>32.4	p-side<br>7.3	PL9202<br>(PQ2 in [12] - end)	**p-L1(1)–UMQ403/404**<br>(*no contact with Chla*)	**p-L2(1)**—PL9402<br>(*PQ1, ≅ sn-1 chain of lipid at p-L2(1)* and contact with *O1D and CED*)	**p-L3(1)—UMQ102/201**<br>(*no contact with β-car*)	p-L4(1)—PL9302/9307<br>(*PQ3, ≡ head+sn-2 chain of p-L4(1)*)	—	**2** + 2<br>PQ
Spinach<br>11, 12-<br>9ES7-I/A<br>**+TSP9**	n-side<br>28.6		—	—	—	**n-L4(1)–SQD201;**<br>**n-L4(2)–UMQ304;**<br>**n-L4(3)–LMG305**<br>(*n-L4(3) ≡ MGDG-head+sn-2 chain*)	—	**3**
	p-side<br>7.2		—	—	**p-L3(1)—UMQ101**<br>(*≡ with head and sn-1 chain of 6RQF, no contact with β-car*)	—	—	**1**
Spinach<br>13, 14-<br>9ES8-I/A<br>**+TSP9**	n-side<br>28.7	DQE307<br>(DPQ,<br>A1H65)	—	—	—	**n-L4(1)–SQD201;**<br>**n-L4(2)–UMQ202;**<br>**n-L4(3)–LMG305**<br>(*n-L4(3) ≡ MGDG-head+sn-2 chain*)	n-L5(1)—DQE307	**3**+DPQ
	p-side<br>7.3		—	—	**p-L3(1)—UMQ101**<br>(*no contact with β-car*)	—	—	**1**
Spinach<br>15, 16-<br>9ES9-I/A<br>**+TSP9**	n-dist.<br>28.5		—	—	—	**n-L4(1)–SQD201;**<br>**n-L4(2)–UMQ304;**<br>**n-L4(3)–LMG305**<br>(*n-L4(3) ≡ MGDG-head+sn-2 chain*)	—	**3**
	p-side<br>8.3	DBMIB<br>(BNT306)	—	—	**p-L3(1)—UMQ102**<br>(*no contact with β-car*)	—	—	**1**

[a] Hydrophobic thickness of cytb_6f in Å, taken from the OPM database [17]; [b] $d_n/d_{p\text{-gate}}$ are the n-side distance Phe40-Phe/Tyr124 and p-side gate width Ala147-Leu76, respectively, in Å [16]; see Figure 1b in the main text. [c] m2 means that this lipid belongs to the other cytb6f monomer, not to the monomer to which this binding site is assigned. The remarks in italics relate to a comparison with the respective lipid-binding sites in the 4OGQ structure. Symbols used to describe the coincidence with the respective site occupant in 4OGQ X-ray crystal structure: ≡ complete coincidence, shift below 2 Å; ≅ almost but not entirely, shift below 4 Å; ≠ do not coincide, shift > 4 Å. The other abbreviations are as in Table S1.

Text S3. A detailed description of the five groups of lipidic binding sites in cryo-EM structures of cyt*b*6*f*.

Table S3 presents lipid-binding sites resolved by the cryo-EM method in dimer structures ordered in the direction of p-gate width increase. Again, 4OGQ [8] is included for easier comparison. As shown in Table S3, the p-gate width increases from 6.0 to 8.3 Å, the n-side distance rises from 26.9 to 28.8 Å, and the hydrophobic thickness d_P changes stepwise from 31.4 to 32.6 Å. From Table S2, it is seen that the respective ranges in X-ray structures are 5.5–8.4 Å, 21.8–28.7 Å, and 28.6–32 Å.

L1 group—occupies the β-side of the Chl*a* macrocycle plane:

n-L1—it is occupied by the native lipid PG (16:0/18:1) in the first six monomeric units (p-gate = 6.0-6.8 Å), a detergent in the next four (p-gate = 7.2-7.3 Å), and is empty in the remaining six monomeric structures (7.2-8.3 Å) (Table S3). The headgroups of the artificially added DOPC and DOPG in X-ray structures, as well as the headgroup of the native lipid PG in cryo-EM structures, form 2-3 H-bonds with the *fg*-loop residues Asn118, Val128, and Ala129. On this basis, it can be proposed that the most probable occupant of the n-L1 site in situ should be the **native anionic PG lipid** (see Table 4). As with X-ray crystal structures, the n-L1 occupant is not changeable. It is a site for permanent signaling from Chl*a* to the n-side-positioned *fg*-loop and also by the mediating role of Tyr105 of the B-helix of cytb_6 [15], which is always in contact with the n-L1 lipid.

p-L1(1) and p-L1(2)—**p-L1(1)** is a **new** site compared to the sites in the X-ray structures. It should be noted that in the older PDB structure 11-2E74, which is the OPM file for this structure, one of the chains of the occupant at p-L2(1) is located on the β-side of the Chl*a* macrocycle, while the other is on the α-side. The site p-L1(1) is occupied by the detergent UMQ only in the 7, 8-7QRM and 9, 10-7ZYV [12]. However, the UMQ chain of p-L1(1) does not contact the β-side of Chl*a* as the n-L1 occupant in all crystals (Table S2) and the other cryo-EM structures (Table S3). p-L1(2) is a site for PQ binding. This site is new relative to PQ/inhibitor-binding sites resolved by X-ray crystallography [12].

L2 group—occupies the α-side of the Chl*a* macrocycle plane and may contact with Phe/Tyr124:

n-L2(1, 2)— they are occupied only in structures with **the smallest p-side lateral gate, n-side plasticity distance, and hydrophobic thickness, similar to 4OGQ.** It is occupied by a PG at an intermediate position compared to 4OGQ. Based on this, **a native PG lipid** is expected to occupy the region around Phe124 in situ. Since structure 1, 2-7ZXY has the smallest dp-gate (6.0-6.1 Å) and hydrophobic thickness d_P among the cryo-EM structures, it might be possible to propose that at this conformational state of cytb_6f, Phe124 is shielded by an annular lipid from the bulk lipid phase, just as in X-ray structures 4OGQ and 4H44 (Table S2).

p-L2(1)—this site is always occupied in the cryo-EM structures. At the smallest p-gate and d_P determined by cryo-EM (the two monomers 1,2-7ZXY), this site is occupied **by a PG** (16:0/18:1), which again suggests a **native PG lipid in situ at this small p-gate width of 6.0-6.1 Å.** Then, at a larger p-gate (6.1-6.8 Å) and d_P, it is occupied **by MGDG** (18:0/18:0) (monomers 3, 5, 6). At the p-gate of 7.2-7.3 Å, the site is occupied by a PQ chain that is 45 C-atoms long (structures 7-10 in Table S3). This site is empty in the newest structures, p-gate of 7.2-8.3 Å. Hence, this site most closely responds as a site for temporally compensating for the positive hydrophobic mismatch with a substitution of PG (16:0/18:1) and a sorting of lipids with longer saturated chains and negative spontaneous (intrinsic) curvature—MGDG (18:0/18:0). Thus, this result confirms the result from X-ray structures, related to this site.

L3 group—carotenoid-dominated group:

n-L3 – it is empty in the cryo-EM structures.

p-L3(1) – at the smallest p-gate, n-plasticity distance, and d_P (dimer 1-, 2-7ZXY), this site is empty. Then, the site is occupied by PC in the next three structures (3, 4, 5) with the *sn*-1 chain toward the n-L3 lipid MGDG in 4OGQ. All others have this chain outside the complex, like an antenna buried in the bulk lipid phase. The p-side lipid head is more mobile than the chains.

p-L3(3) is occupied only in the monomer 2-7ZXY and 4-7R0W structures by an eicosane (LFA101).

L4 **group**—SQDG-dominated group of sites:

n-L4(1-3)—at the smallest p-gate (p-gate = 6.0-6.1 Å), n-plasticity distance, and d_P (dimer 1-, 2-7ZXY), this group of sites is empty, thus indicating that, indeed, the lipid response to reaching the conformation with the smallest p-gate and d_P is the SQDG release from its

binding site. In all other structures characterized first by the increased d_P but still smaller p-gate (p-gate = 6.1-6.8 Å), SQDG occupies the empty site at an intermediate position between n-L4(1) and n-L4(3) in structures 3-6 in Table S3. Then, at the larger p-gate detected by cryo-EM (7.2-7.3 Å) in structure 7, 8-7QRM, this group of sites is occupied by SQDG and two detergents, as in the X-ray structures (Table S2). Then, in the newest 11-16 ones (9ES7, 9ES8, and 9ES9), the MGDG (18-C) with its head and *sn*-2 chain substitutes the detergent at the n-L4(3) site. Interestingly, the second part of the *sn*-2 chains of MGDG and SQDG move synchronously in the 9ES9 structures relative to the first two (9ES7 and 9ES8). Hence, as suggested by cytb_6f crystal structures, this site responds to the altered conformation of cytb_6f and is the site where an exchange of lipids occurs during the conformational dynamics of cytb6f, characterized by a decreasing p-gate and d_P. Notably, comparing the cytb_6f 9ES7-9 structure with the series of cytbc_1 (complex $III_{2)}$ of [20], e.g., 8AB8, SQDG at n-L4(1) in cytb_6f corresponds to the phosphatidylethanolamine (PTY504) in cytbc_1. The MGDG at n-L4(3) in cytb_6f overlaps its glycerol backbone and the first half of its chains with the cardiolipin CDL3001 in cytbc_1, while the second half of the MGDG chains in cytb_6f overlap with the cardiolipin CDL3002 in cytbc_1. The detergent at n-L4(2) in cytb_6f is close to part of the cardiolipin CDL3001 in cytbc_1.

p-L4—in most of the structures (1-6, p-gate = 6.1-6.8 Å, Table S3), p-L4(1) is occupied by a PG (16:0/18:1), thus indicating that the **native occupant can be a PG** in place of the DAG in the crystal structures (Table S2). At a p-gate of 7.2-7.3 Å (structures 7-10), this site is partially occupied by the native substrate PQ displacing the PG from its p-L4(1) binding site (structure 8-7QRM).

p-L4(5) is a new pose and is occupied only in the monomer 2-7ZXY (p-gate = 6.1 Å) by an eicosane (LFA302). Although there is an eicosane in the crystal structure 1Q90 at the p-L4(1), their positions differ.

L5 group—around Phe40 and the lipid–lipid intermonomer (dimer) interface:

n-L5(1)—PQ mainly occupies these sites and it can be empty or an SQDG (5-6RQF) and PG (7-8-7QRM) can also transiently bind there.

n-/p-L5(2)—lipids that coincide with the native substrate PQ in recent structures define a role for those lipids to form a part of the hydrophobic diffusion pathway for the native substrate—plastoquinone/ol (PQ) into and out of the Qp and cn pockets. The overlapping of PQ molecules with lipid sites in the recent spinach cytb_6f structures 7QRM and 7ZYV [12] demonstrates the role of these lipids in providing a hydrophobic pathway for PQ diffusion to and from the Qp-site. It can also be seen in Table S3 that the *cytb$_6$f* structures of Synechocystis and spinach have similar native lipids.

n-L5(4)—this partially shielded site is not occupied in cryo-EM structures of cytb_6f.

Text S4, Table S4, Table S5, and Figure S1. Comparison of cytb6f and cytbc1 X-ray crystal and cryo-EM structures (related to Subsection 3.1.2 in the main text).

To understand why at equally small p-gate widths, i.e., p-gate ≤ 6.6 Å, the hydrophobic thickness d_P for cryo-EM structures is larger by 1-2 Å than for X-ray structures of cytb_6f , the mean distances between the metal centers Fe of the hemes for both cytbc (cytb6f and cytbc1) and between the Mg of the two Chla in the cytb6f dimer in cryo-EM structures were compared with those in X-ray structures (Table S14). The number of selected X-ray and cryo-EM cytbc1 structures equals the respective cytb6f structures. In addition, the included cryo-EM structures of cytbc_1 are only those that are not part of a supercomplex with the other proteins of the respiratory electron transport chain, e.g., with complex I [21] or with complex I and

complex IV in situ [22]. The reason for this is that the involvement of cytbc1 in such supercomplexes either increases or decreases its hydrophobic thickness (as calculated in the OPM database [17]), and a tilting of the complex may also occur (as noted in the OPM database [17]).

Table S4. Mean heme (Fe-to-Fe) and Chl*a*(Mg)–Chl*a*(Mg) distances (Å, ± SD) from diverse X-ray crystal and cryo-EM structures of cyt*bc* dimers.

Method and cyt*bc* (number of monomeric structures)	Chl(Mg)–Chl(Mg)	*b*p(L)–*b*p(L)	*b*n(H)–*b*n(H)	*b*p(L)–*b*n(H)	*b*p(L)–*f* (c_1)	*b*n(H)–*f* (c_1)
X-ray cytb_6f (n=13, 15) [a]	61.1 ± 0.4	22.1 ± 0.1	34.9 ± 0.2	20.6 ± 0.2	44.6 ± 0.2	57.2± 0.2
**Cryo-EM cytb_6f (n=8, 12)**	**62.4 ± 1.6**	**22.6 ± 0.5**	**35.9 ± 0.8**	**21.0 ± 0.6**	**45.5 ± 1.3**	**58.4 ± 1.7**
X-ray cytbc_1 (n=15) [b]		21.0 ± 0.3	33.7 ± 0.4	20.7 ± 0.2	34.4 ± 0.2	51.0 ± 0.4
Cryo-EM cyt bc_1 (n=9, 11) [c]		21.1 ± 0.4	33.1 ± 0.8	20.5 ± 0.4	34.3 ± 0.5	50.9 ± 0.7

[a] The number(s) in brackets represents the number of analyzed dimers (the first three columns) and monomers (the remaining three columns). For cytb_6f, all the available 21 dimers and 26 asymmetric monomers are from Table S1. [b] The included X-ray crystal cytbc_1 structures are selected from Table 4 in [16] with the idea being to cover the whole range of p-gate width values and to have the highest resolution: 1P84 [23], 4PD4 [24], 1SQX [25], 1PP9 [26], 3CX5 [18], 2QJY [27], 2FYU [28], 1L0L [29], 3L75 [30], 1SQQ [25], 1SQB [25], 3TGU [31], 1NTM [32], 1BGY [33], 3H1H [34]. [c] The respective cytbc_1 cryo-EM structures are: 8AB7 [20], 7TZ6-P,C [35], 8ASI-F [36], 7TLJ [37], 8ASI-B [36], 8AB9 [20], 7RJE [38], 7RJA [38], 8AB6 [20], and 6KLV [39].

As seen in Table 1, the cryo-EM cytb_6f structures (the row in bold) have larger Chl(Mg)–Chl(Mg), b_n–b_n, b_p–f, and b_n–f distances ~ 1 Å than the X-ray crystal cytb_6f structures. This result indicates that, on average, the cryo-EM cytb_6f structures are more swollen in the n-side membrane half of the protein, and the heme *f* is more distant from the membrane part of cytb_6f in cryo-EM than in the crystal cytb_6f structures. In contrast, no such difference is observed between the cytbc_1 cryo-EM and X-ray crystal structures (Table S4). This means that the more swollen cryo-EM structures of cytb_6f are not due to the structure determination method. This may reflect the more plastic structure of cytb_6f relative to cytbc_1 in the n-side leaflet and the more distant heme *f* from the membrane hemes in cytb_6f relative to heme c_1 in cytbc_1 (see Figure S1). Since the hydrophobic thickness of cytb_6f exhibits a very strong positive linear correlation with the n-distance in crystal structures (r = 0.928, P < 0.001) [16], the larger n-distance may explain the larger hydrophobic thickness of cytb_6f in cryo-EM structures.

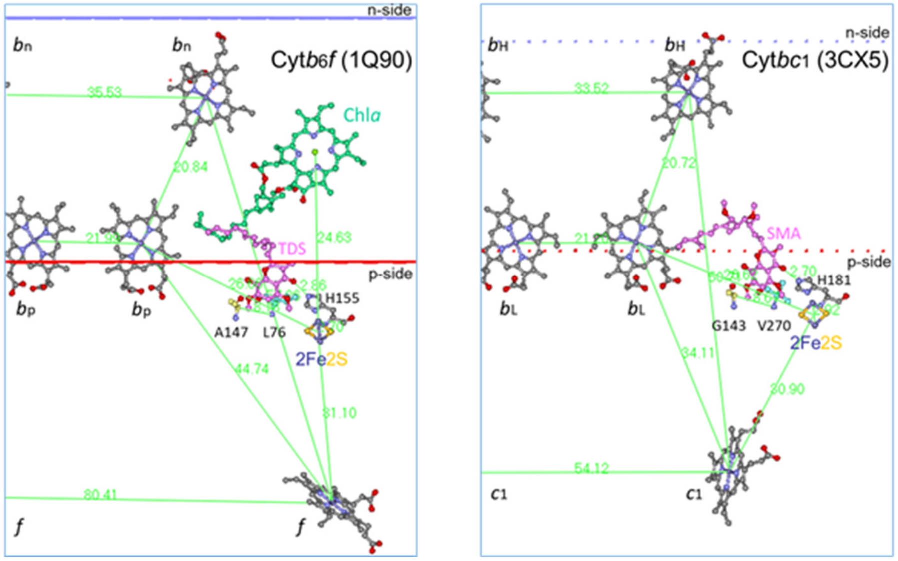

(a) **(b)**

Figure S1. Heme–heme and [2Fe-2S]–metal center distances in cytochromes *bc* complexes with [2Fe-2S] cluster at *b*, fixed position in X-ray crystal structures. (a) Cytochrome b_6f with the bound TDS inhibitor at the Qp-site (OPM 1Q90 [1]); (b) cytochrome bc_1 with the bound SMA inhibitor at the Qp(o)-site (OPM 3CX5 [40]). The distances in green are in Å. The blue and red lines mark the calculated electronegative n-side (blue) and electropositive p-side (red) lipid hydrophobic boundary planes (at the level of carbonyls) of a modeled lipid bilayer, which is in hydrophobic matching with the embedded protein, as provided by the OPM database [17].

As seen in Figure 1, the heme *f* in cytb_6f is more distant from the heme b_p and heme b_n by 10-11 Å and 6-7 Å, respectively, compared to the heme c_1 in cytbc_1. This is reflected in the enlarged distance between the hemes *f* in the cytb_6f dimer (~ 80 Å) relative to the distance between the hemes c_1 in the cytbc_1 dimer (54 Å).To see whether there is an effect of the structure method on the [2Fe-2S] position, the [2Fe-2S] center–Fe-hemes' distances in cyt*bc* complexes in X-ray crystal and cryo-EM structures with the [2Fe-2S] cluster at the *b*, fixed position (i.e., there is an H-bond between the inhibitor/substrate and the His residue from loop 2 of the ISP-ED which is a ligand to the [2Fe-2S] cluster) are compared in Table S5.

Table S5. [2Fe-2S] center–Fe-heme distances in cyt*bc* complexes in X-ray crystal and cryo-EM structures with the [2Fe-2S] cluster at the *b*, fixed position (H-bond between the inhibitor/substrate and the His residue from loop 2 of the ISP-ED which is a ligand to the [2Fe-2S] cluster).

Method and cyt*bc* (number of monomeric structures)	[2Fe-2S]–$b_{p(L)}$ Å	[2Fe-2S]–$b_{n(H)}$ Å	[2Fe-2S]–$f(c_1)$ Å
**X-ray cytb_6f (n=3) [a]**	**26.8 ± 0.0**	**34.8 ± 0.1**	**31.0 ± 0.1**
Cryo-EM cytb_6f (n=1) [b]	28.1	36.6	32.5
**X-ray cytbc_1 (n=25) [c]**	**26.8 ± 0.3**	**34.6 ± 0.3**	**31.3 ± 0.4**
**Cryo-EM cyt bc_1 (n=4) in complex [d]**	**26.8 ± 0.1**	**34.6 ± 0.1**	**31.3 ± 0.1**
Cryo-EM cyt bc_1 (n=5) [e]	27.3 ± 0.6	35.2 ± 0.7	31.7 ± 0.5

[a] The included X-ray crystal cytb_6f structures are 1Q90 (+TDS (Qp), ?PQ (Qn)) [1], 2E76 (+TDS (Qp, Qn)) [4], and 4H13 (+TDS (Qp, Qn)) [6]; p-gate range (8.4-7.9 Å). [b] Cryo-EM cytb_6f structure 9ES9 (+DBMIB (Qp)), p-gate = 8.3 Å [13]. [c] X-ray crystal structures of cytbc_1 taken from Table 4 (the first group with the p-gate range 9.8-8.1 Å) in [16]. [d] The included cryo-EM monomeric unit structures of cytbc_1 (complex III_2) from the supercomplex with complex I are 8BPX-x, 8BQ5-x, 8BQ6-x, and 8BEL-C (+UQ5 (Qp), UQ5 (Qn)) [21], with a p-gate range of (7.4-7.1 Å). [e] The included cryo-EM cytbc_1 structures are 8AB7 (+Atovaquone (Qp) and Antimycin A (Qn)) [20], 7TZ6-P,C (+ck-2-68 (Qp)) [35], 8ASI-F (+UQ10 (Qp)) [36], and 8AB8 (+dUQ (Qp)) [20].

As seen from Table S5 in bold, the *b*, fixed position of the [2Fe-2S] center in crystal cytb_6f [16], crystal cytbc_1, and cryo-EM cytbc_1 structures in a supercomplex with the respiratory complex I is strictly equivalent concerning the [2Fe-2S] distances to the three heme metal centers. The only available cryo-EM cytb_6f structure with the [2Fe-2S] cluster at the *b*, fixed position (its His128 ligand, in spinach numbering, is H-bonded to the inhibitor DBMIB) is the very recent 9ES9 [13] cytb_6f structure with the bound DBMIB at the Qp-site (unbold row). The three distances in the 9ES9 structure of detergent-solubilized cytb_6f are longer than those in the crystal cytb_6f and cytbc_1 structures but closer to those of detergent-solubilized [20,35] and nanodisk-solubilized [36] cryo-EM structures of cytbc_1 (Table 2, unbold). Note that these cryo-EM cytbc_1 structures possess more considerable variations in these three distances. Interestingly, the detergent- and nanodisk-solubilized cytbc_1 and detergent-solubilized cytb_6f cryo-EM structures have a p-gate width (p-gate = 9.2–8.1 Å) within the same p-gate Range 1 as the X-ray crystal structures (p-gate = 9.8–8.1 Å). The only outlier is the cryo-EM of cytbc_1 in complex with the complex I (in bold, p-gate = 7.4–7.1 Å). The reason for this is currently not apparent. Other factors, such as the decreased hydrophobic thickness of the complexed cytbc_1 and/or the smaller headgroup of the native substrate, may probably have something in common. It remains to be seen whether the [2Fe-2S]–Fe-heme distances will be shortened when cytb_6f is in a supercomplex or in situ. In short, Table S5 shows that the structure method has some effect on the [2Fe-2S]-Fe-heme distances for both cyt*bc* complexes.

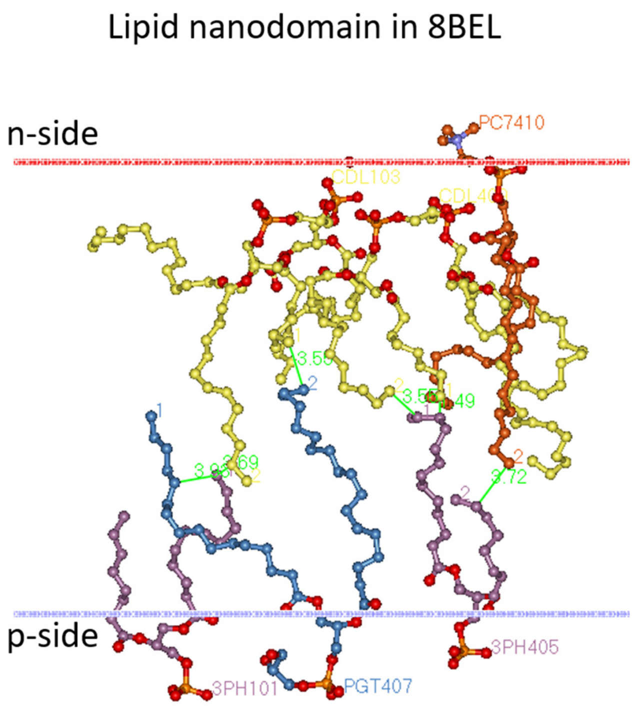

Figure S2. Lipid nanodomain at the interface between cytbc_1 (complex III) and complex I (8BEL, [21]). There are transmembrane contacts between the n- and p-side lipids. However, they disobey the chirality rule characteristic for the signaling lipid nanodomain in the 4OGQ crystal structure of cytb_6f (Section 4.4 in the main text).

References:

1. Stroebel, D.; Choquet, Y.; Popot, J.L.; Picot, D. An atypical haem in the cytochrome *b6f* complex. *Nature* **2003**, *426*, 413–418. https://doi.org/10.1038/nature02155
2. Kurisu, G.; Zhang, H.; Smith, J.L.; Cramer, W.A. Structure of the cytochrome b6f complex of oxygenic photosynthesis: tuning the cavity. *Science* **2003**, *302*, 1009–1014. https://doi.org/10.1126/science.1090165
3. Yan, J.; Kurisu, G.; Cramer, W.A. Intraprotein transfer of the quinone analogue inhibitor 2,5-dibromo-3-methyl-6-isopropyl-p-benzoquinone in the cytochrome *b6f* complex. *Proc. Natl. Acad. Sci. USA* **2006**, *103*, 69–74. https://doi.org/10.1073/pnas.0504909102
4. Yamashita, E.; Zhang, H.; Cramer, W.A. Structure of the cytochrome *b6f* complex: quinone analogue inhibitors as ligands of heme c_n. *J. Mol. Biol.* **2007**, *370*, 39–52. https://doi.org/10.1016/j.jmb.2007.04.011

5. Baniulis, D.; Yamashita, E.; Whitelegge, J.P.; Zatsman, A.I.; Hendrich, M.P.; Hasan, S.S.; Ryan, C.M.; Cramer, W.A. Structure-Function, Stability, and Chemical Modification of the Cyanobacterial Cytochrome *b6f* Complex from *Nostoc* sp. PCC 7120. *J. Biol. Chem.* **2009**, *284*, 9861–9869. https://doi.org/10.1074/jbc.M809196200
6. Hasan, S.S.; Yamashita, E.; Baniulis, D.; Cramer W.A. Quinone-dependent proton transfer pathways in the photosynthetic cytochrome b_6f complex. *Proc. Natl. Acad. Sci. USA* **2013**, *110*, 4297–4302. https://doi.org/10.1073/pnas.1222248110
7. Hasan, S.S.; Stofleth, J.T.; Yamashita, E.; Cramer, W.A. Lipid-induced conformational changes within the cytochrome *b6f* complex of oxygenic photosynthesis. *Biochemistry* **2013**, *52*, 2649–2654. https://doi.org/10.1021/bi301638h
8. Hasan, S.S.; Cramer, W.A. Internal Lipid Architecture of the Hetero-Oligomeric Cytochrome b6f Complex. *Structure* **2014**, *22*, 1008–1015. https://doi.org/10.1016/j.str.2014.05.004
9. Hasan, S.S.; Proctor, E.A.; Yamashita, E.; Dokholyan, N.V.; Cramer, W.A. Traffic within the Cytochrome *b6f* Lipoprotein Complex: Gating of the Quinone Portal. *Biophys. J.* **2014**, *107*, 1620–1628. https://doi.org/10.1016/j.bpj.2014.08.003
10. Malone, L.A.; Qian, P.; Mayneord, G.E.; Hitchcock, A.; Farmer, D.A.; Thompson, R.F.; Swainsbury, D.J.K.; Ranson, N.A.; Hunter, C.N.; Johnson, M.P. Cryo-EM structure of the spinach cytochrome *b6f* complex at 3.6 Å resolution. *Nature* **2019**, *575*, 535–539. https://doi.org/10.1038/s41586-019-1746-6
11. Proctor, M.S.; Malone, L.A.; Farmer, D.A.; Swainsbury, D.J.K.; Hawkings, F.R.; Pastorelli, F.; Emrich-Mills, T.Z.; Siebert, C.A.; Hunter, C.N.; Johnson, M.P.; Hitchcock, A. Cryo-EM structures of the *Synechocystis* sp. PCC 6803 cytochrome *b6f* complex with and without the regulatory PetP subunit. *Biochem. J.* **2022**, *479*, 1487–1503. https://doi.org/10.1042/BCJ20220124
12. Sarewicz, M.; Szwalec, M.; Pintscher, S.; Indyka, P.; Rawski, M.; Pietras, R.; Mielecki, B.; Koziej, Ł.; Jaciuk, M.; Glatt, S.; Osyczka, A. High-resolution cryo-EM structures of plant cytochrome b6f at work. *Sci. Adv.* **2023**, *9*, eadd9688. https://doi.org/10.1126/sciadv.add9688
13. Pintscher, S.; Pietras, R.; Mielecki, B.; Szwalec, M.; Wójcik-Augustyn, A.; Indyka, P.; Rawski, M.; Koziej, Ł.; Jaciuk, M.; Ważny, G.; Glatt, S.; Osyczka, A. Molecular basis of plastoquinone reduction in plant cytochrome b_6f. *Nat. Plants* **2024**, *10*, 1814–1825. https://doi.org/10.1038/s41477-024-01804-x
14. Hasan, S.S.; Yamashita, E.; Ryan, C.M.; Whitelegge, J.P.; Cramer, W.A. Conservation of lipid functions in cytochrome bc complexes. J. Mol. Boil. **2011**, *414*, 145–162. https://doi.org/10.1016/j.jmb.2011.09.023
15. Di Trani, J. M.; Liu, Z.; Whitesell, L.; Brzezinski, P.; Cowen, L.E.; Rubinstein, J.L. Rieske head domain dynamics and indazole-derivative inhibition of Candida albicans complex III. *Structure* **2022**, *30*, 129–138.e4. https://doi.org/10.1016/j.str.2021.08.006
16. Vladkova R. Chlorophyll *a* is the crucial redox sensor and transmembrane signal transmitter in the cytochrome b_6f complex. Components and mechanisms of state transitions from the hydrophobic mismatch viewpoint. *J. Biomol. Struct. Dyn.* **2016**, *34*, 824–854. Epub 2015 Jul 8. http://doi.org/10.1080/07391102.2015.1056551
17. Lomize, M.A.; Pogozheva, I.D.; Joo, H.; Mosberg, H.I.; Lomize, A.L. OPM database and PPM web server: resources for positioning of proteins in membranes. *Nucleic Acids Res.* **2012**, *40(Database issue)*, D370–D376. https://doi.org/10.1093/nar/gkr703
18. Solmaz, S.R.; Hunte, C. Structure of complex III with bound cytochrome *c* in reduced state and definition of a minimal core interface for electron transfer. *J. Biol. Chem.* **2008**, *283*, 17542–17549. https://doi.org/10.1074/jbc.M710126200
19. Palsdottir, H.; Hunte, C. Lipids in membrane protein structures. *Biochim. Biophys. Acta* **2004**, *1666*, 2–18. https://doi.org/10.1016/j.bbamem.2004.06.012
20. Wieferig, J.P.; Kühlbrandt, W. Analysis of the conformational heterogeneity of the Rieske iron-sulfur protein in complex III2 by cryo-EM. *IUCrJ* **2023,** *10*, 27–37. https://doi.org/10.1107/S2052252522010570
21. Klusch, N.; Dreimann, M.; Senkler, J.; Rugen, N.; Kühlbrandt, W.; Braun, H.P. Cryo-EM structure of the respiratory I + III2 supercomplex from Arabidopsis thaliana at 2 Å resolution. *Nat. Plants*, **2023**, *9*, 142–156. https://doi.org/10.1038/s41477-022-01308-6
22. Zheng, W.; Chai, P.; Zhu, J.; Zhang, K. High-resolution in situ structures of mammalian respiratory supercomplexes. *Nature*, **2024**, *631*, 232–239. https://doi.org/10.1038/s41586-024-07488-9

23. Palsdottir, H.; Lojero, C.G.; Trumpower, B.L.; Hunte, C. Structure of the yeast cytochrome bc1 complex with a hydroxyquinone anion Qo site inhibitor bound. *J. Biol. Chem.* **2003**, *278*, 31303–31311. https://doi.org/10.1074/jbc.M302195200
24. Birth, D.; Kao, W.C.; Hunte, C. Structural analysis of atovaquone-inhibited cytochrome bc1 complex reveals the molecular basis of antimalarial drug action. *Nat. Commun.* **2014**, *5*, 4029. https://doi.org/10.1038/ncomms5029
25. Esser, L.; Quinn, B.; Li, Y.F.; Zhang, M.; Elberry, M.; Yu, L.; Yu, C.A.; Xia, D. Crystallographic studies of quinol oxidation site inhibitors: a modified classification of inhibitors for the cytochrome bc(1) complex. *J. Mol. Biol.* **2004**, *341*, 281–302. https://doi.org/10.1016/j.jmb.2004.05.065
26. Huang, L.S.; Cobessi, D.; Tung, E.Y.; Berry, E.A. Binding of the respiratory chain inhibitor antimycin to the mitochondrial bc1 complex: a new crystal structure reveals an altered intramolecular hydrogen-bonding pattern. *J. Mol. Biol.* **2005**, *351*, 573–597. https://doi.org/10.1016/j.jmb.2005.05.053
27. Esser, L.; Elberry, M.; Zhou, F.; Yu, C.A.; Yu, L.; Xia, D. Inhibitor-complexed structures of the cytochrome bc1 from the photosynthetic bacterium Rhodobacter sphaeroides. *J. Biol. Chem.* **2008**, *283*, 2846–2857. https://doi.org/10.1074/jbc.M708608200
28. Esser, L.; Gong, X.; Yang, S.; Yu, L.; Yu, C.A.; Xia, D. Surface-modulated motion switch: capture and release of iron-sulfur protein in the cytochrome bc1 complex. *Proc. Natl. Acad. Sci. USA* **2006**, *103*, 13045–13050. https://doi.org/10.1073/pnas.0601149103
29. Gao, X.; Wen, X.; Yu, C.; Esser, L.; Tsao, S.; Quinn, B.; Zhang, L.; Yu, L.; Xia, D. The crystal structure of mitochondrial cytochrome bc1 in complex with famoxadone: the role of aromatic-aromatic interaction in inhibition. *Biochemistry* **2002**, *41*, 11692–11702. https://doi.org/10.1021/bi026252p
30. Berry, E.A.; Huang, L.S. Conformationally linked interaction in the cytochrome bc_1 complex between inhibitors of the Q_o site and the Rieske iron–sulfur protein. *Biochim. Biophys. Acta – Bioenerg.* **2011**, *1807*, 1349–1363. https://doi.org/10.1016/j.bbabio.2011.04.005
31. Hao, G.F.; Wang, F.; Li, H.; Zhu, X.L.; Yang, W.C.; Huang, L.S.; Wu, J.W.; Berry, E.A.; Yang, G.F. Computational discovery of picomolar Q(o) site inhibitors of cytochrome bc1 complex. *J. Am. Chem. Soc.* **2012**, *134*, 11168–11176. https://doi.org/10.1021/ja3001908
32. Gao, X.; Wen, X.; Esser, L.; Quinn, B.; Yu, L.; Yu, C.A.; Xia, D. Structural basis for the quinone reduction in the bc1 complex: a comparative analysis of crystal structures of mitochondrial cytochrome bc1 with bound substrate and inhibitors at the Qi site. *Biochemistry*, **2003**, *42*, 9067–9080. https://doi.org/10.1021/bi0341814
33. Iwata, S.; Lee, J.W.; Okada, K.; Lee, J.K.; Iwata, M.; Rasmussen, B.; Link, T.A.; Ramaswamy, S.; Jap, B.K. Complete structure of the 11-subunit bovine mitochondrial cytochrome bc1 complex. *Science* **1998,** *281*, 64–71. https://doi.org/10.1126/science.281.5373.64
34. Zhang, Z.; Huang, L.; Shulmeister, V.M.; Chi, Y.I.; Kim, K.K.; Hung, L.W.; Crofts, A.R.; Berry, E.A.; Kim, S.H. Electron transfer by domain movement in cytochrome bc1. *Nature* **1998**, *392*, 677–684. https://doi.org/10.1038/33612
35. Esser, L.; Zhou, F.; Zeher, A.; Wu, W.; Huang, R.; Yu, C.A.; Lane, K.D.; Wellems, T.E.; Xia, D. Structure of complex III with bound antimalarial agent CK-2-68 provides insights into selective inhibition of Plasmodium cytochrome bc1 complexes. *J. Biol. Chem.* **2023**, 299, 104860. https://doi.org/10.1016/j.jbc.2023.104860
36. Swainsbury, D.J.K.; Hawkings, F.R.; Martin, E.C.; Musiał, S.; Salisbury, J.H.; Jackson, P.J.; Farmer, D.A.; Johnson, M.P.; Siebert, C.A.; Hitchcock, A.; Hunter, C.N. Cryo-EM structure of the four-subunit Rhodobacter sphaeroides cytochrome bc1 complex in styrene maleic acid nanodiscs. *Proc. Natl. Acad. Sci. USA* **2023**, *120*, e2217922120. https://doi.org/10.1073/pnas.2217922120
37. Zhou, F.; Xia, D.; Esser, L. Conformation switch of Rieske isp subunit is revealed by the crystal structure of bacterial cytochrome bc1 in complex with azoxystrobin. To be published (last visited 17 Feb 2025). https://www.rcsb.org/structure/7TLJ
38. Di Trani, J. M.; Liu, Z.; Whitesell, L.; Brzezinski, P.; Cowen, L.E.; Rubinstein, J.L. Rieske head domain dynamics and indazole-derivative inhibition of Candida albicans complex III. *Structure* **2022**, *30*, 129–138.e4. https://doi.org/10.1016/j.str.2021.08.006
39. Zhu, G.; Zeng, H.; Zhang, S.; Juli, J.; Pang, X.; Hoffmann, J.; Zhang, Y.; Morgner, N.; Zhu, Y.; Peng, G.; Michel, H.; Sun, F. A 3.3 Å-Resolution Structure of Hyperthermophilic Respiratory Complex III Reveals the Mechanism of Its Thermal Stability. *Angew. Chem. Int. Ed. Engl.* **2020**, *59*, 343–351. https://doi.org/10.1002/anie.201911554
40. Solmaz, S.R.; Hunte, C. Structure of complex III with bound cytochrome c in reduced state and definition of a minimal core interface for electron transfer. *J. Biol. Chem.* **2008**, *283*, 17542–17549. https://doi.org/10.1074/jbc.M710126200
