# Supplementary material for: X-Ray Crystal and Cryo-Electron Microscopy Structure Analysis Unravels How the Unique Thylakoid Lipid Composition Is Utilized by Cytochrome b6f for Driving Reversible Proteins’ Reorganization During State Transitions
Source: Membranes (Basel). 2025 May 8;15(5):143. doi: 10.3390/membranes15050143 (PMC12112970; doi:10.3390/membranes15050143)
Supplement: Supplementary file 1 [file membranes-15-00143-s001.zip › 2-Suppl_Images-1.pdf]

Supplementary Material for

X-ray crystal and cryo-EM structure analysis unravel how the unique thylakoid lipid composition is utilized by cytochrome b_6f for driving reversible proteins' reorganization during state transitions

Radka Vladkova (rvladkova@bio21.bas.bg)

Supplementary Package of Images – Part 1

(Related to Figure 2, Figure 3 and to the assessment of the lipid binding sites in the cytochrome b_6f crystal structure 4OGQ)

Cyt*b6f* dimer p-side view

Axes of symmetry

Monomer 1 without
the protein subunits

Monomer 2 with
the subunits:

cyt*b6* (A-D)

subunit IV (E-G)

cyt*f*

Rieske (ISP)

PetG

PetL

PetM

PetN

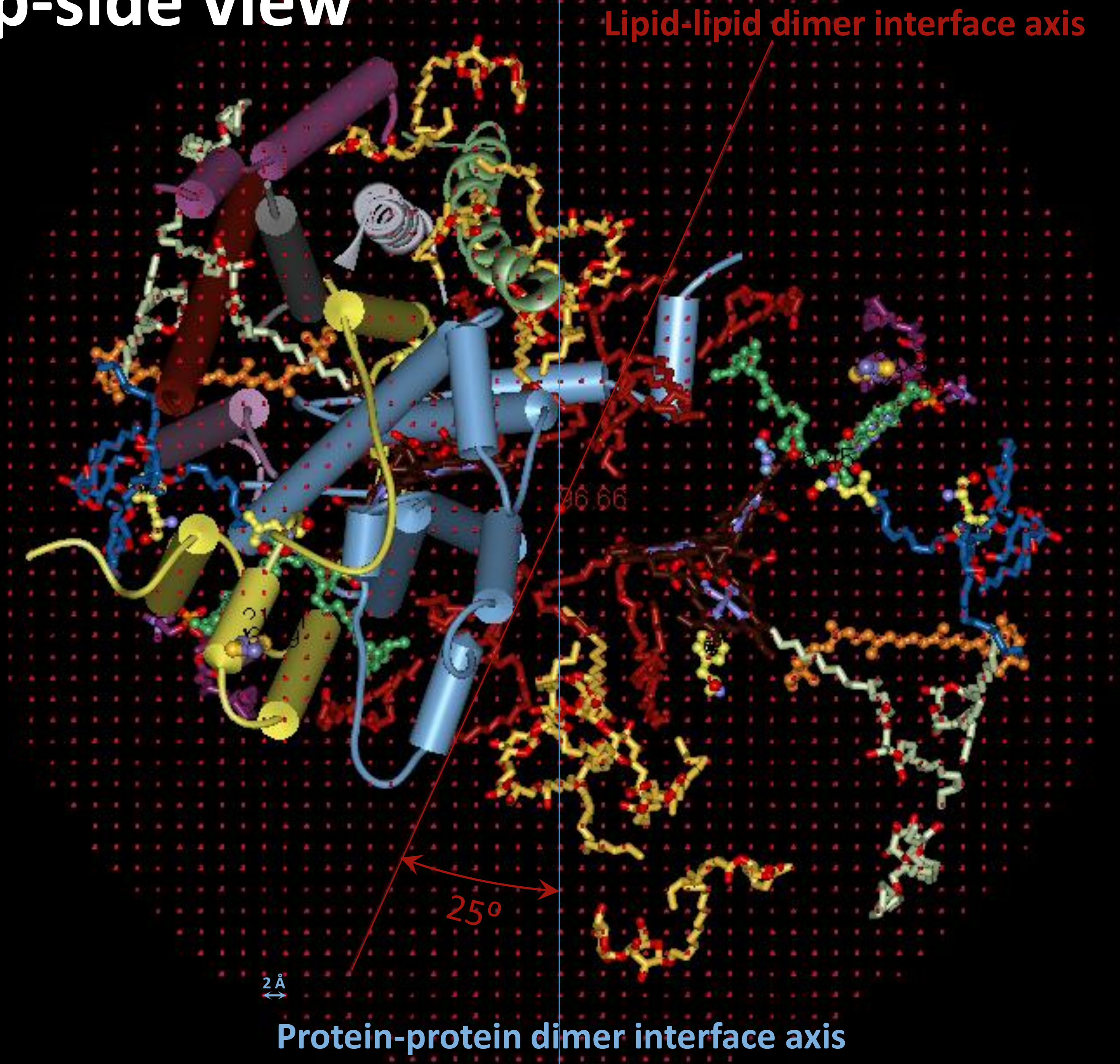

Chl*a*

β-Car

hemes

n-L1

n-, p-L2

n-, p-L3

n-, p-L4

n-, p-L5

Cyt*b6f* dimer n-side view

Chlorophyll *a* orientation

Monomer 1 without the protein subunits

Monomer 2 with the subunits:
cyt*b6* (A-D)
subunit IV (E-G)
cyt*f*
Rieske (ISP)
PetG
PetL
PetM
PetN

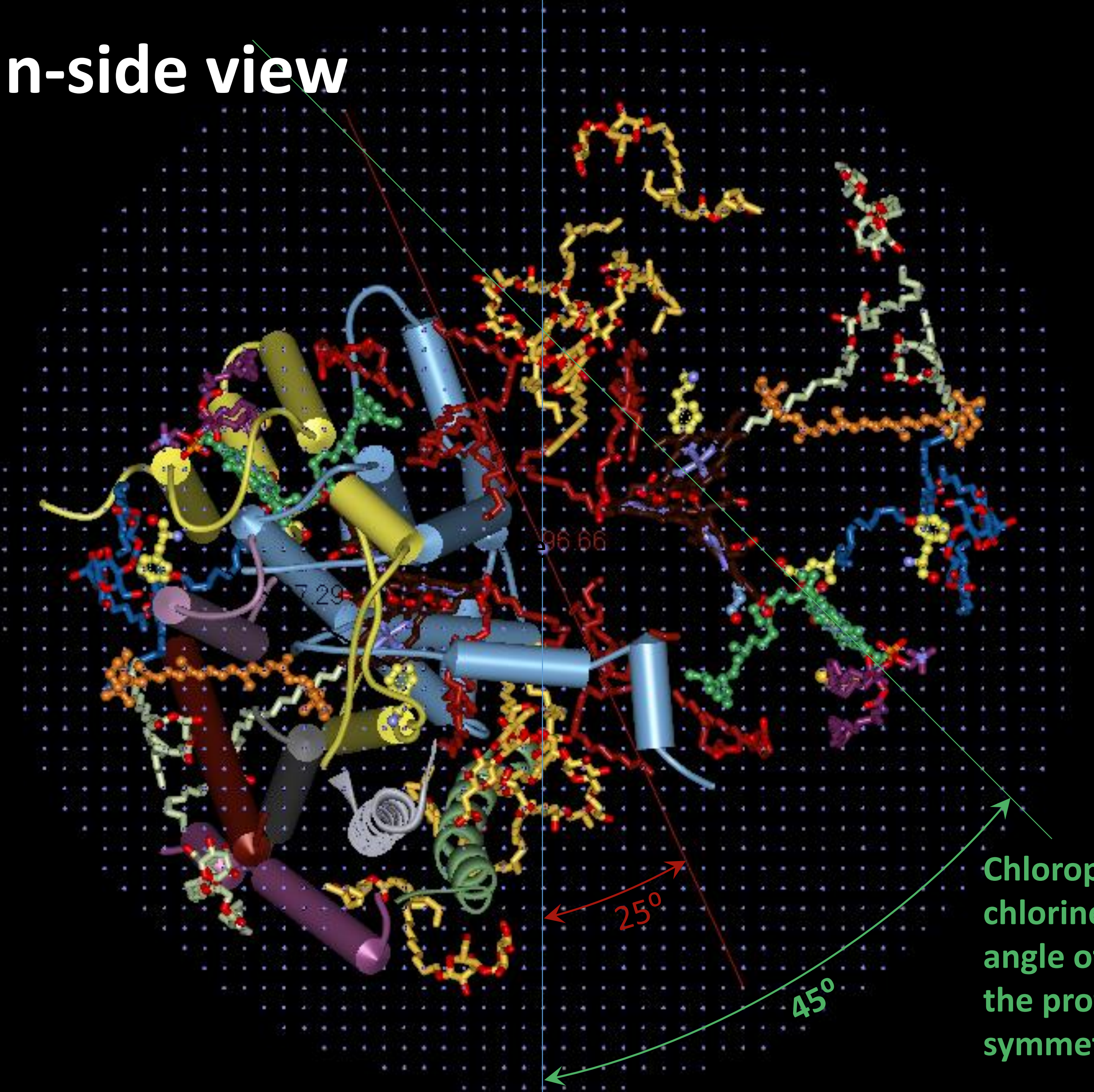

Chl*a*
β-Car
hemes
n-L1
n-, p-L2
n-, p-L3
n-, p-L4
n-, p-L5

Chlorophyll *a* chlorine ring is at an angle of 45° with the protein symmetry axis

Cyt*b6f* dimer n-side view

Monomer 1 + surface
Monomer 2 – surface

Cyt*b6f* subunits:
cyt*b6* (A-D)
subunit IV (E-G)
cyt*f*
Rieske (ISP)
PetG
PetL
PetM
PetN

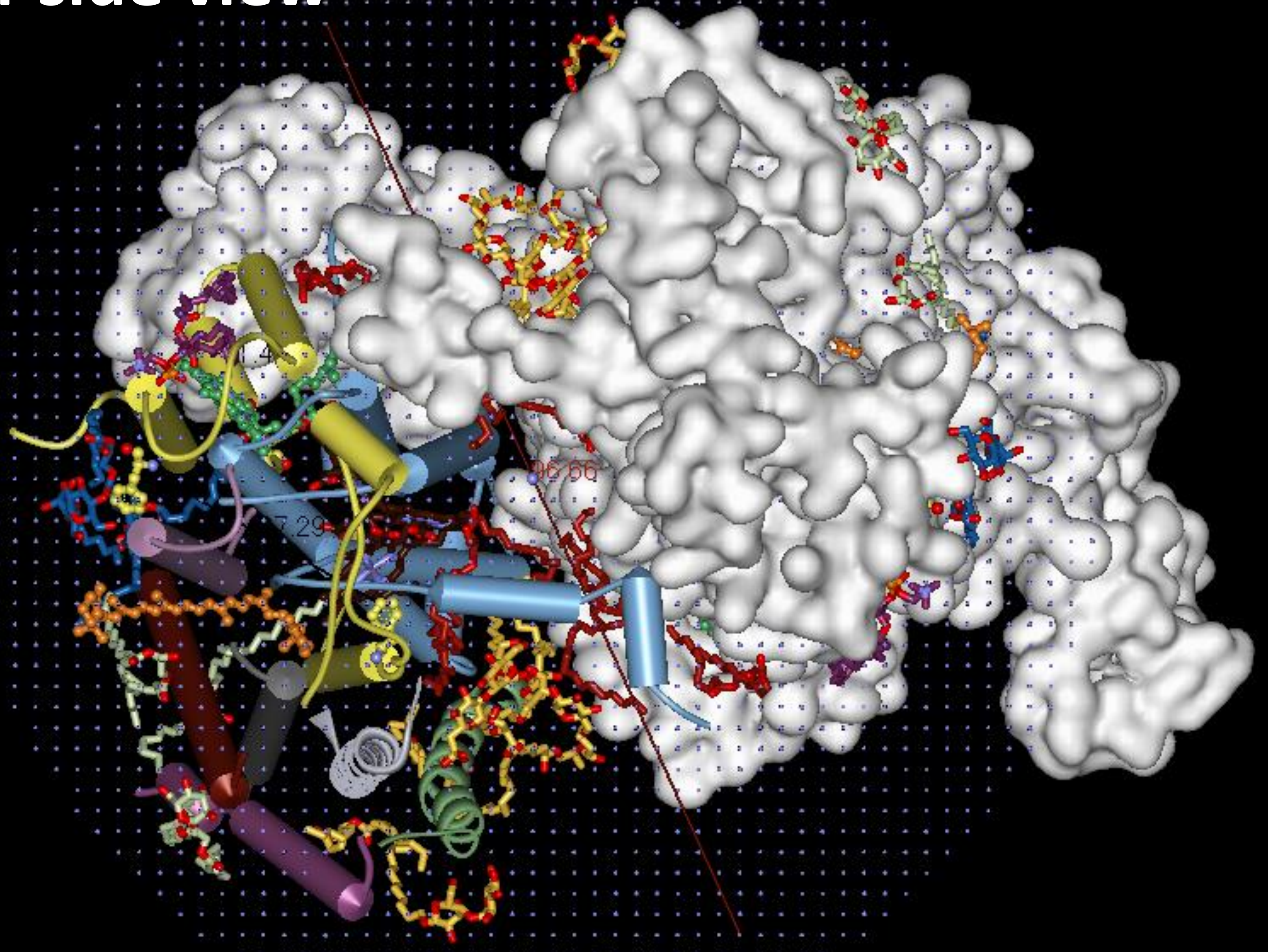

Chl*a*
β-Car
hemes
n-L1
n-,p-L2
n-, p-L3
n-, p-L4
n-, p-L5

Cyt*b6f* dimer n-side view

Monomer 1 + surface

Monomer 2 + surface

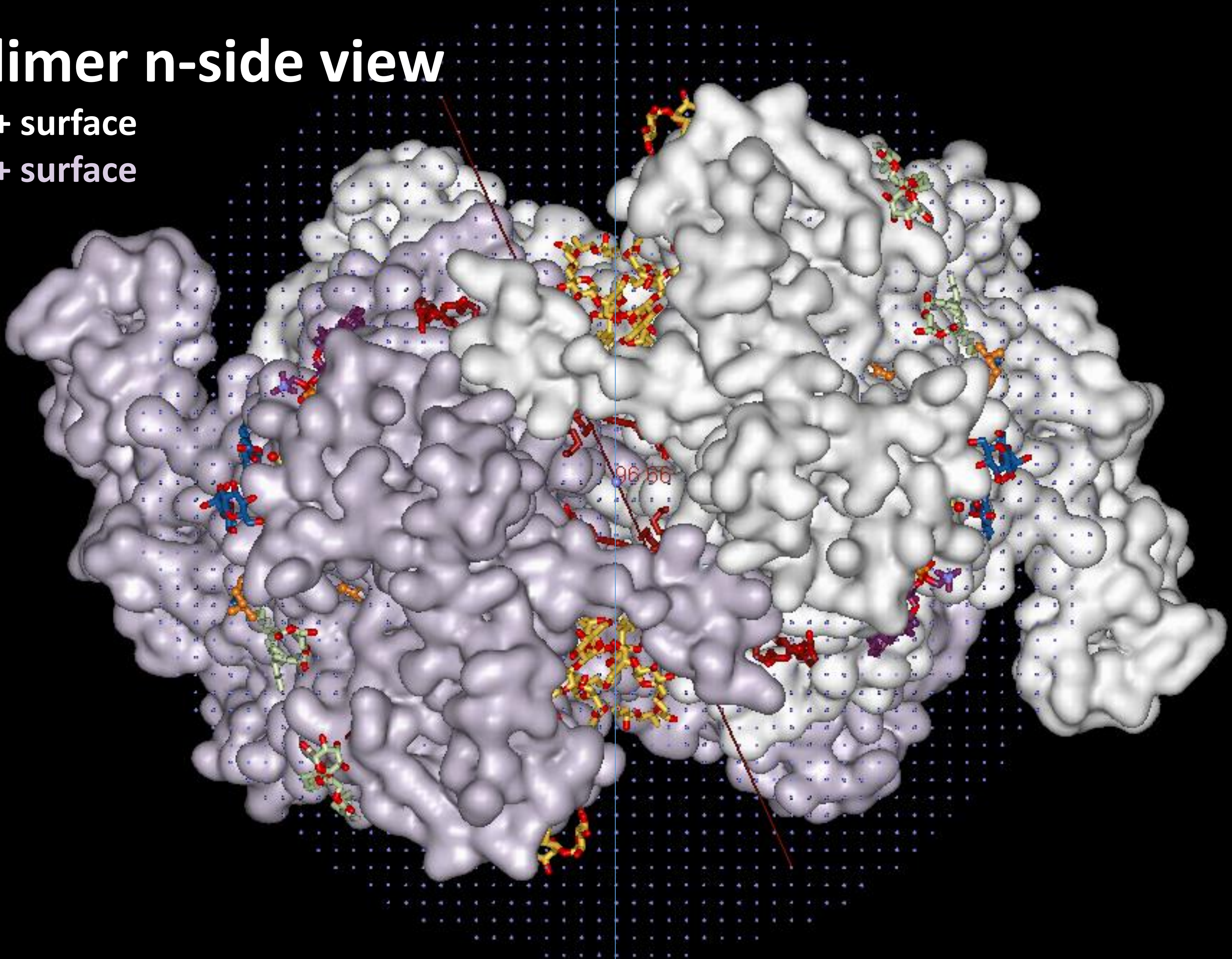

n-L1

n-, p-L2

n-, p-L3

n-, p-L4

n-, p-L5

Cyt*b6f* dimer p-side view

Monomer 1 + surface
Monomer 2 + surface

Visible are:
n-L4(1) – SQDG
n-L4(2) – UMQ201
p-L4(1) – 7PH203
p-L4(4) – 7PH303

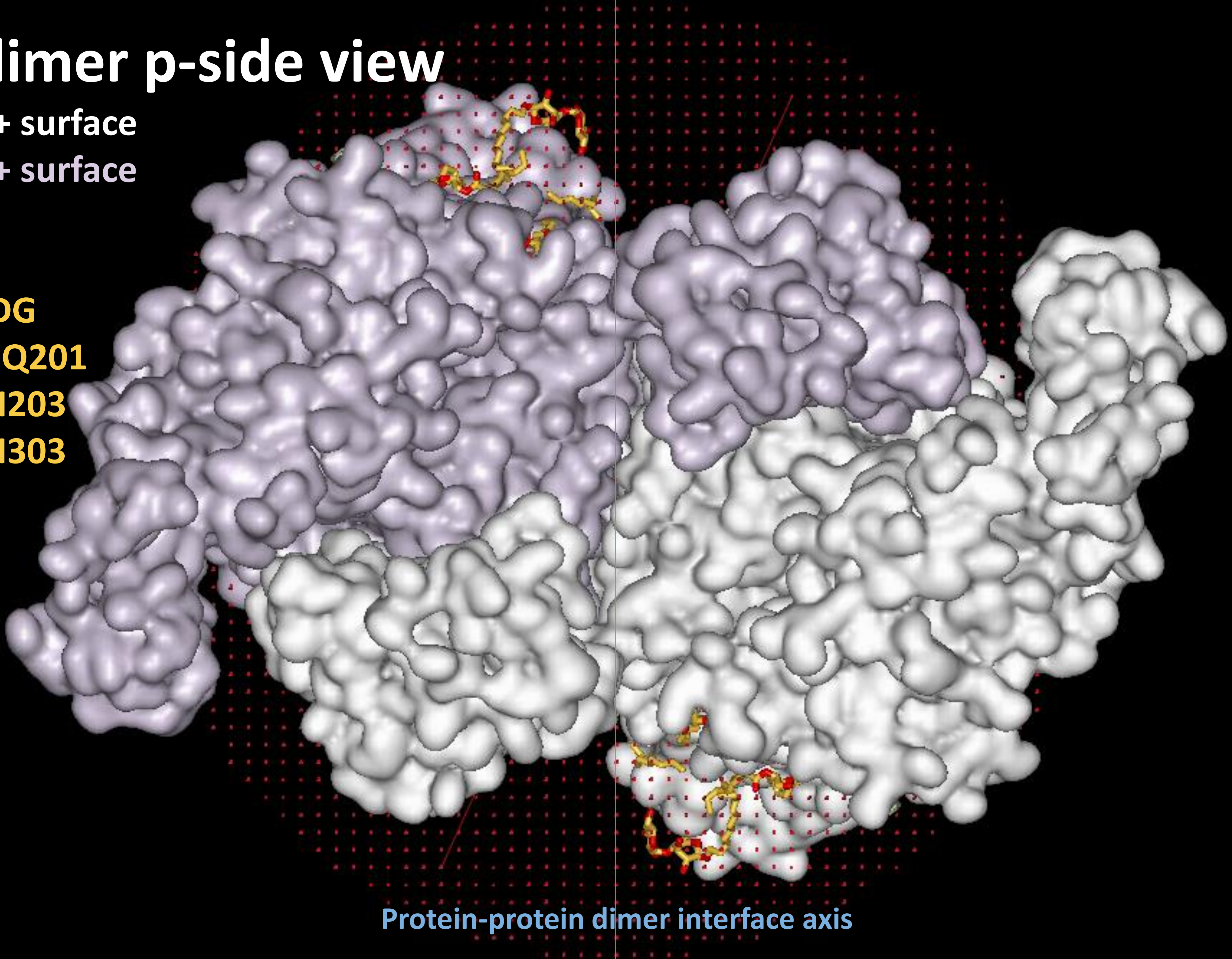

Cyt*b6f* dimer front view

Spin 0º

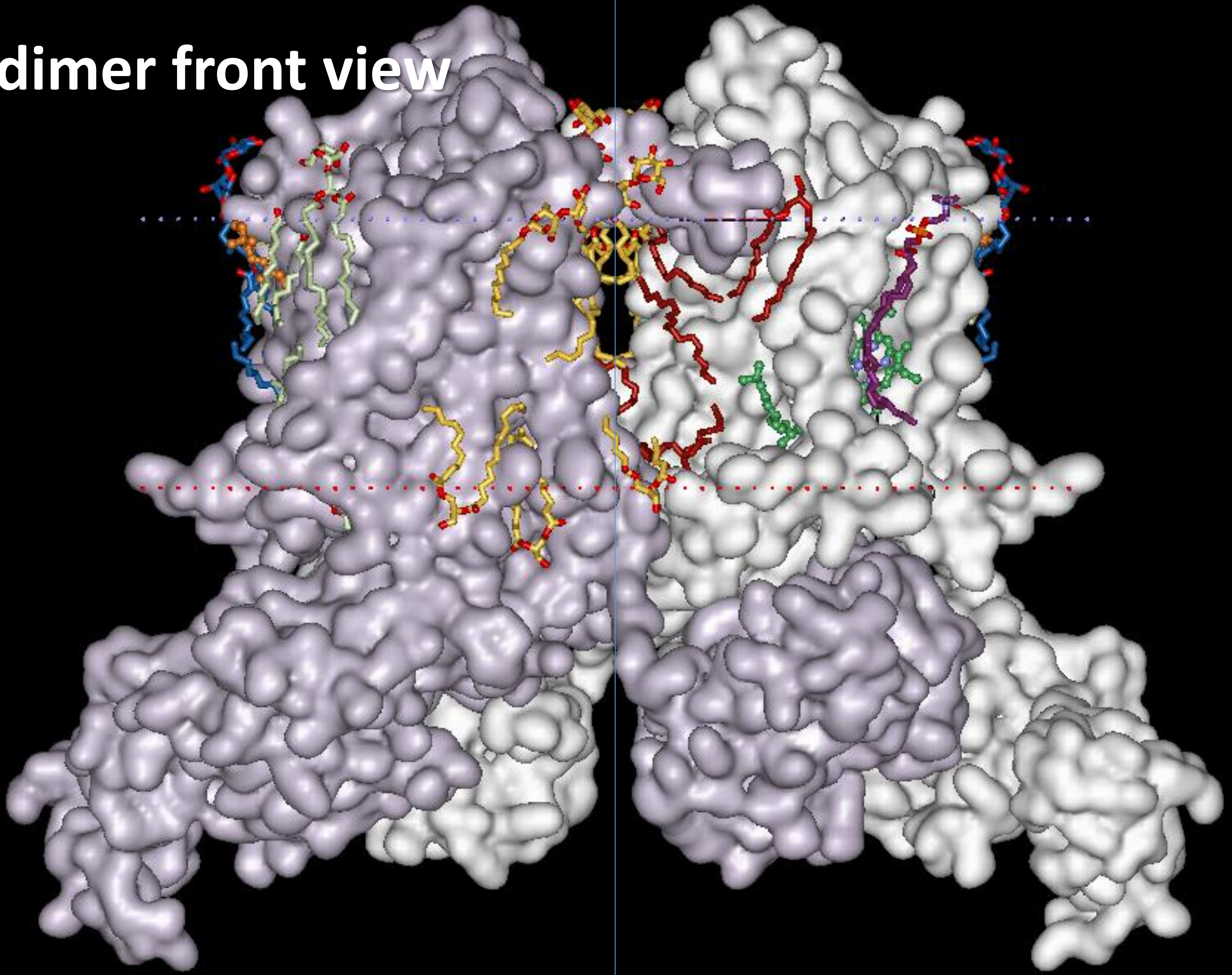

Cyt*b6f*:
Monomer 1
Monomer 2

Chl*a*
β-Car
hemes
n-L1
n-, p-L2
n-, p-L3
n-, p-L4
n-, p-L5

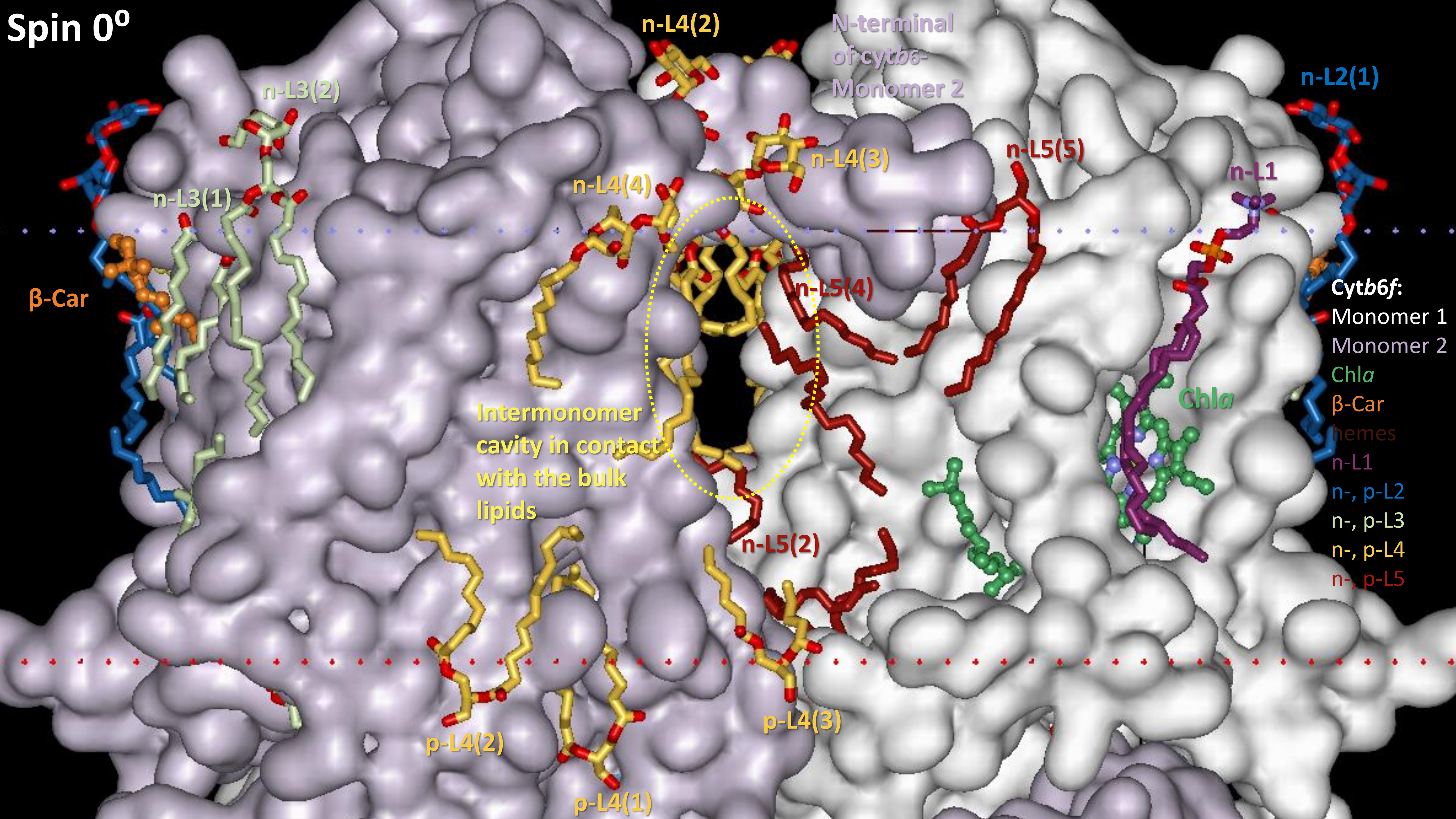
Spin 0°
n-L4(2)
N-terminal of cytb6- Monomer 2
n-L3(2)
n-L2(1)
n-L4(3)
n-L5(5)
n-L1
n-L4(4)
n-L3(1)
β-Car
n-L5(4)
Cytb6f:
Monomer 1
Monomer 2
Chla
β-Car
hemes
n-L1
n-, p-L2
n-, p-L3
n-, p-L4
n-, p-L5
Chla
Intermonomer cavity in contact with the bulk lipids
n-L5(2)
p-L4(3)
p-L4(2)
p-L4(1)

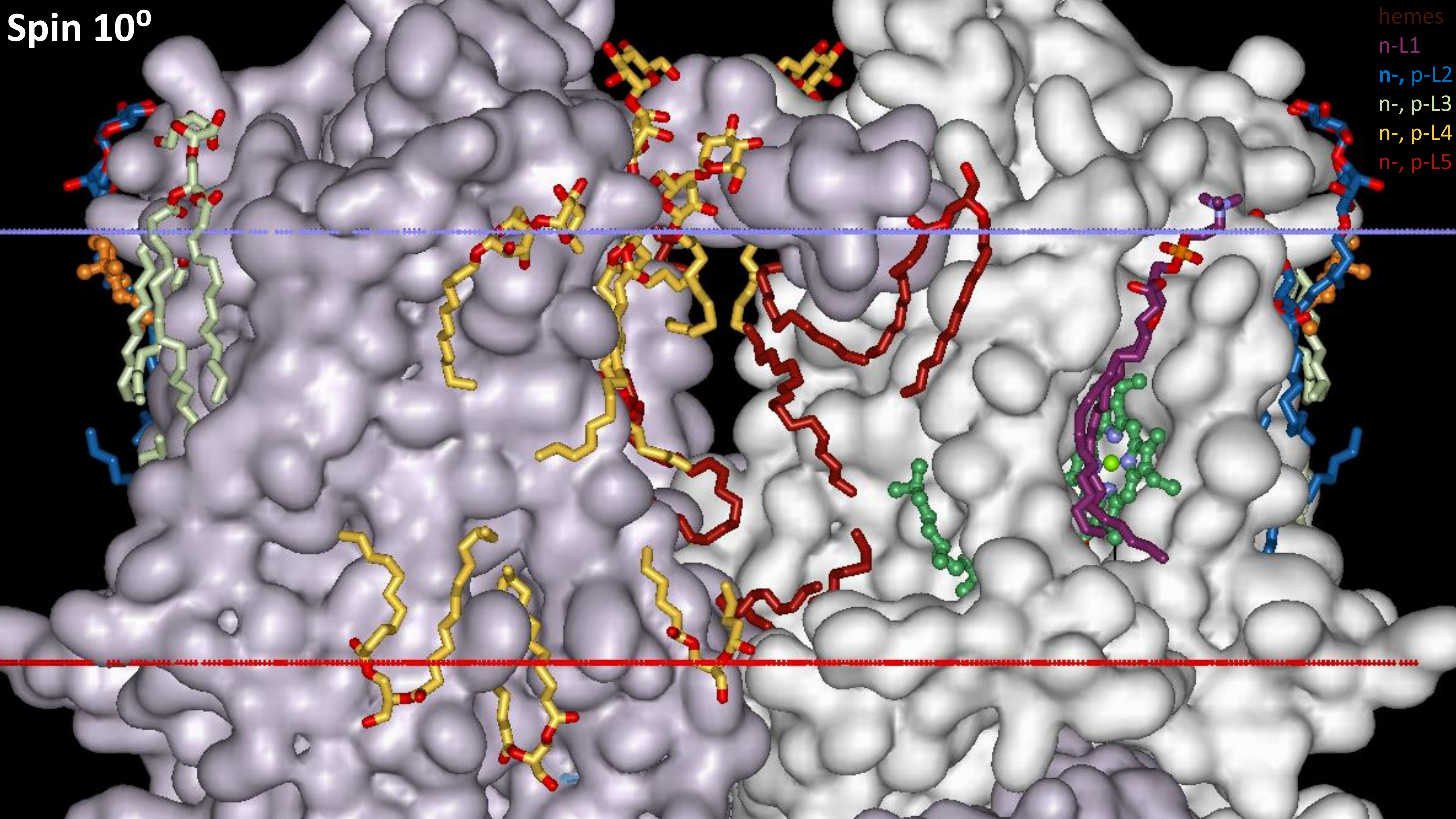
Spin 10º
hemes
n-L1
n-, p-L2
n-, p-L3
n-, p-L4
n-, p-L5

Spin 20º

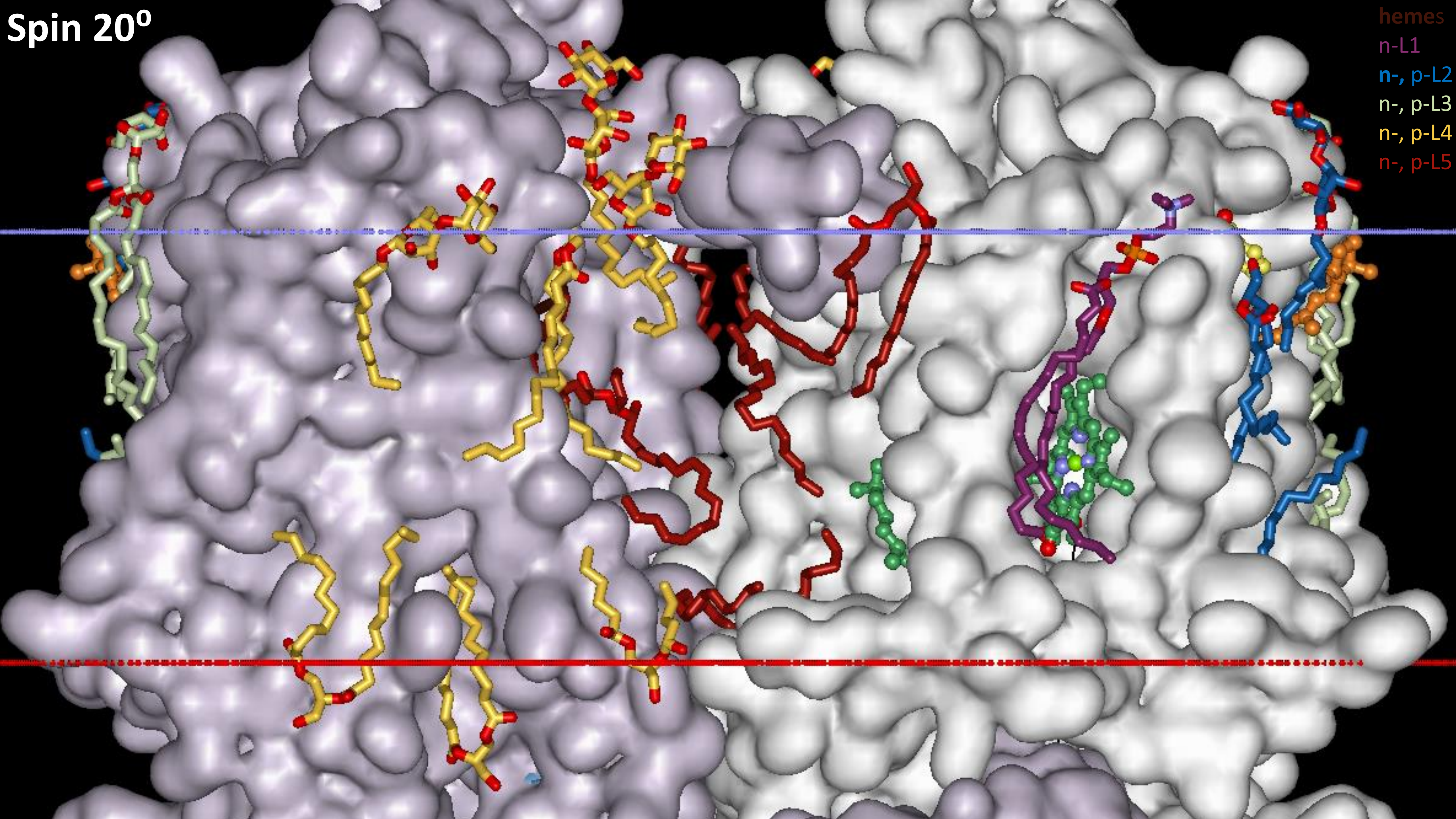

Spin 30º

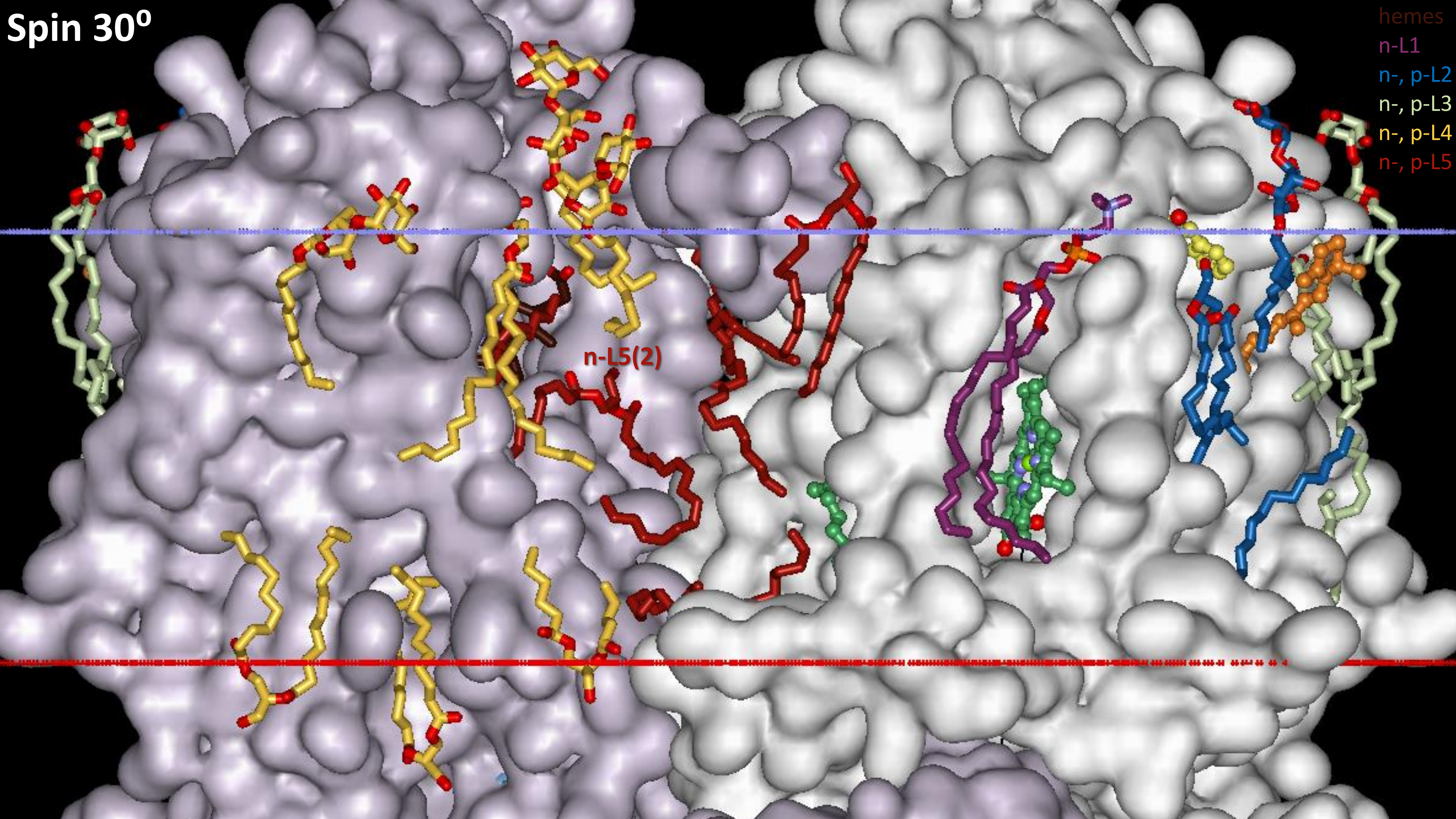

Spin 40º

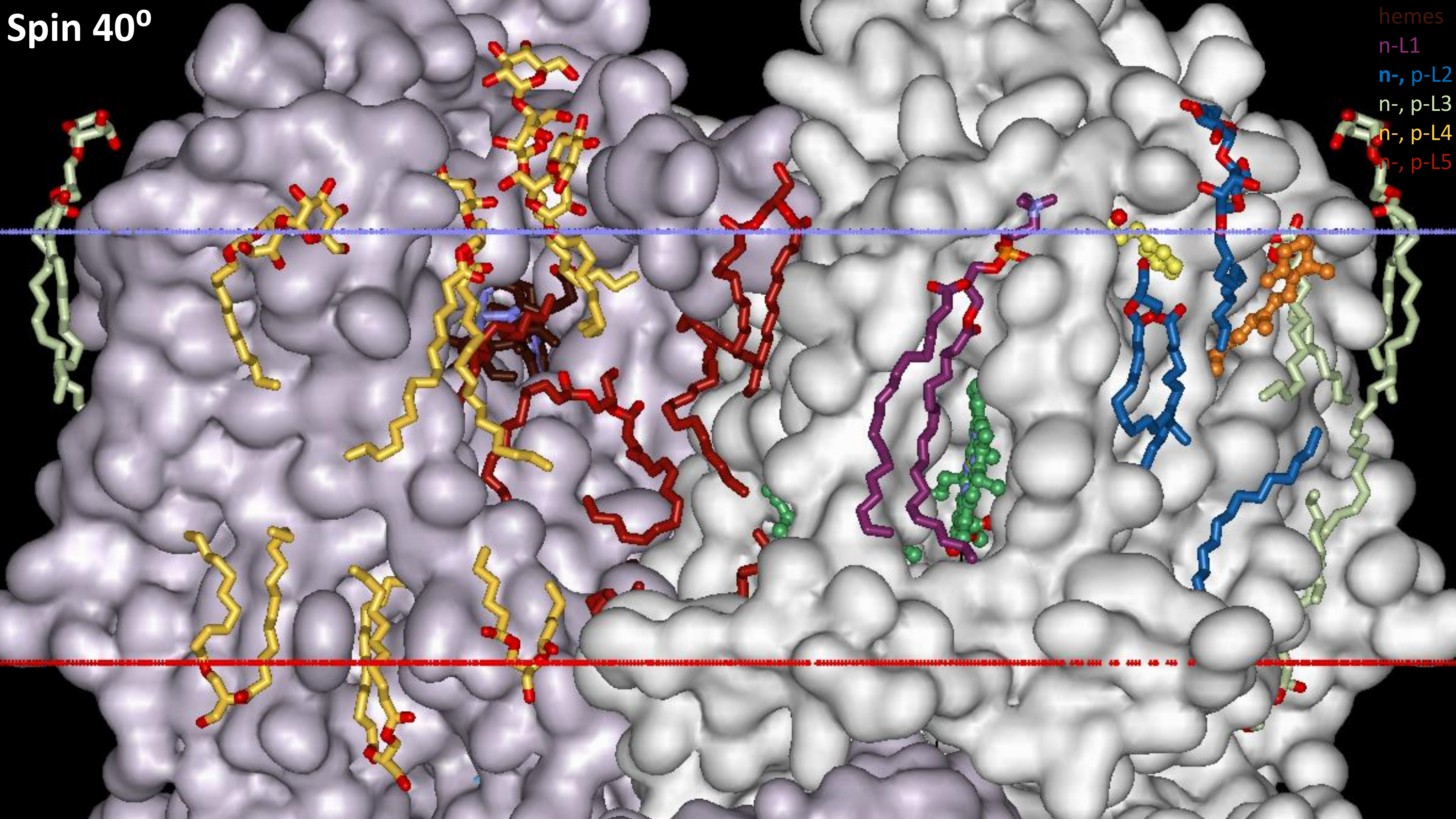

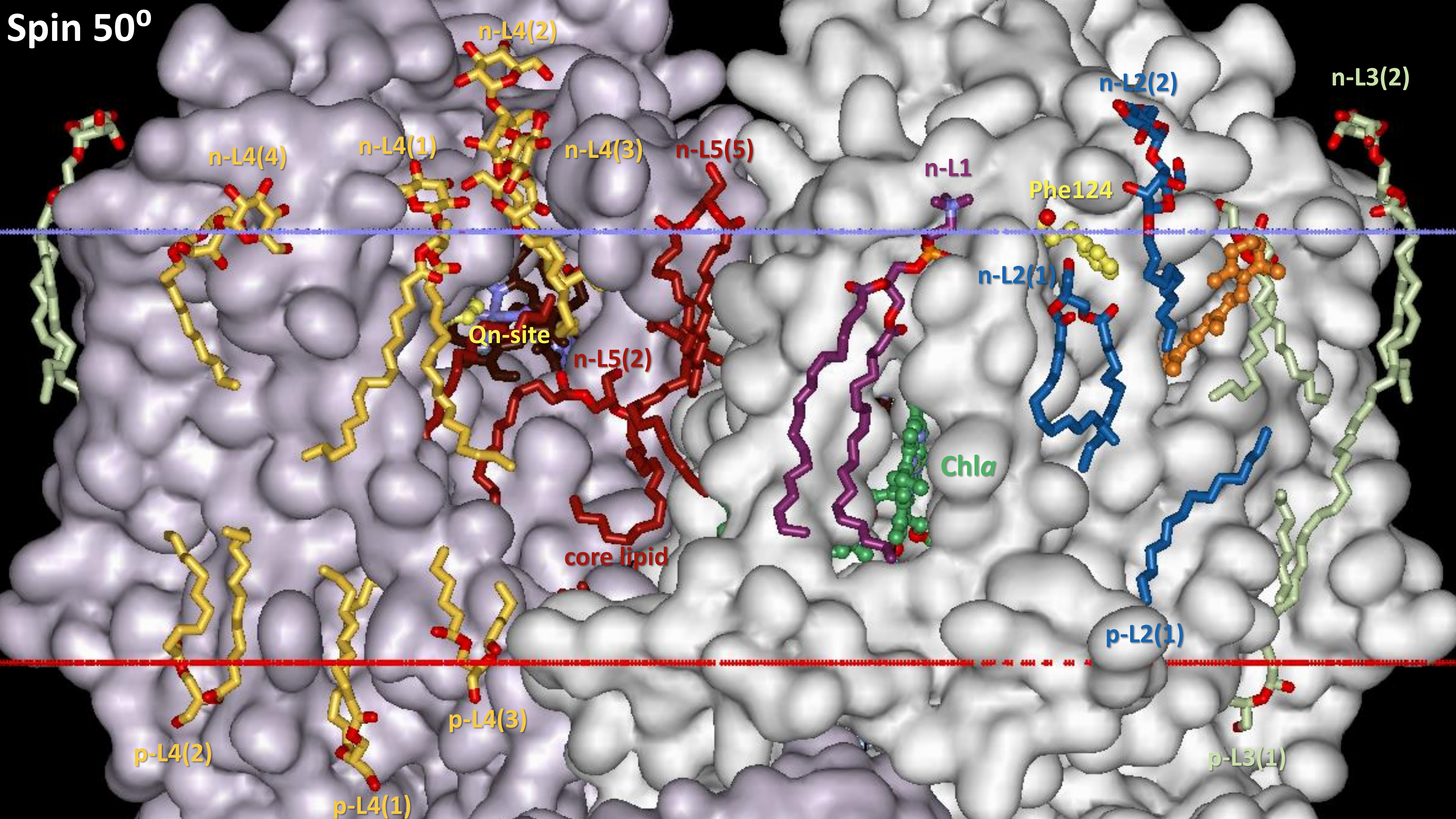
Spin 50°
n-L4(2)
n-L2(2)
n-L3(2)
n-L4(4)
n-L4(1)
n-L4(3)
n-L5(5)
n-L1
Phe124
n-L2(1)
Qn-site
n-L5(2)
Chl*a*
core lipid
p-L2(1)
p-L4(3)
p-L4(2)
p-L3(1)
p-L4(1)

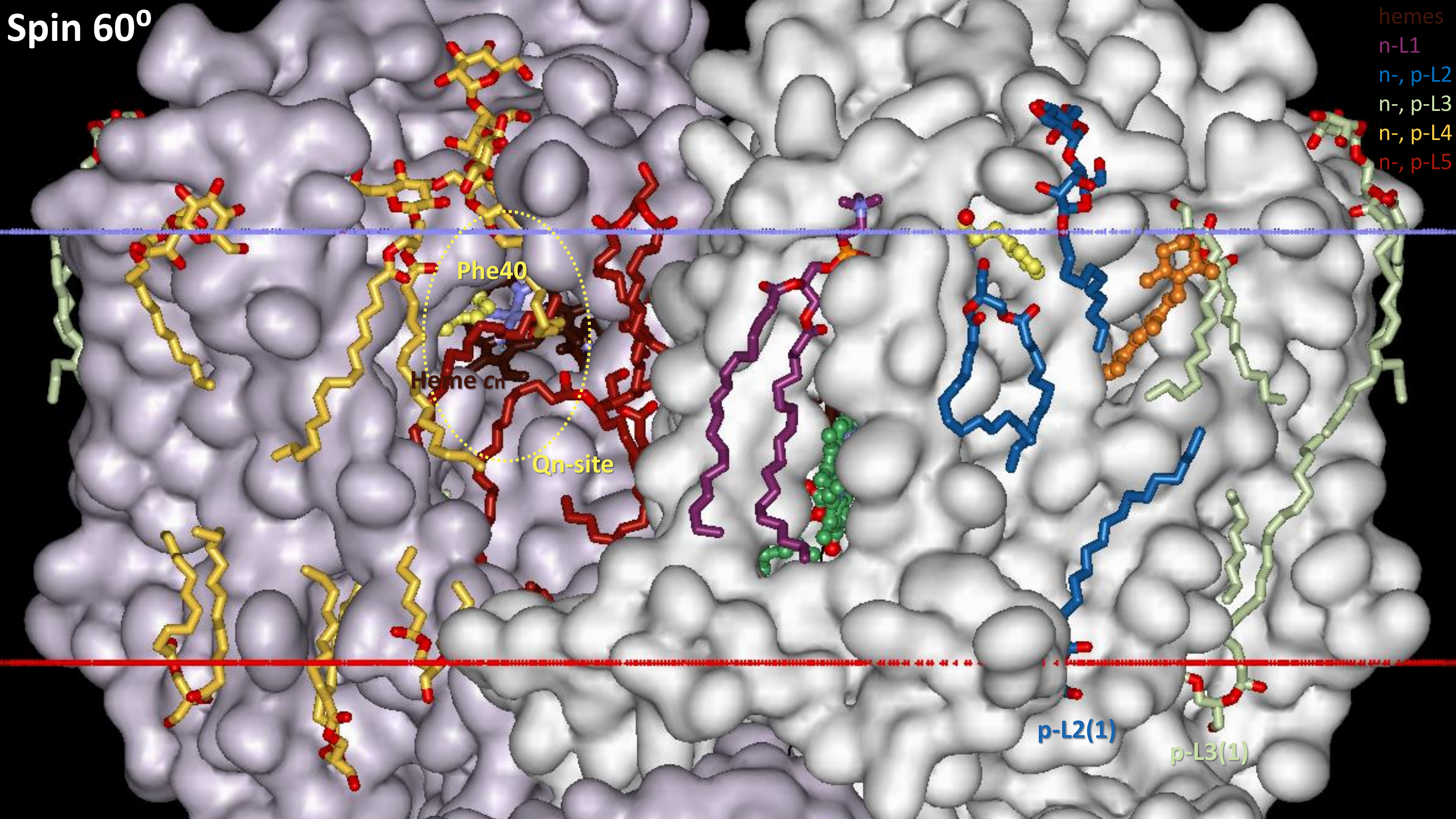

Spin 60º
hemes
n-L1
n-, p-L2
n-, p-L3
n-, p-L4
n-, p-L5
Phe40
Heme cn
Qn-site
p-L2(1)
p-L3(1)

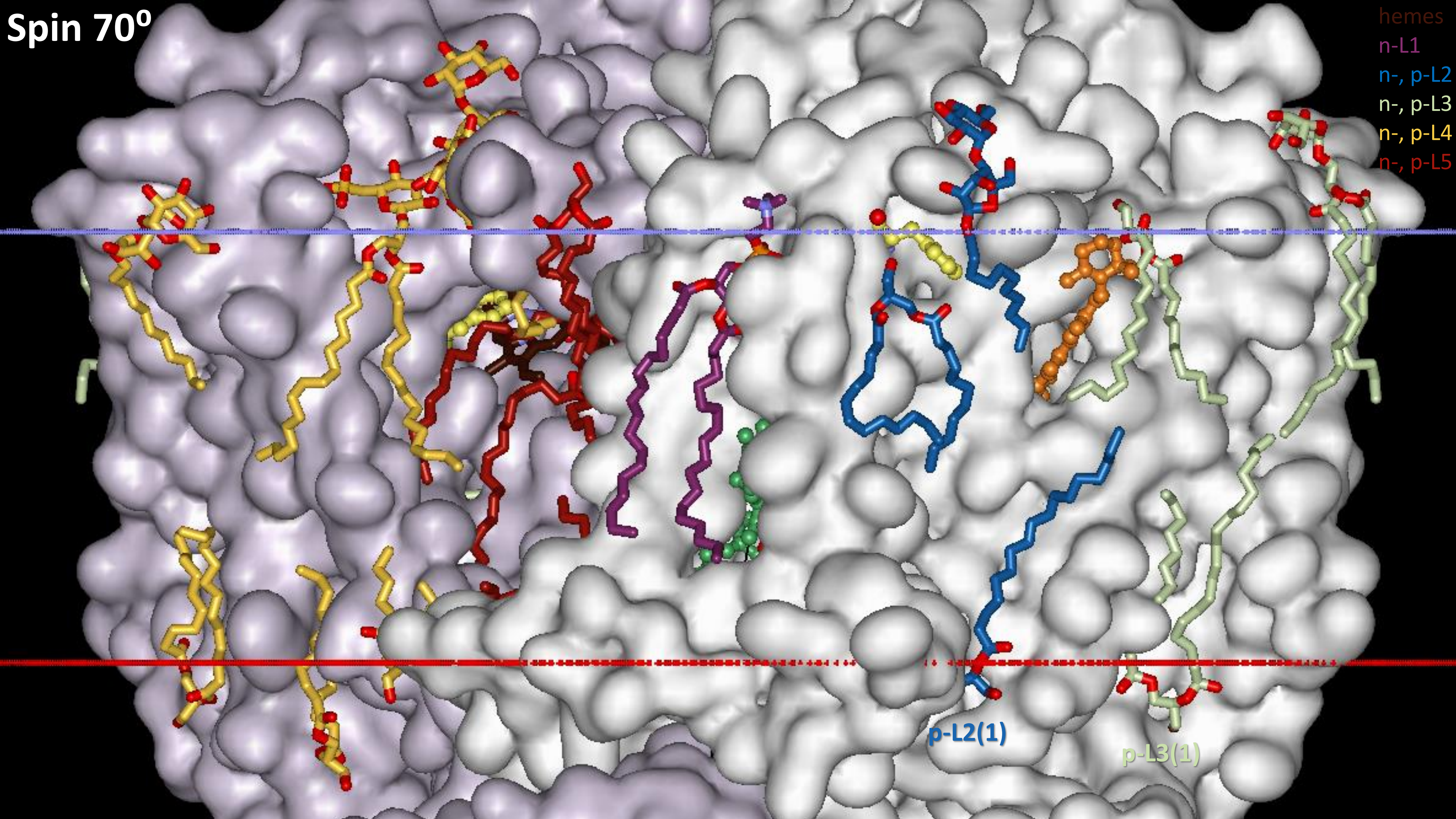
Spin 70º
hemes
n-L1
n-, p-L2
n-, p-L3
n-, p-L4
n-, p-L5
p-L2(1)
p-L3(1)

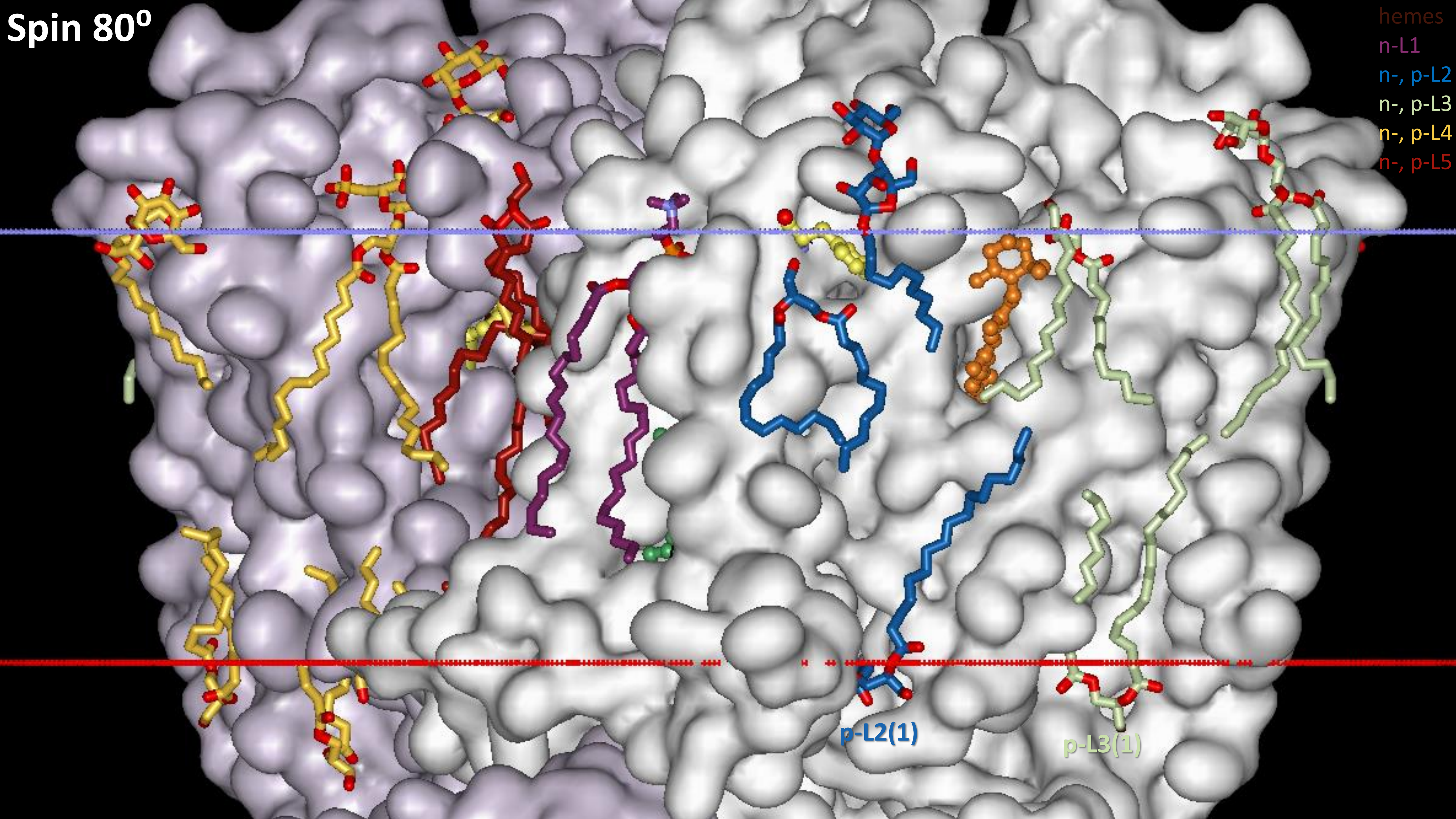
Spin 80º
hemes
n-L1
n-, p-L2
n-, p-L3
n-, p-L4
n-, p-L5
p-L2(1)
p-L3(1)

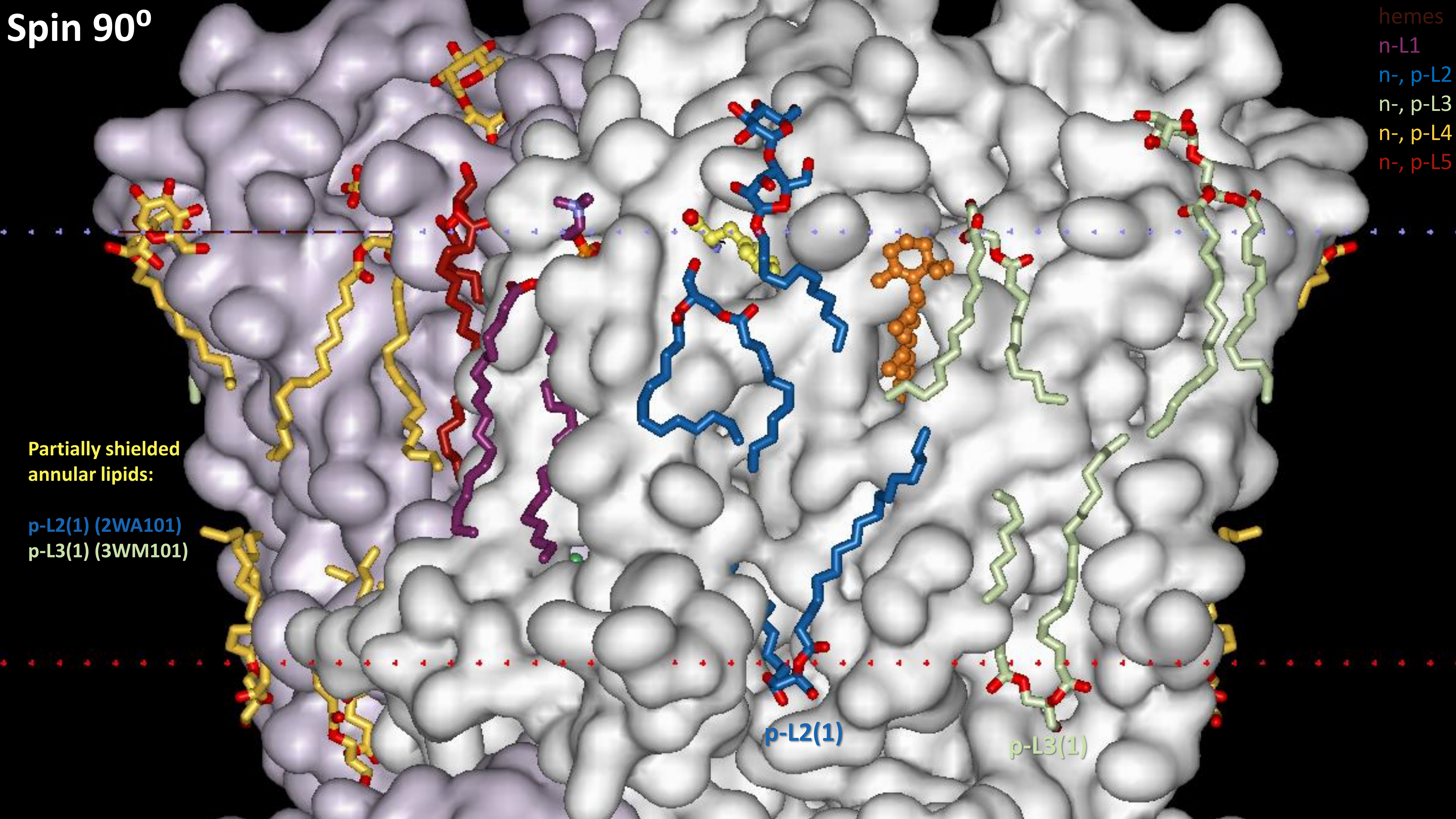
Spin 90º
hemes
n-L1
n-, p-L2
n-, p-L3
n-, p-L4
n-, p-L5
Partially shielded
annular lipids:
p-L2(1) (2WA101)
p-L3(1) (3WM101)
p-L2(1)
p-L3(1)

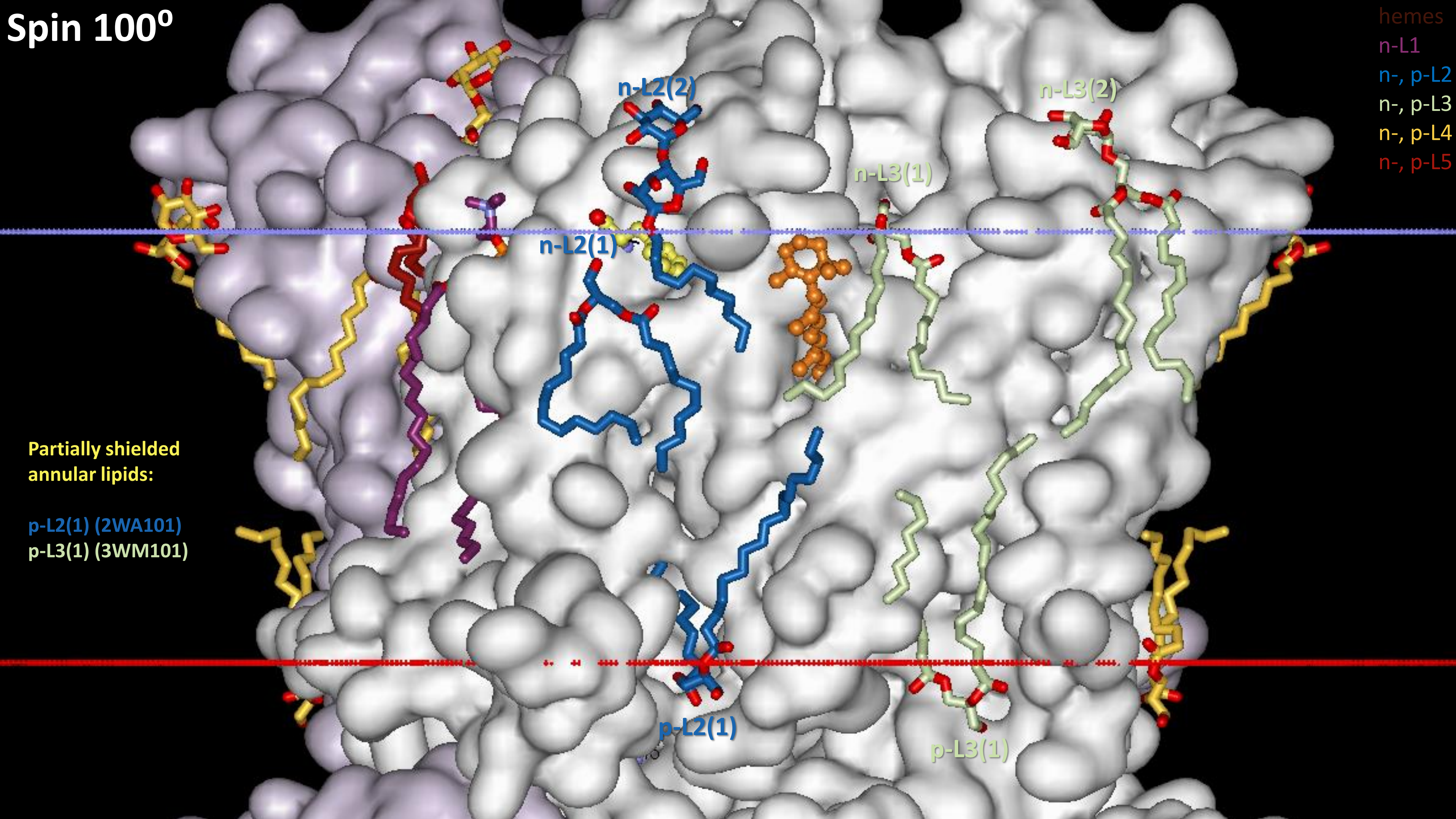
Spin 100º
hemes
n-L1
n-, p-L2
n-, p-L3
n-, p-L4
n-, p-L5
n-L2(2)
n-L2(1)
n-L3(1)
n-L3(2)
Partially shielded
annular lipids:
p-L2(1) (2WA101)
p-L3(1) (3WM101)
p-L2(1)
p-L3(1)

Spin 110º

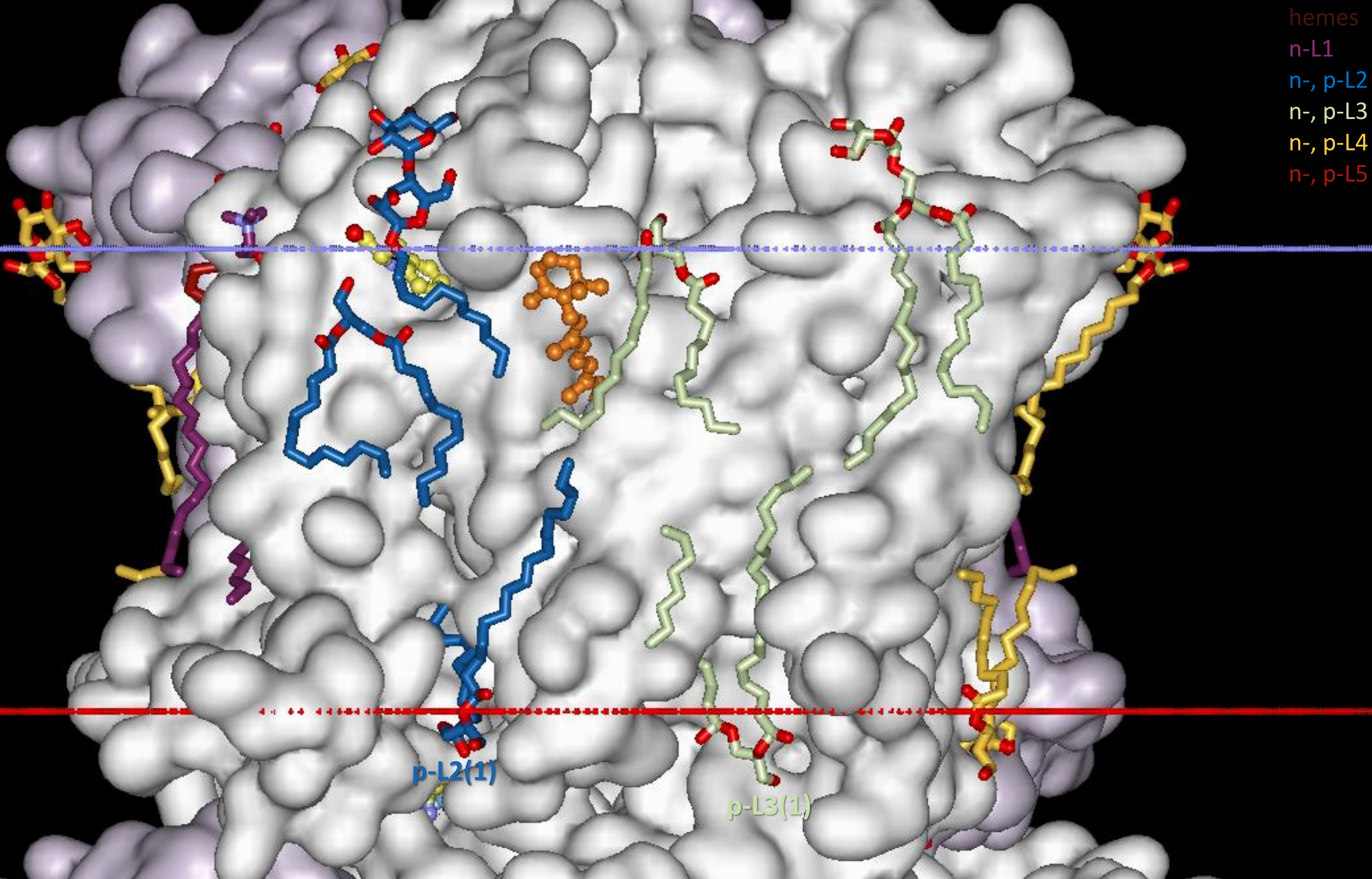

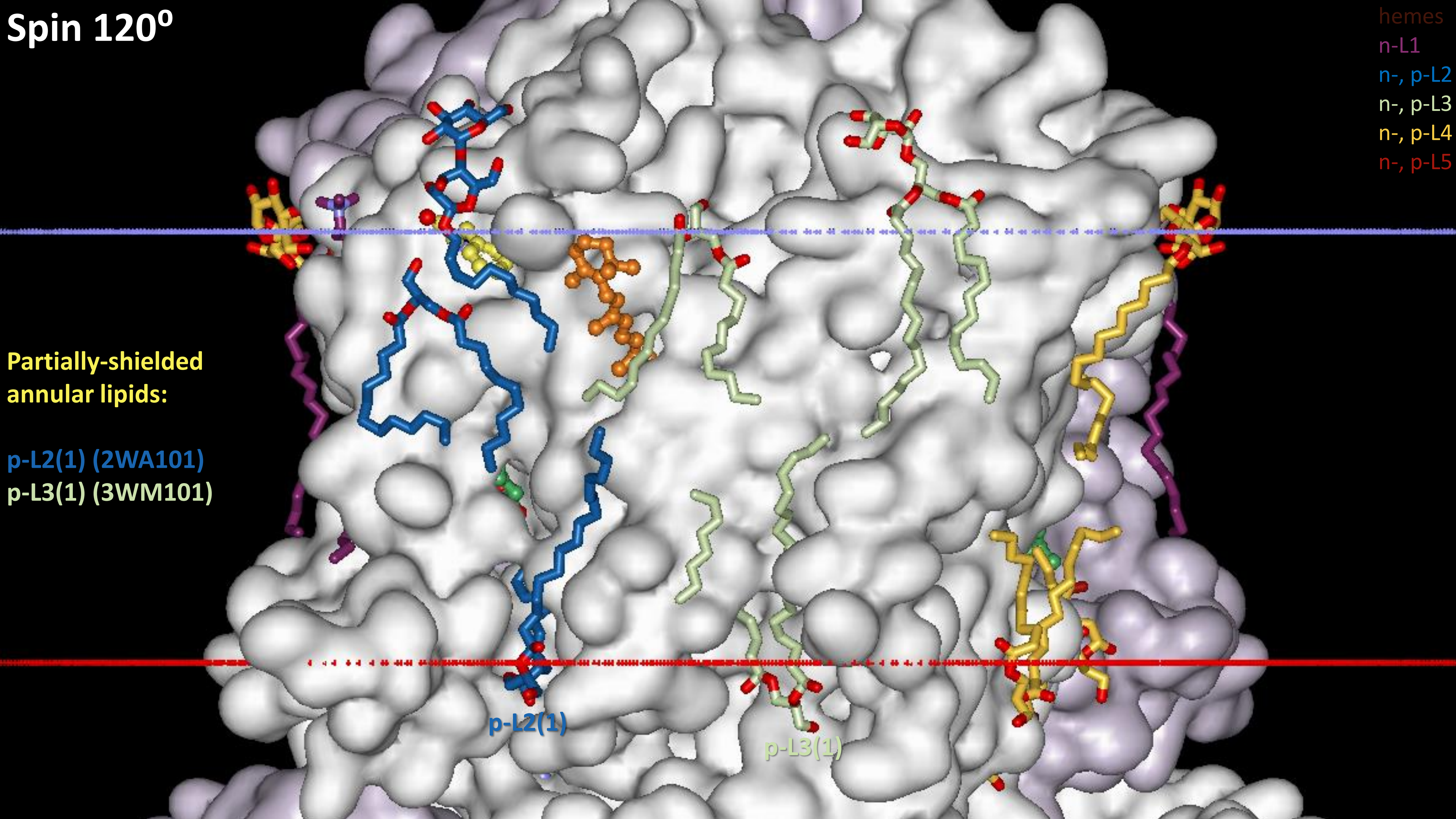
Spin 120°
hemes
n-L1
n-, p-L2
n-, p-L3
n-, p-L4
n-, p-L5
Partially-shielded annular lipids:
p-L2(1) (2WA101)
p-L3(1) (3WM101)
p-L2(1)
p-L3(1)

Spin 130º

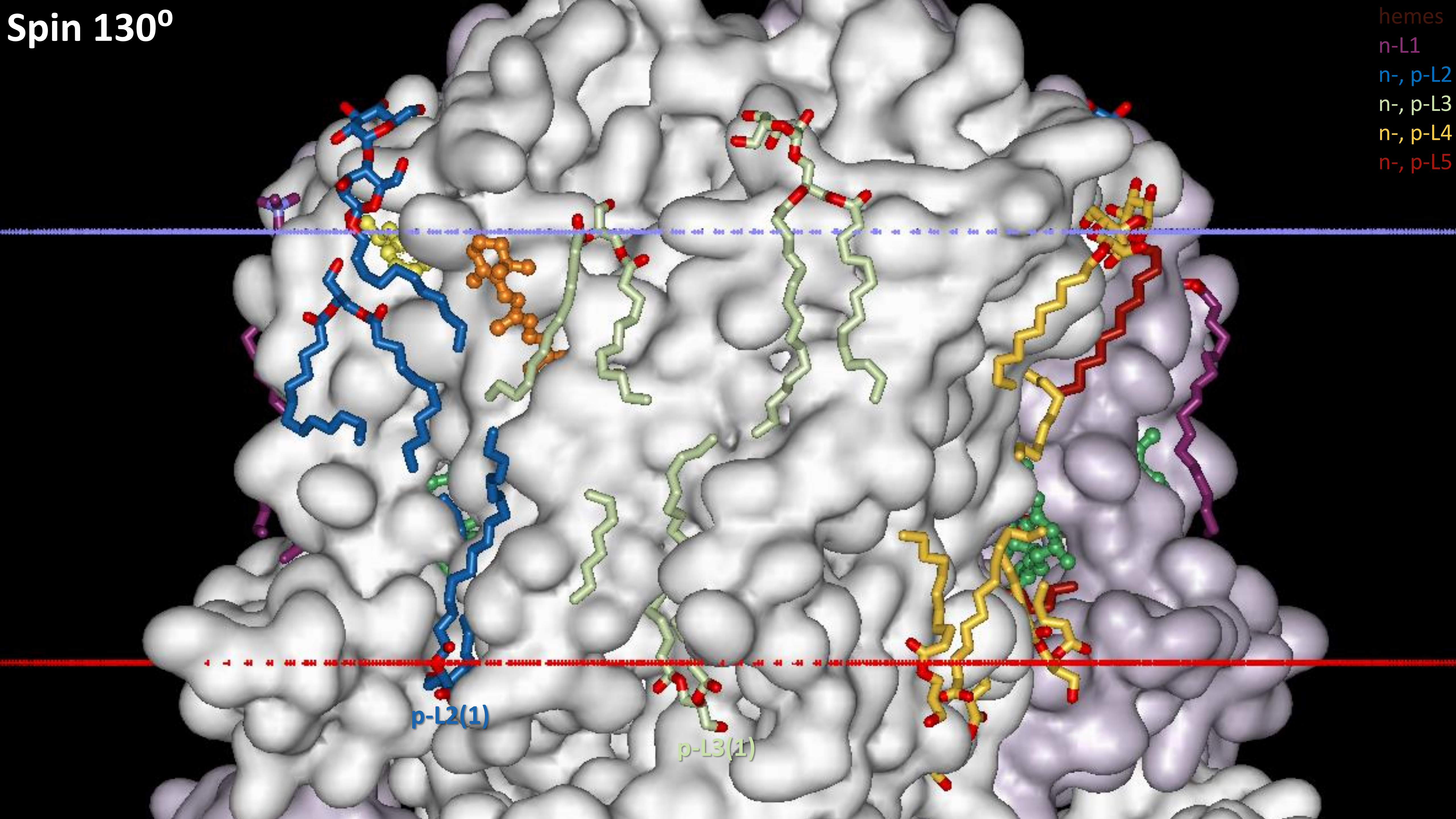

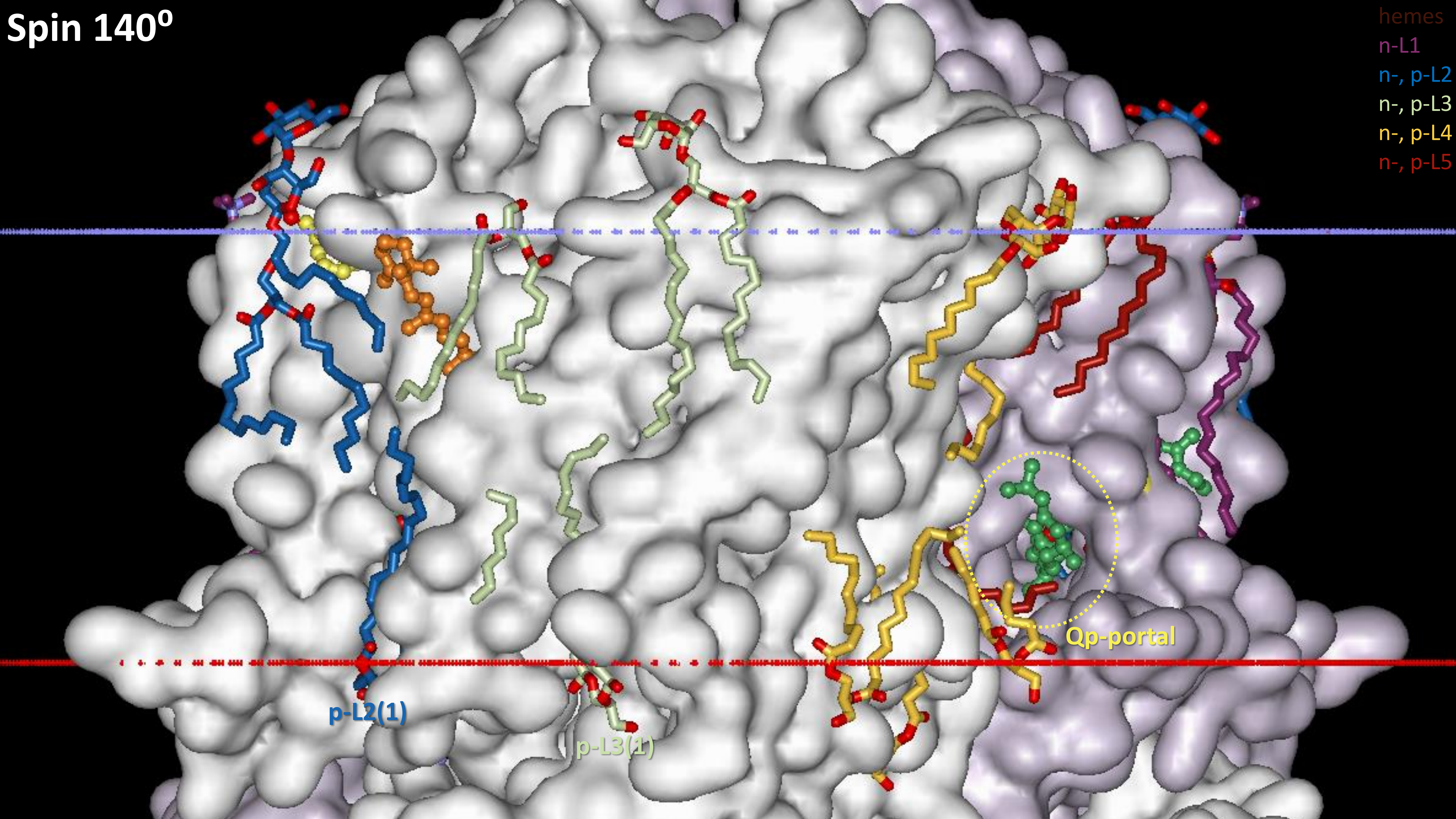
Spin 140°
hemes
n-L1
n-, p-L2
n-, p-L3
n-, p-L4
n-, p-L5
Qp-portal
p-L2(1)
p-L3(1)

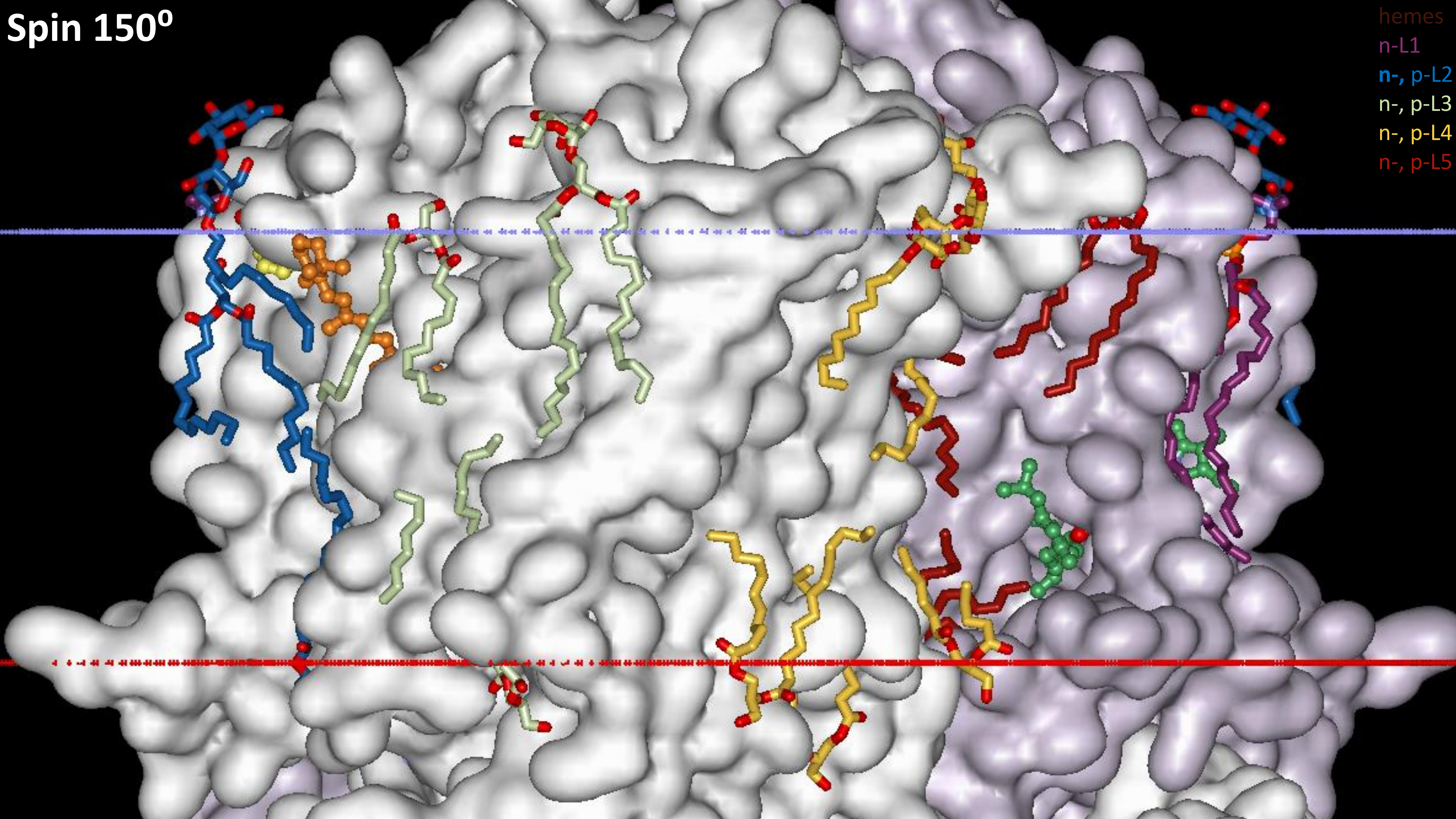
Spin 150º
hemes
n-L1
n-, p-L2
n-, p-L3
n-, p-L4
n-, p-L5

Spin 160º

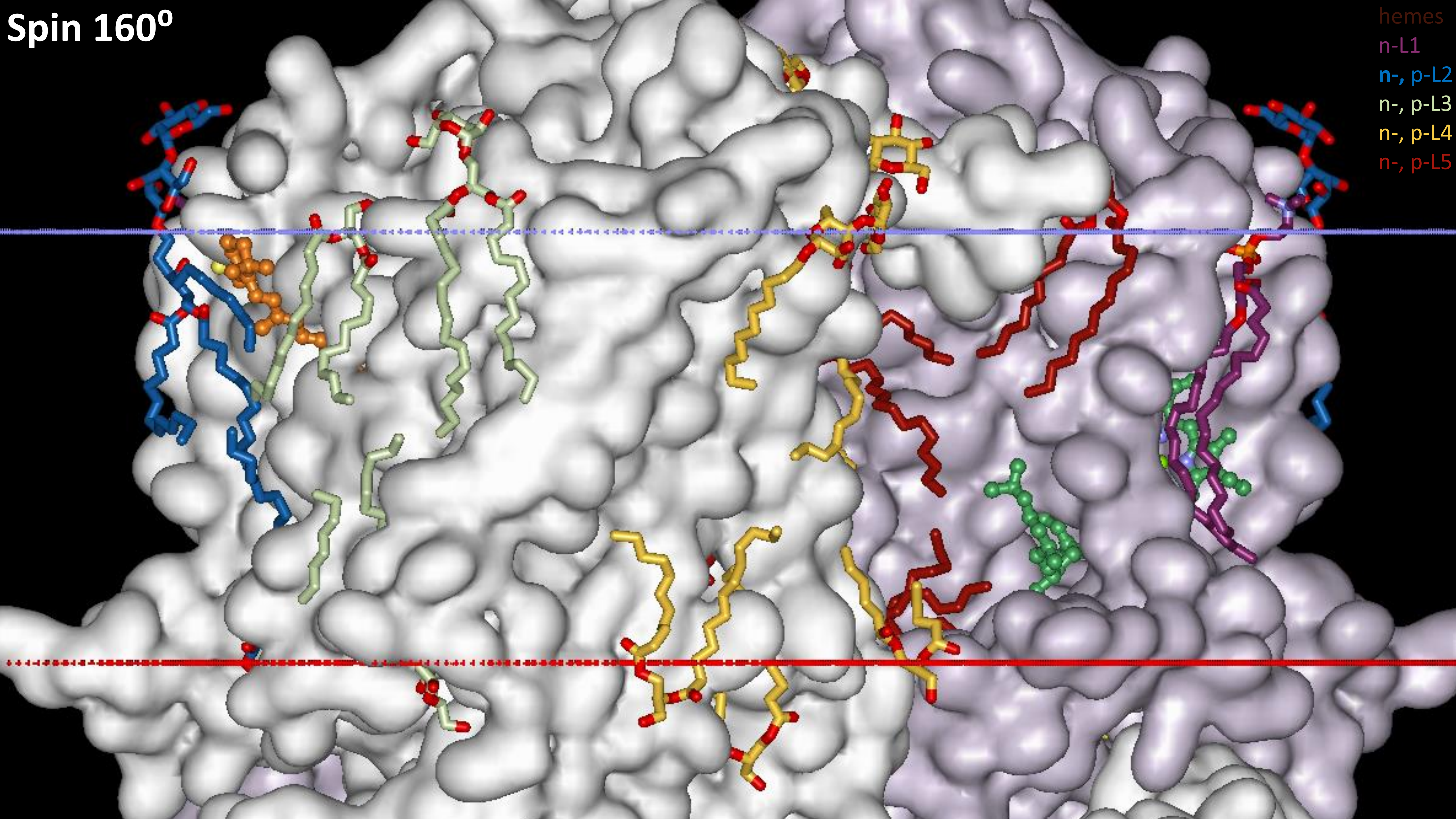

Spin 170º

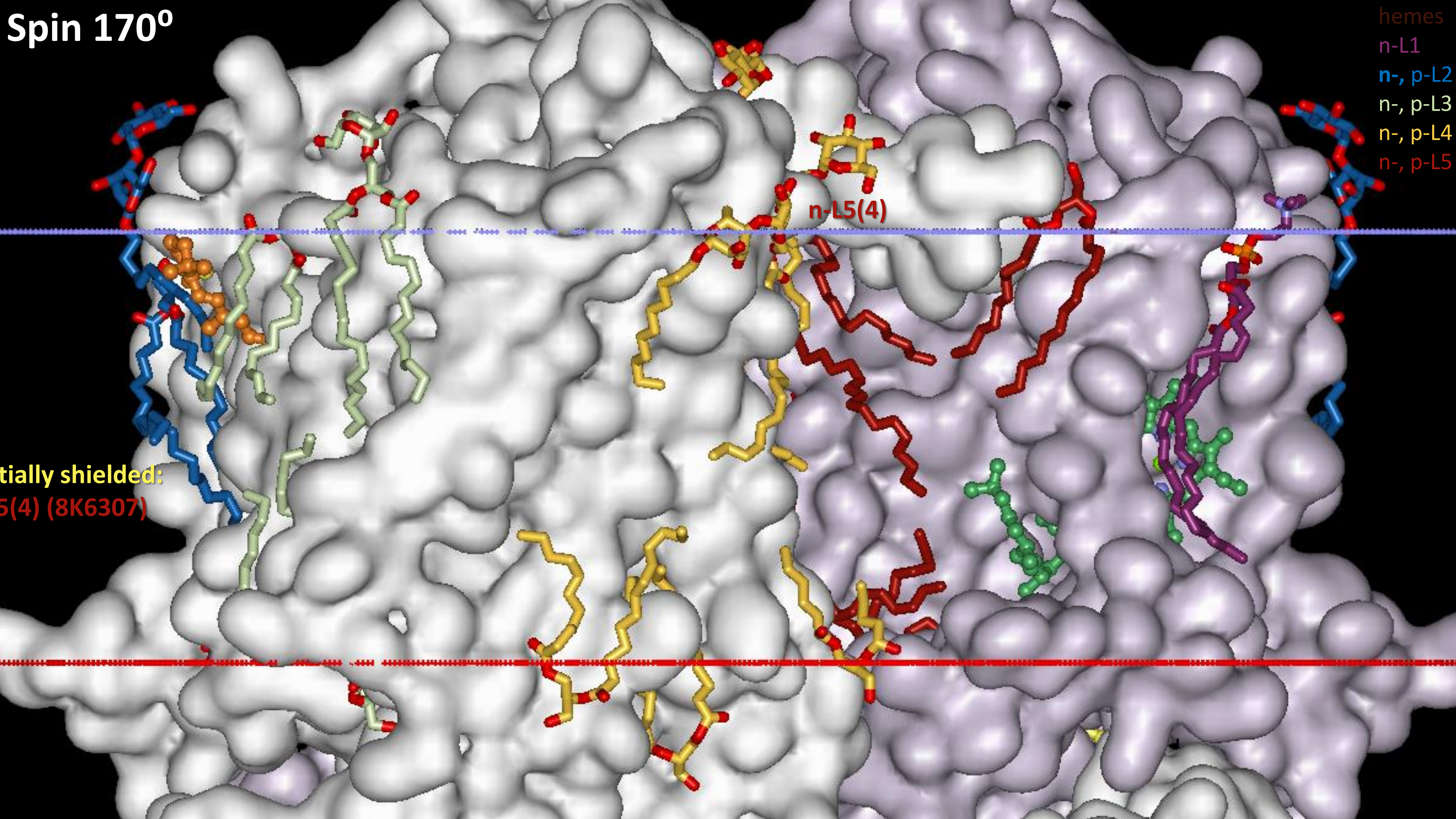
hemes
n-L1
n-, p-L2
n-, p-L3
n-, p-L4
n-, p-L5
n-L5(4)
tially shielded:
5(4) (8K6307)

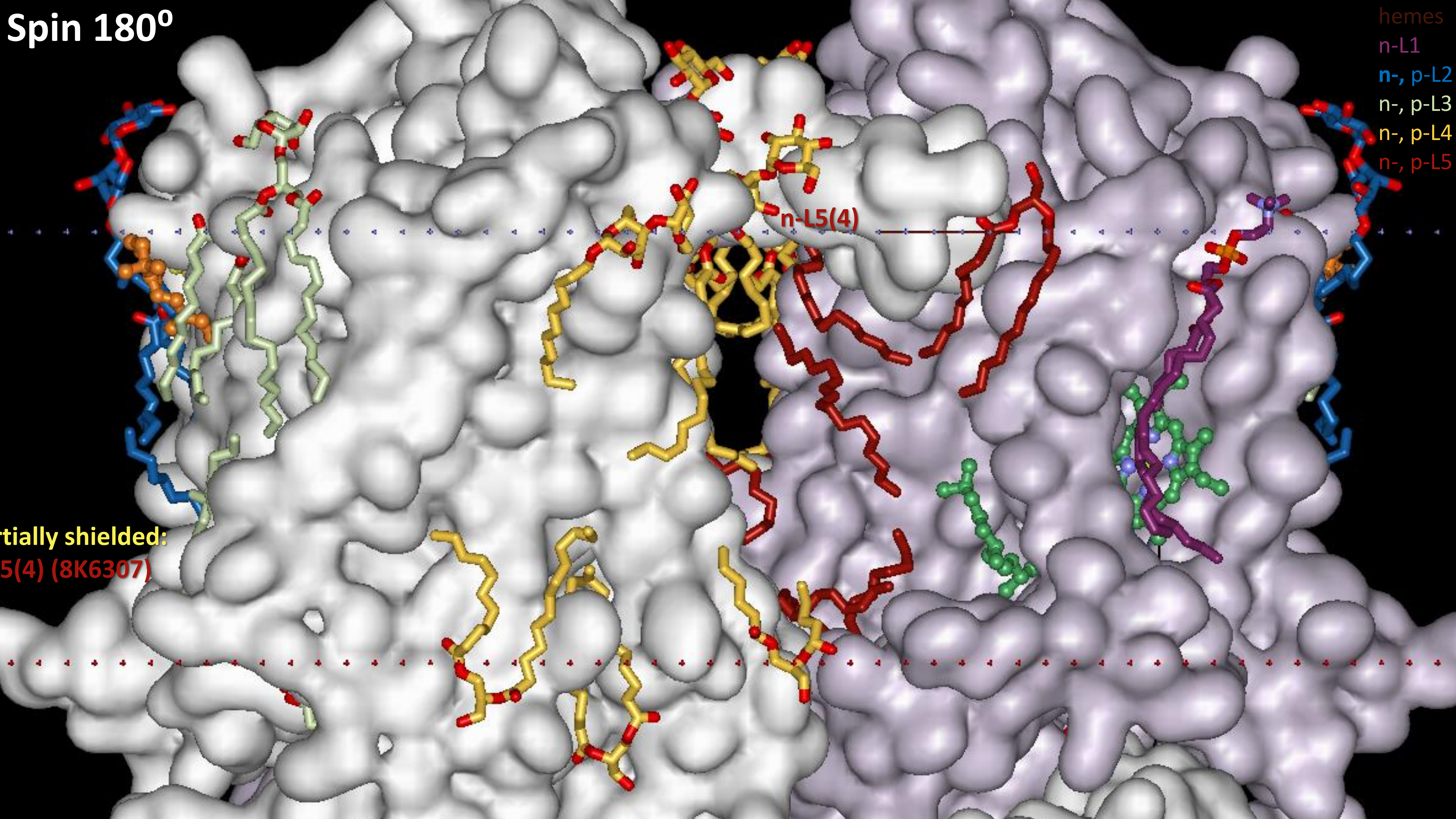
Spin 180º
hemes
n-L1
n-, p-L2
n-, p-L3
n-, p-L4
n-, p-L5
n-L5(4)
rtially shielded:
5(4) (8K6307)

Cyt*b6f* monomer front view

Spin 0º

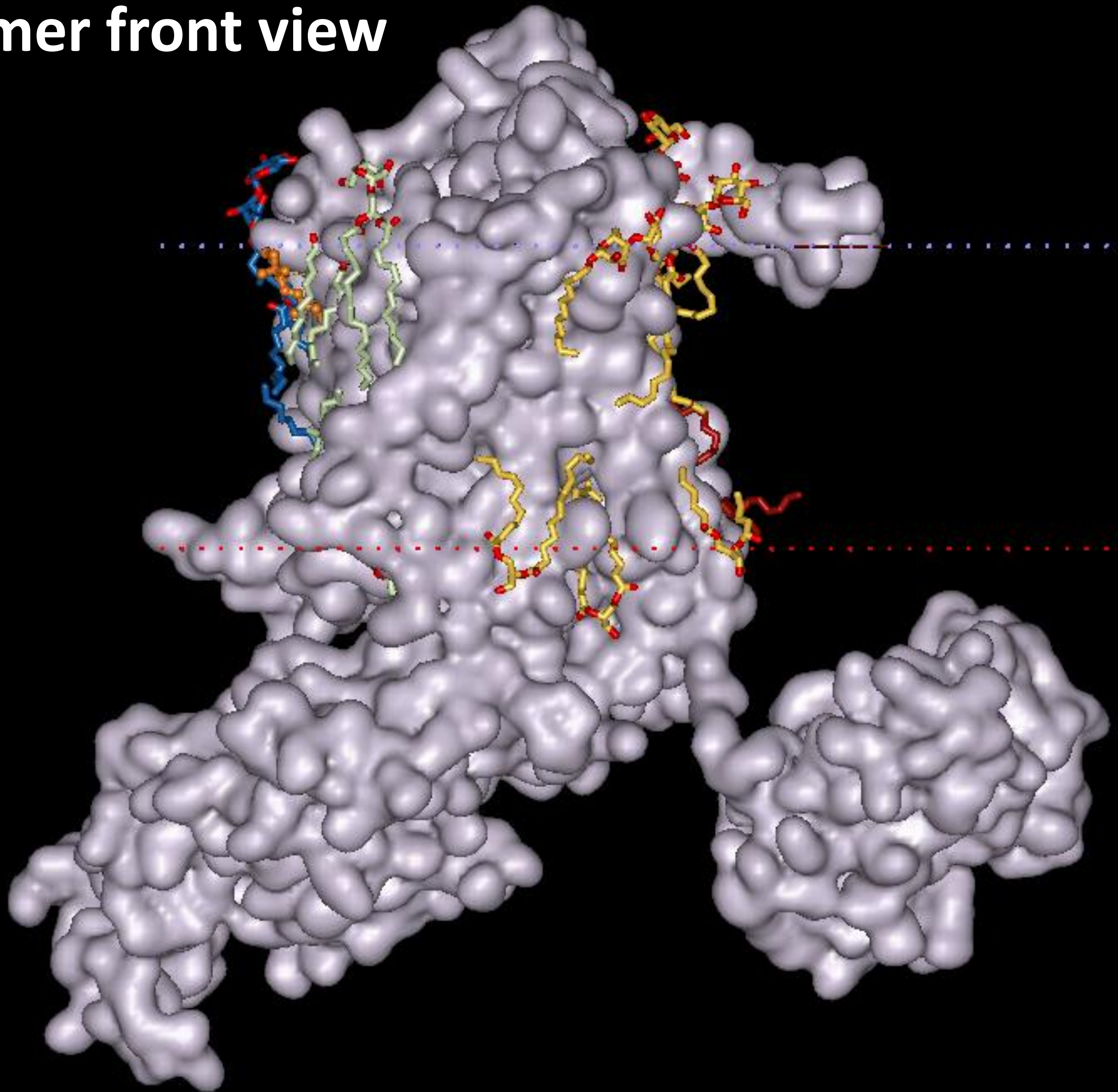

Cyt*b6f*:
Monomer 2

Chl*a*
β-Car
hemes
n-L1
n-, p-L2
n-, p-L3
n-, p-L4
n-, p-L5

Cyt*b*6*f* monomer front view the membrane part

Spin 0º

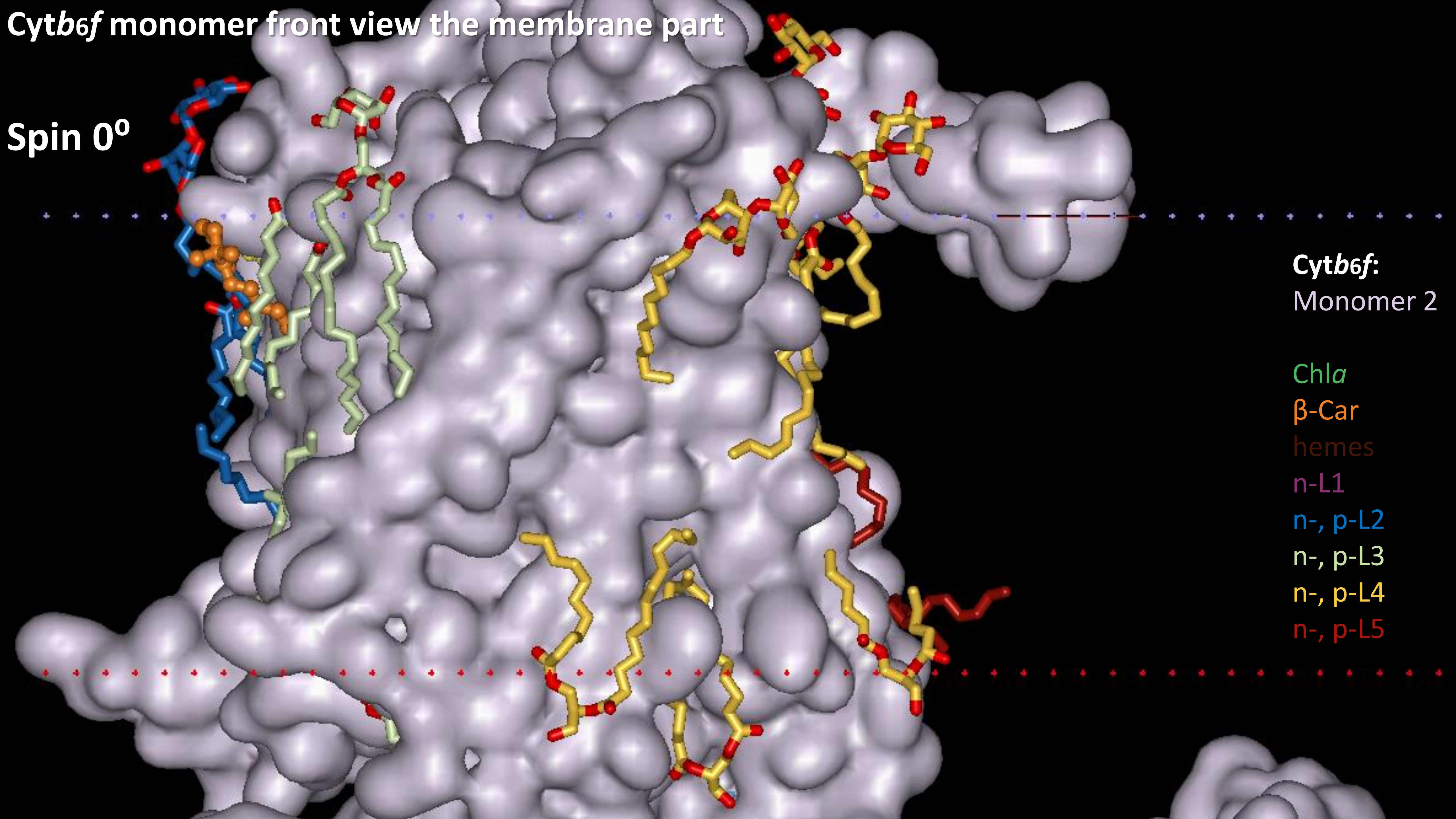

Spin 10°

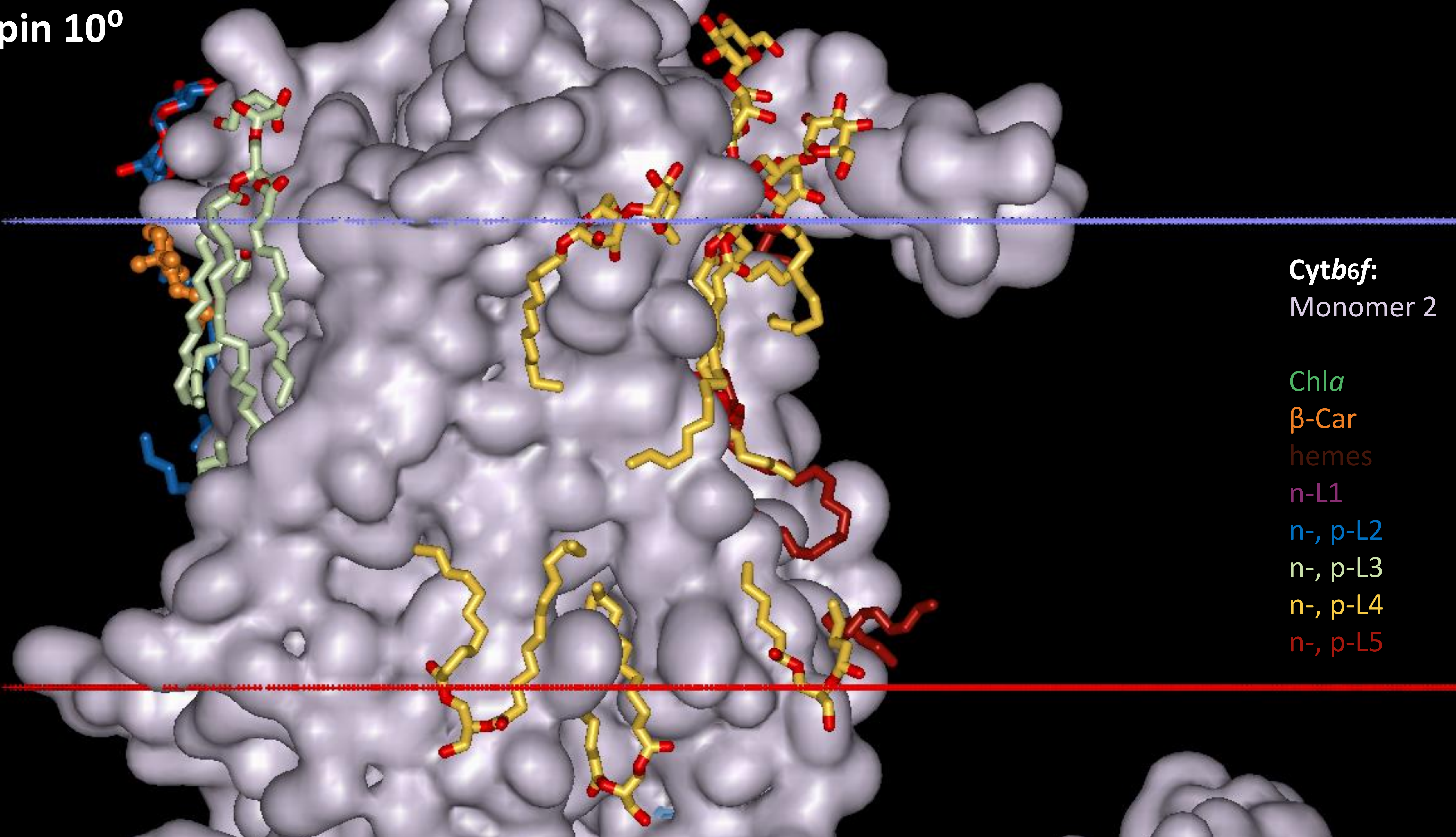

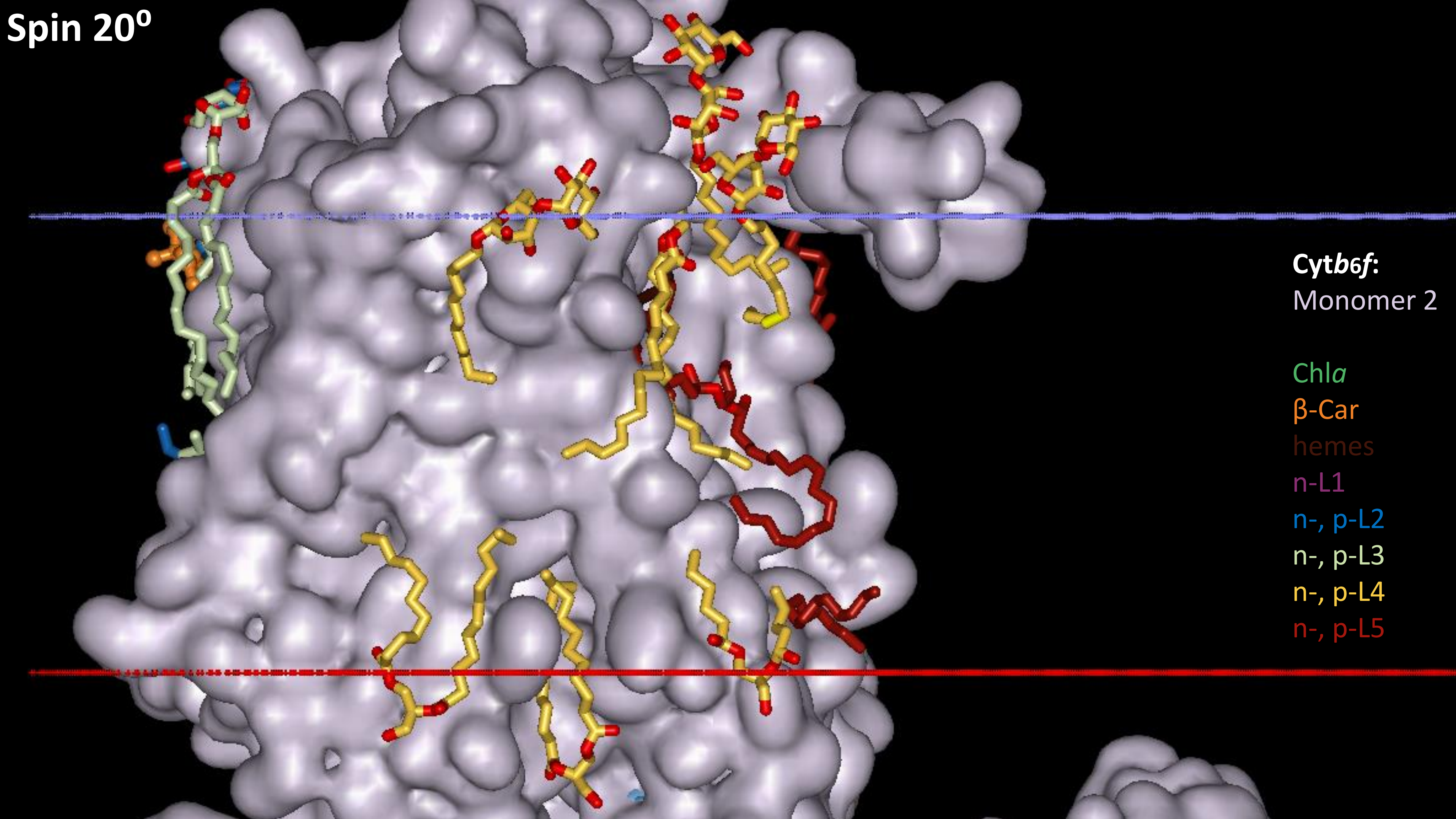
Spin 20°
Cyt*b6f*:
Monomer 2
Chl*a*
β-Car
hemes
n-L1
n-, p-L2
n-, p-L3
n-, p-L4
n-, p-L5

Spin 30°

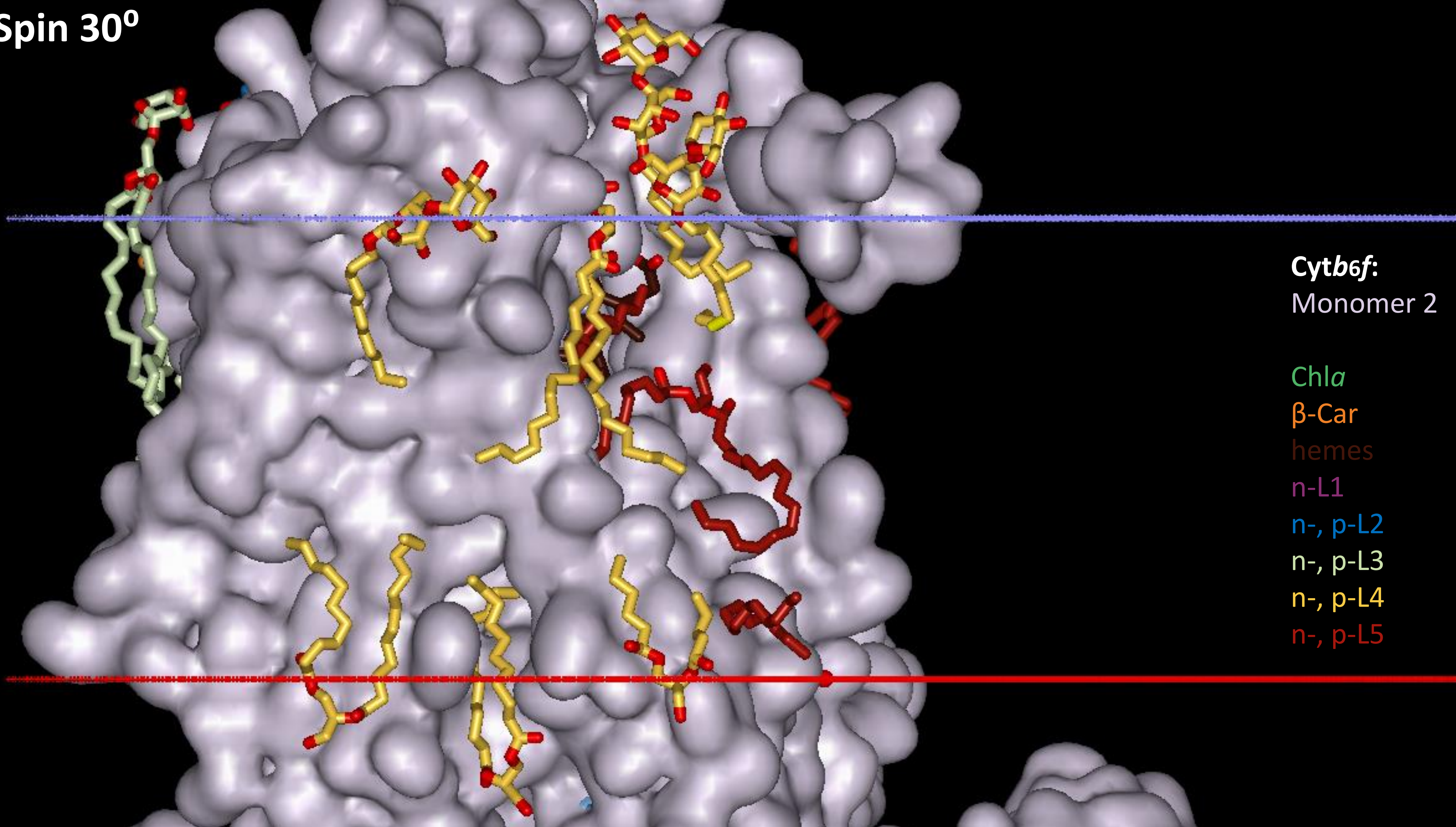

Cyt*b6f*:
Monomer 2

Chl*a*
β-Car
hemes
n-L1
n-, p-L2
n-, p-L3
n-, p-L4
n-, p-L5

Spin 40º

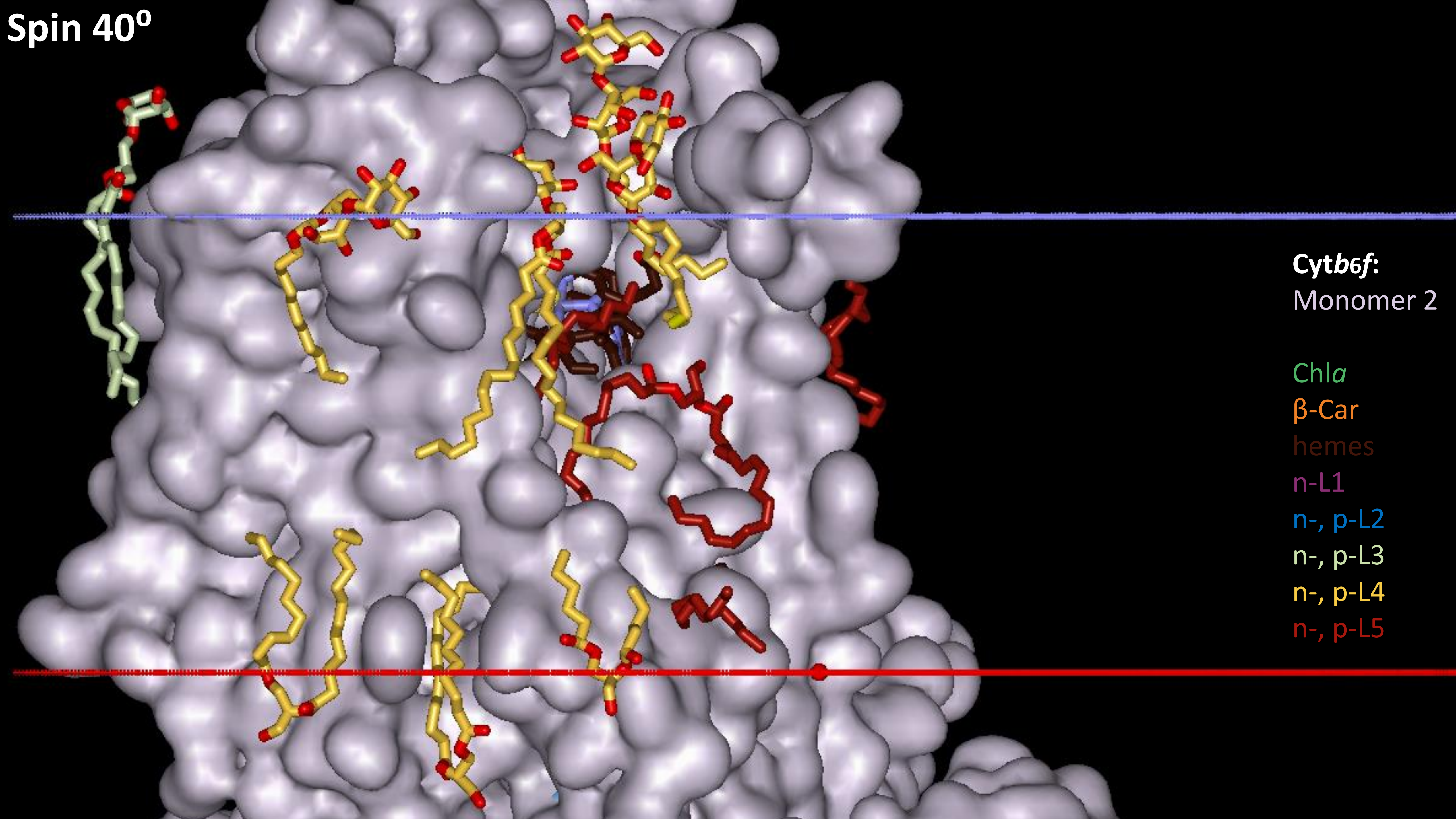

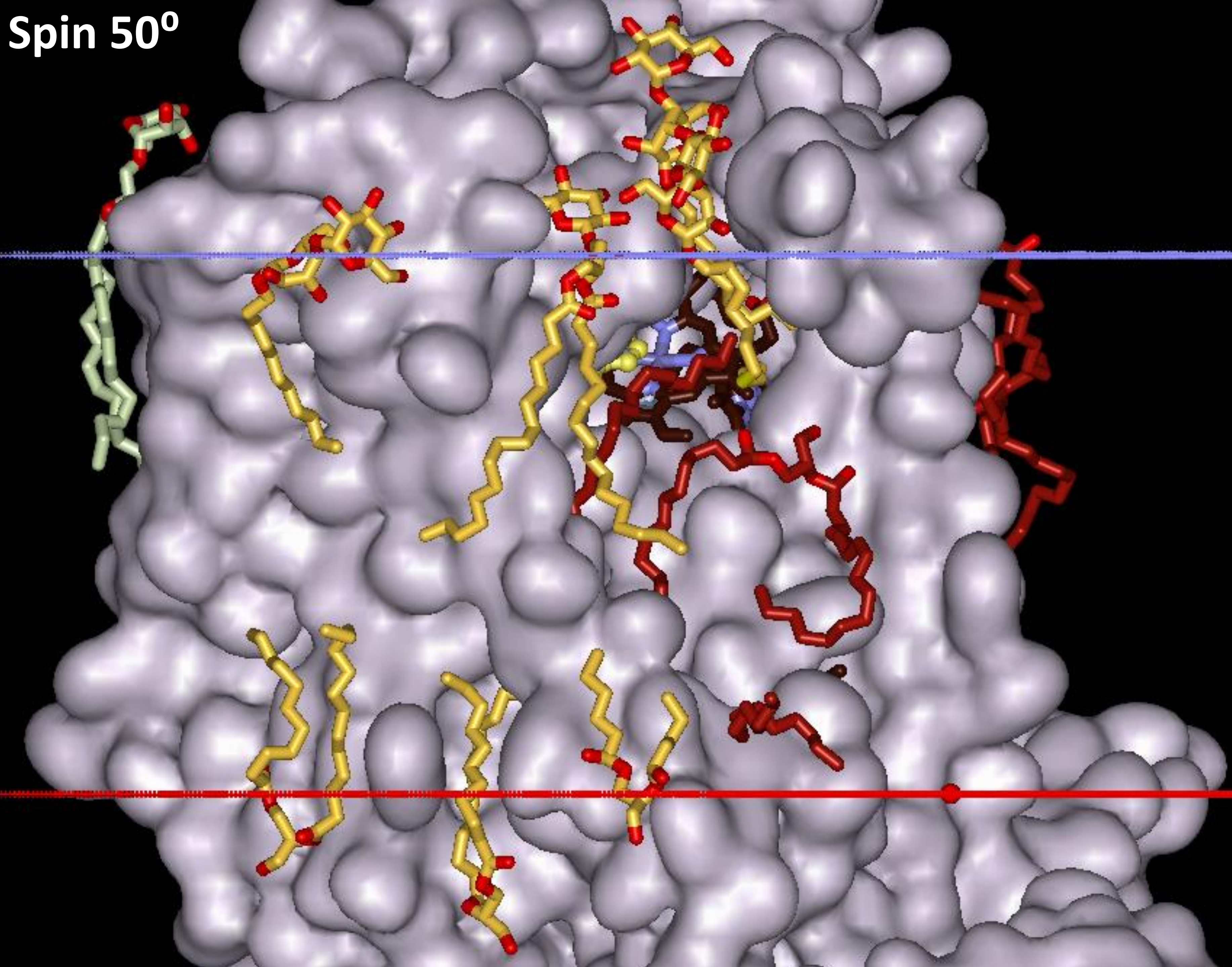

Cyt*b6f*:
Monomer 2

Chl*a*
β-Car
hemes
n-L1
n-, p-L2
n-, p-L3
n-, p-L4
n-, p-L5

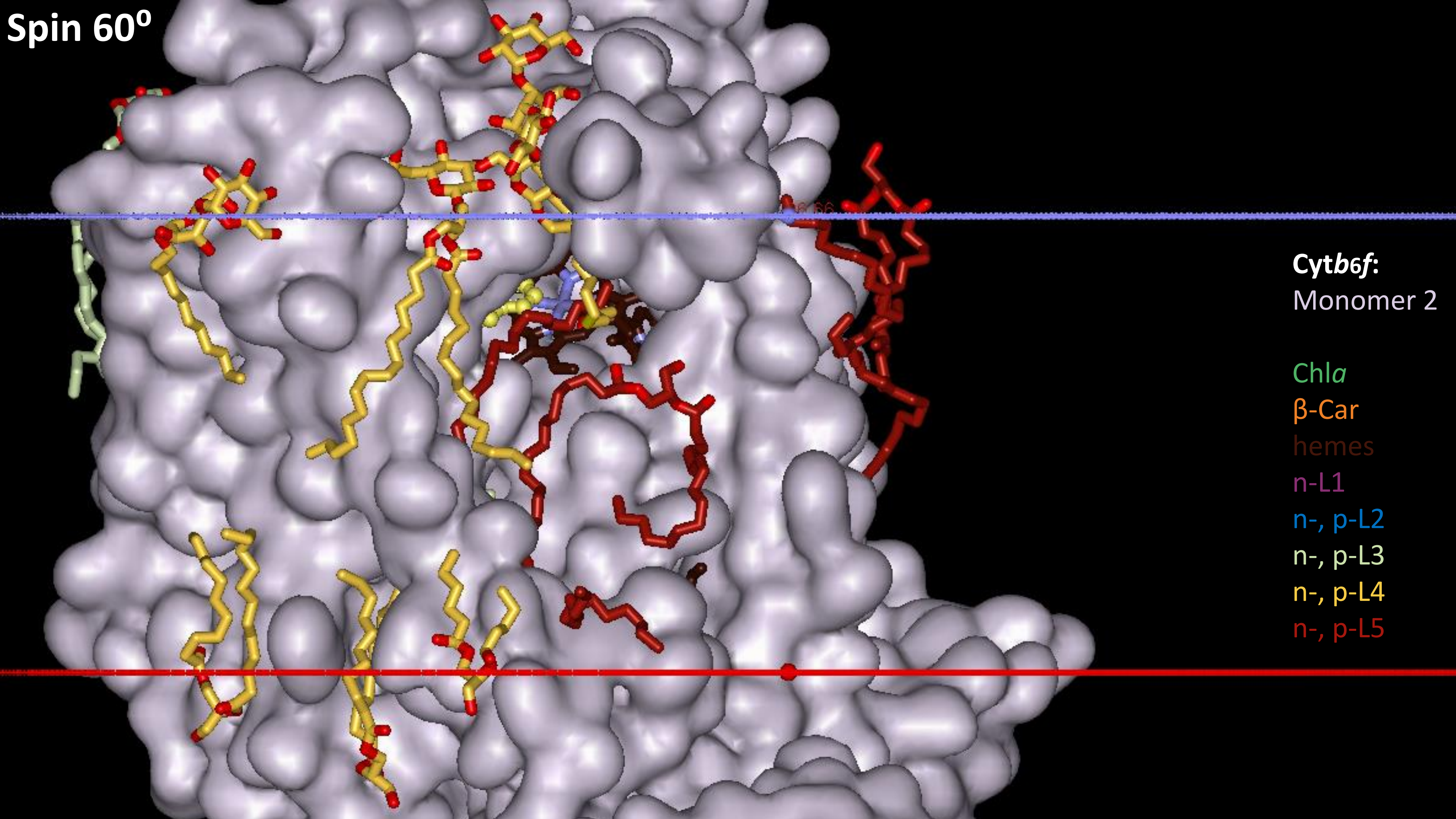
Spin 60º
Cyt*b6f*:
Monomer 2
Chl*a*
β-Car
hemes
n-L1
n-, p-L2
n-, p-L3
n-, p-L4
n-, p-L5

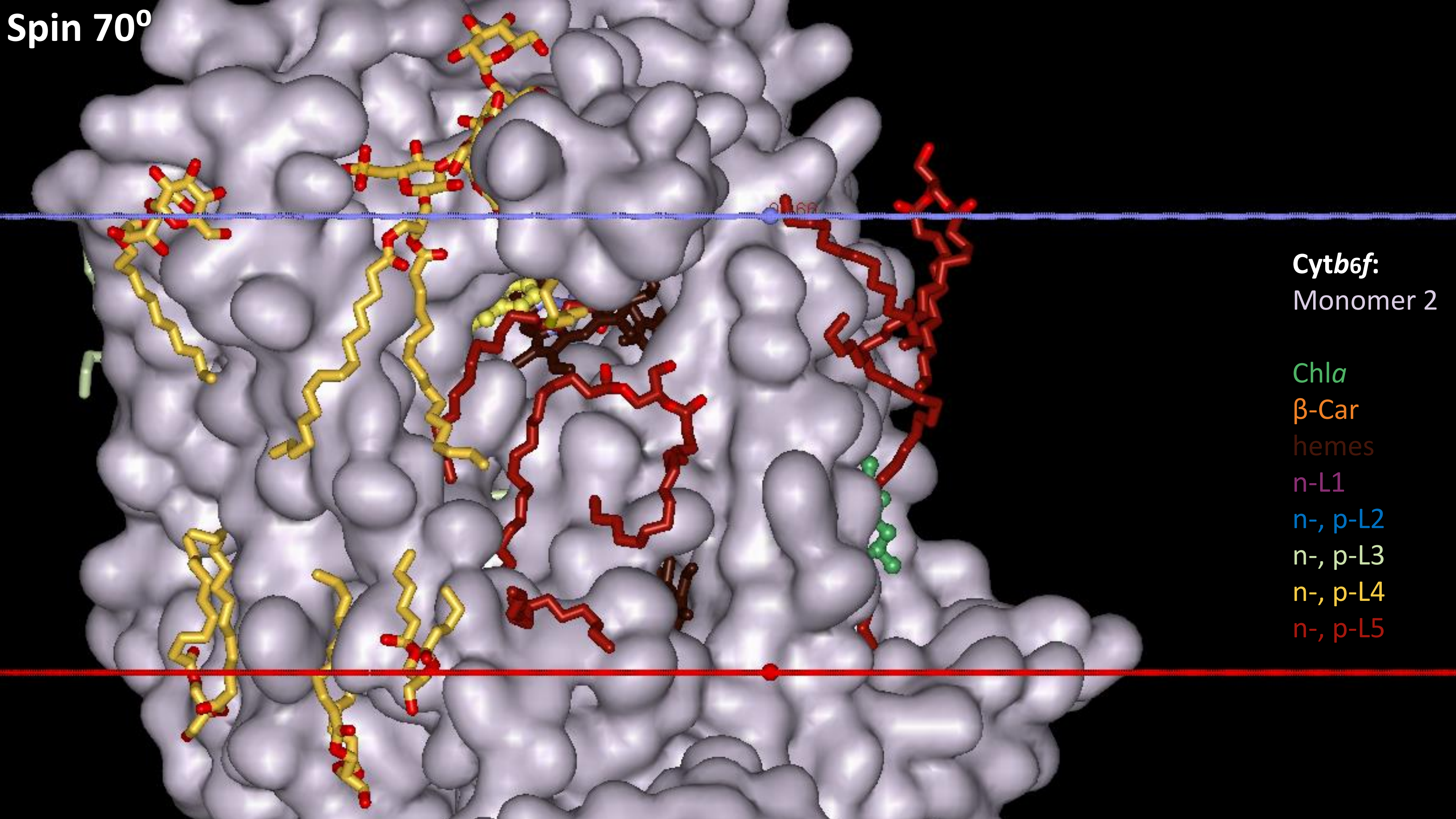

Spin 70°
Cytb6f:
Monomer 2
Chla
β-Car
hemes
n-L1
n-, p-L2
n-, p-L3
n-, p-L4
n-, p-L5

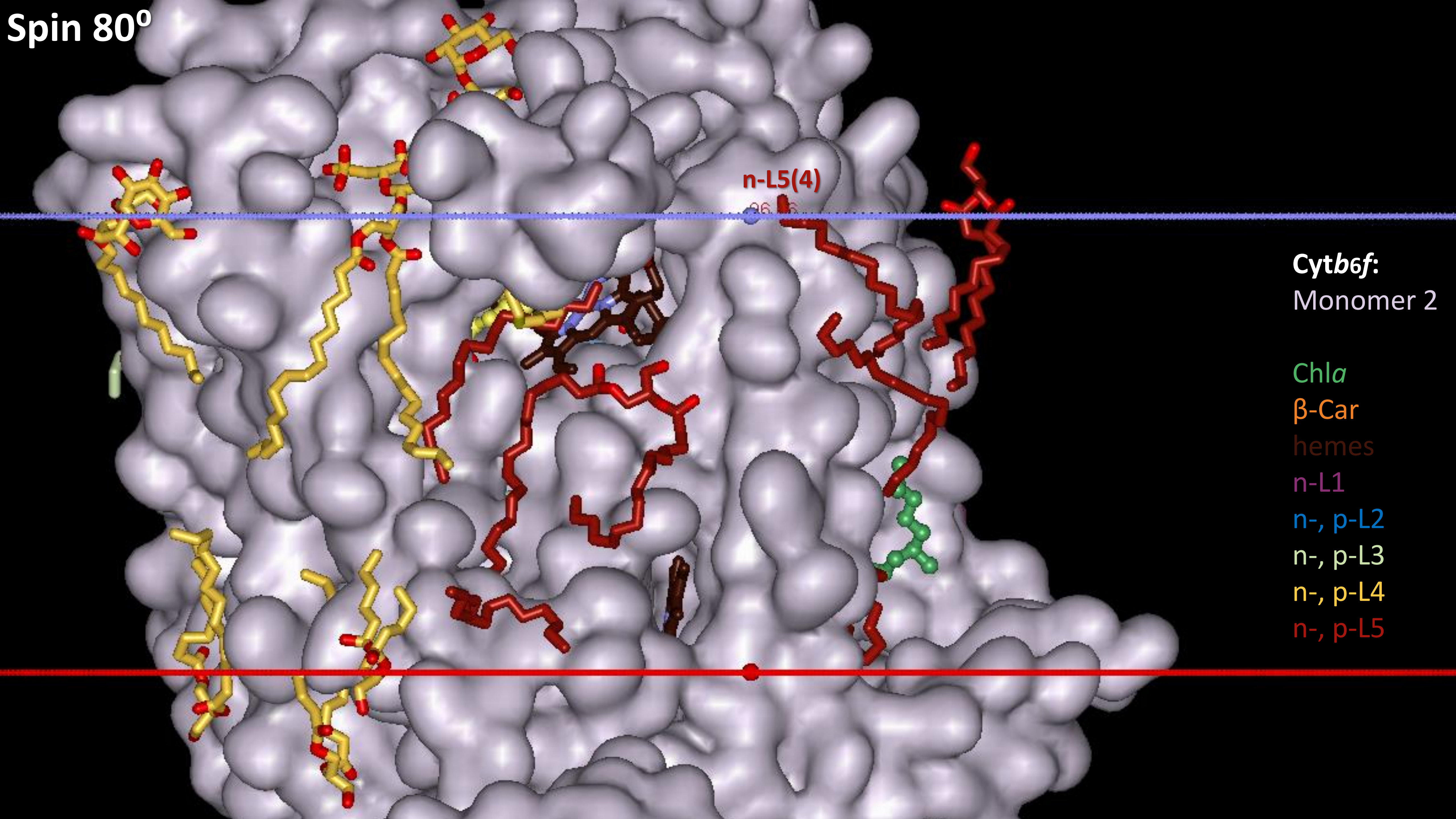
Spin 80°
n-L5(4)
Cyt*b6f*:
Monomer 2
Chl*a*
β-Car
hemes
n-L1
n-, p-L2
n-, p-L3
n-, p-L4
n-, p-L5

Spin 90º

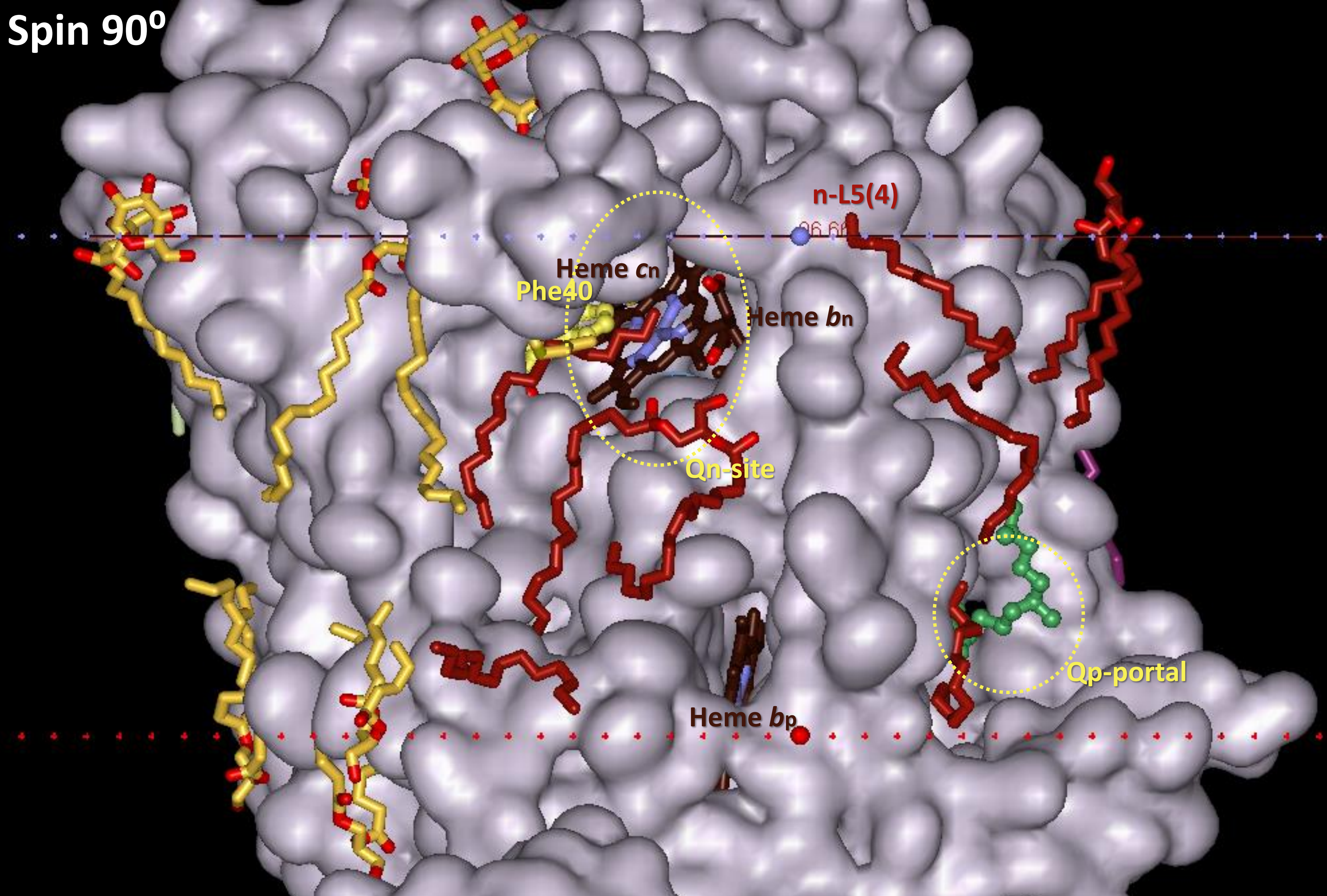

Cyt*b6f*:
Monomer 2

Chl*a*
β-Car
hemes
n-L1
n-, p-L2
n-, p-L3
n-, p-L4
n-, p-L5

Spin 100º

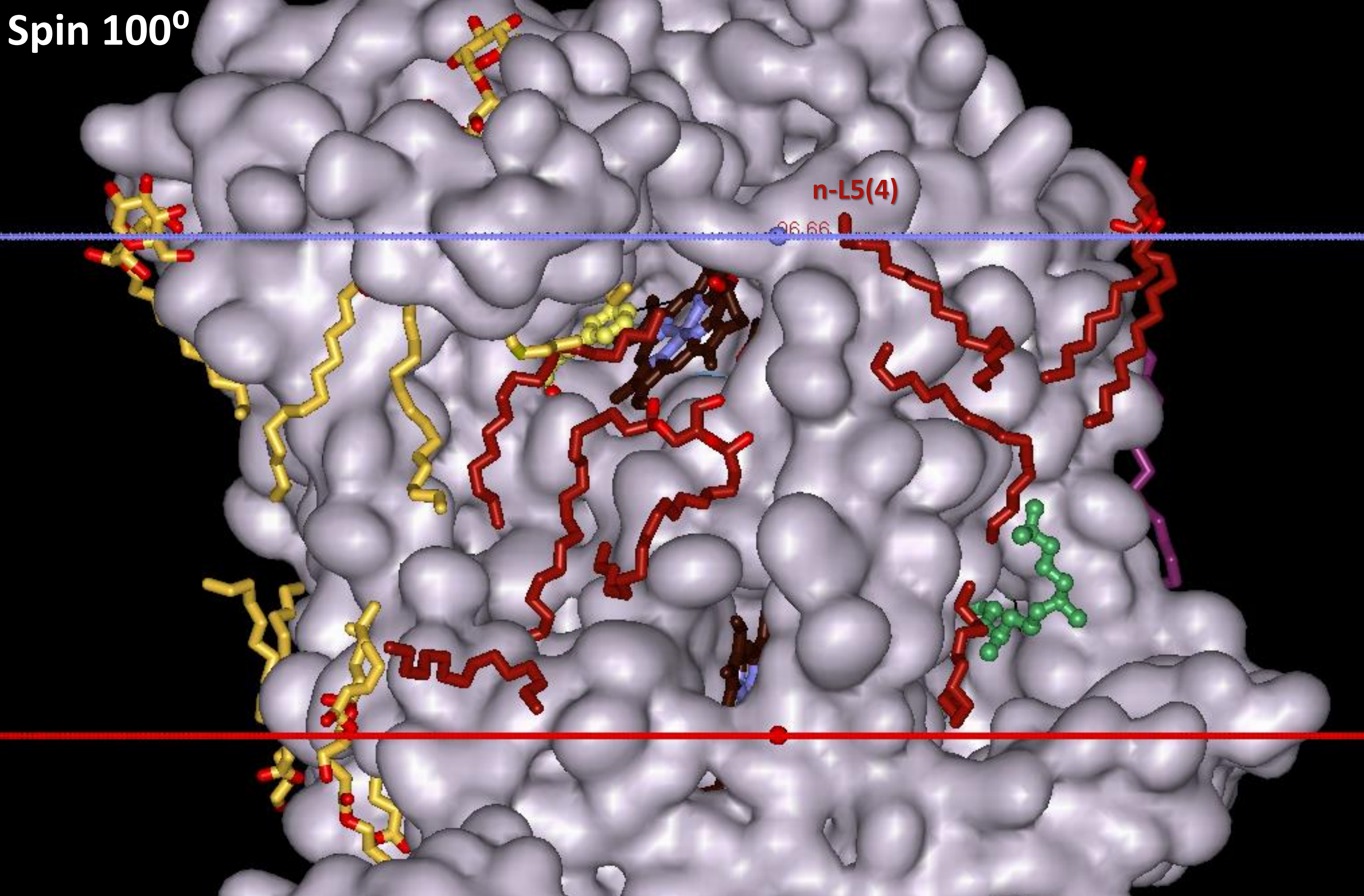
n-L5(4)
96.66
Cyt*b6f*:
Monomer 2
Chl*a*
β-Car
hemes
n-L1
n-, p-L2
n-, p-L3
n-, p-L4
n-, p-L5

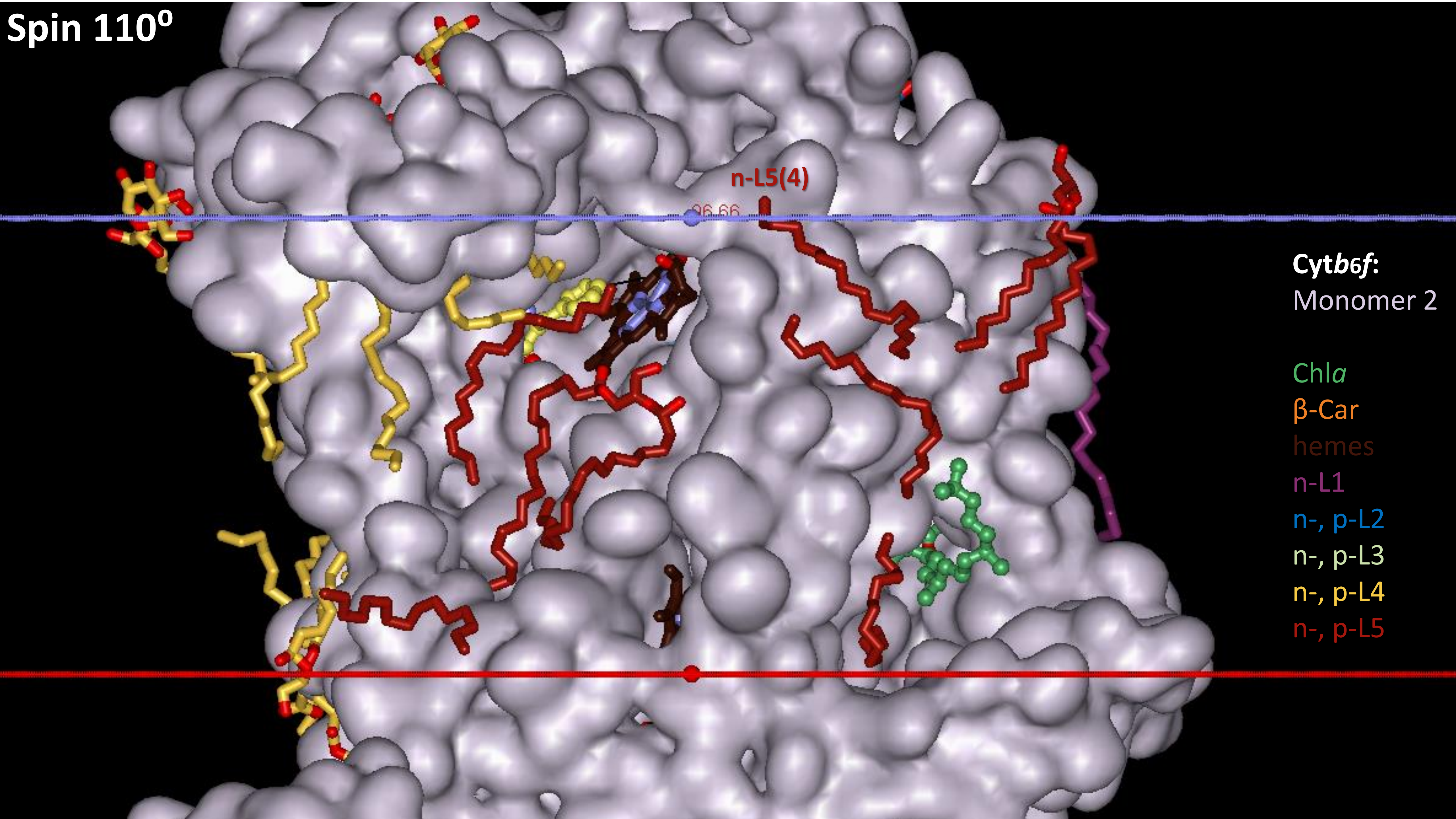
Spin 110°
n-L5(4)
Cytb6f:
Monomer 2
Chla
β-Car
hemes
n-L1
n-, p-L2
n-, p-L3
n-, p-L4
n-, p-L5

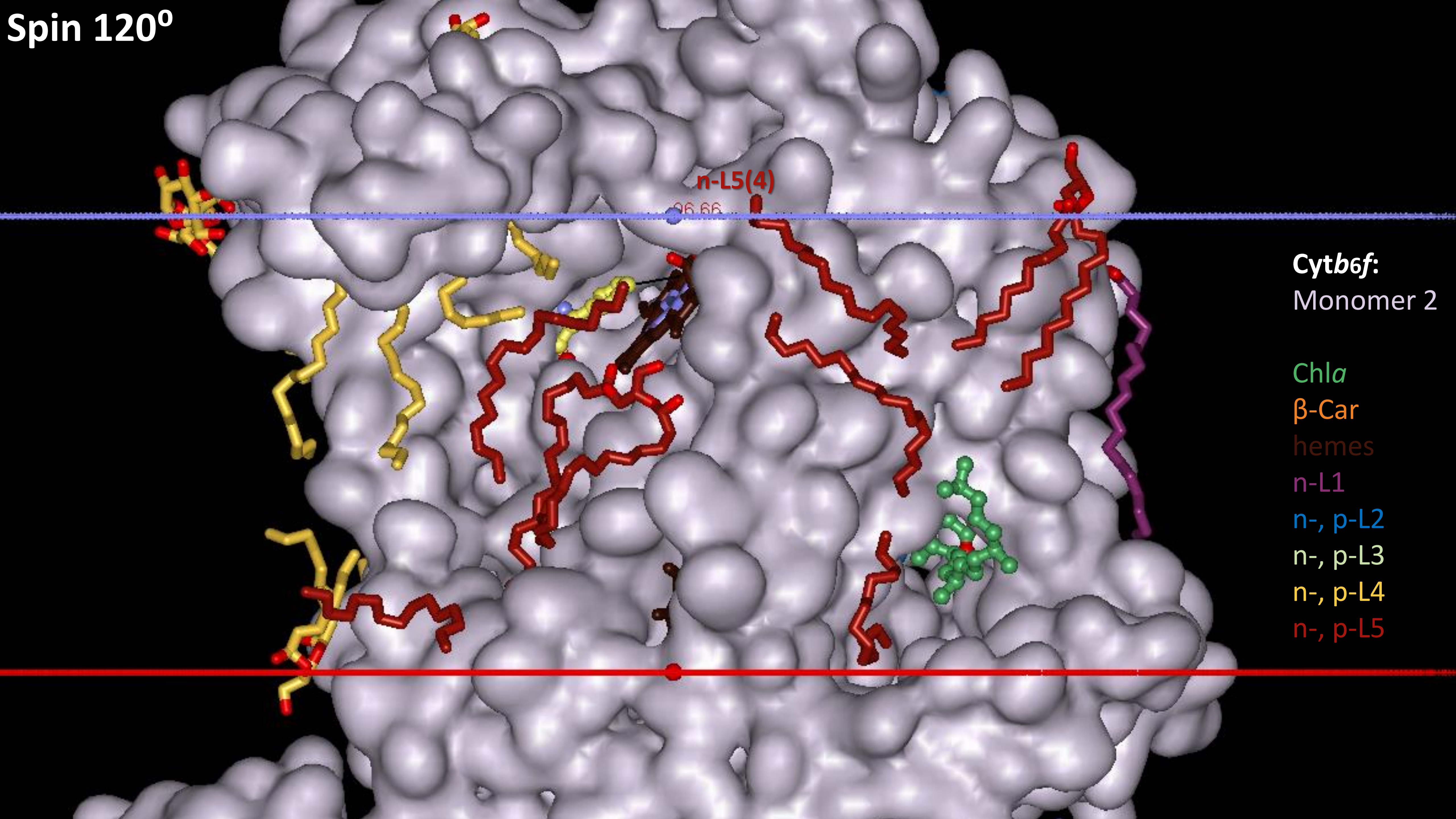
Spin 120º
n-L5(4)
96.66
Cyt*b*6*f*:
Monomer 2
Chl*a*
β-Car
hemes
n-L1
n-, p-L2
n-, p-L3
n-, p-L4
n-, p-L5

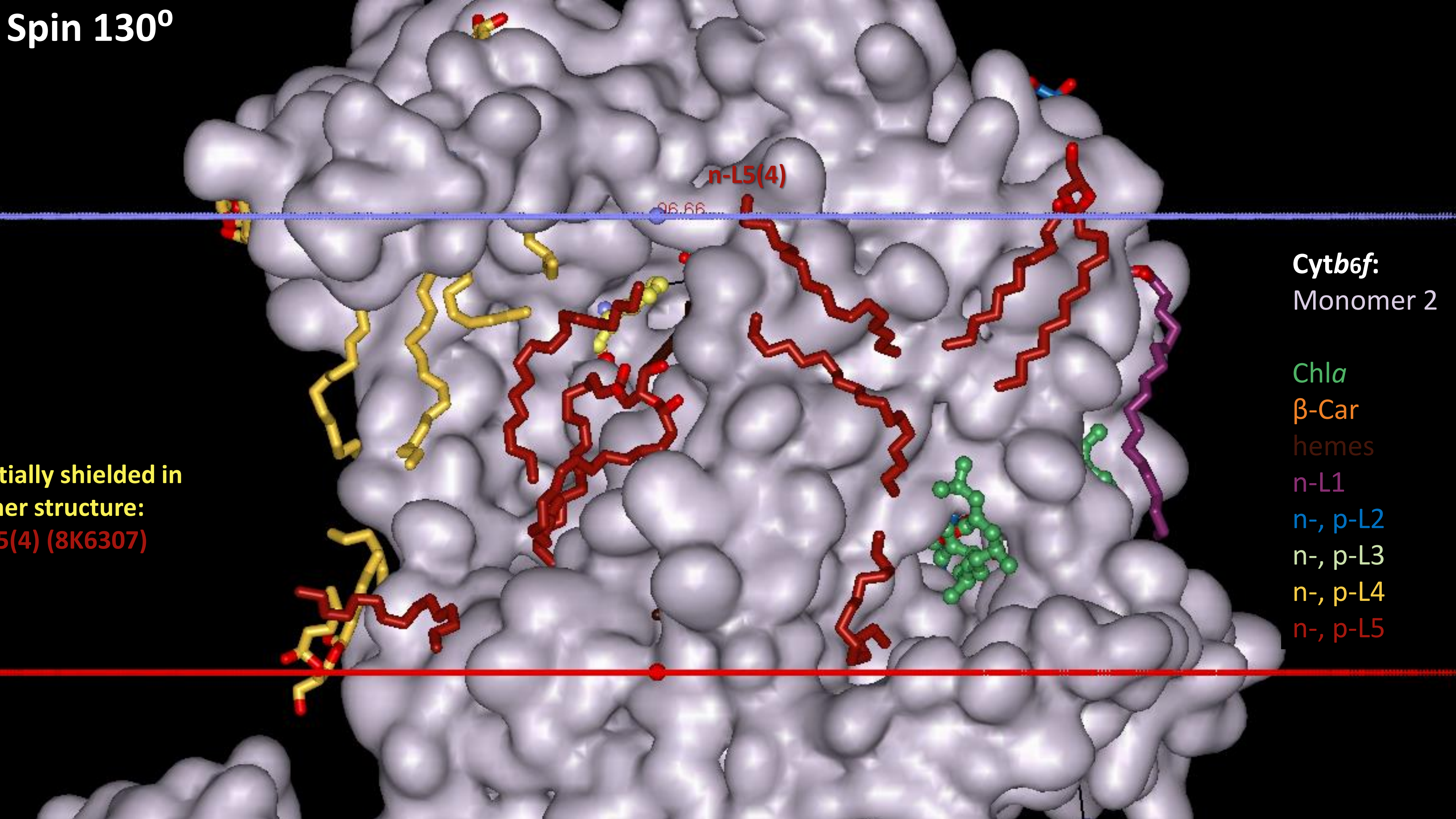

Spin 130º
n-L5(4)
96.66
tially shielded in
ner structure:
5(4) (8K6307)
Cytb6f:
Monomer 2
Chla
β-Car
hemes
n-L1
n-, p-L2
n-, p-L3
n-, p-L4
n-, p-L5

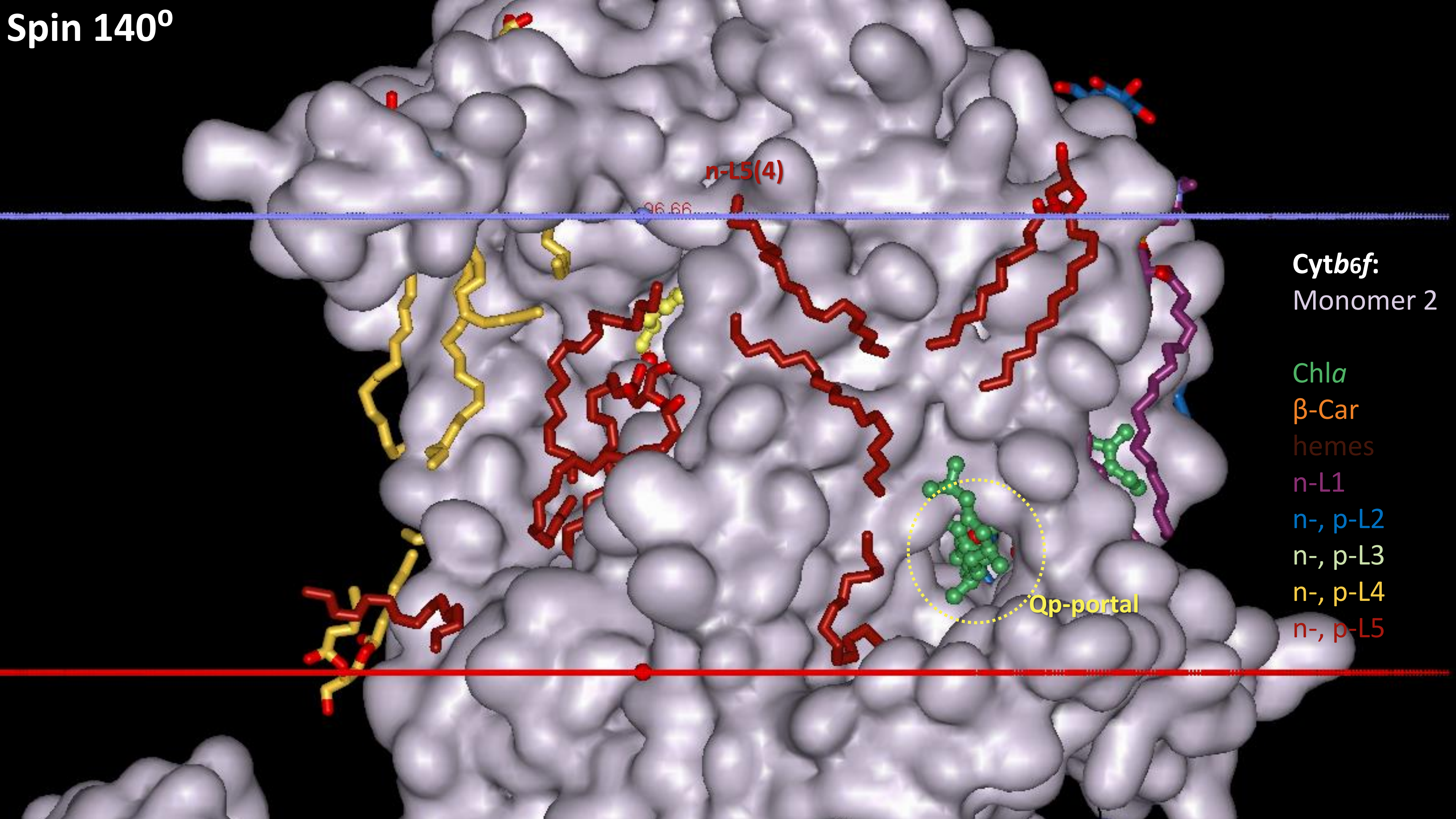
Spin 140º
n-L5(4)
96.66
Qp-portal
Cytb6f:
Monomer 2
Chla
β-Car
hemes
n-L1
n-, p-L2
n-, p-L3
n-, p-L4
n-, p-L5

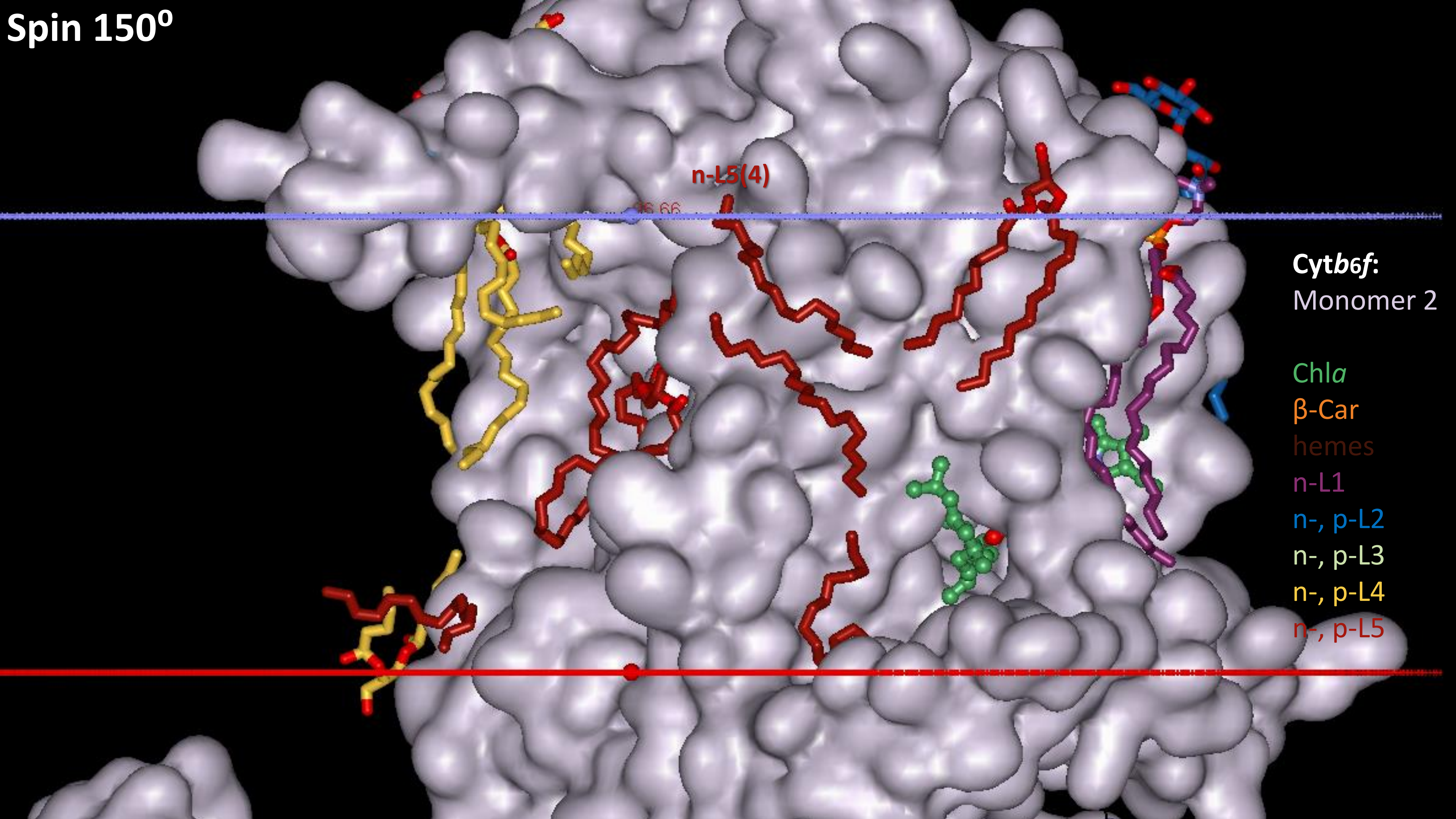
Spin 150º
n-L5(4)
Cytb6f:
Monomer 2
Chla
β-Car
hemes
n-L1
n-, p-L2
n-, p-L3
n-, p-L4
n-, p-L5

Spin 160°

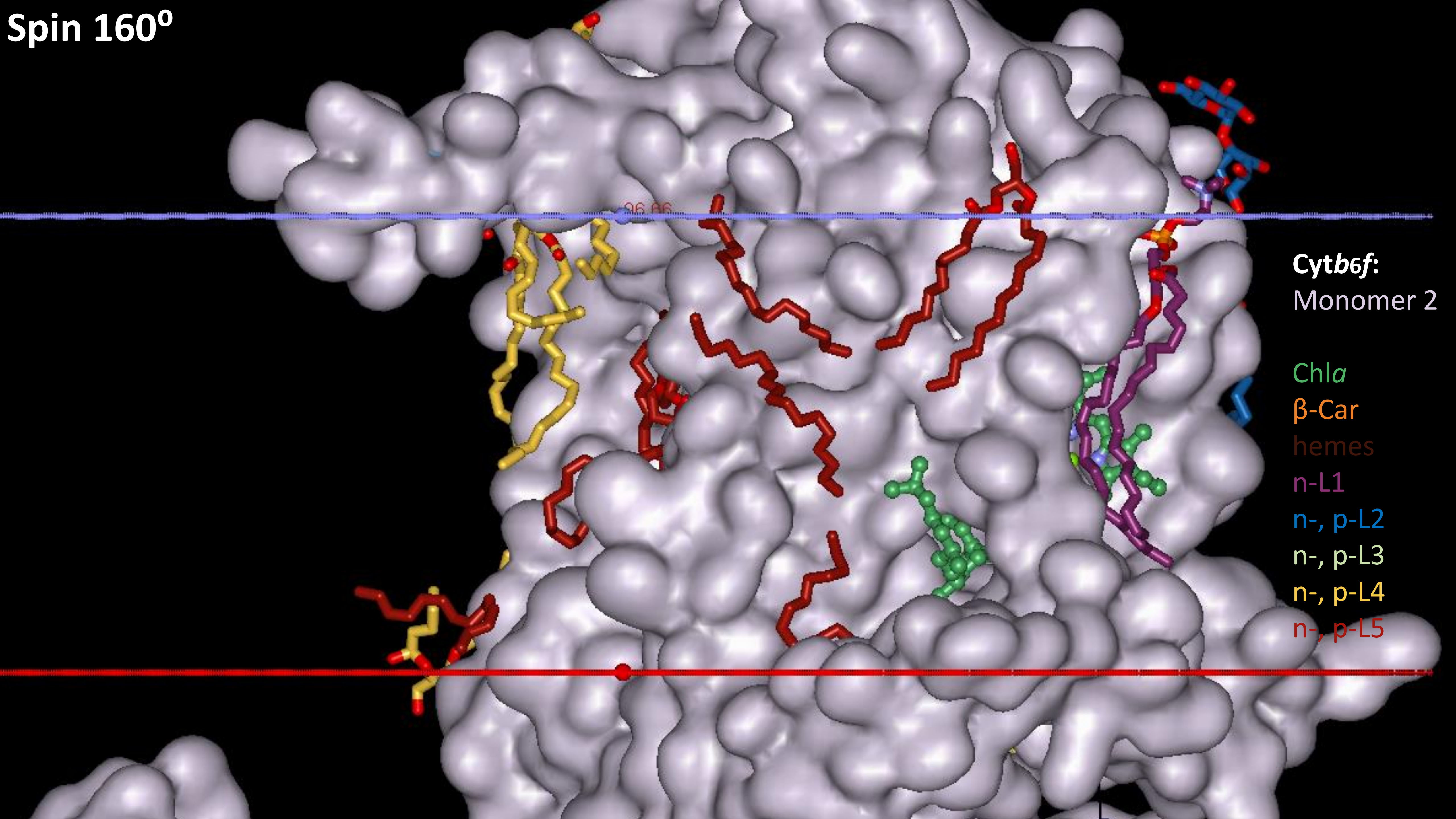
96.66
Cyt*b6f*:
Monomer 2
Chl*a*
β-Car
hemes
n-L1
n-, p-L2
n-, p-L3
n-, p-L4
n-, p-L5

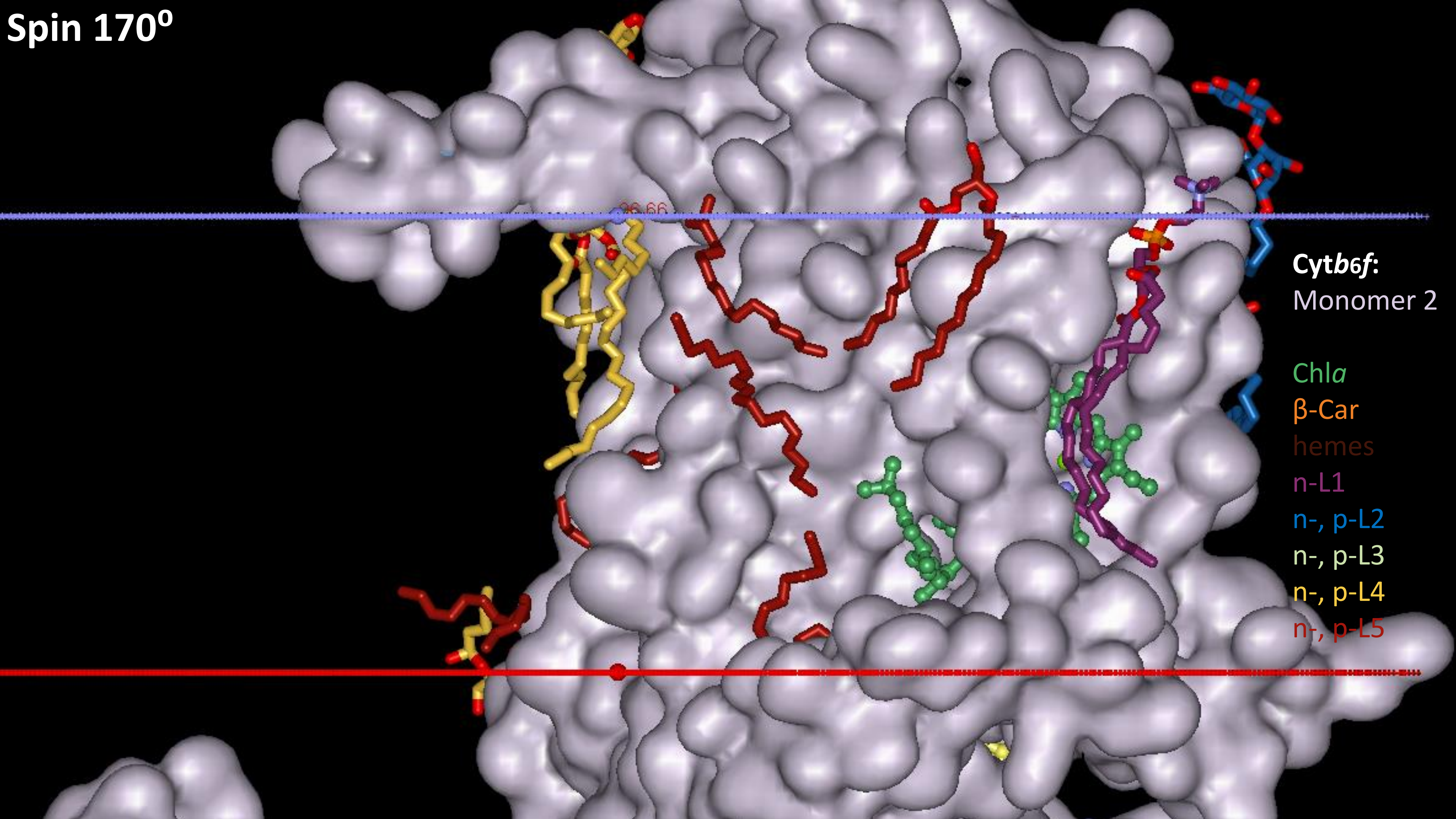
Spin 170°
Cytb6f:
Monomer 2
Chla
β-Car
hemes
n-L1
n-, p-L2
n-, p-L3
n-, p-L4
n-, p-L5

Spin 180°

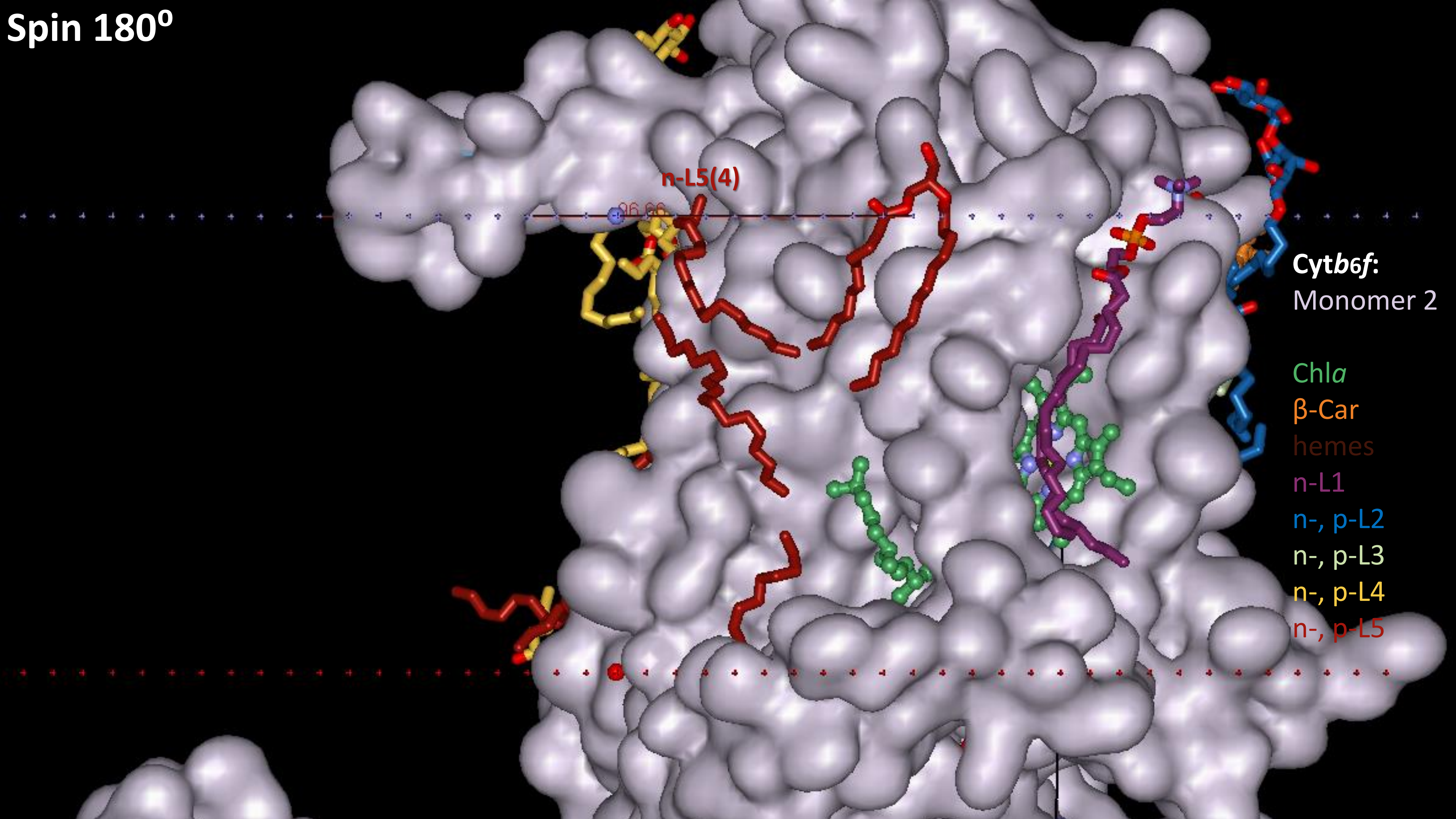
n-L5(4)
Cyt*b6f*:
Monomer 2
Chl*a*
β-Car
hemes
n-L1
n-, p-L2
n-, p-L3
n-, p-L4
n-, p-L5

Spin 180º

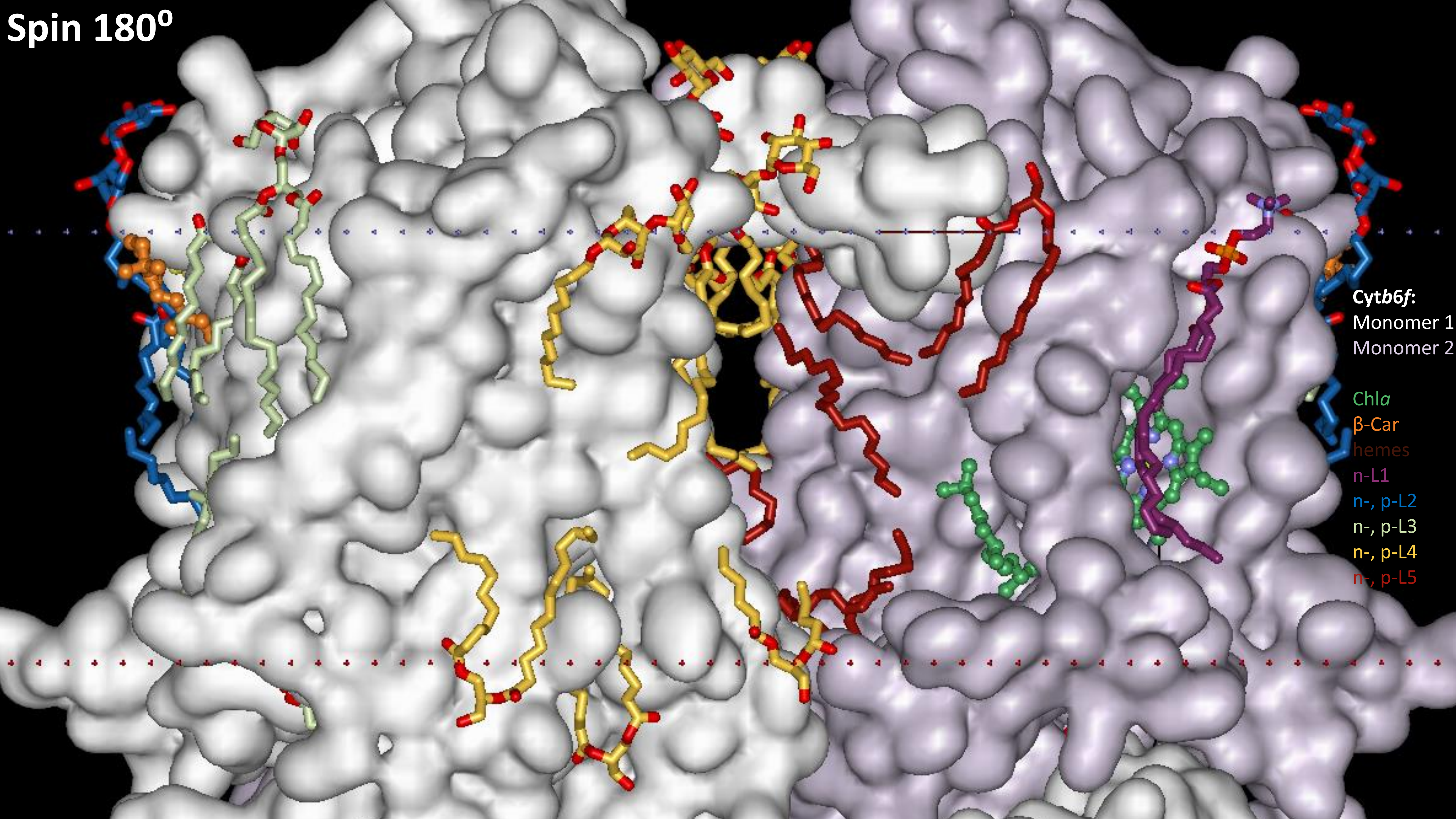
Cytb6f:
Monomer 1
Monomer 2
Chla
β-Car
hemes
n-L1
n-, p-L2
n-, p-L3
n-, p-L4
n-, p-L5
