# Supplementary material for: X-Ray Crystal and Cryo-Electron Microscopy Structure Analysis Unravels How the Unique Thylakoid Lipid Composition Is Utilized by Cytochrome b6f for Driving Reversible Proteins’ Reorganization During State Transitions
Source: Membranes (Basel). 2025 May 8;15(5):143. doi: 10.3390/membranes15050143 (PMC12112970; doi:10.3390/membranes15050143)
Supplement: Supplementary file 1 [file membranes-15-00143-s001.zip › 3-Suppl_Images-2.pdf]

Supplementary Material for

X-ray crystal and cryo-EM structure analysis unravel how the unique thylakoid lipid composition is utilized by cytochrome b_6f for driving reversible proteins' reorganization during state transitions

Radka Vladkova (rvladkova@bio21.bas.bg)

Supplementary Package of Images – Part 2

(Related to Figure 6 and visualizes in detail the occupation and dynamics of each lipid binding site in the X-ray crystal structures of cytochrome b_6f during the induction phase of the transition to state 2 and to state 1)

Transition to state 2 (11 structures)

Group - Slides:

- L1 - 2- 8
- L2 - 9-14
- L3 - 15-21
- L4 - 22-40
- L5 - 41-61

Transition to state 1 (8 structures)

Group - Slides:

- L4 - 62-79
- L5 - 80-95

L1-site - β-side of the Chl*a* macrocycle plane

p-side view

- The n-L1 lipid always contacts the β-side of the Chlorophyll *a* (Chl*a*)

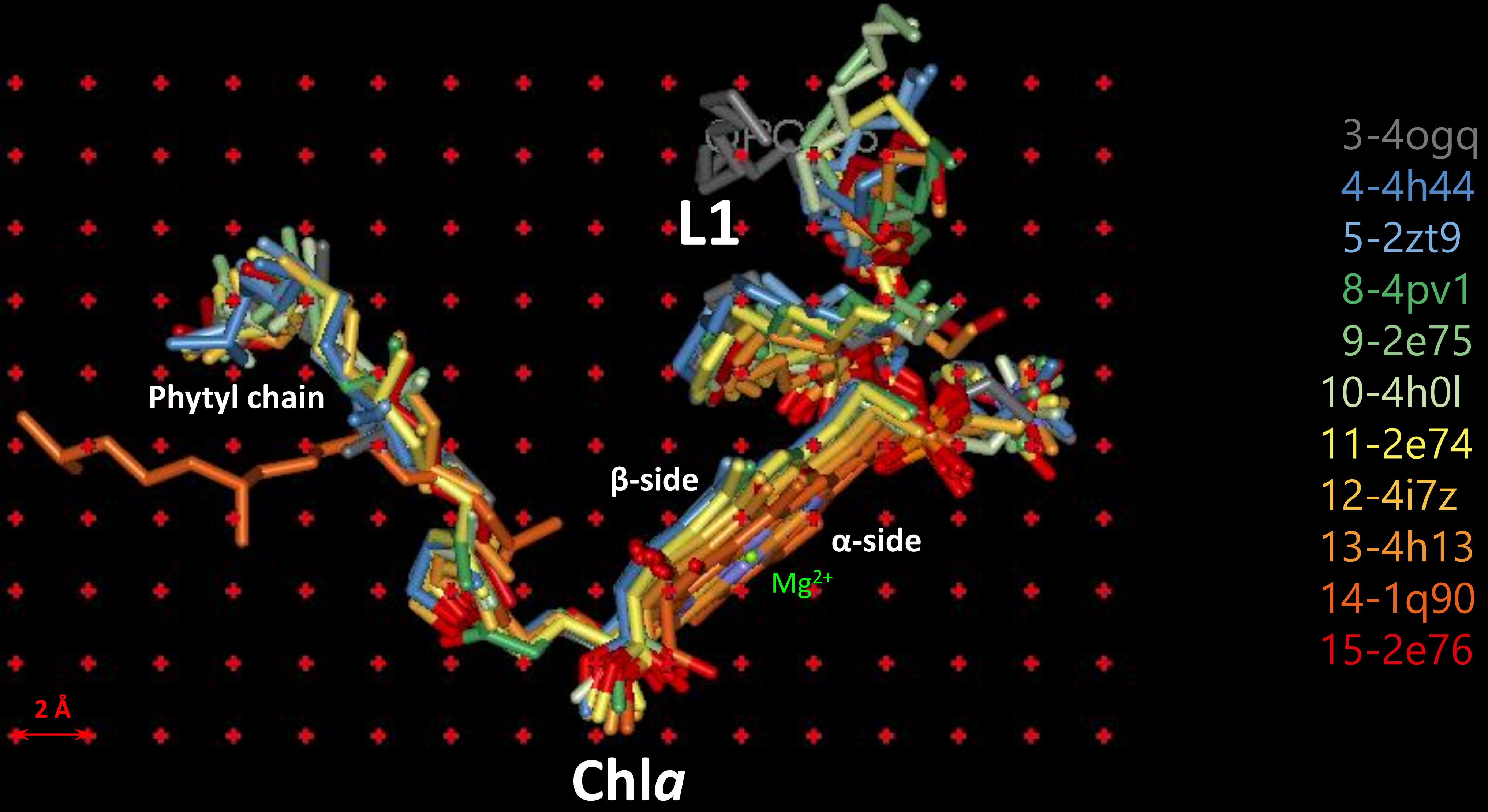

L1-site - β-side of the Chl*a* macrocycle plane

n-side view

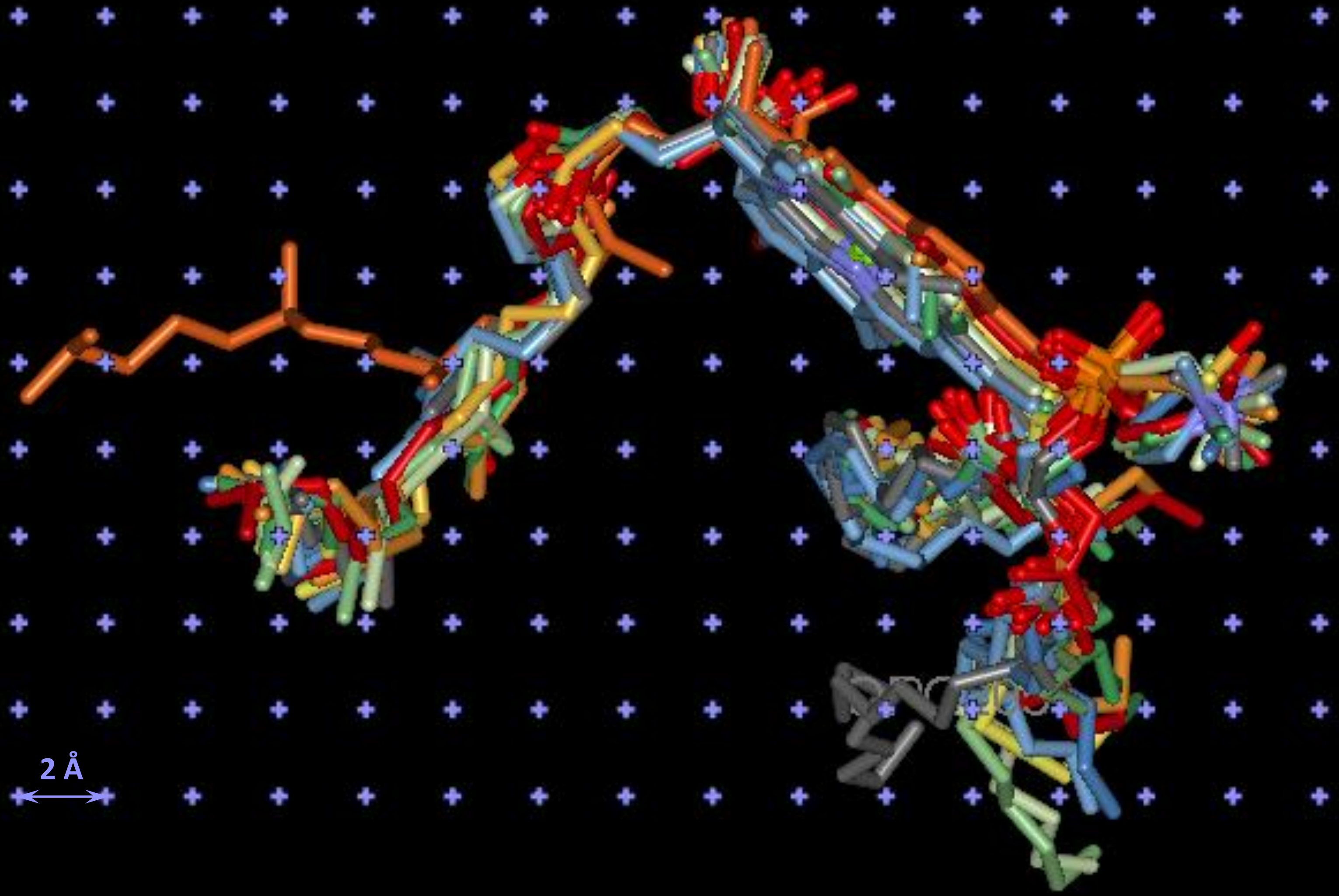

3-4ogq
4-4h44
5-2zt9
8-4pv1
9-2e75
10-4h0l
11-2e74
12-4i7z
13-4h13
14-1q90
15-2e76

L1-site front view

Spin 0º

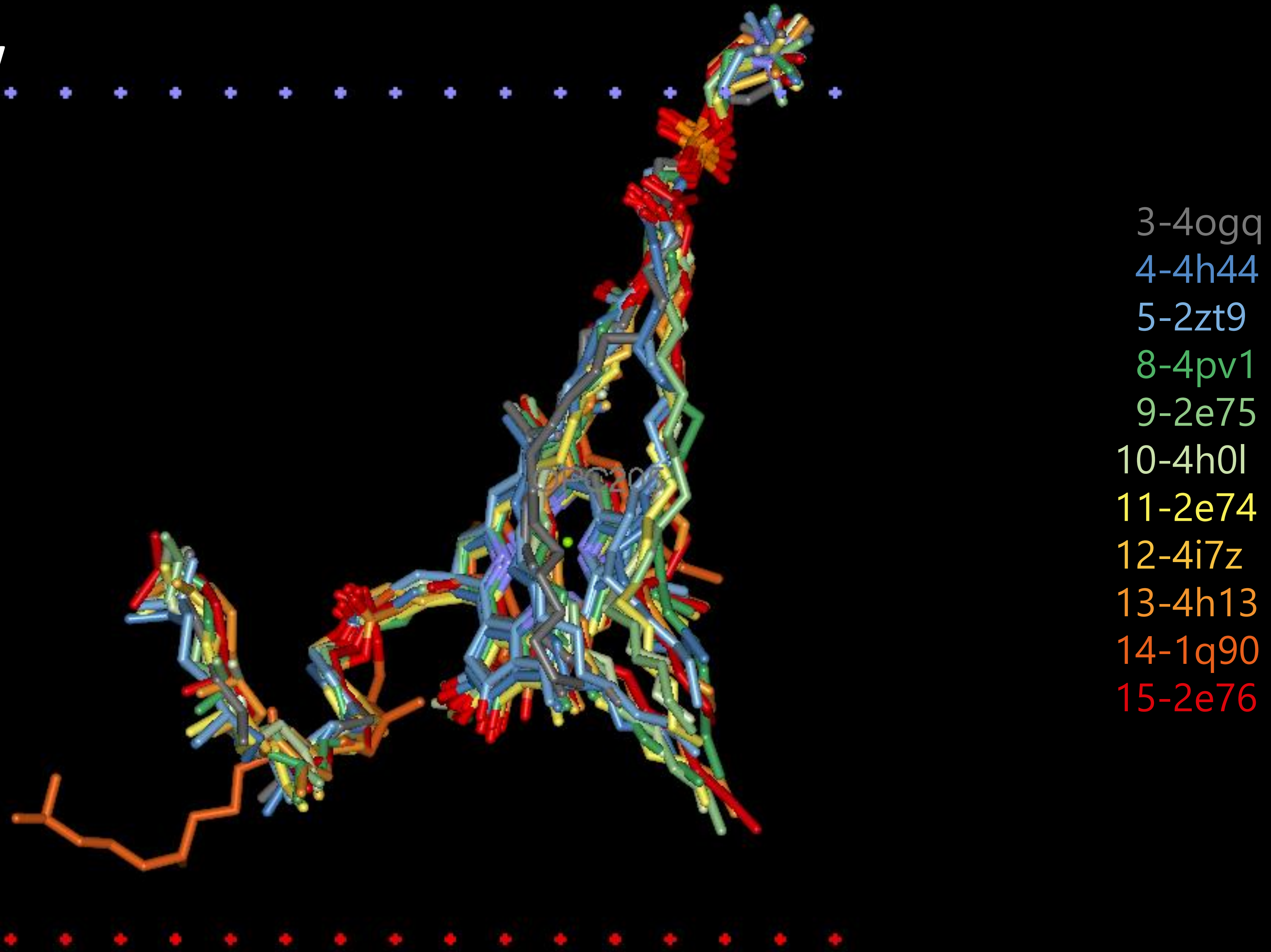

L1-site front view

Spin 50º

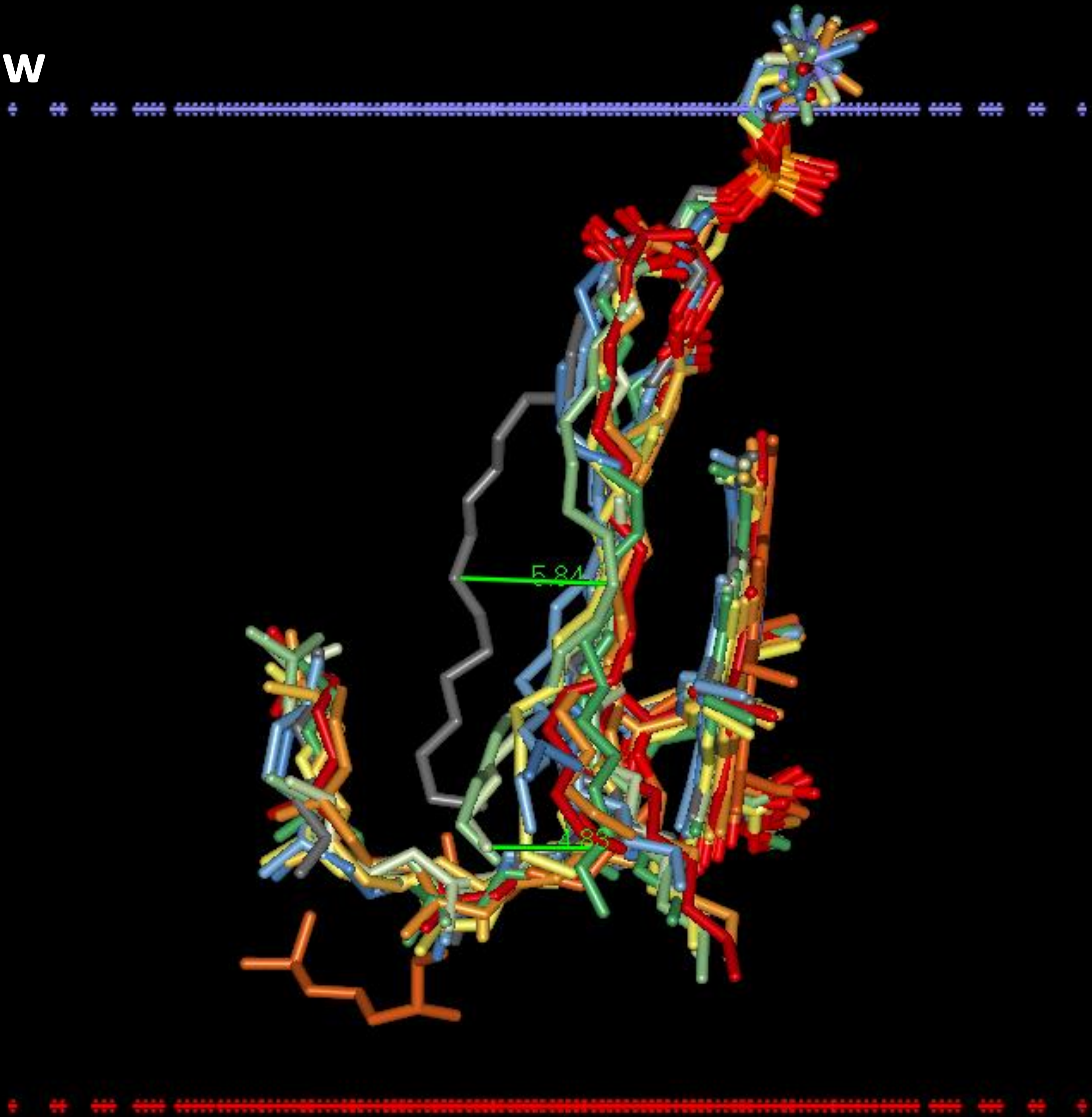

L1-site front view

Spin 230°

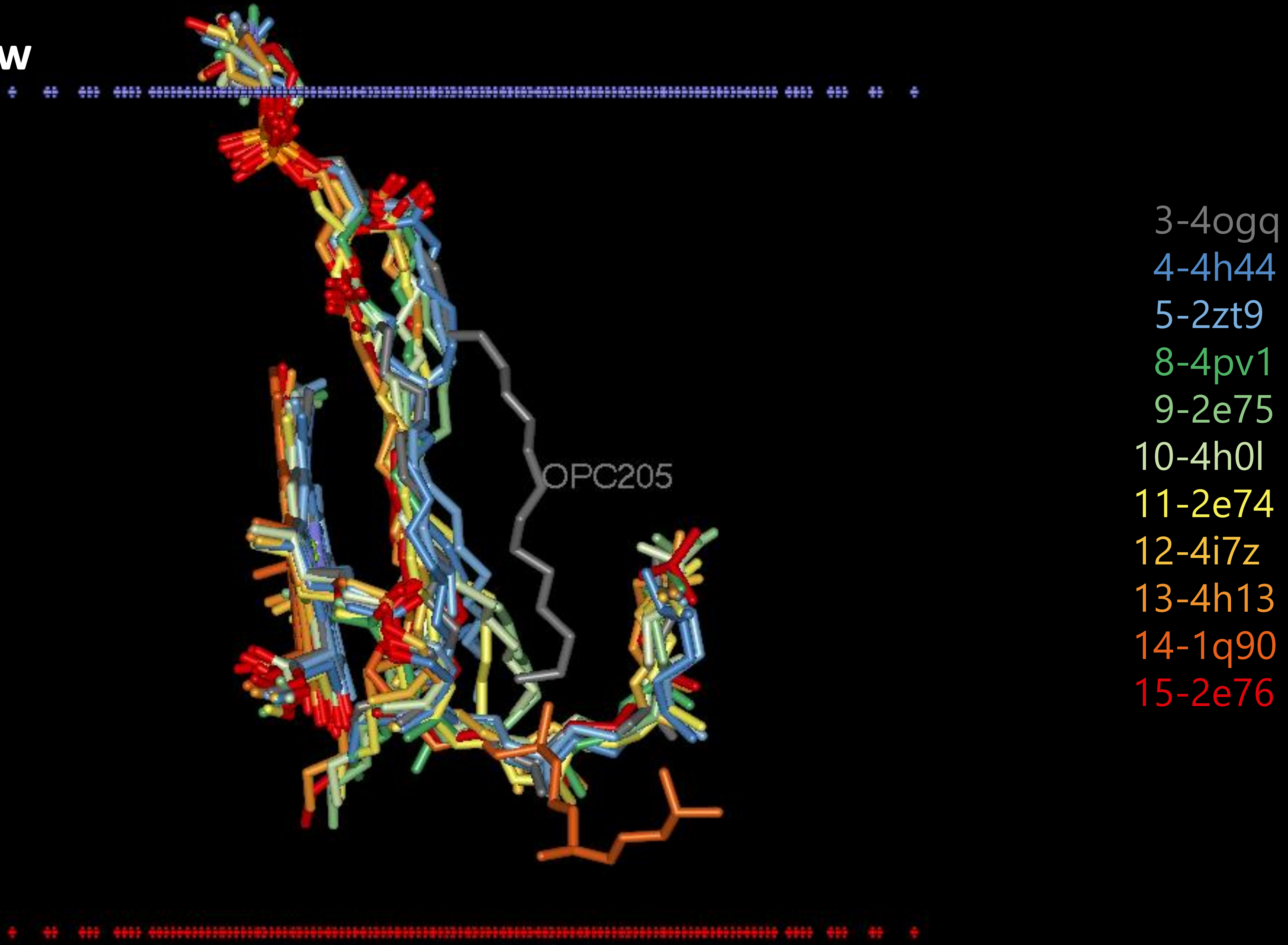

L1-site front view

Spin 80°

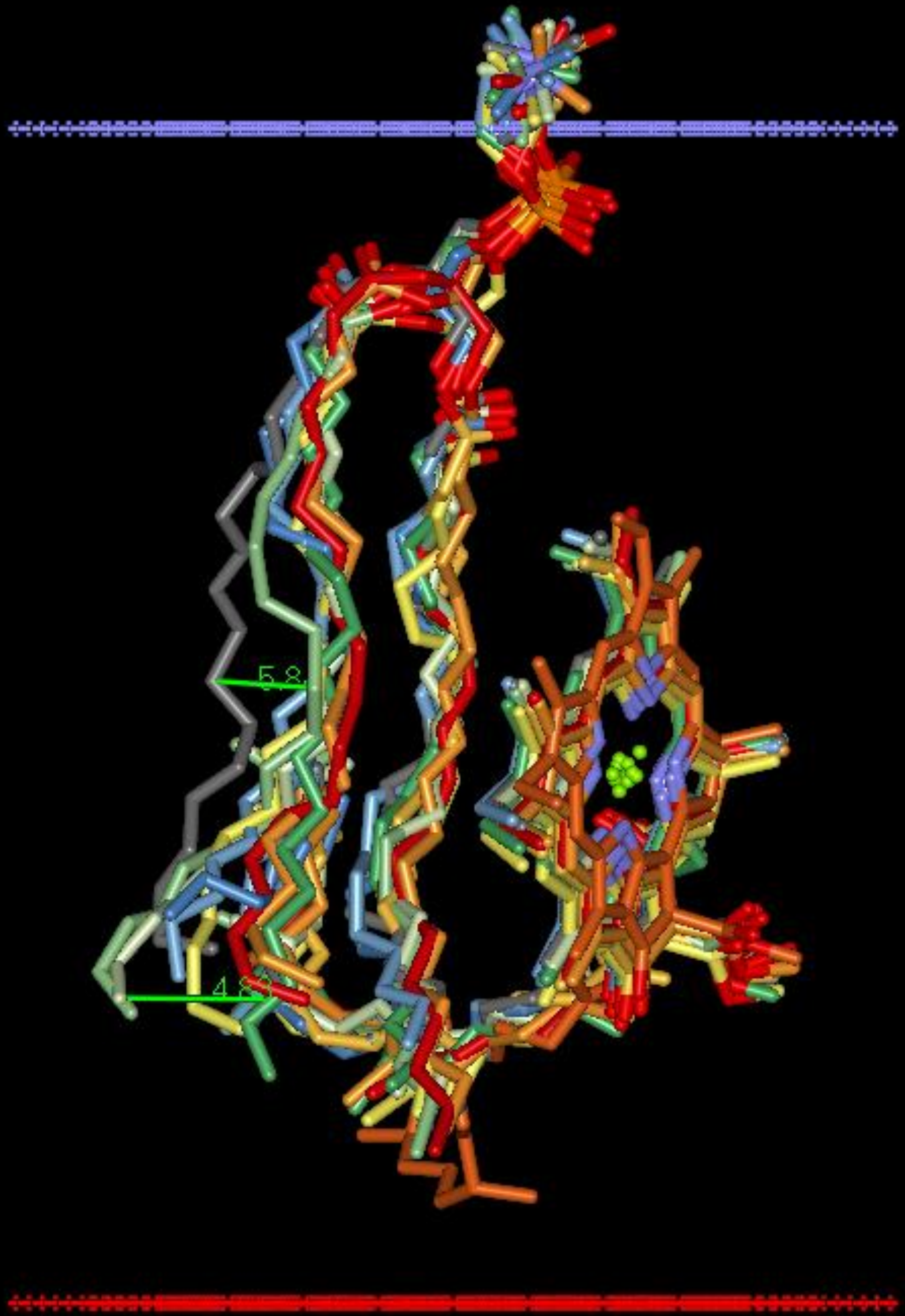

L1-site front view

Spin 90°

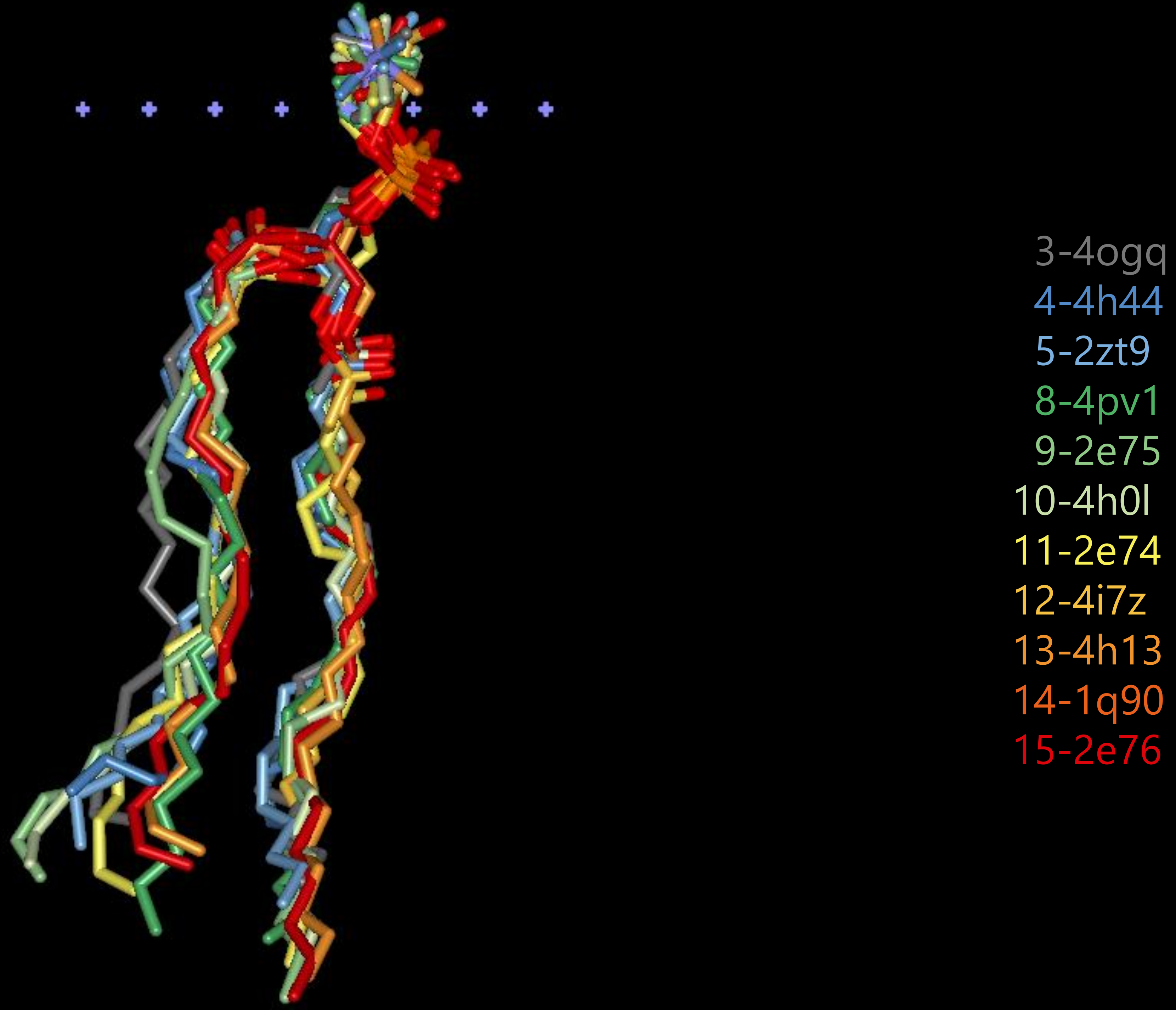

L2-sites (Phe124) p-side view

- n-L2(1-2) sites contact Phe/Tyr124 of *fg*-loop and are occupied only in 4ogq by one DAG and one detergent;
- p-L2 site always contacts the α-side of Chl*a* (O1D) and can also contact the Mg atom.

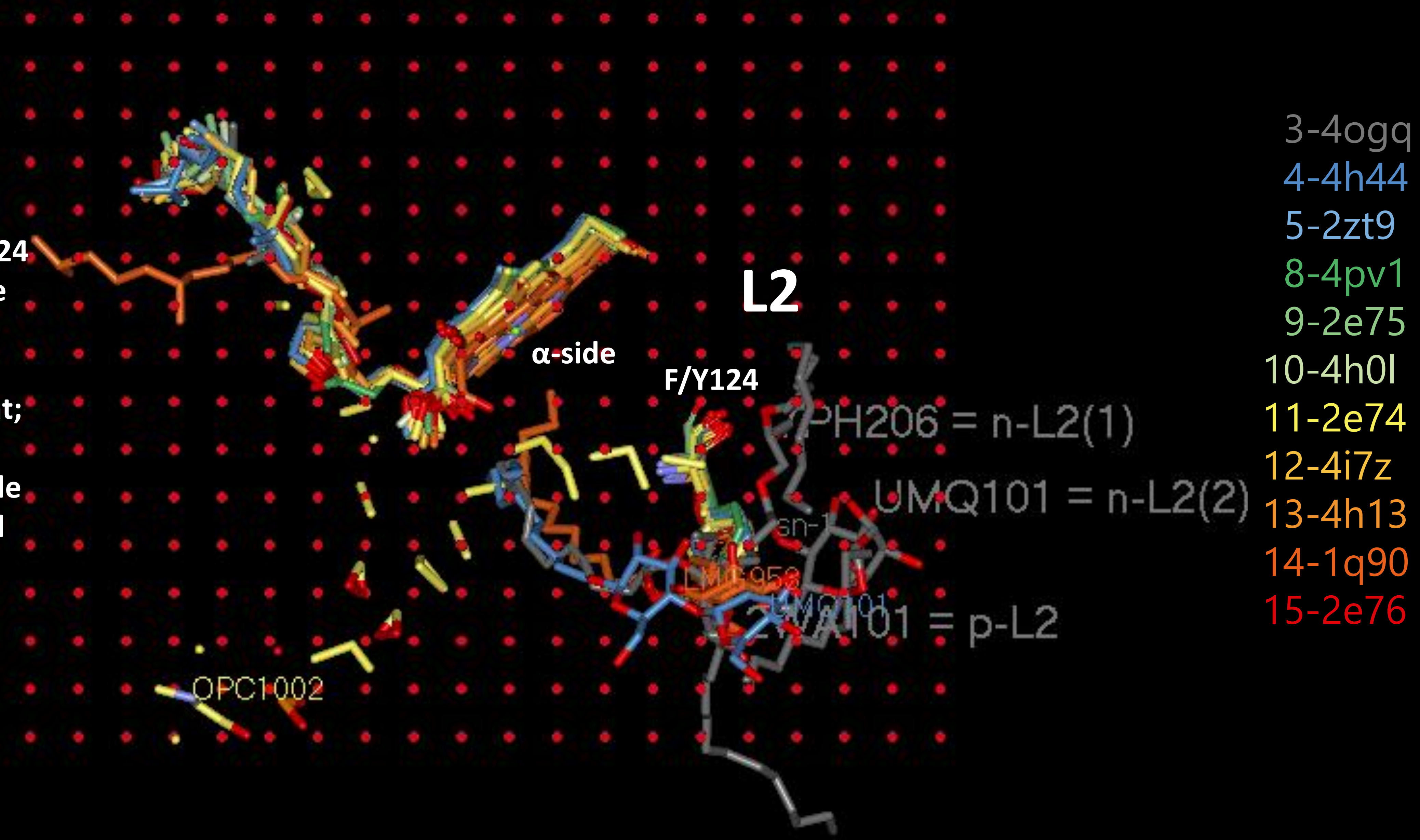

L2-sites n-side view

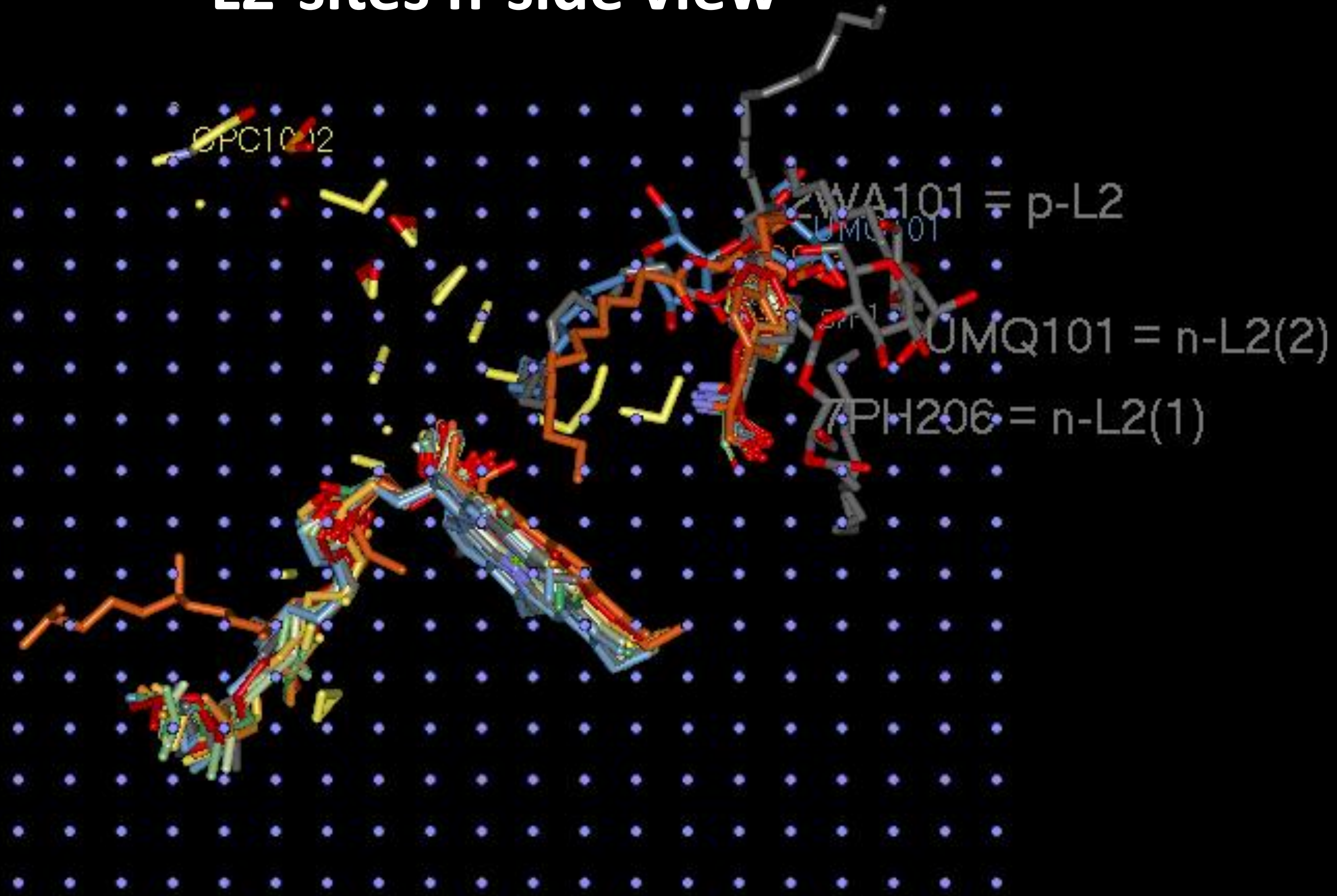

3-4ogq
4-4h44
5-2zt9
8-4pv1
9-2e75
10-4h0l
11-2e74
12-4i7z
13-4h13
14-1q90
15-2e76

L2-sites front view

Spin 0º

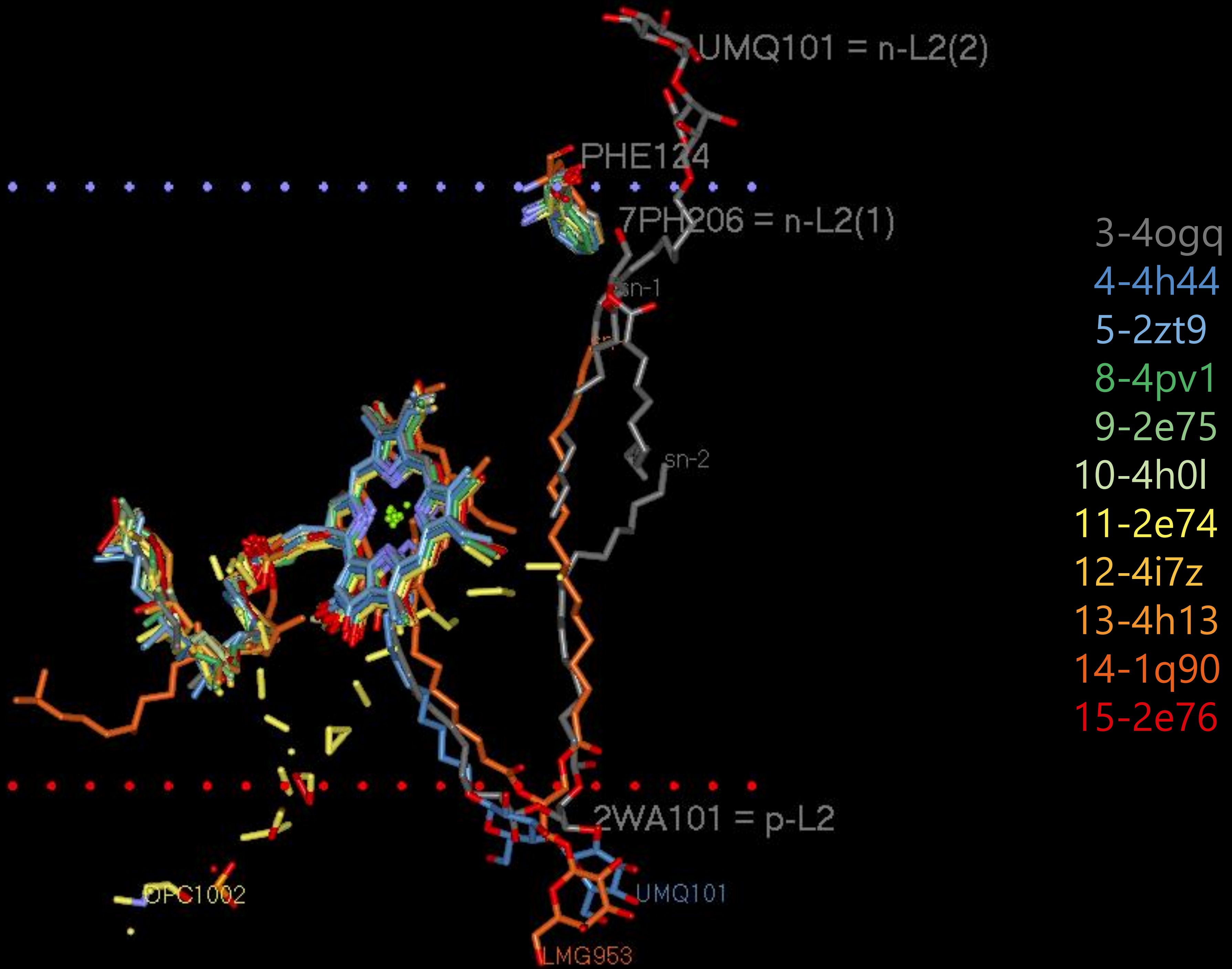

L2-sites front view

Spin 50°

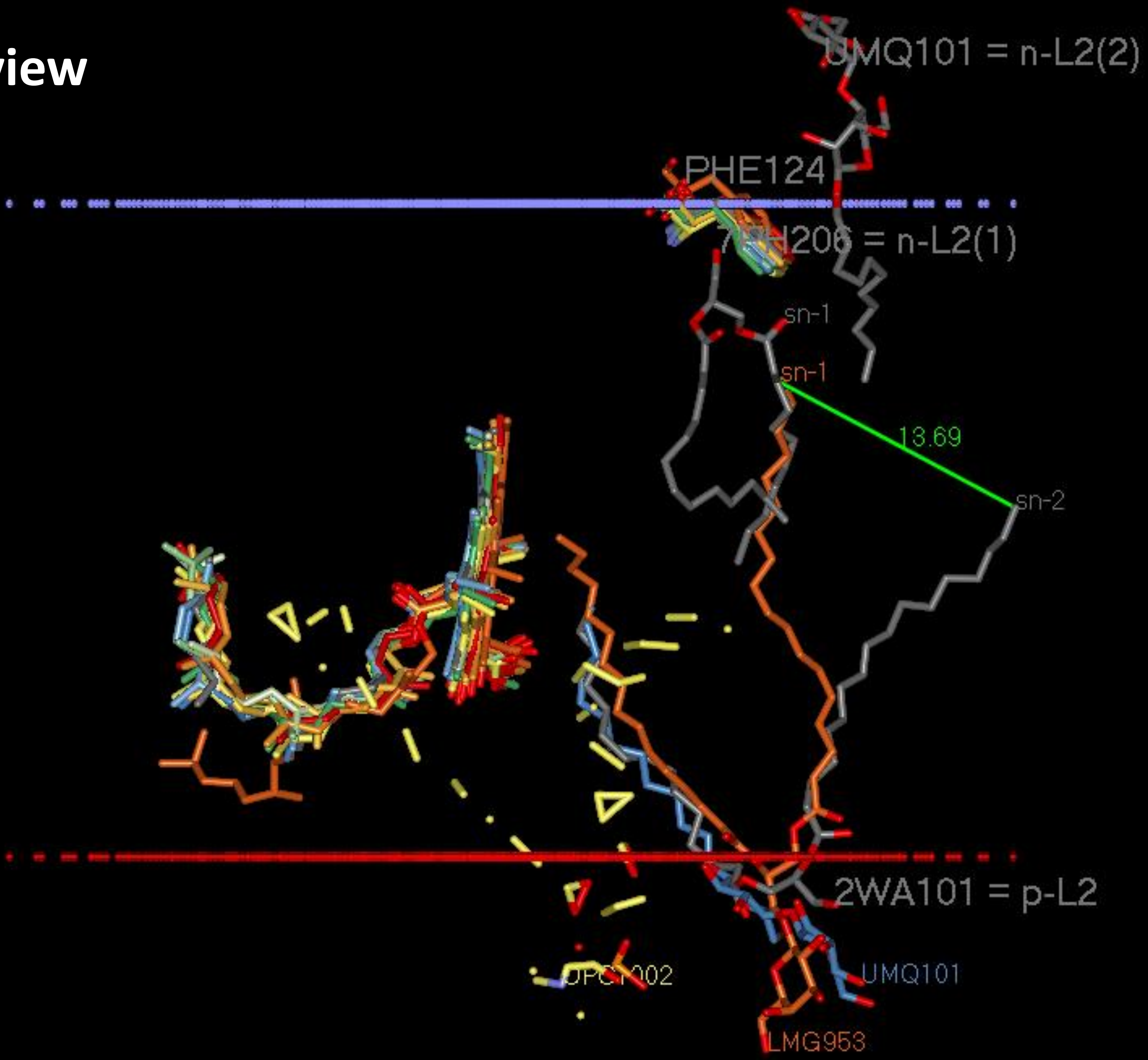

3-4ogq
4-4h44
5-2zt9
8-4pv1
9-2e75
10-4h0l
11-2e74
12-4i7z
13-4h13
14-1q90
15-2e76

L2-sites front view

Spin 90°

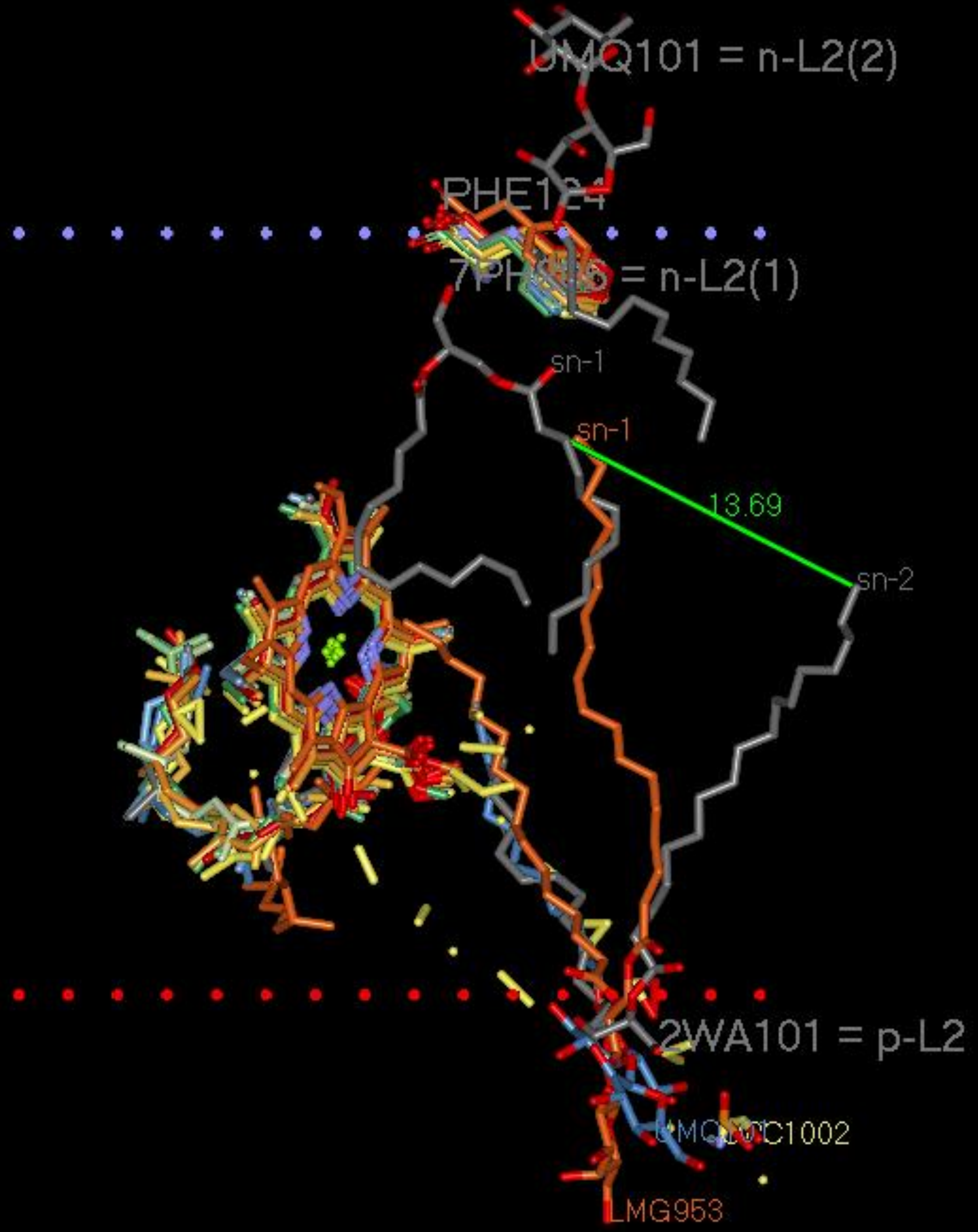

3-4ogq
4-4h44
5-2zt9
8-4pv1
9-2e75
10-4h0l
11-2e74
12-4i7z
13-4h13
14-1q90
15-2e76

L2-sites front view

Spin 180°

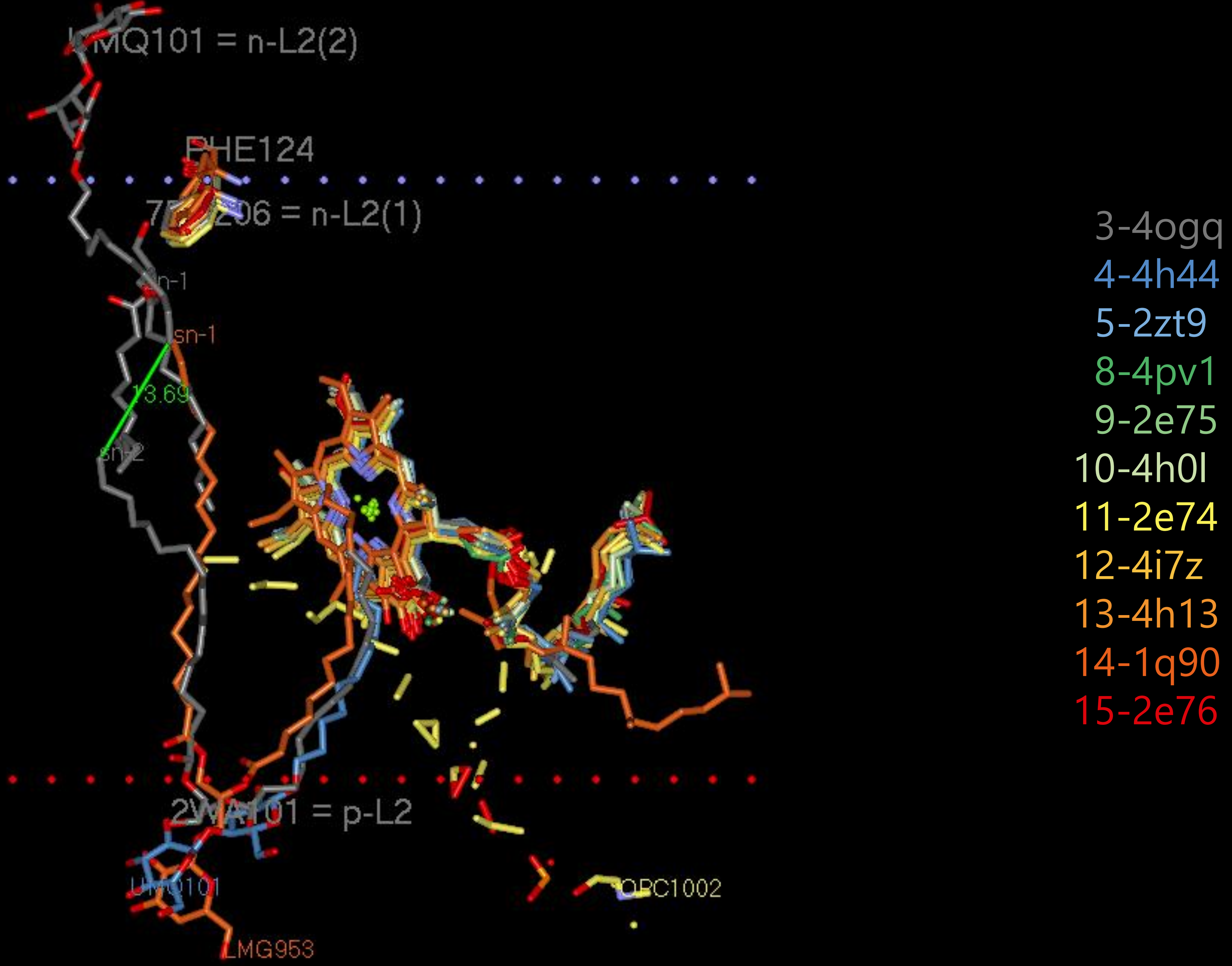

L3-sites (β-Car) p-side view

- p-L3(1) chain always contacts the β-Car ring (distance below 4 Å) that is deep buried inside the hydrophobic membrane core of the protein, except in 2e75 (distance 4.6 Å);
- n-L3(1) contacts β-Car only in 4ogq but is modelled by non-contacting octane in others three structures;
- p-L3(2) and n-L3(2) are visible only in 4ogq.

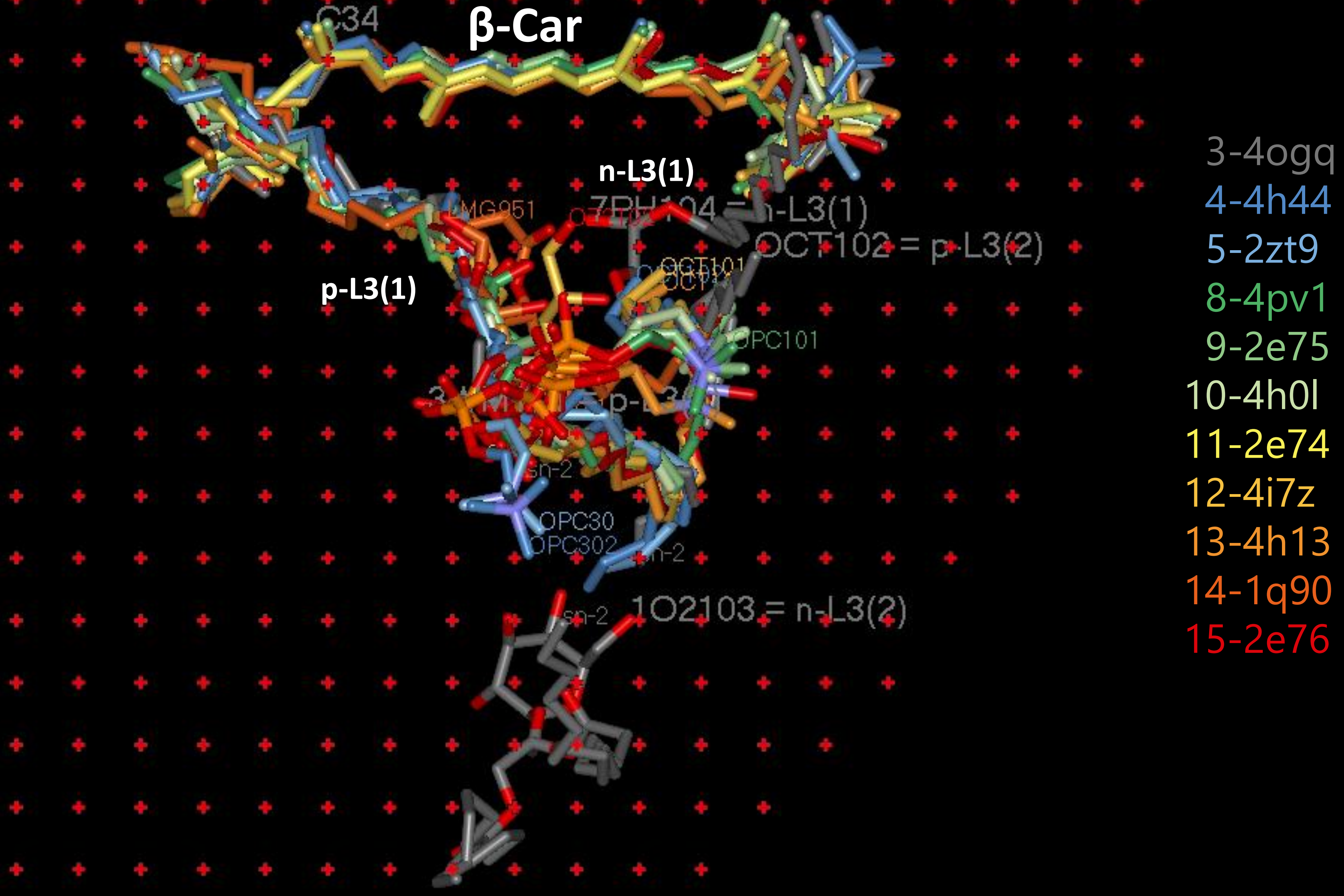

L3-sites n-side view

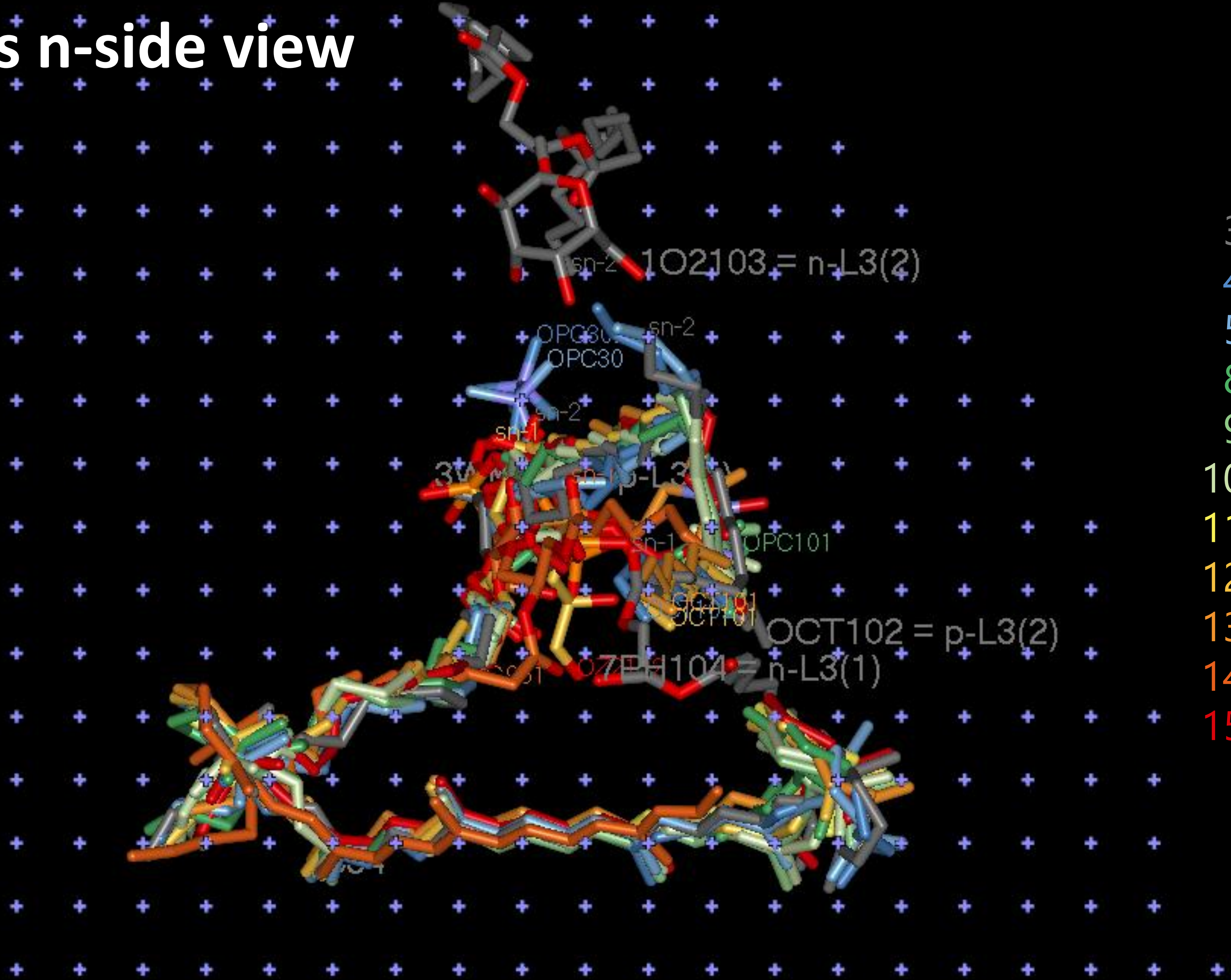

3-4ogq
4-4h44
5-2zt9
8-4pv1
9-2e75
10-4h0l
11-2e74
12-4i7z
13-4h13
14-1q90
15-2e76

L3-sites front view

Spin 0°

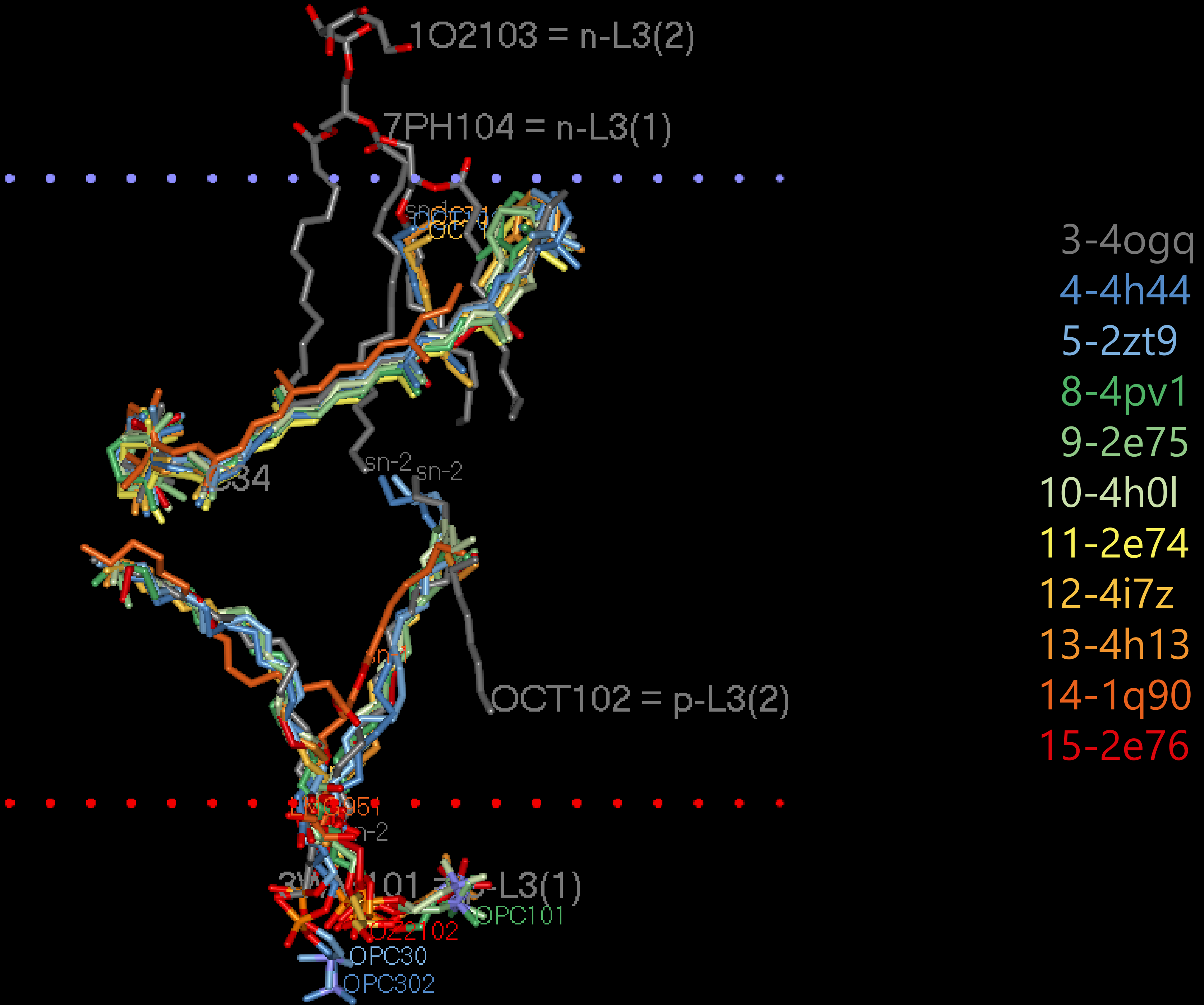

L3-sites front view

Spin 50°

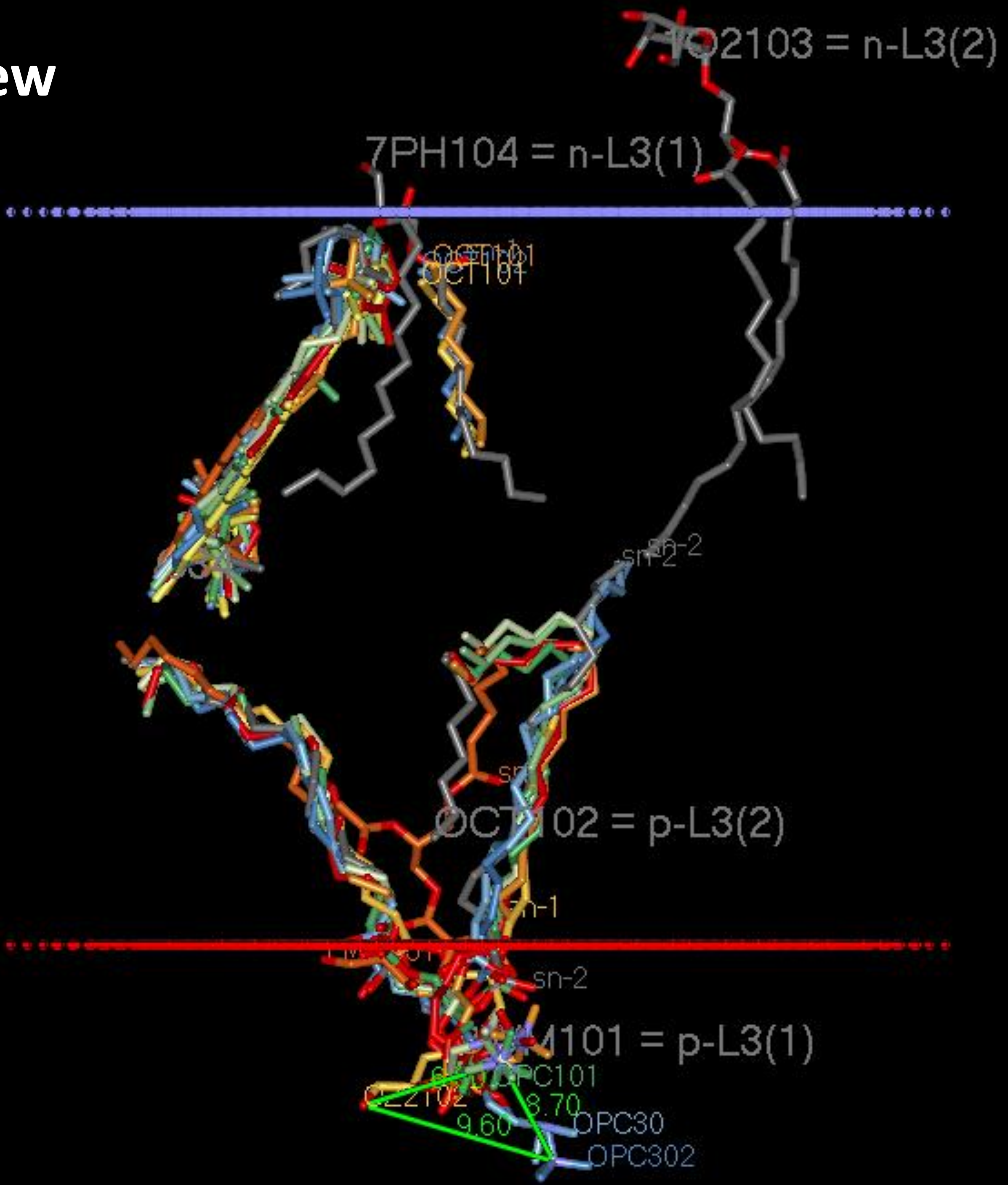

3-4ogq
4-4h44
5-2zt9
8-4pv1
9-2e75
10-4h0l
11-2e74
12-4i7z
13-4h13
14-1q90
15-2e76

L3-sites front view

Spin 90°

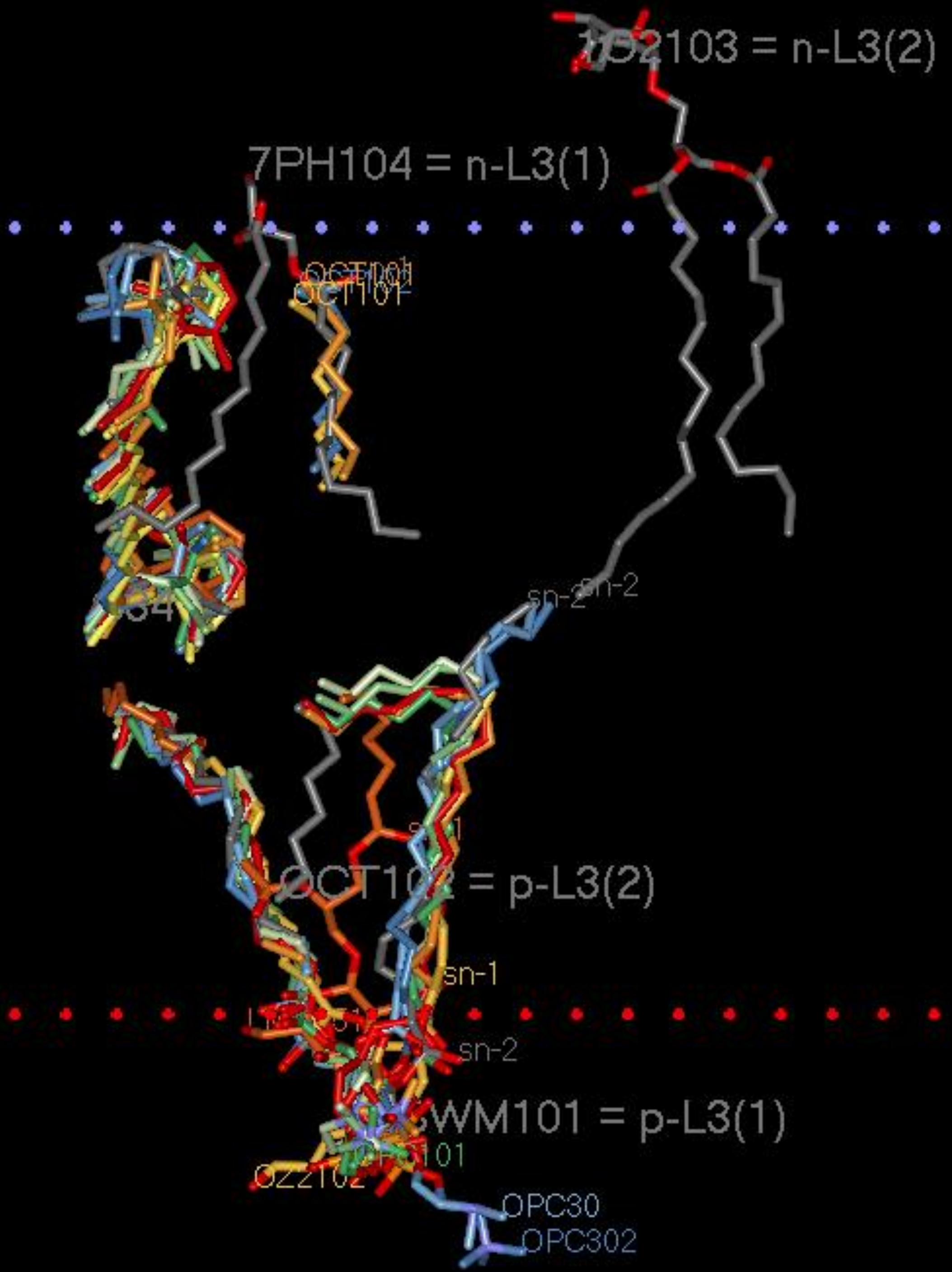

3-4ogq
4-4h44
5-2zt9
8-4pv1
9-2e75
10-4h0l
11-2e74
12-4i7z
13-4h13
14-1q90
15-2e76

L3-sites front view

Spin 180°

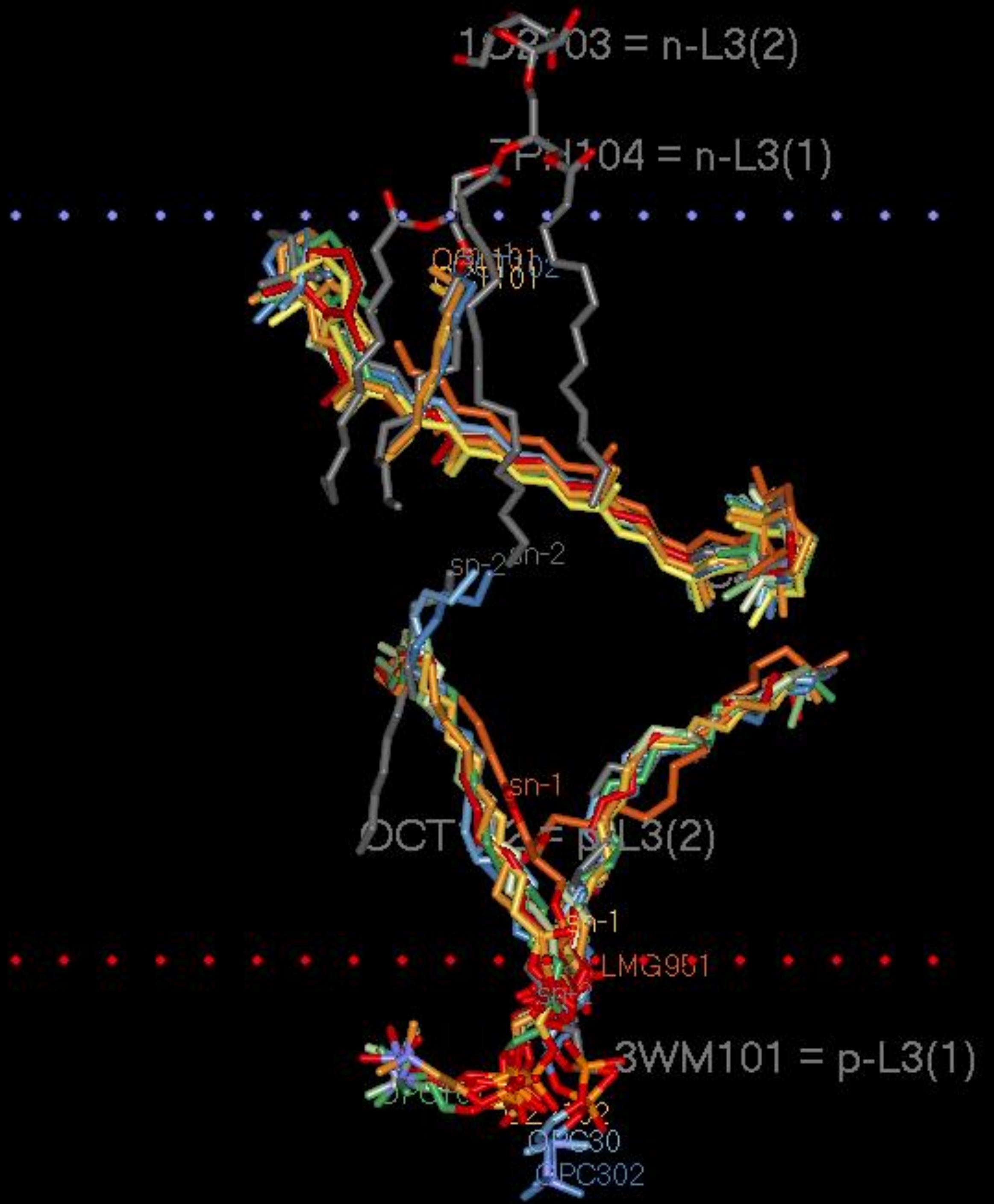

3-4ogq
4-4h44
5-2zt9
8-4pv1
9-2e75
10-4h0l
11-2e74
12-4i7z
13-4h13
14-1q90
15-2e76

L3-sites front view

Spin 270°

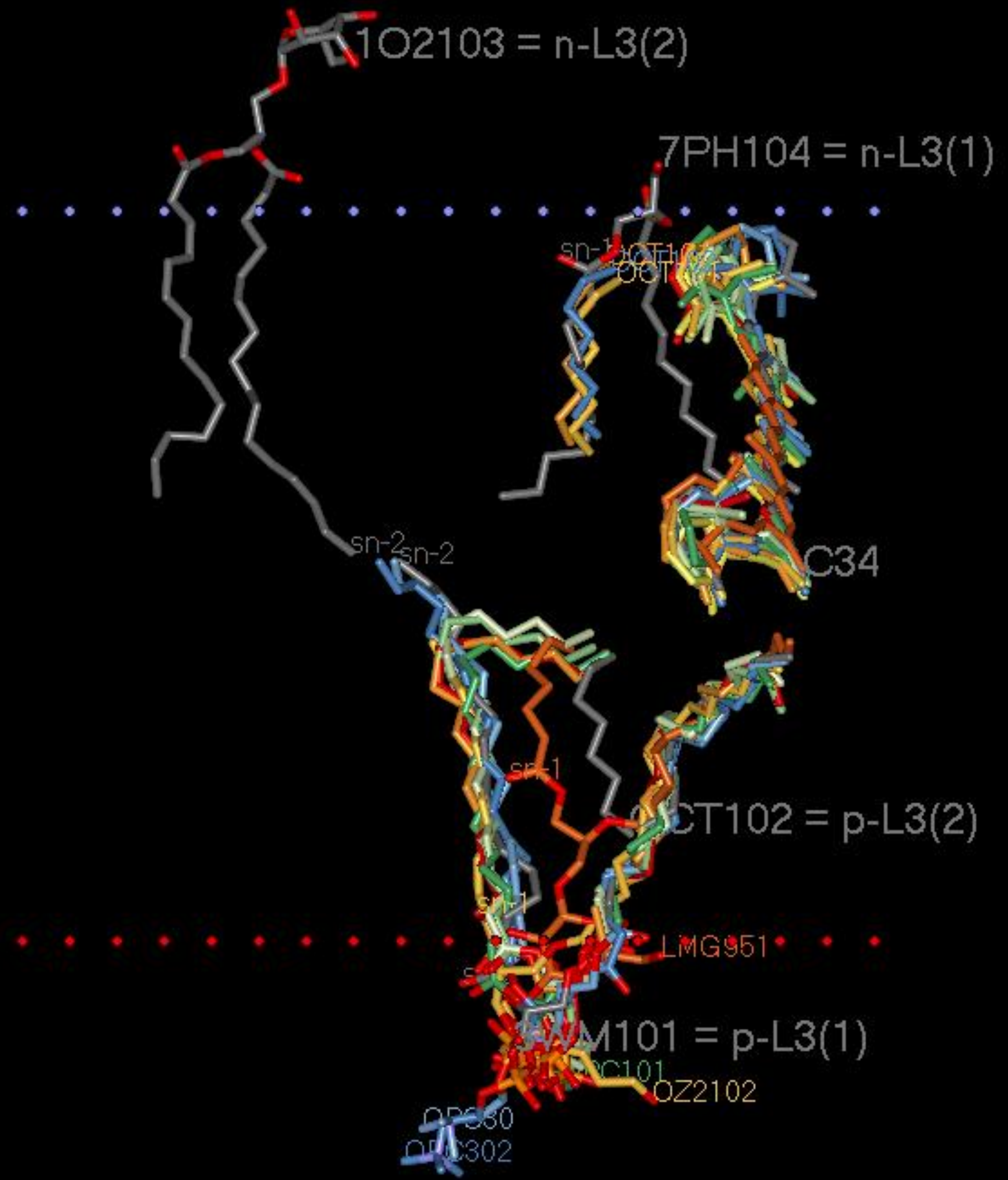

3-4ogq
4-4h44
5-2zt9
8-4pv1
9-2e75
10-4h0l
11-2e74
12-4i7z
13-4h13
14-1q90
15-2e76

L4-sites (SQDG) p-side view

- Here are grouped seven binding sites; four at n-side and three at p-side.
- The n-L4(4), p-L4(3) and p-L4(4) are visible only in 3-4ogq.

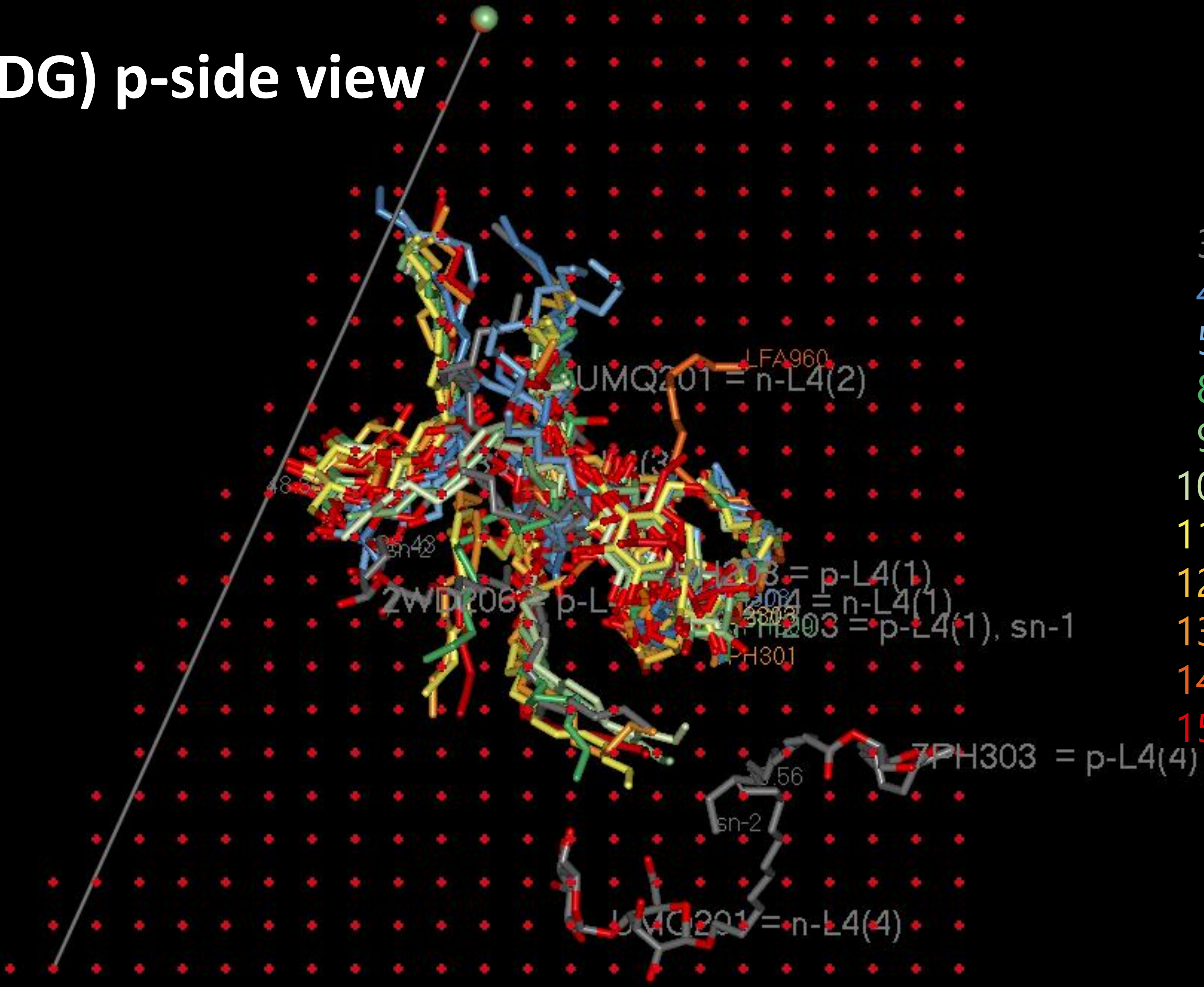

3-4ogq
4-4h44
5-2zt9
8-4pv1
9-2e75
10-4h0l
11-2e74
12-4i7z
13-4h13
14-1q90
15-2e76

L4-sites n-side view

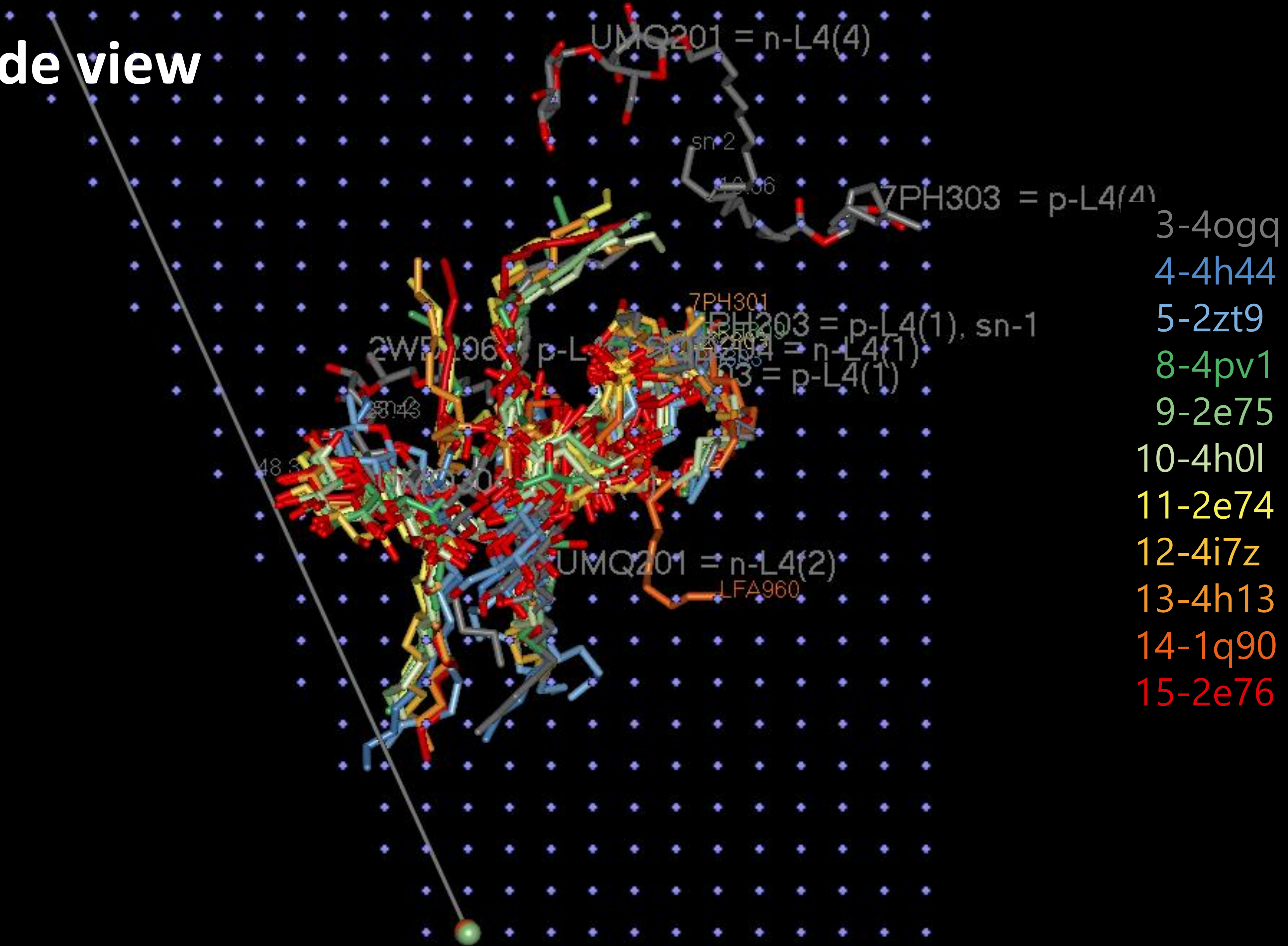

L4-sites front view

Spin 0º

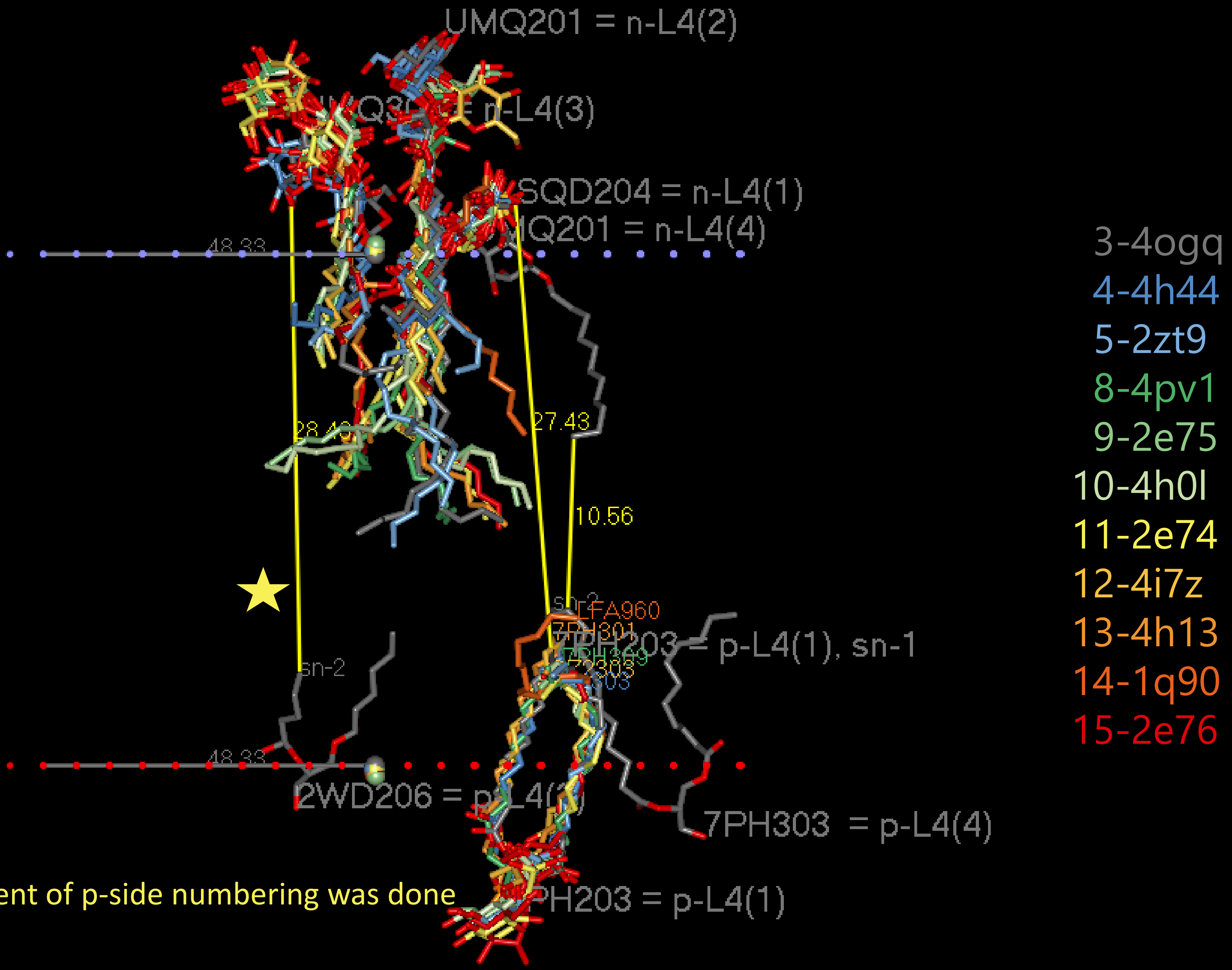

★ Example of how the assignment of p-side numbering was done

L4-sites front view

Spin 50º

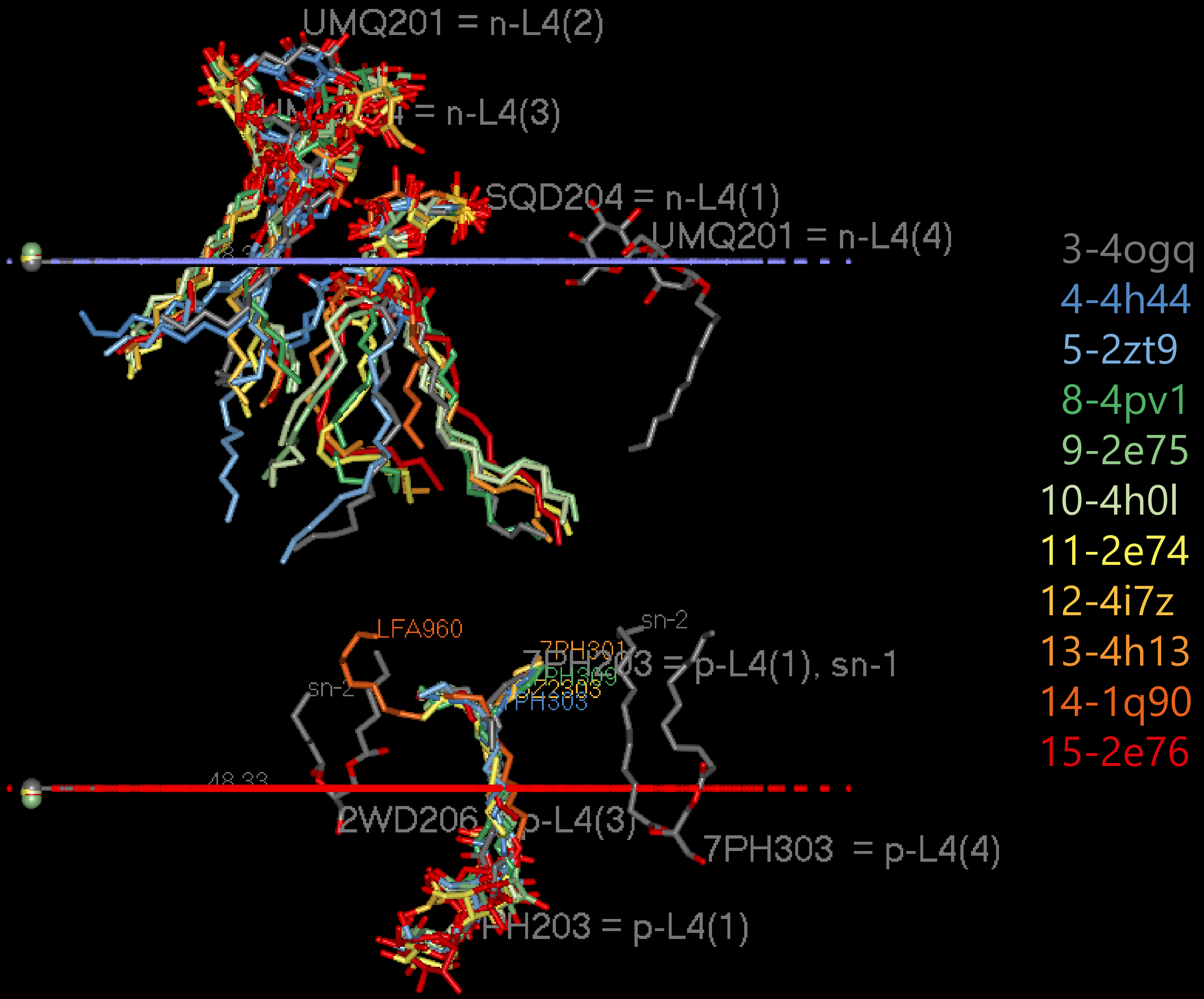

L4-sites front view

Spin 90°

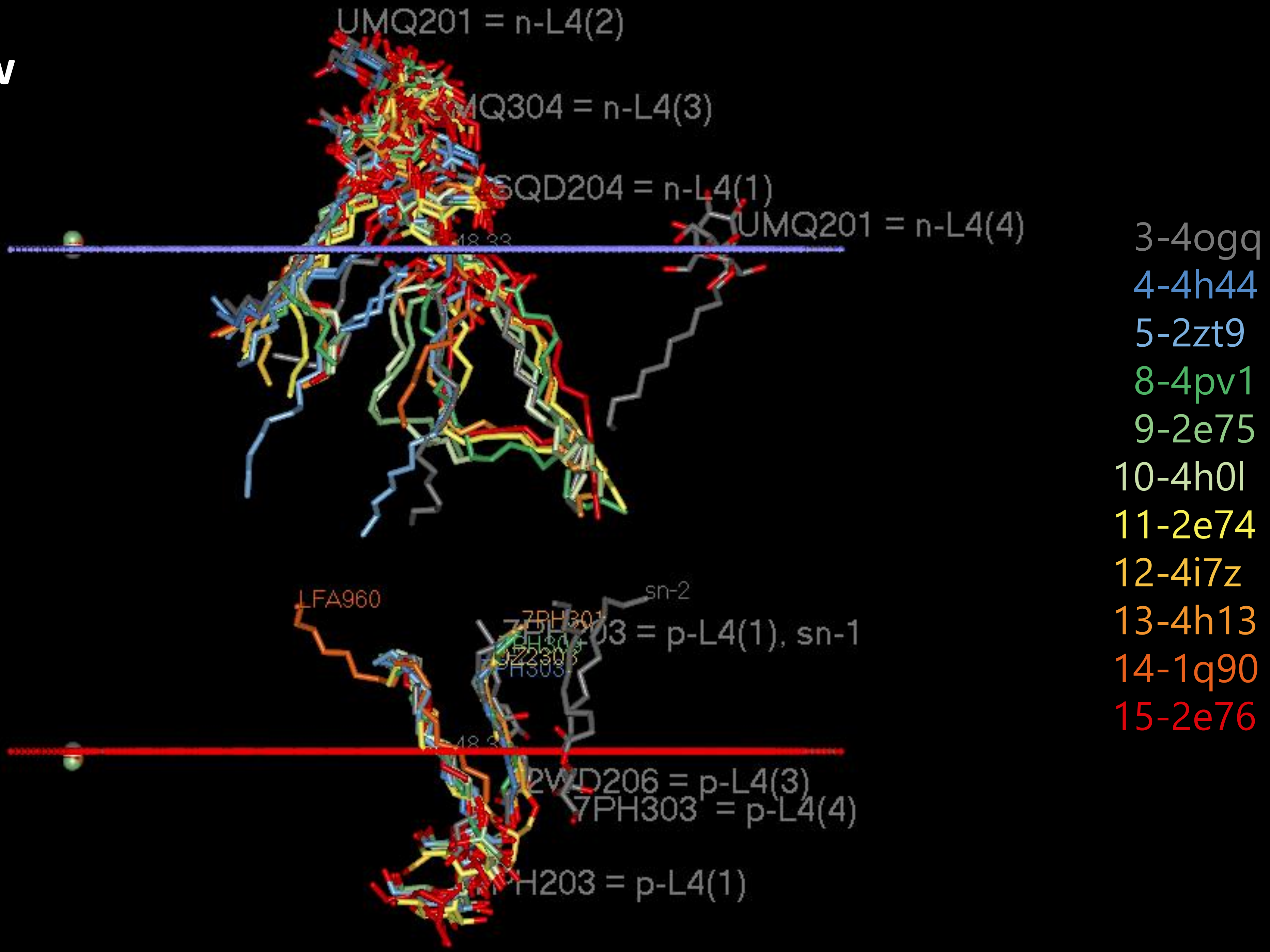

L4-sites front view

Spin 180°

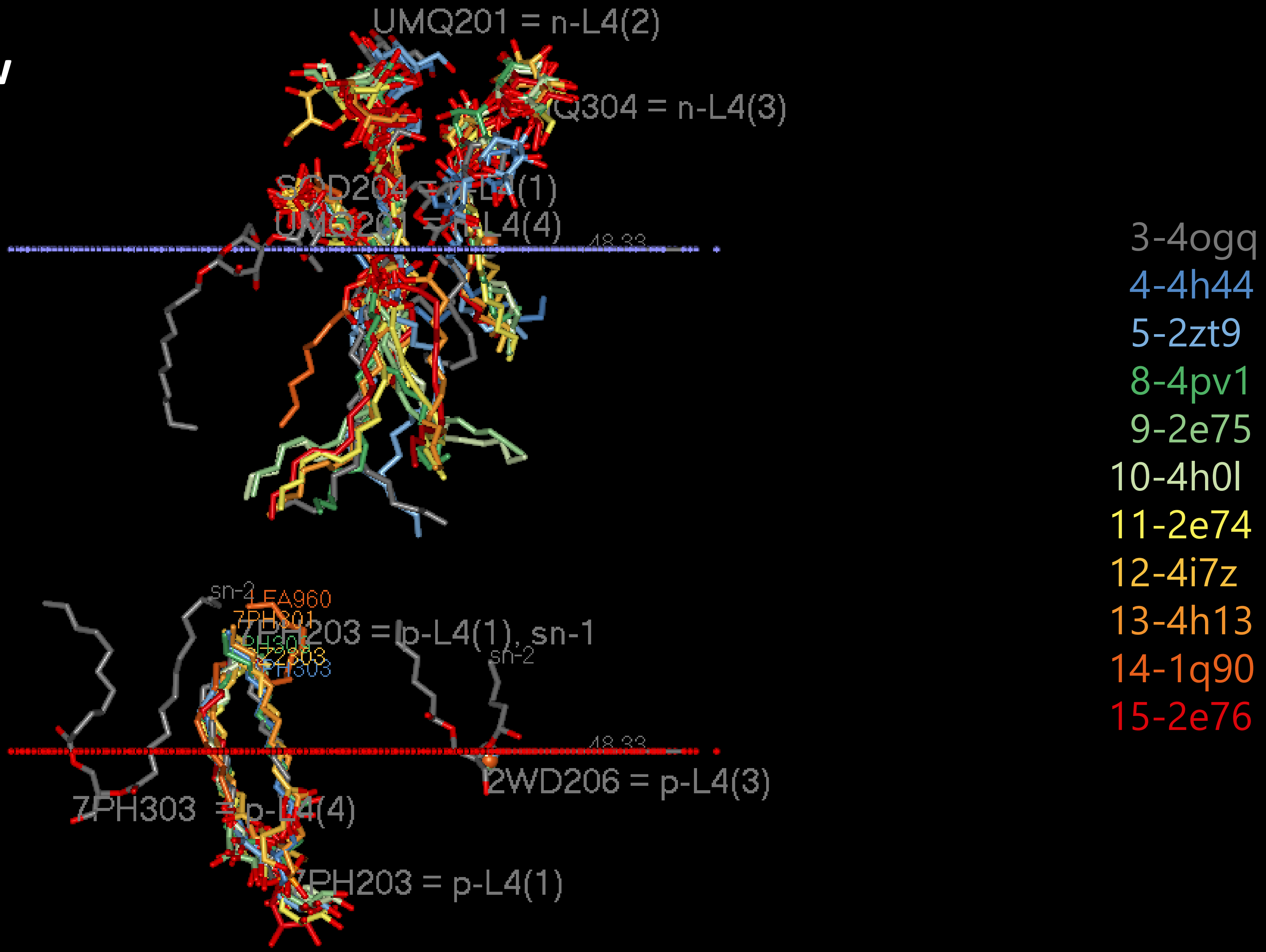

L4-sites front view

Spin 210°

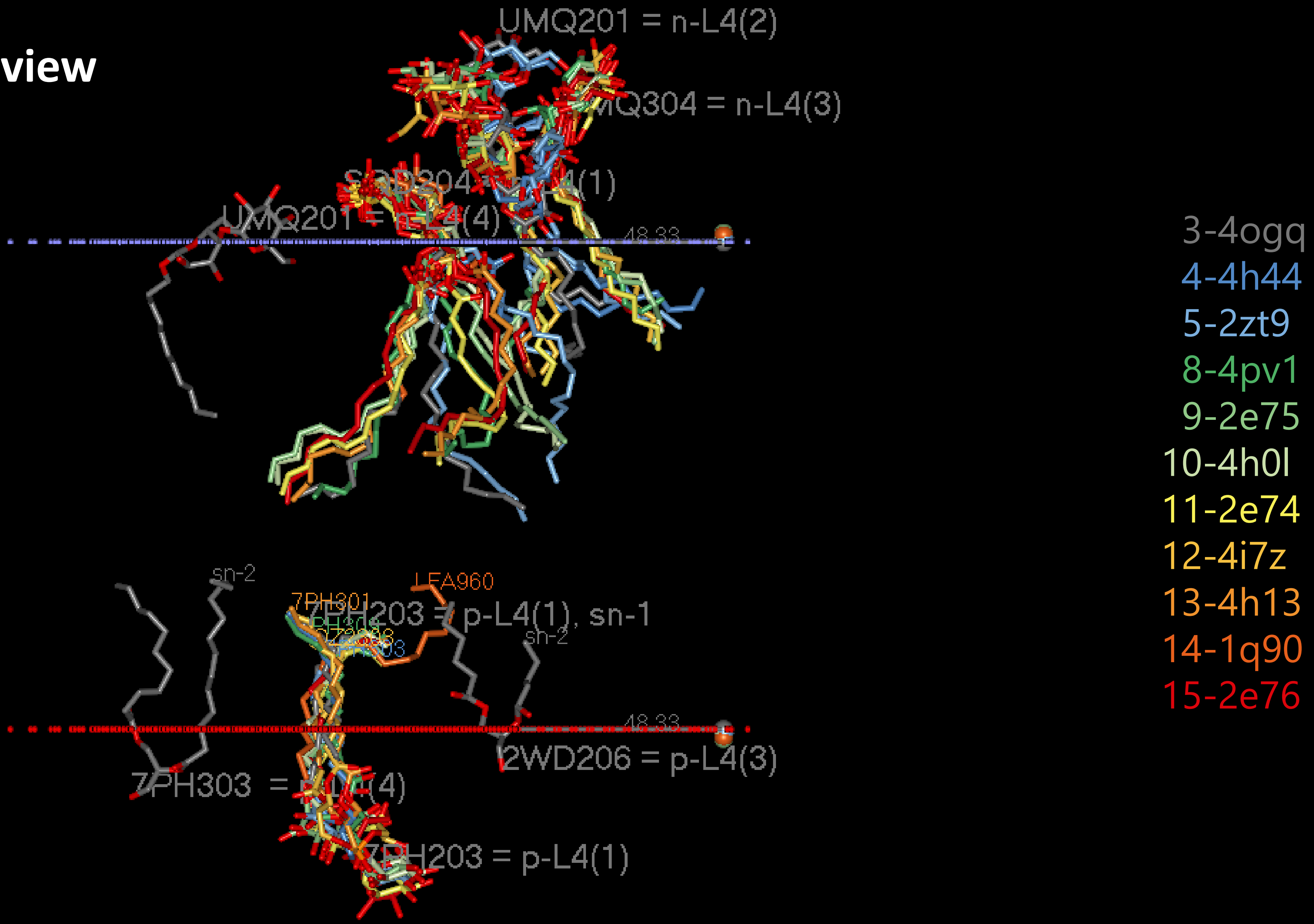

L4-sites front view

Spin 270⁰

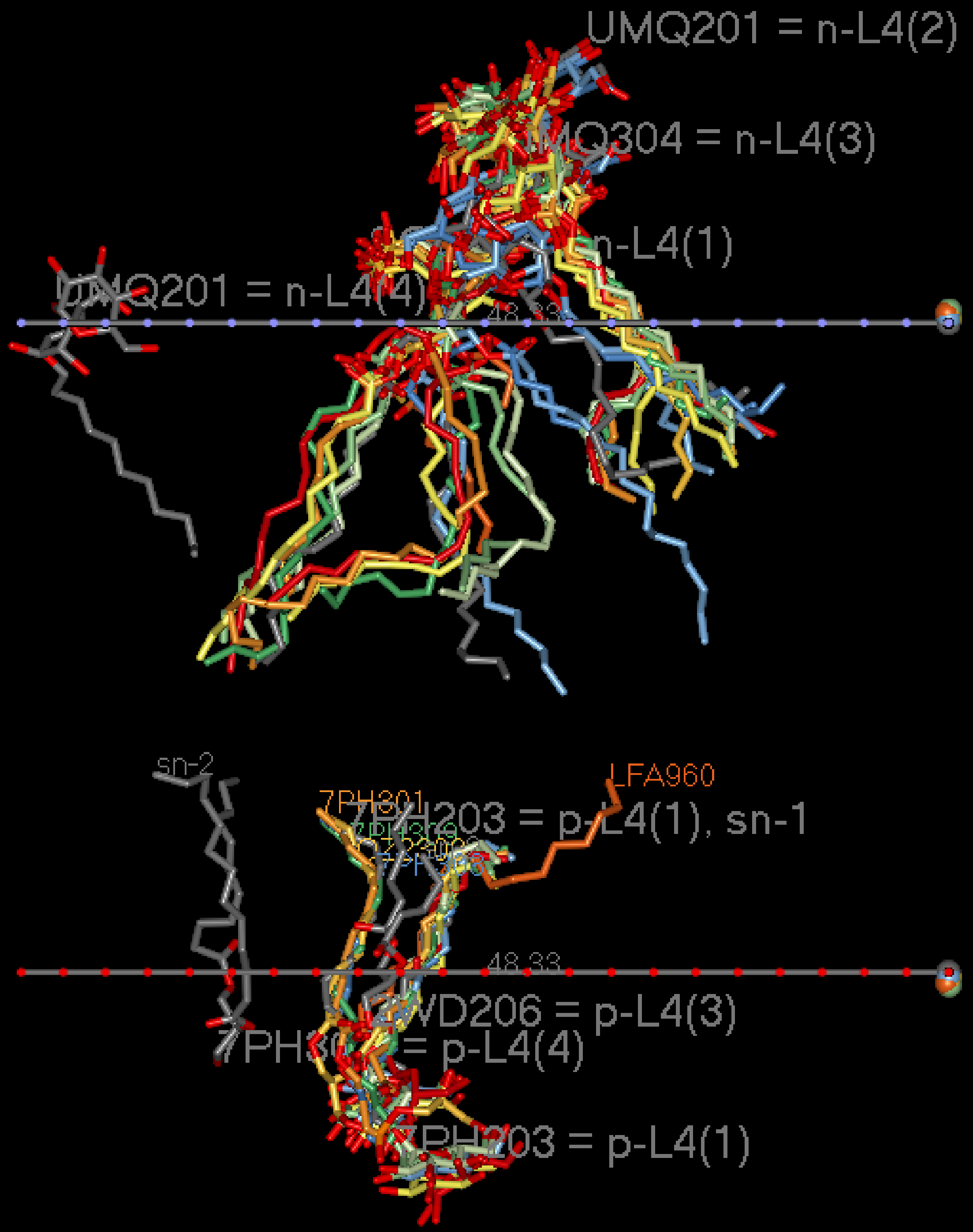

3-4ogq
4-4h44
5-2zt9
8-4pv1
9-2e75
10-4h0l
11-2e74
12-4i7z
13-4h13
14-1q90
15-2e76

n-L4(1-3)-sites front view

Spin 90°

n-L4(3) in stick and ball

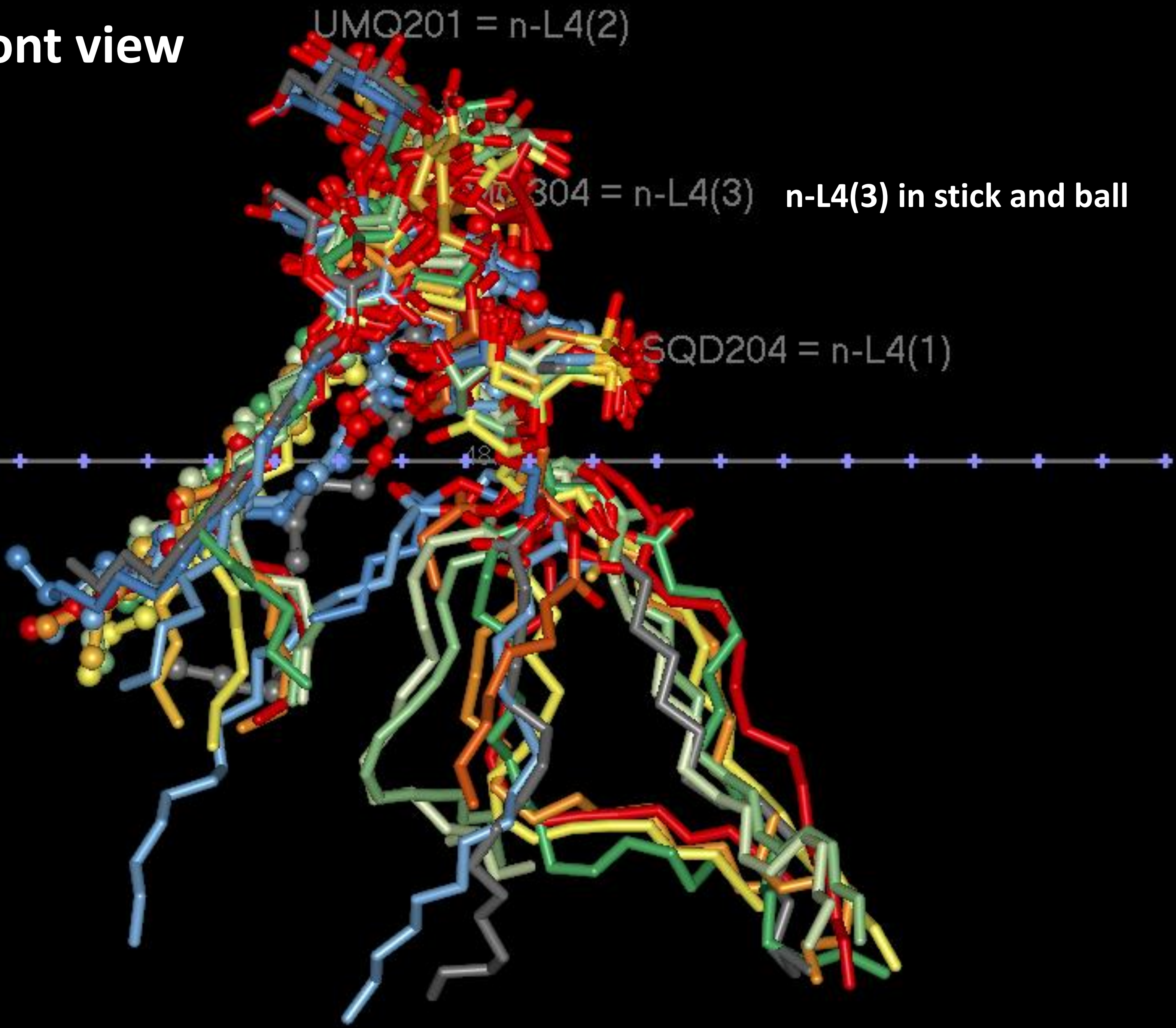

3-4ogq
4-4h44
5-2zt9
8-4pv1
9-2e75
10-4h0l
11-2e74
12-4i7z
13-4h13
14-1q90
15-2e76

- n-L4(1-3) are present in all 11 structures as a cluster of SQDG and two detergent molecules, except in 1q90 – only SQDG;
- n-L4(1-3) can be a part of larger clusters of four or eight coupled lipid/detergent molecules

n-L4(1) site (SQDG) n-side view

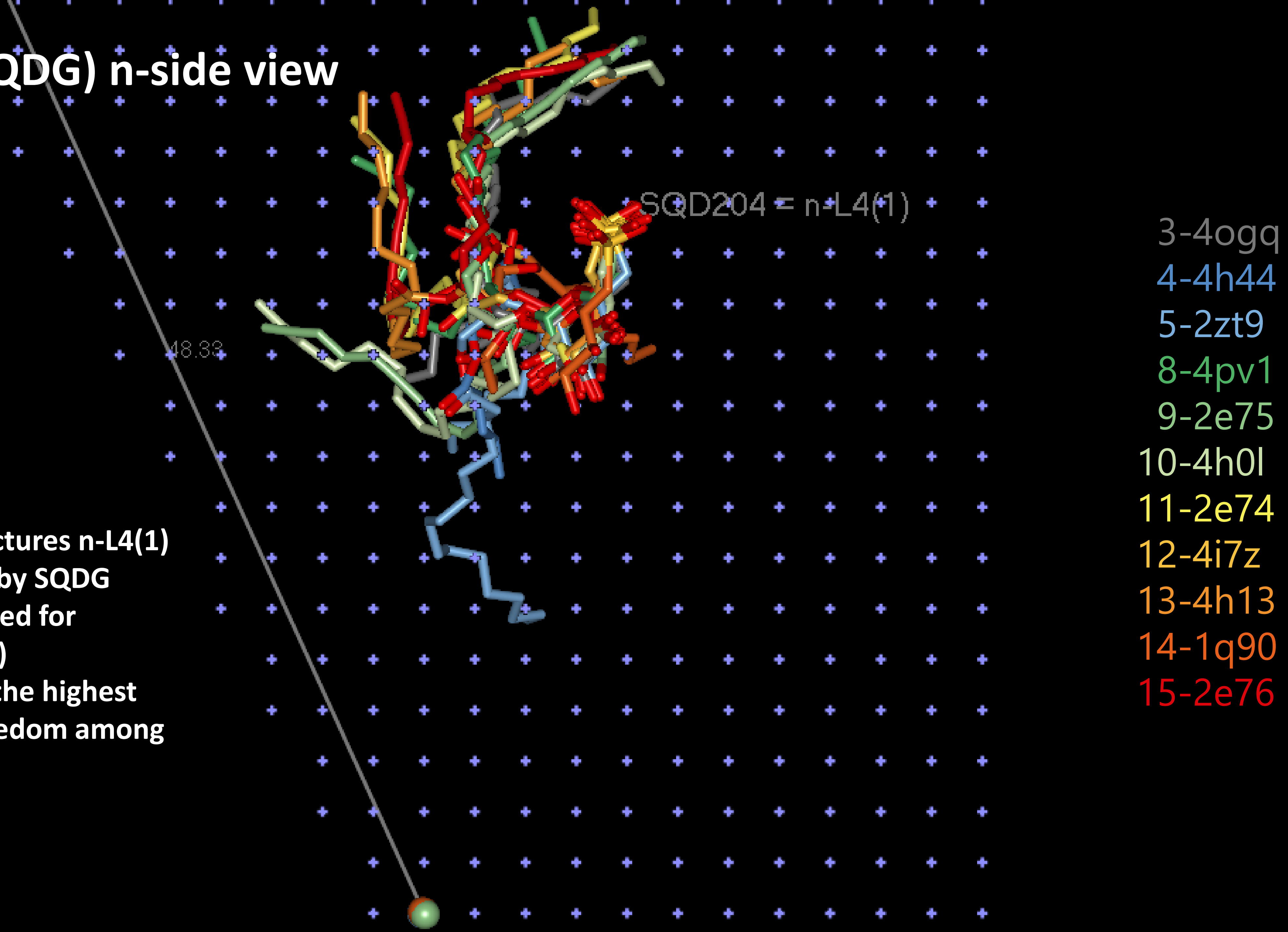

- In all 11 X-ray structures n-L4(1) is always occupied by SQDG even when PG is used for crystallization (4i7z)
- SQDG chains have the highest conformational freedom among the cytb6f lipids

n-L4(1) site (SQDG) front view

Spin 90º

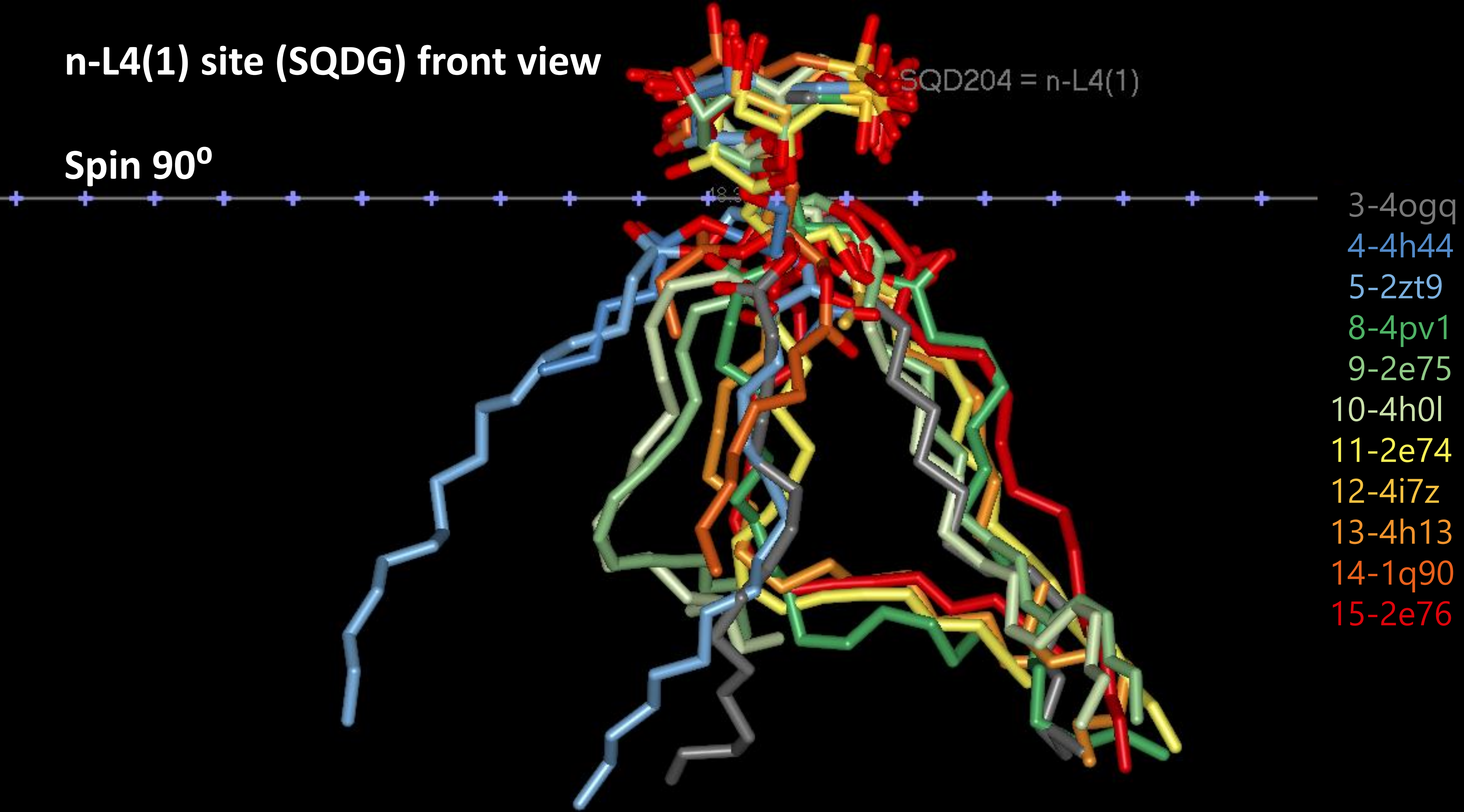

n-L4(2) site (UMQ201) n-side view

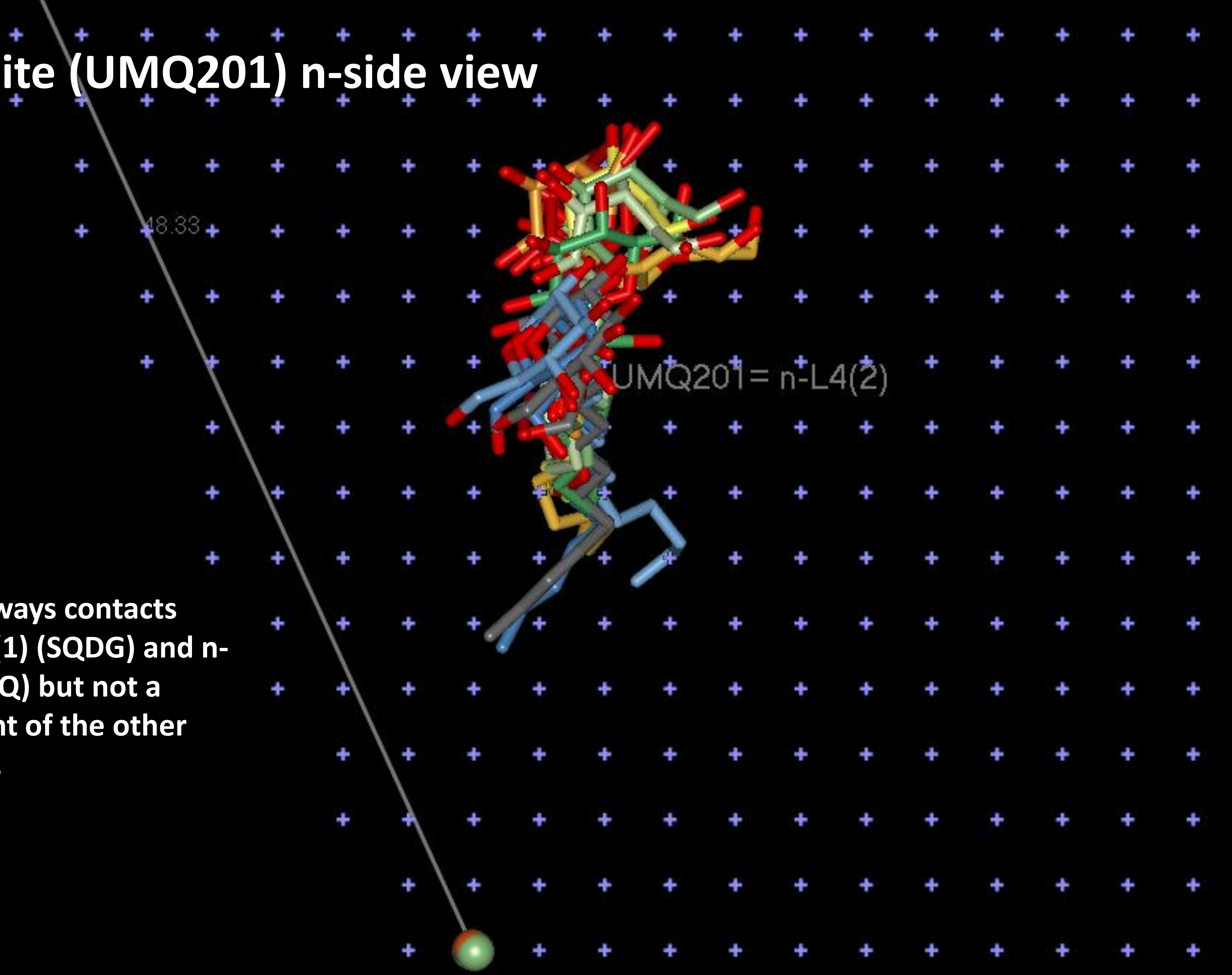

- **n-L4(2) always contacts both n-L4(1) (SQDG) and n-L4(3) (UMQ) but not a component of the other monomer.**

3-4ogq
4-4h44
5-2zt9
8-4pv1
9-2e75
10-4h0l
11-2e74
12-4i7z
13-4h13
14-1q90
15-2e76

n-L4(2) site (UMQ201) front view

Spin 90°

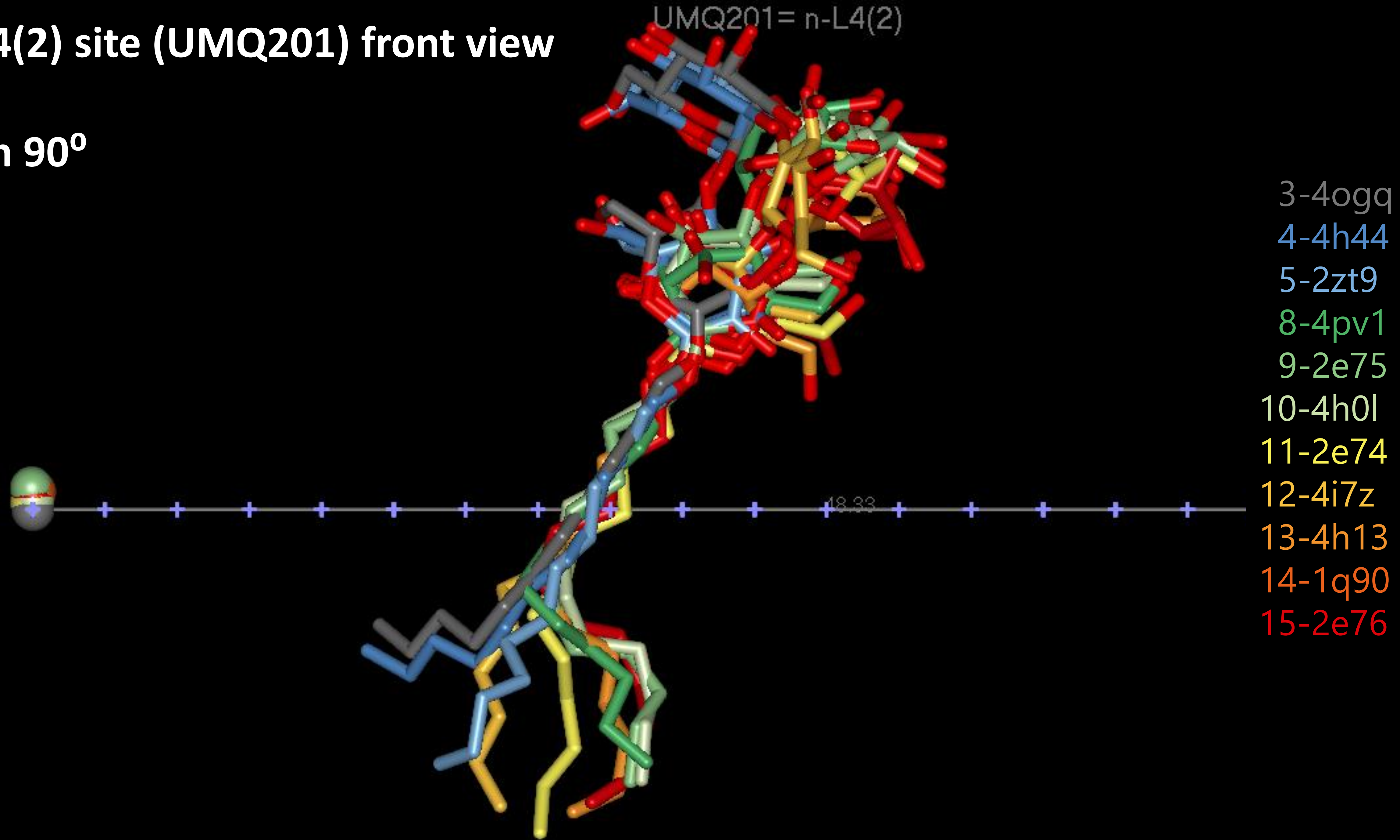

n-L4(3) site (UMQ304) n-side view

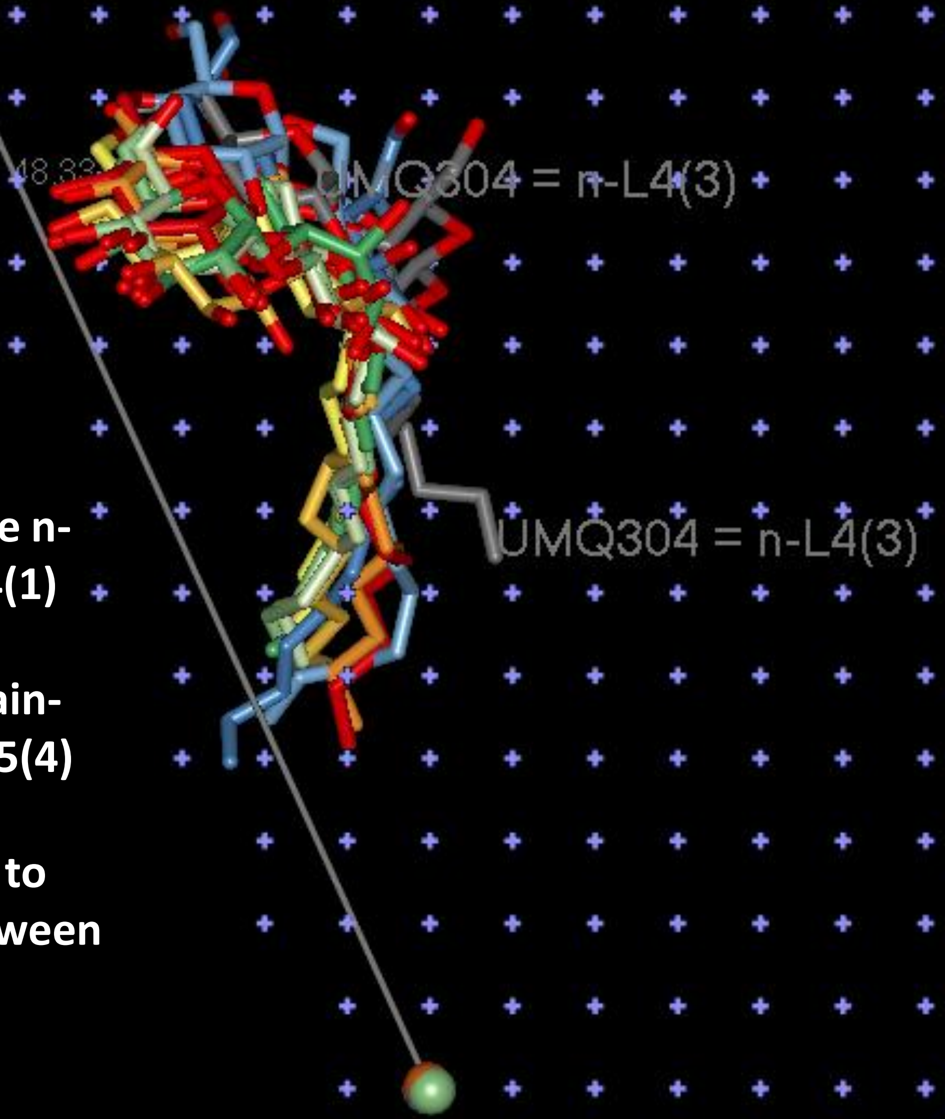

3-4ogq
4-4h44
5-2zt9
8-4pv1
9-2e75
10-4h0l
11-2e74
12-4i7z
13-4h13
14-1q90
15-2e76

- **n-L4(3) always contacts the n-L4(2), can contact the n-L4(1) (SQDG) and can provide intermonomer (dimer) chain-chain contact with the n-L5(4) (UMQ) of the monomer 2;**
- **This site is the closest one to the lipid interface line between the two cytb6f monomers**

n-L4(3) site (UMQ304) front view

Spin 0º

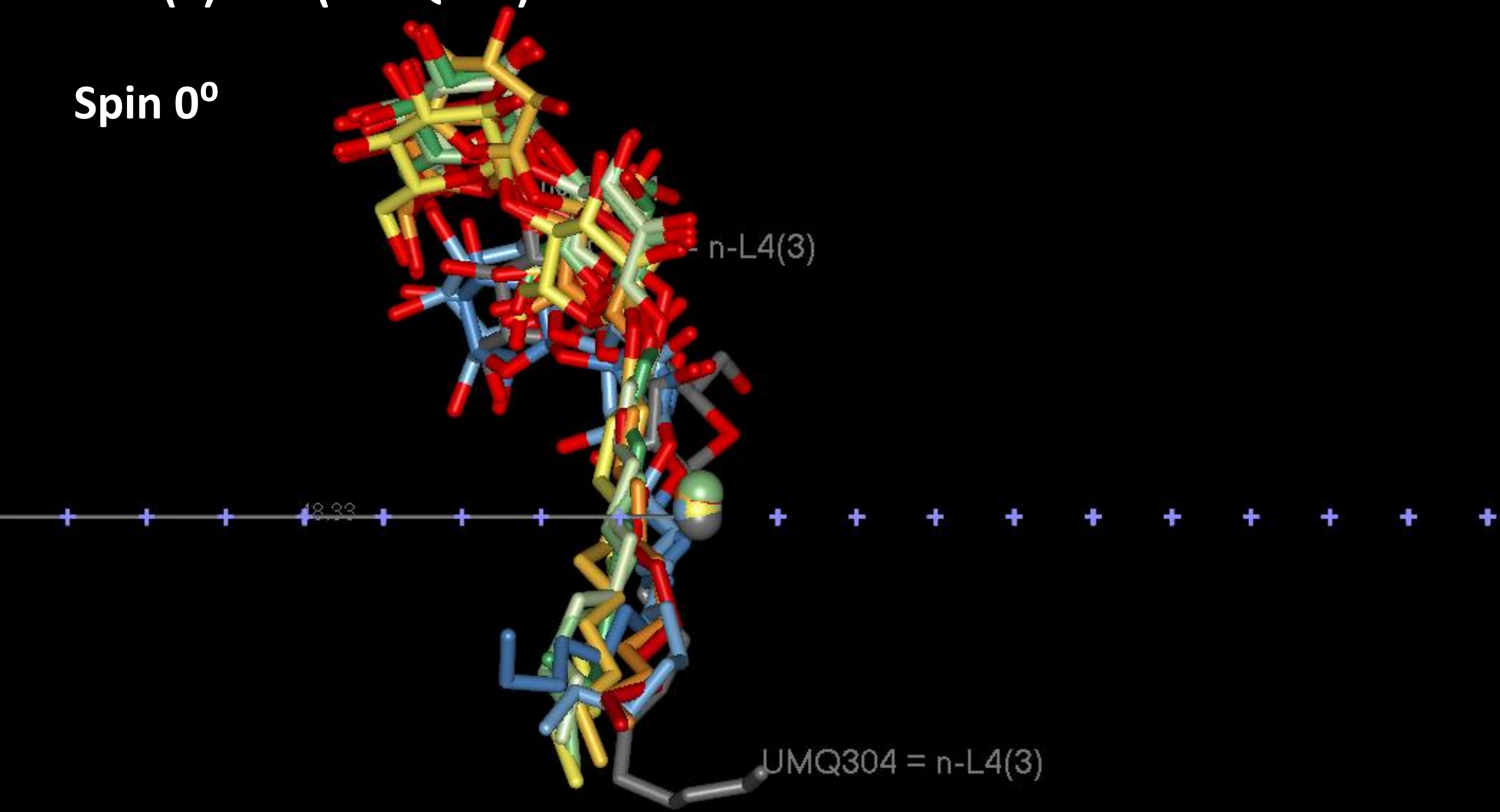

3-4ogq
4-4h44
5-2zt9
8-4pv1
9-2e75
10-4h0l
11-2e74
12-4i7z
13-4h13
14-1q90
15-2e76

n-L4(3) site (UMQ304) front view

Spin 90°

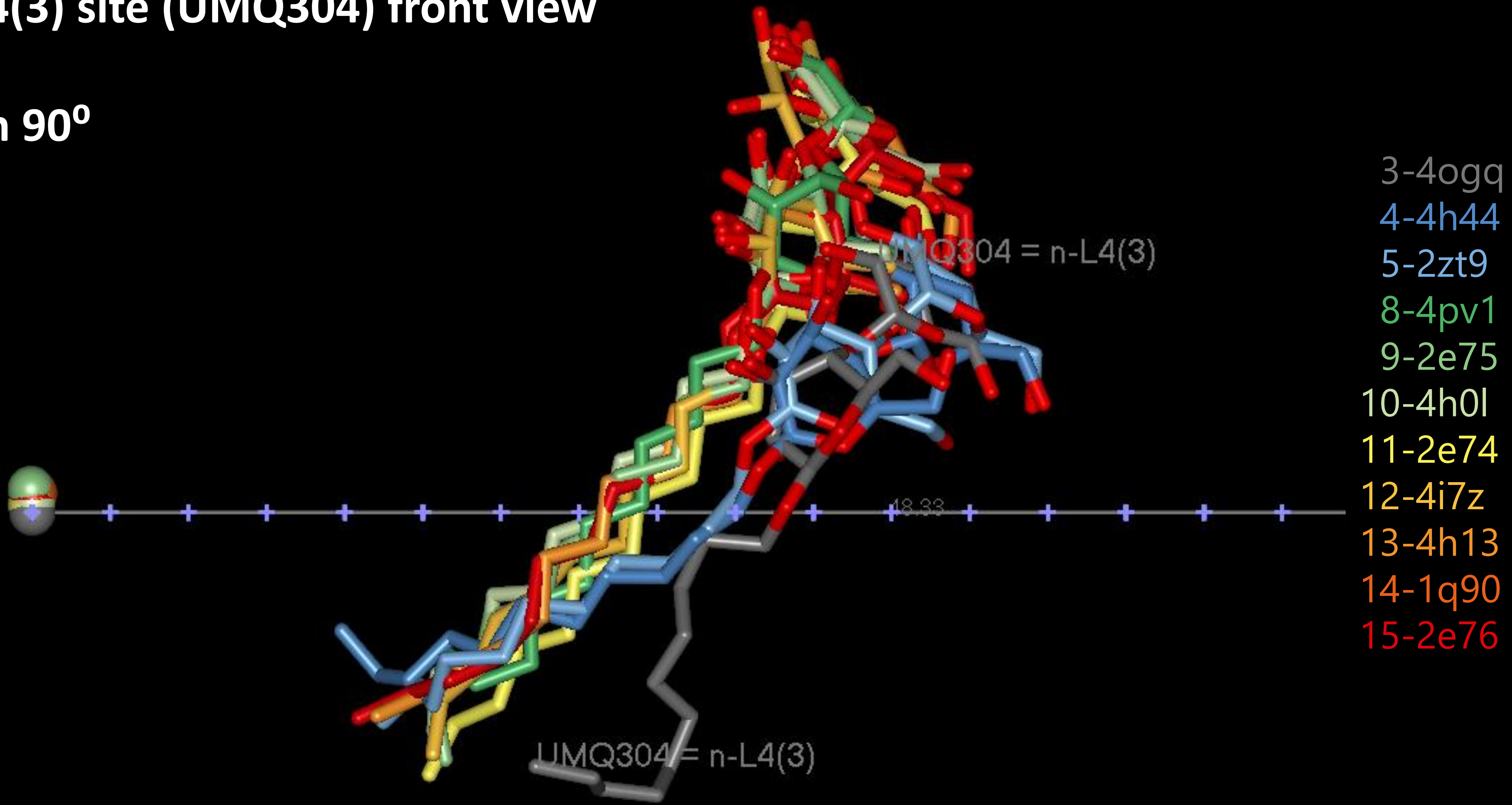

p-L4(1) site (7PH203) closer p-side view

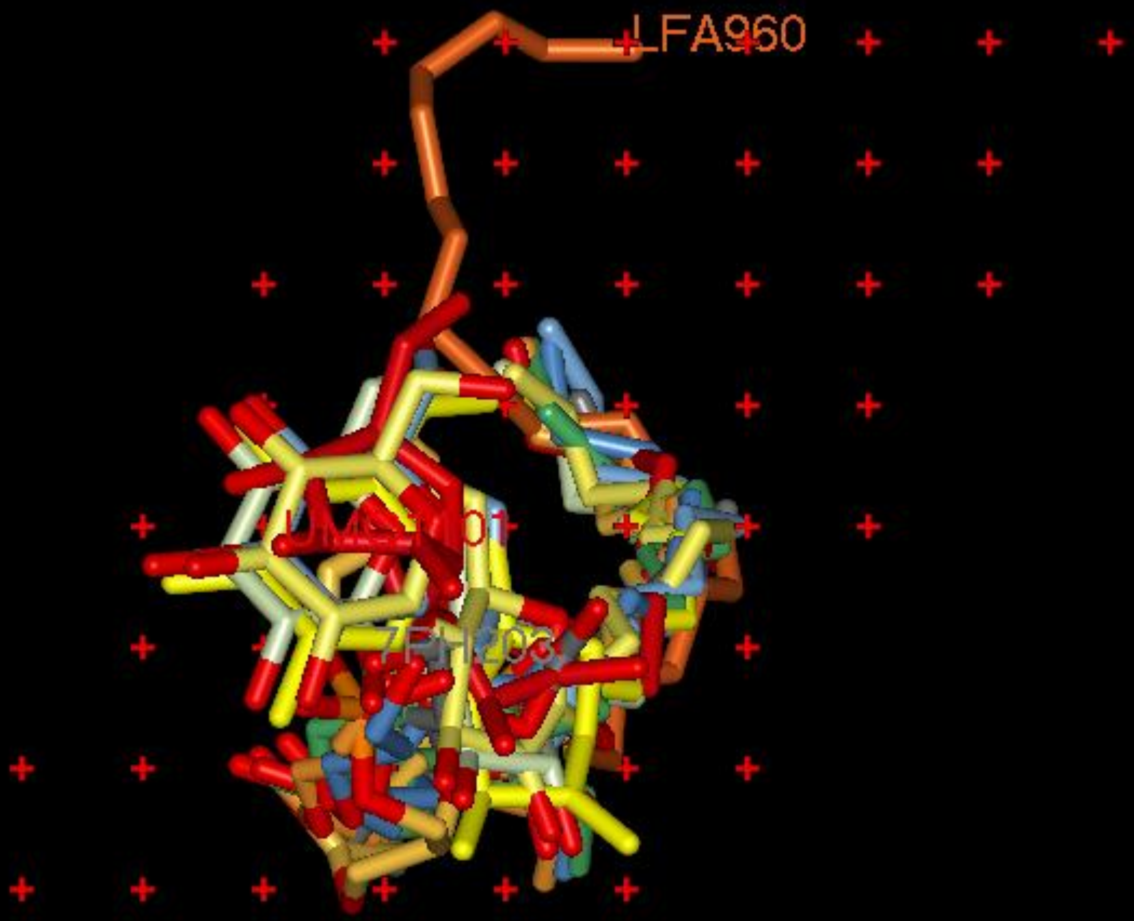

3-4ogq
4-4h44
5-2zt9
8-4pv1
9-2e75
10-4h0l
11-2e74
12-4i7z
13-4h13
14-1q90
15-2e76

p-L4(1) site (7PH203) front view

Spin 0º

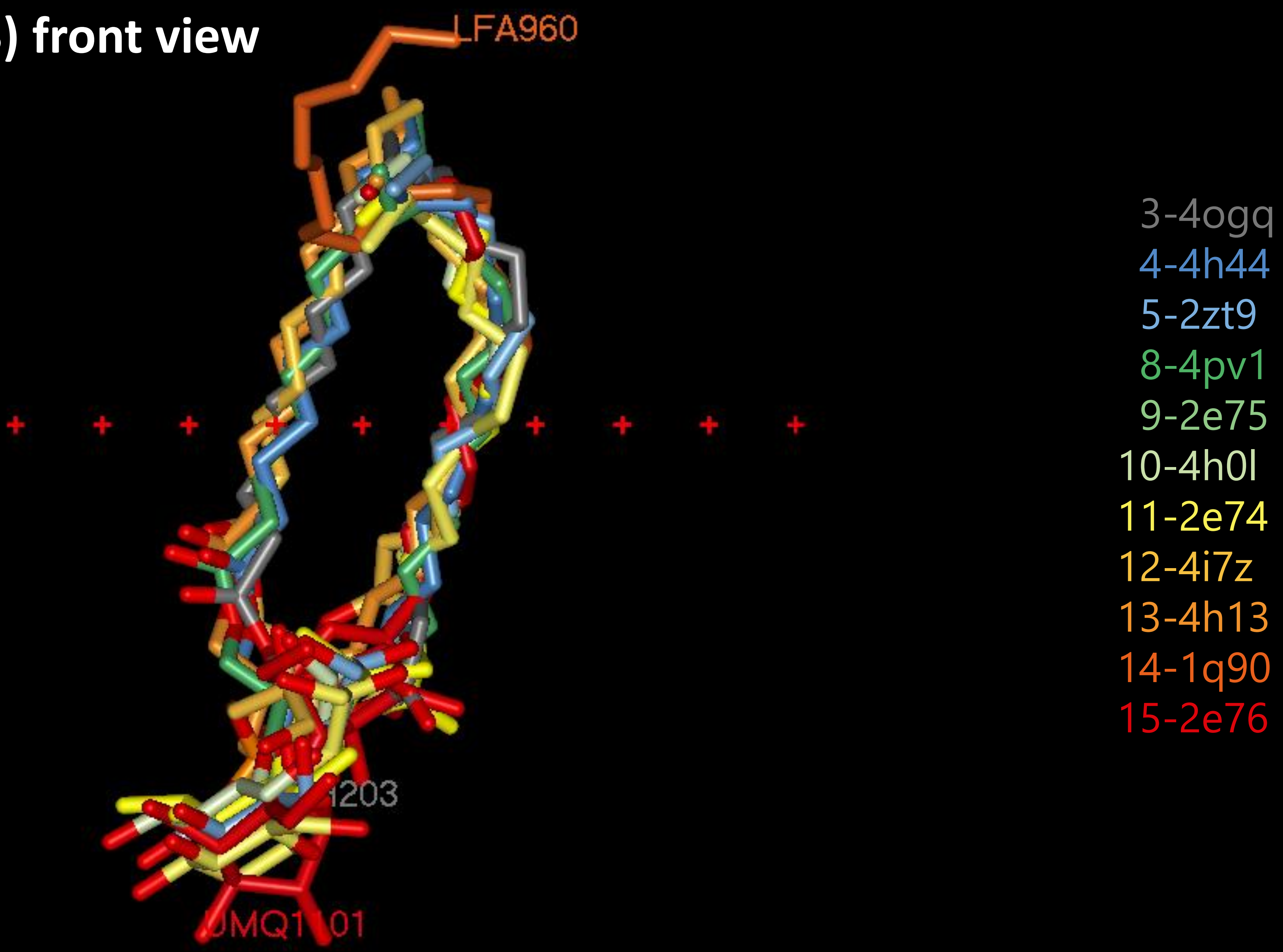

p-L4(1) site (7PH203)
front view

Spin 90⁰

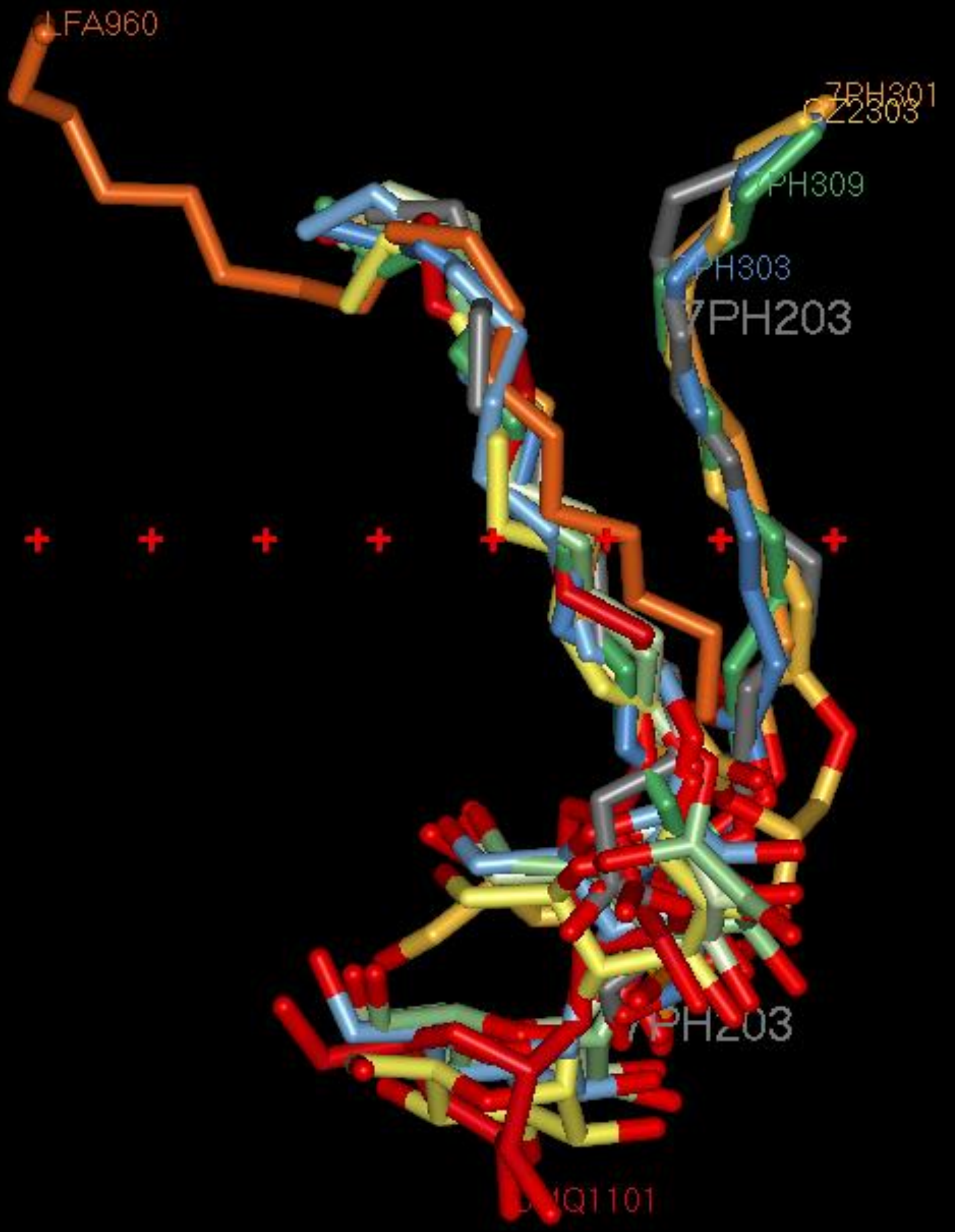

3-4ogq
4-4h44
5-2zt9
8-4pv1
9-2e75
10-4h0l
11-2e74
12-4i7z
13-4h13
14-1q90
15-2e76

L5-sites (Phe40, lipidic intermonomer interface) p-side view

Chl*a*, Phe40 and heme *c*n of 4ogq are only shown

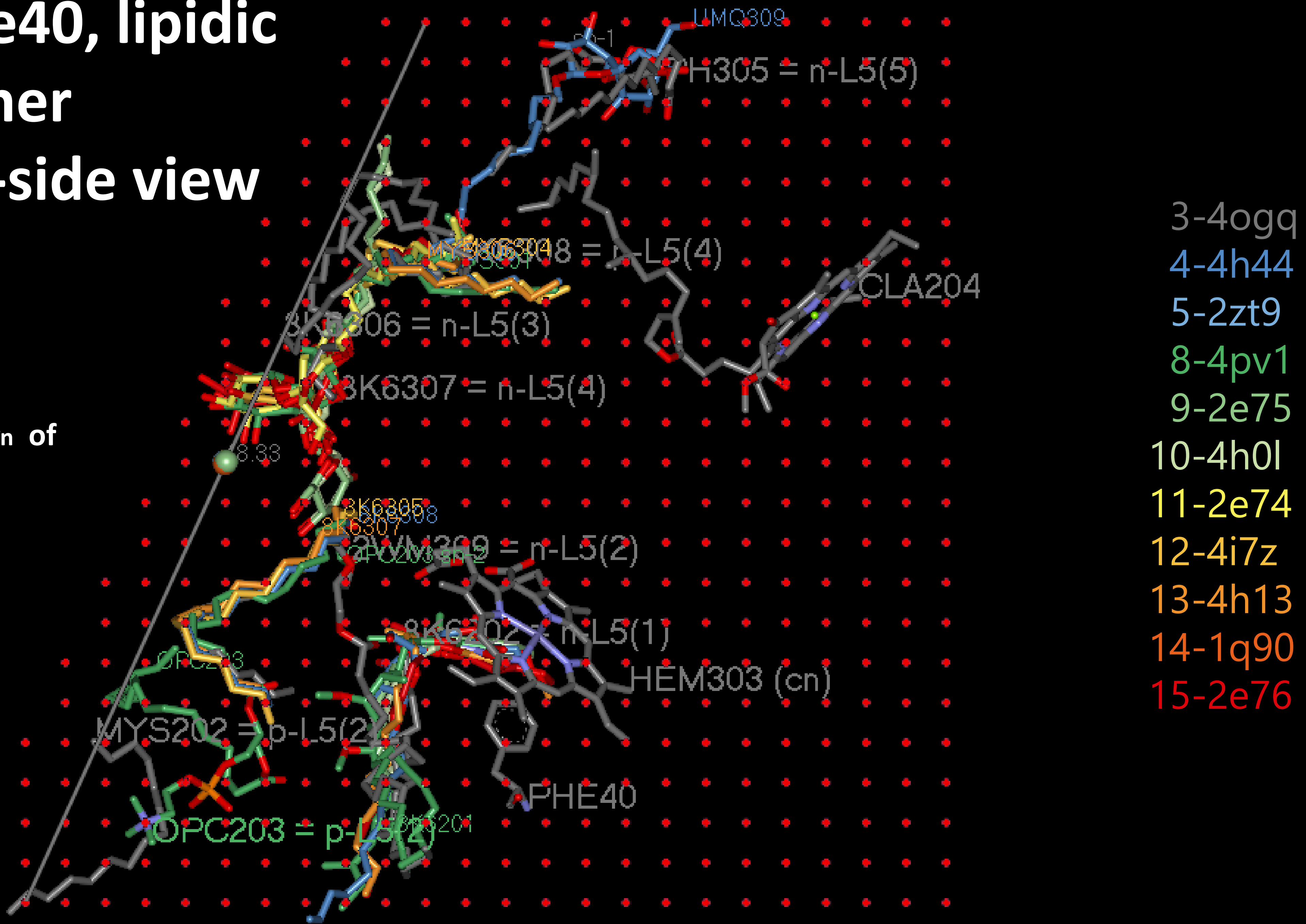

L5-sites n-side view

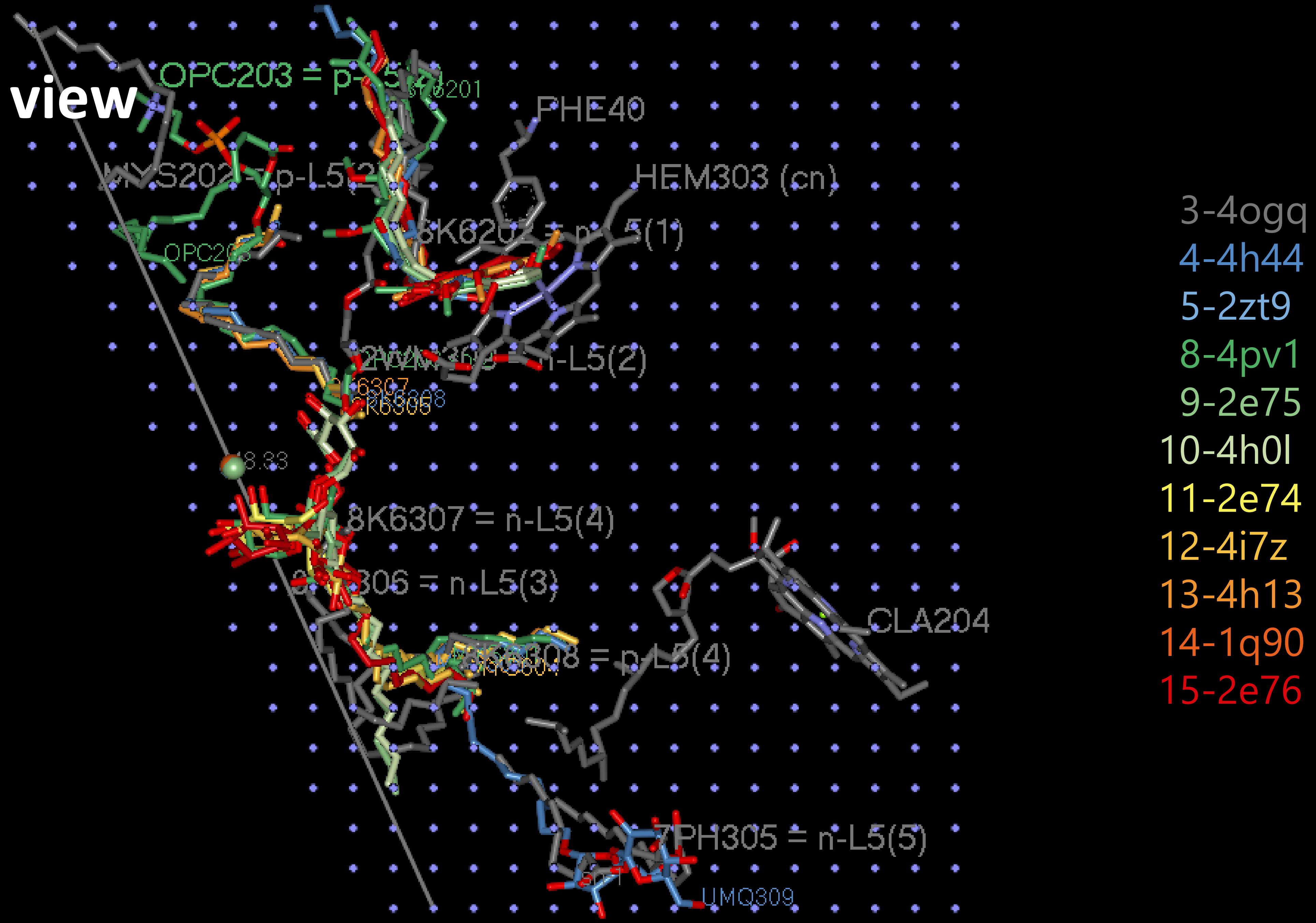

L5-sites front view

Spin 0º

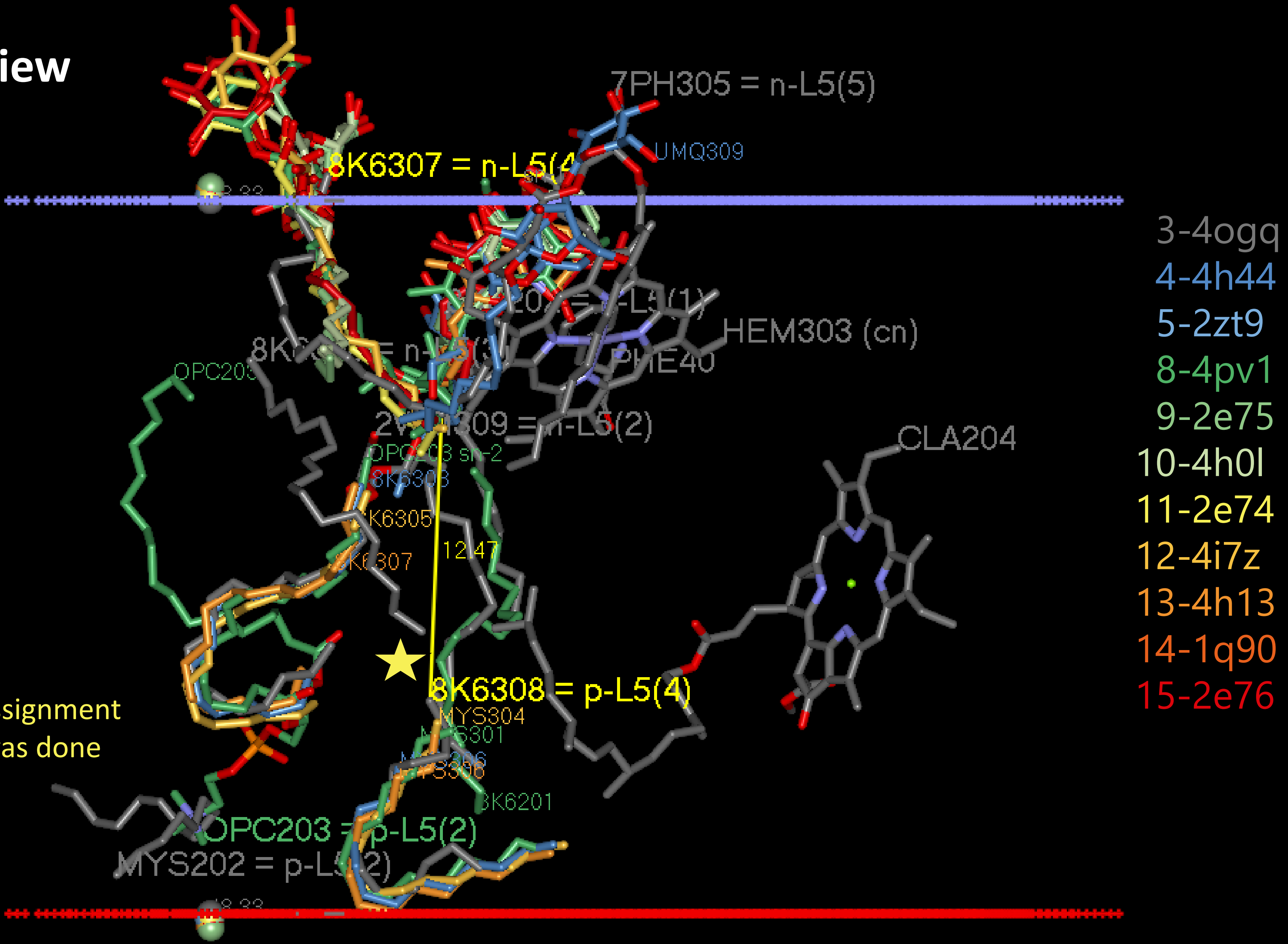

★ Example of how the assignment of p-side numbering was done

L5-sites front view

Spin 90º

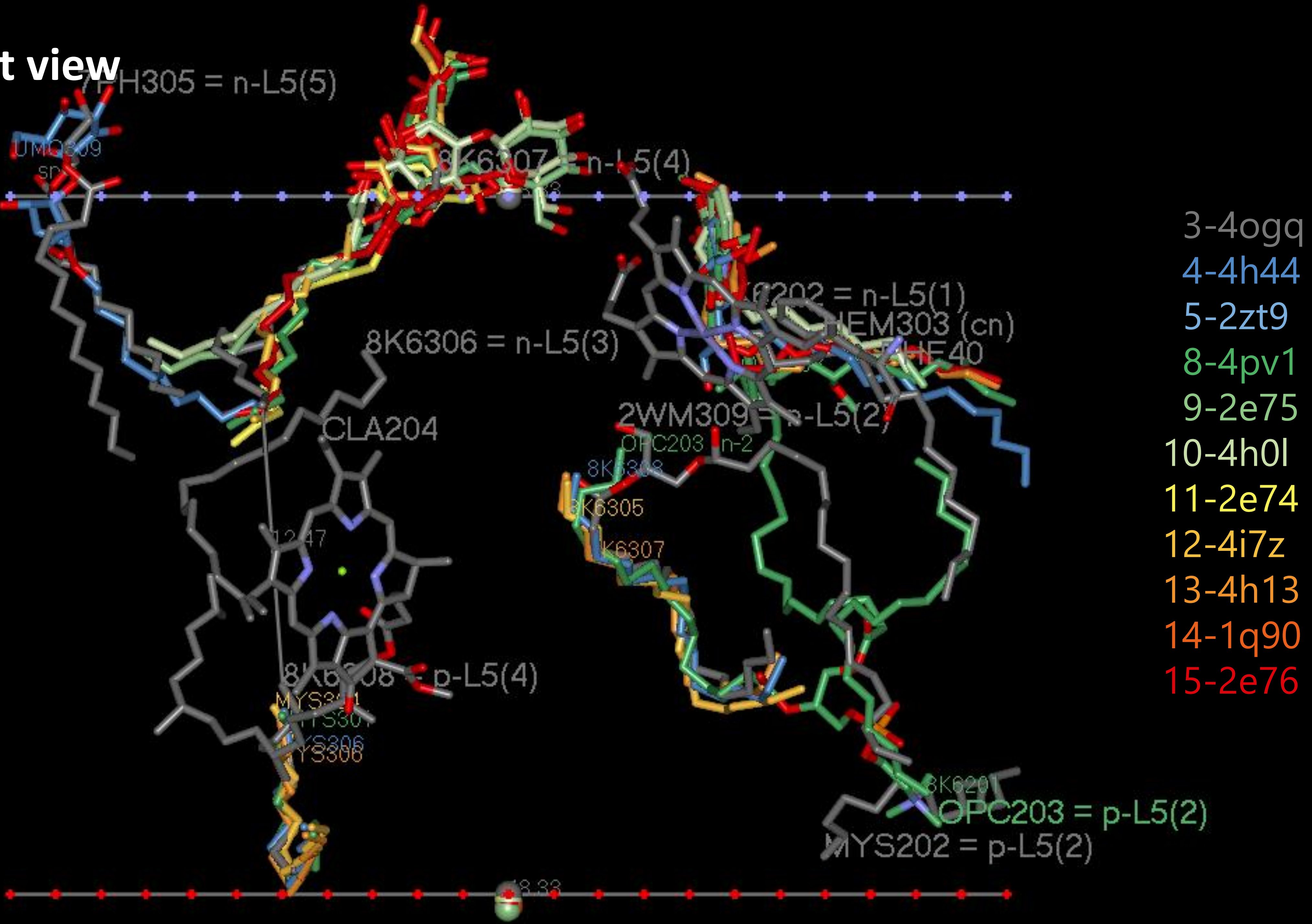

L5-site front view

Spin 180°

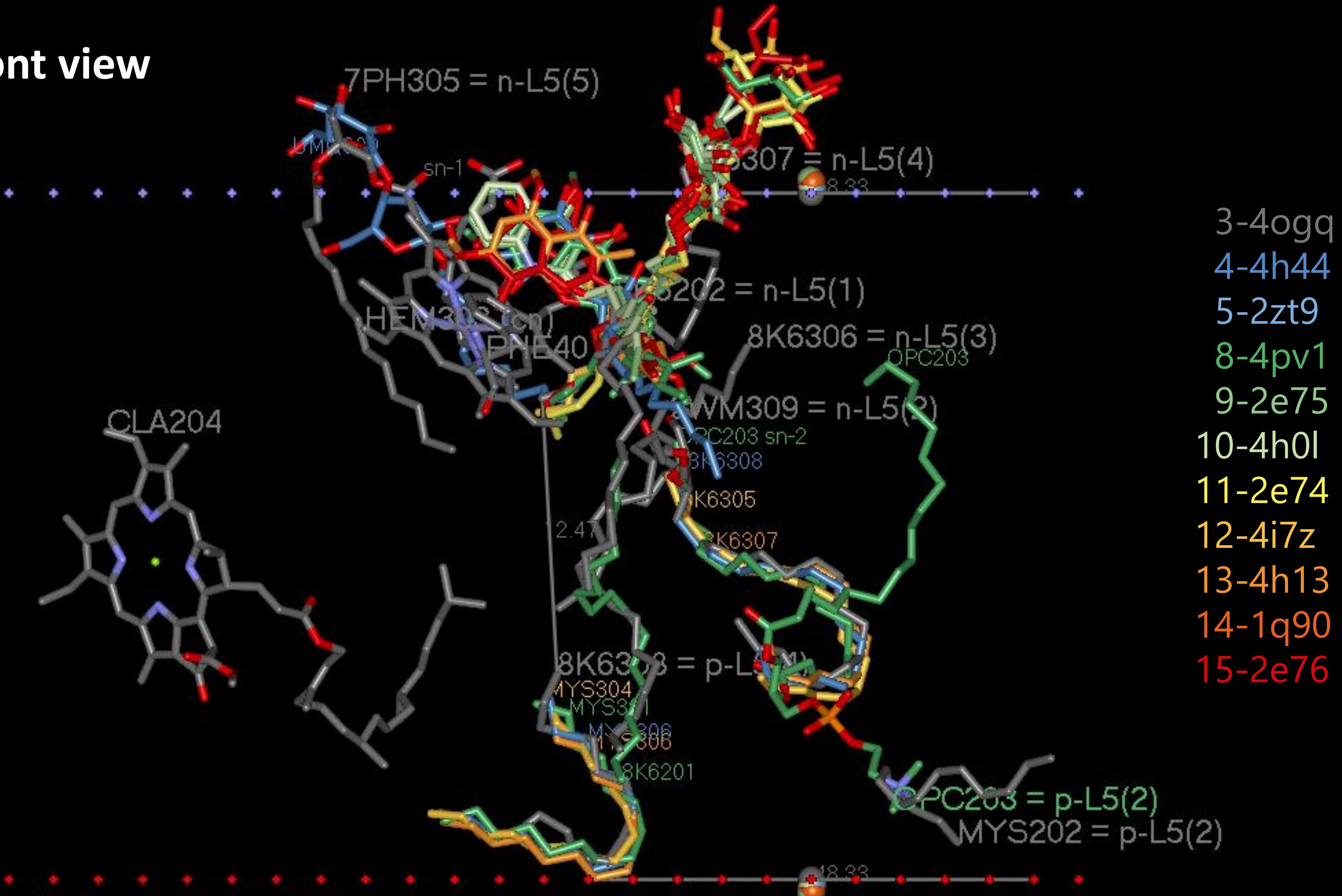

n-L5(1) site (Phe40)
n-side view

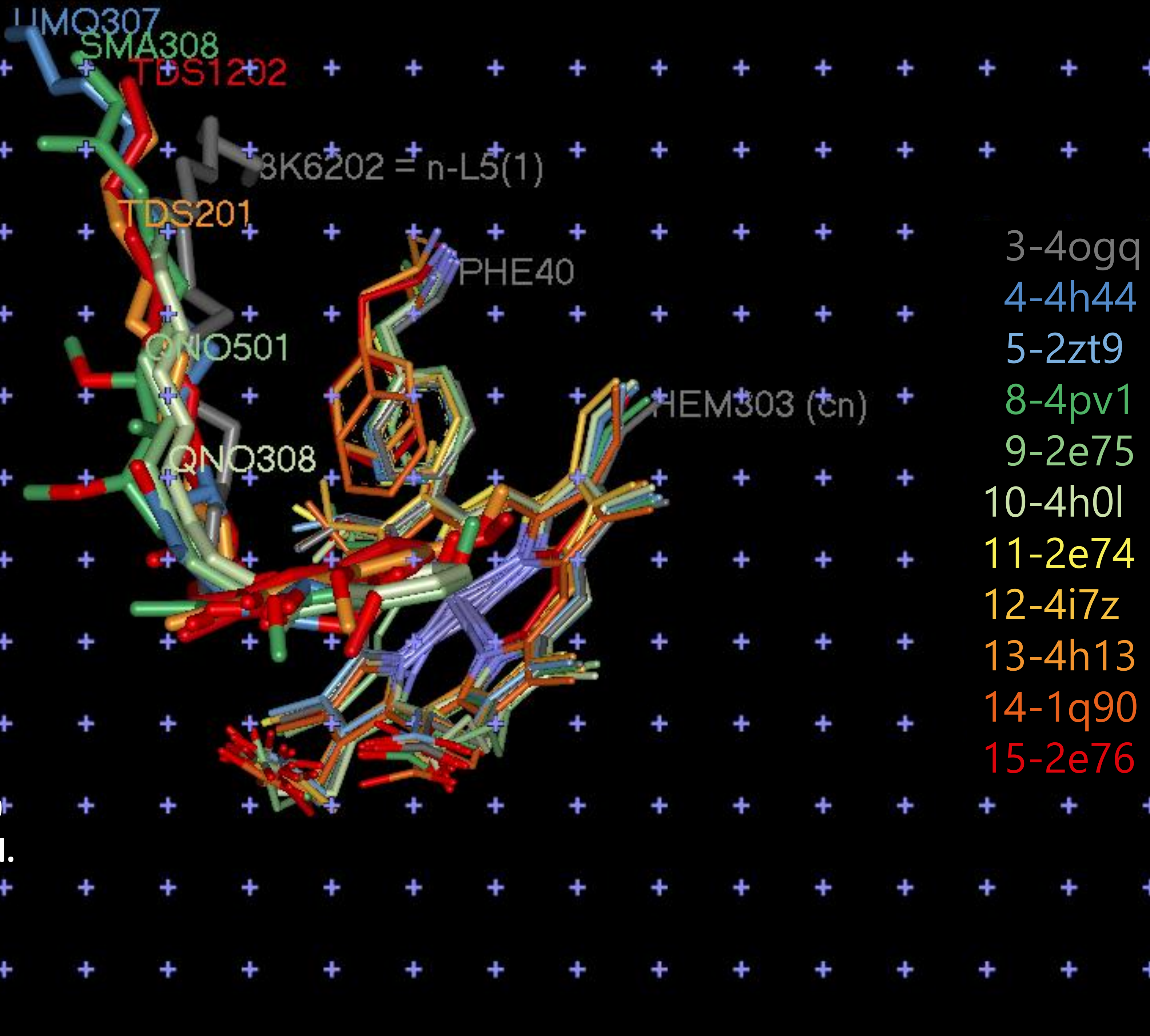

- n-L5(1) site is in fact the Qn site – the site of PQ reduction.
- The occupant (thicker stick) always contacts both Phe40 and heme cn.
- Note that only TDS shifts the Phe40.
- An occupant was not modeled in 1q90 but there is probably PQ [Stroebel et al. (2003) In the main text].

n-L5(1) site (Qn) front view

Spin 130⁰

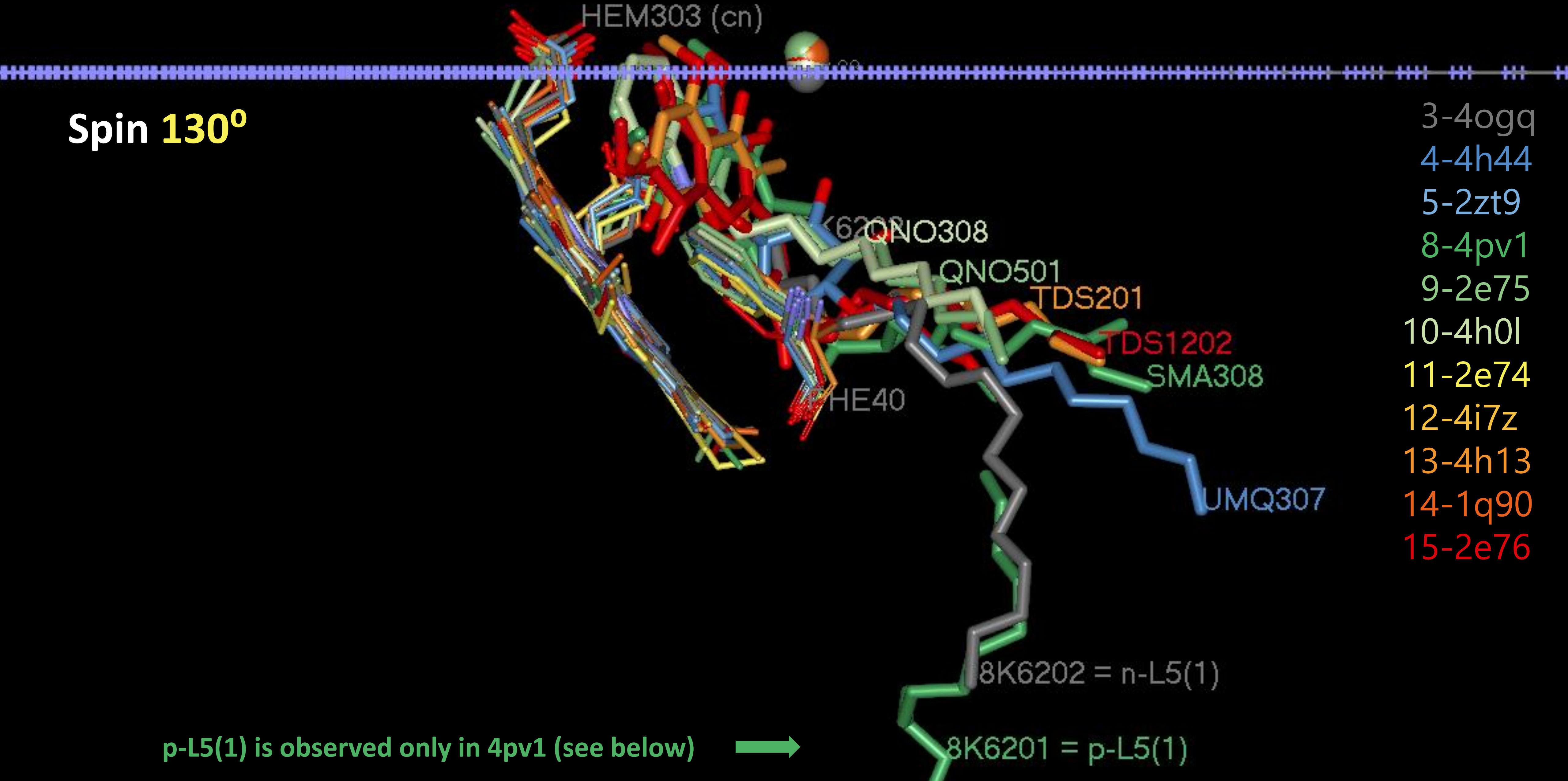

p-L5(1) is observed only in 4pv1 (see below) →

n-, p-L5(2) core lipid sites p-side view

- The sn-2 chain of n-L5(2) (3-4ogq) and p-L5(2) (8-4pv1) lipids coincide, but they have opposite head group orientation;
- The sn-2 chain position is also occupied by hydrocarbon chain in 4-4h44, 12-4i7z and 13-4h13. These are the same five structures having together the same occupants for p-L4(1) and n-L3(1) sites;
- This coincidence indicates similar dynamics (mobility) of these sites in these five structures. At the end are the images for the 1vf5 and 2d2c.

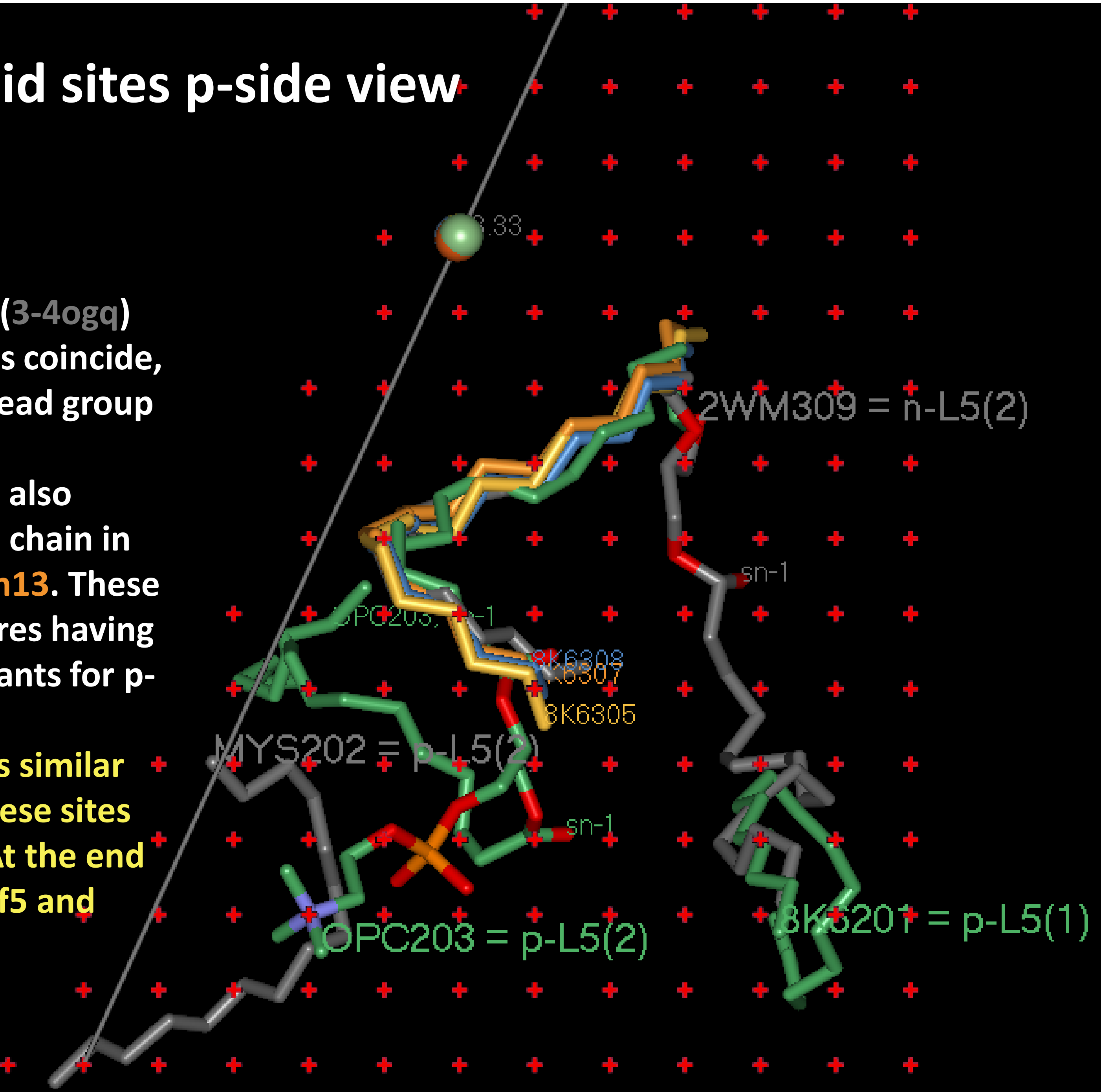

3-4ogq
4-4h44
5-2zt9
8-4pv1
9-2e75
10-4h0l
11-2e74
12-4i7z
13-4h13
14-1q90
15-2e76

n-, p-L5(2) core sites front view

Spin 0º

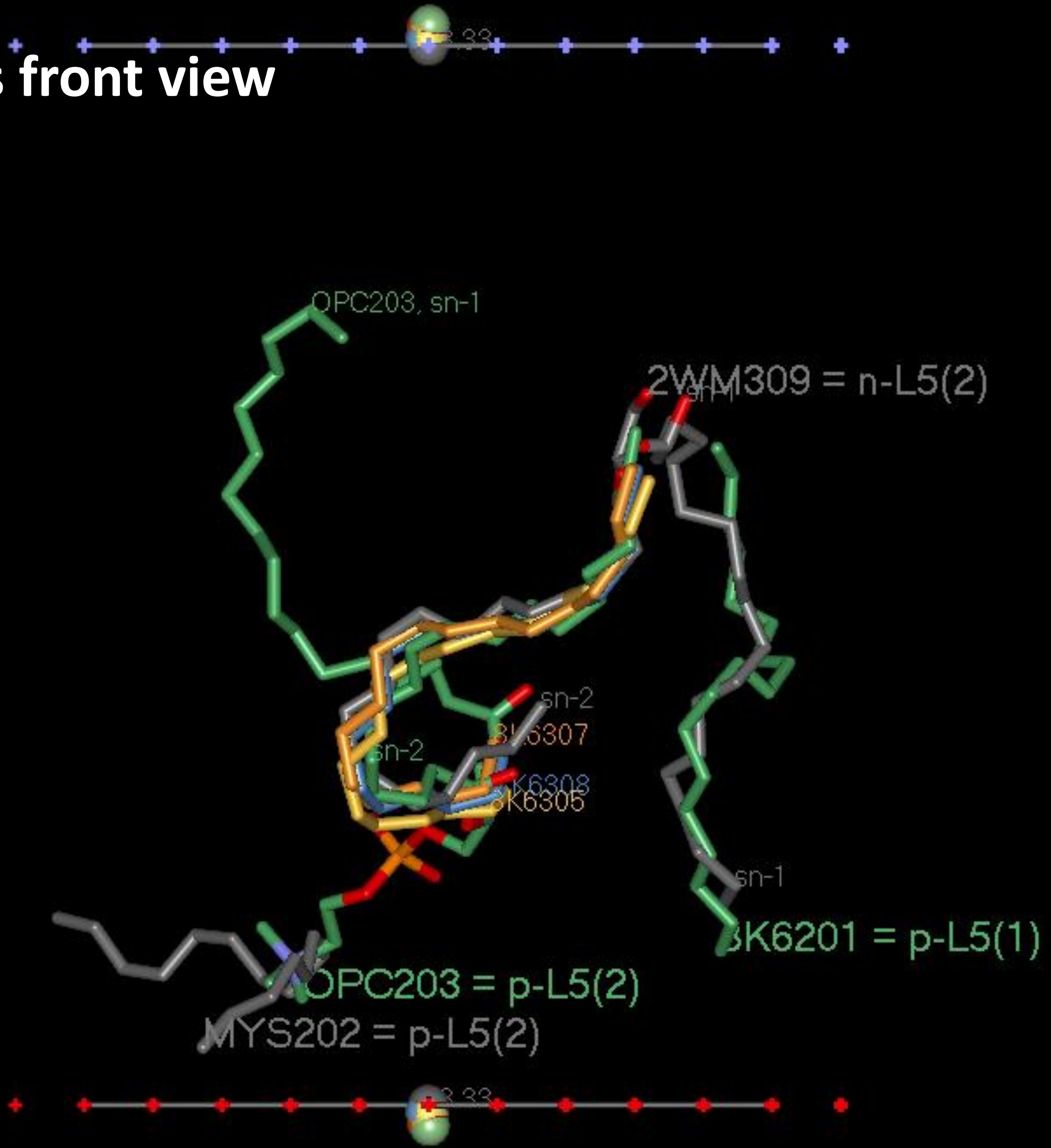

3-4ogq
4-4h44
5-2zt9
8-4pv1
9-2e75
10-4h0l
11-2e74
12-4i7z
13-4h13
14-1q90
15-2e76

n-, p-L5(2) core sites front view

Spin 60°

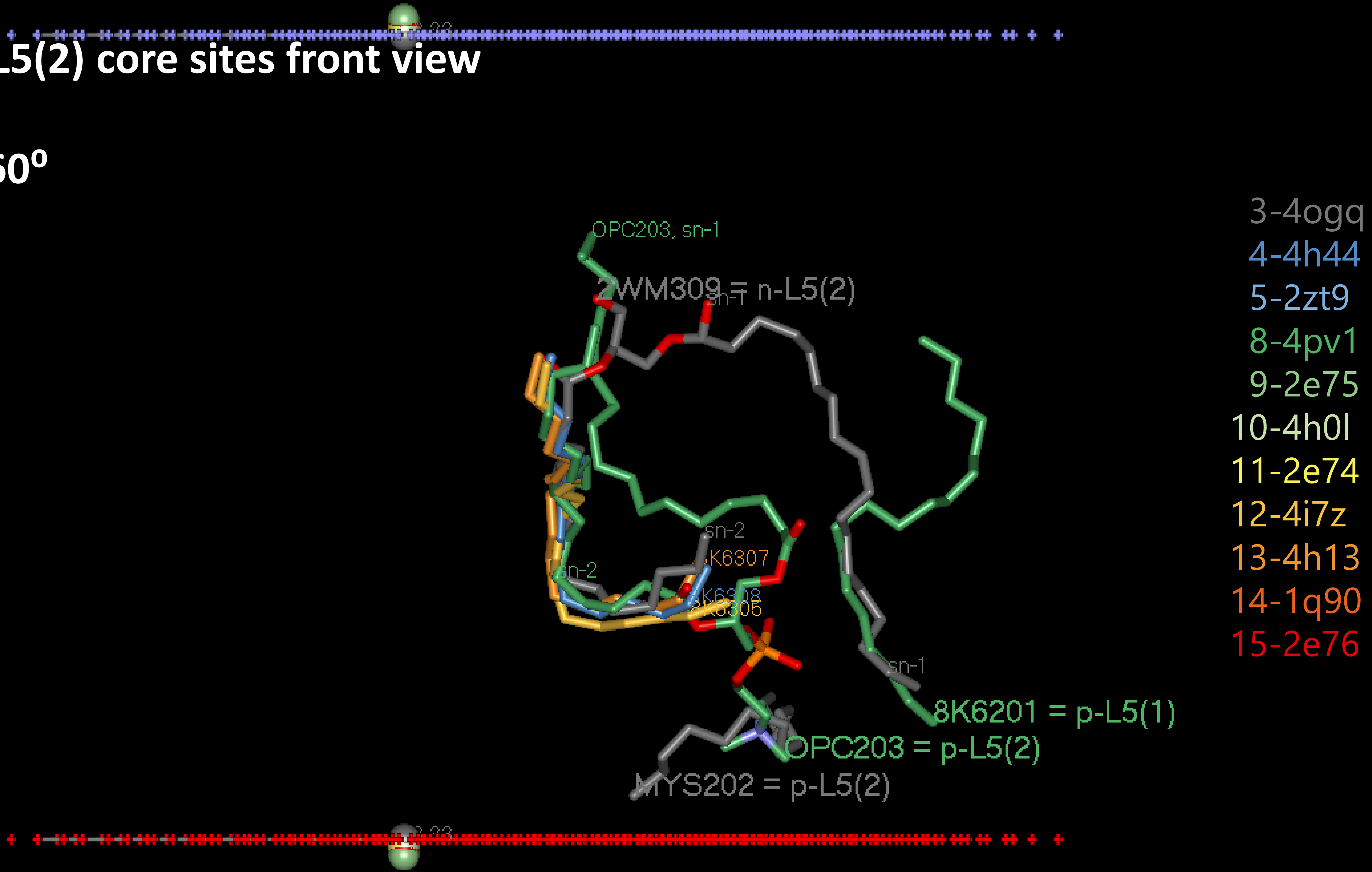

n-L5(4) – intermonomer lipid-lipid contact site

p-side view

- The detergent at n-L5(4) site can contact with the same from the other monomer via head-head contact;
- At the same time, it can also contact with the n-L4(3) detergent from the other monomer to create 2 x 4 coupled or 8 coupled molecules at the lipid-lipid intermonomer interface;
- Only in 4ogq, it is a hydrocarbon – 8K6307.

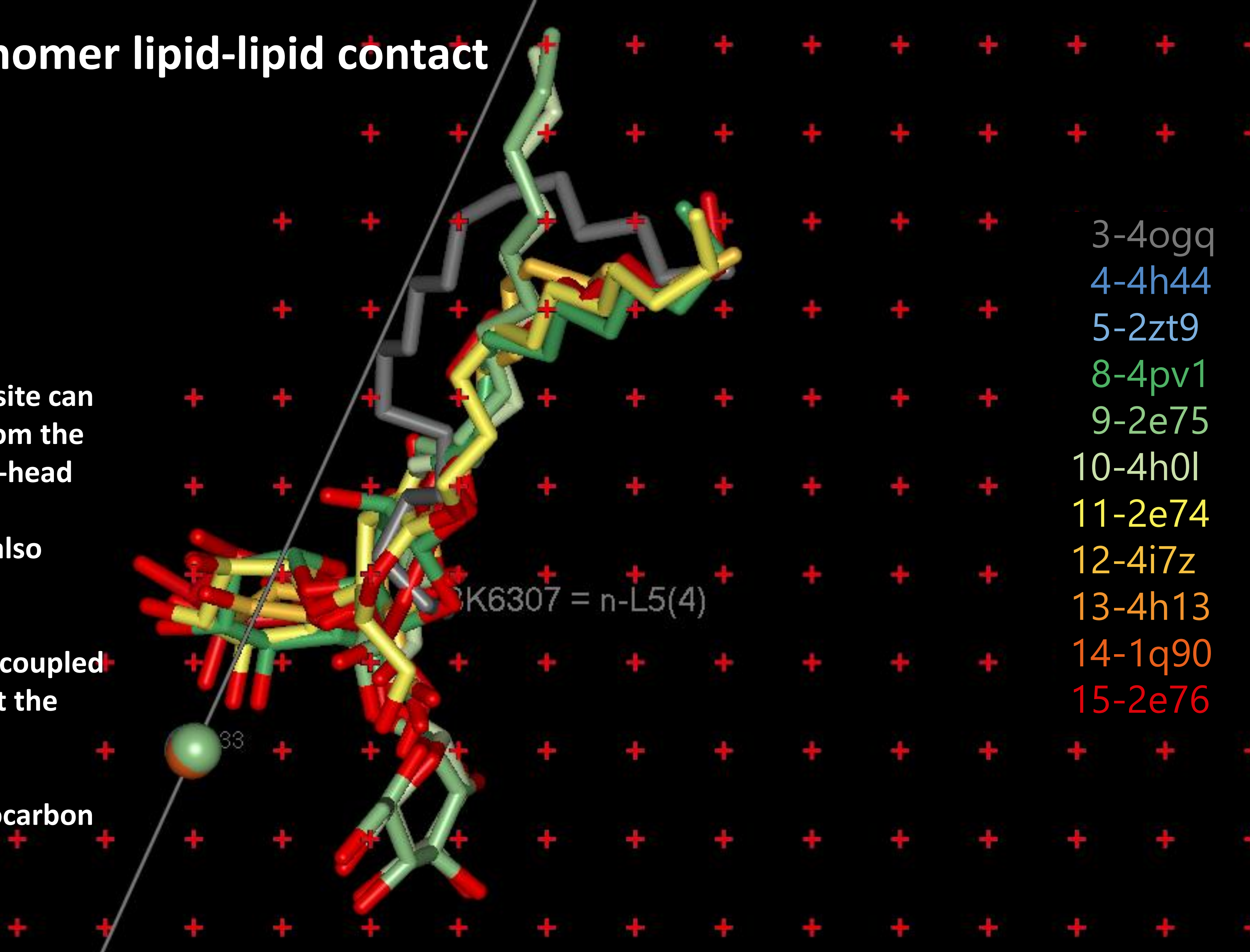

n-L5(4) – intermonomer lipid-lipid contact site

n-side view

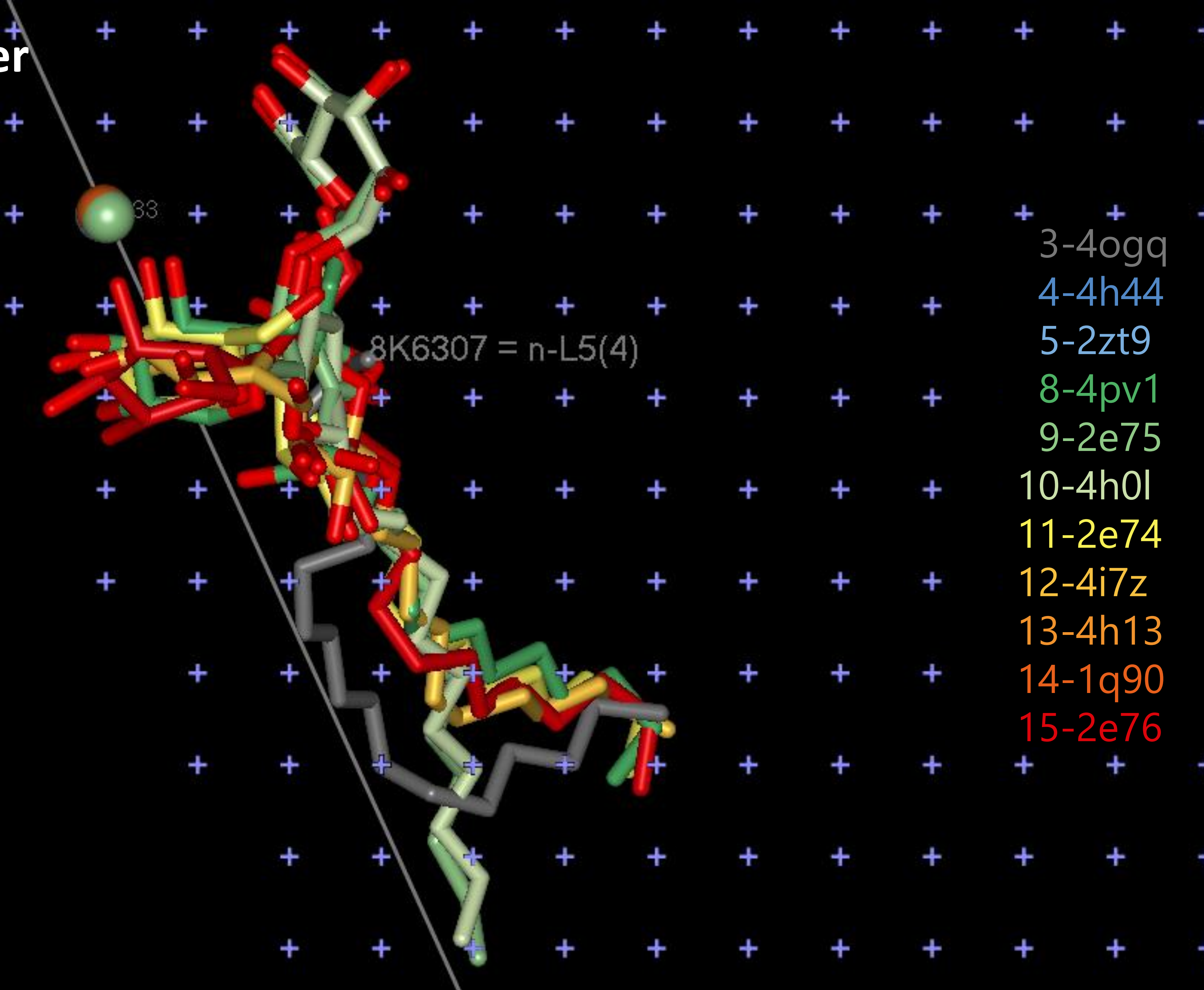

n-L5(4) – intermonomer lipid-lipid contact site

Front view

Spin 0º

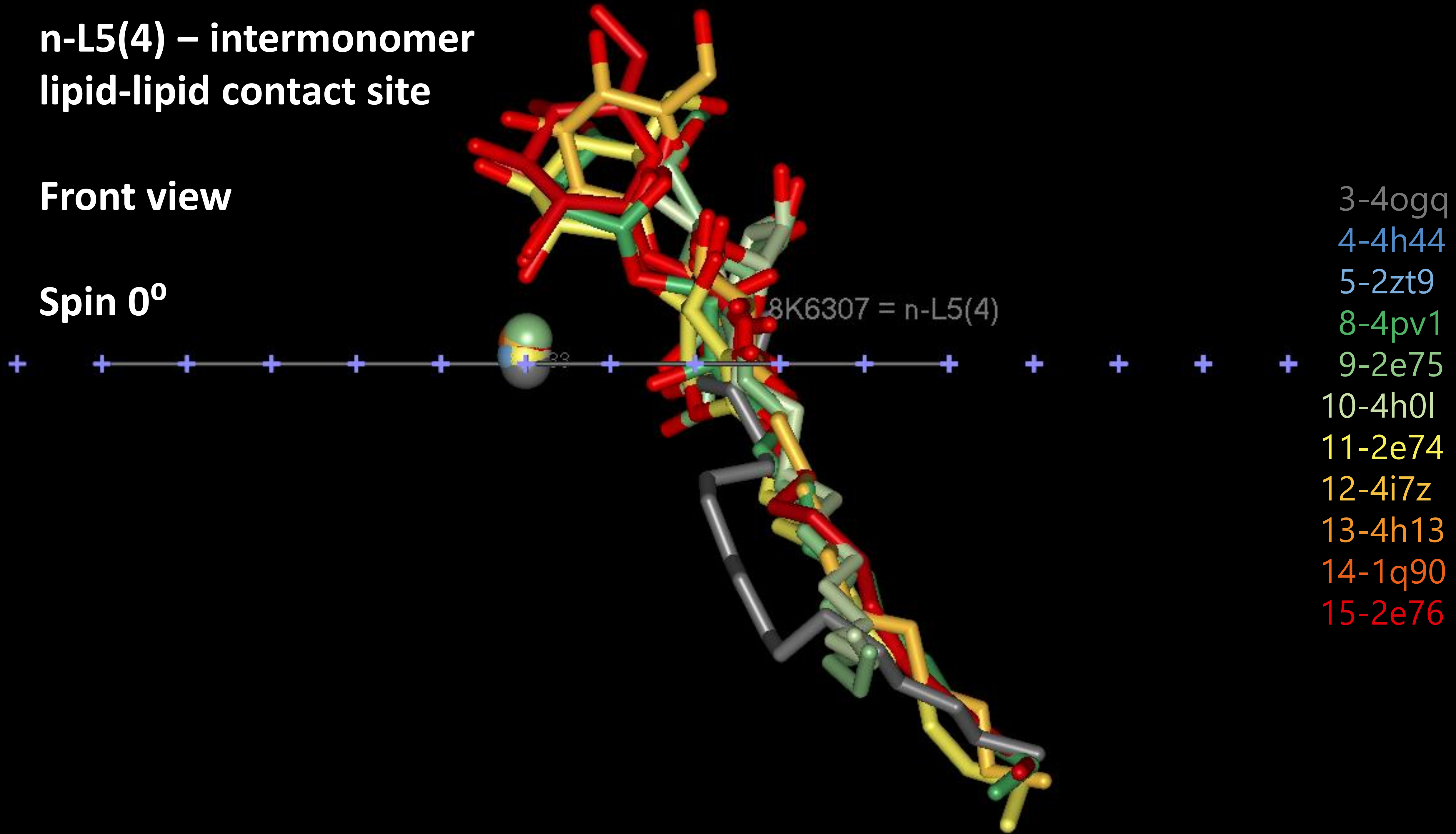

n-L5(4) – intermonomer lipid-lipid contact site

Front view

Spin 90º

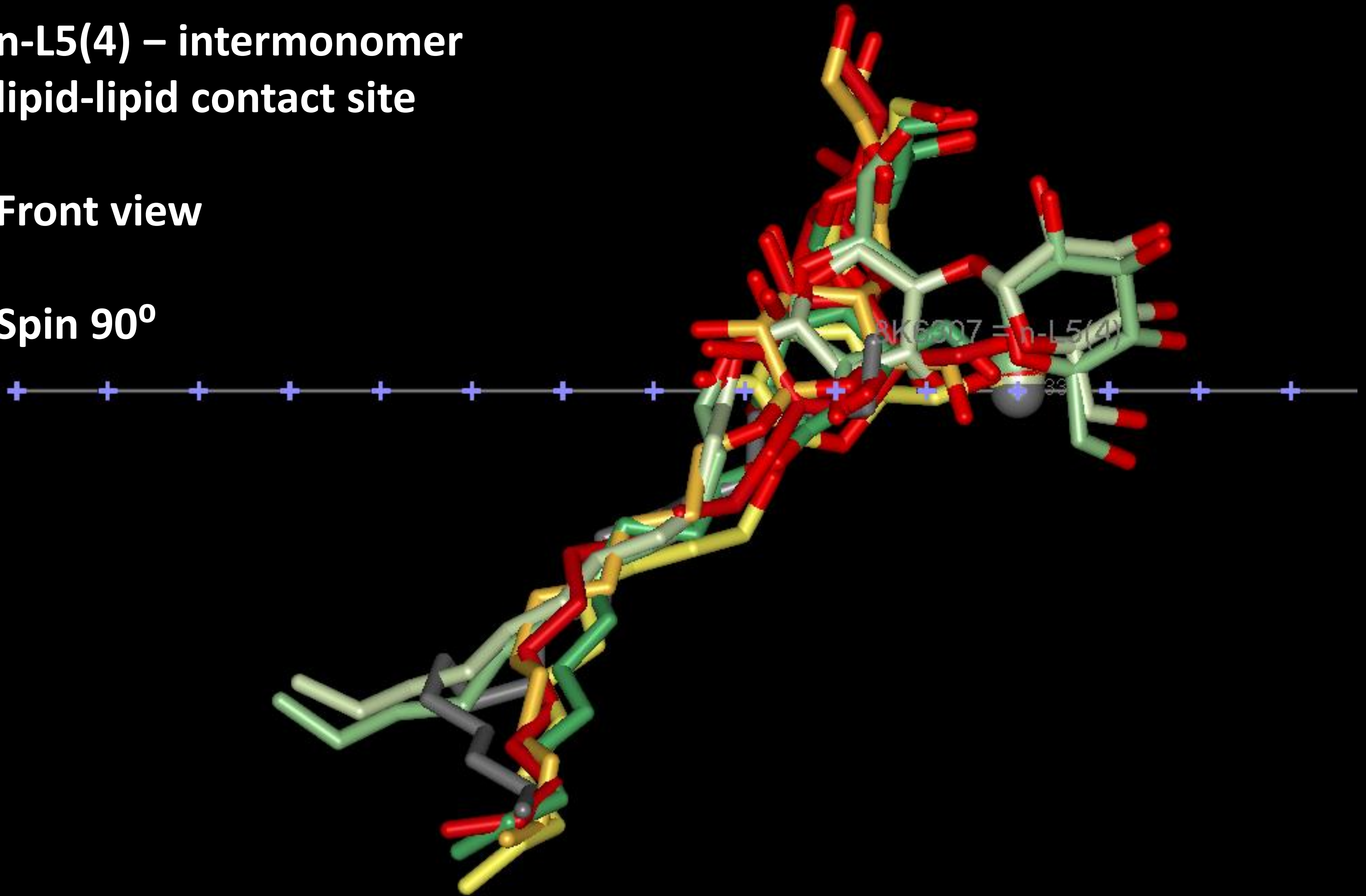

3-4ogq
4-4h44
5-2zt9
8-4pv1
9-2e75
10-4h0l
11-2e74
12-4i7z
13-4h13
14-1q90
15-2e76

n-L5(4) – intermonomer lipid-lipid contact site

front view

spin 270°

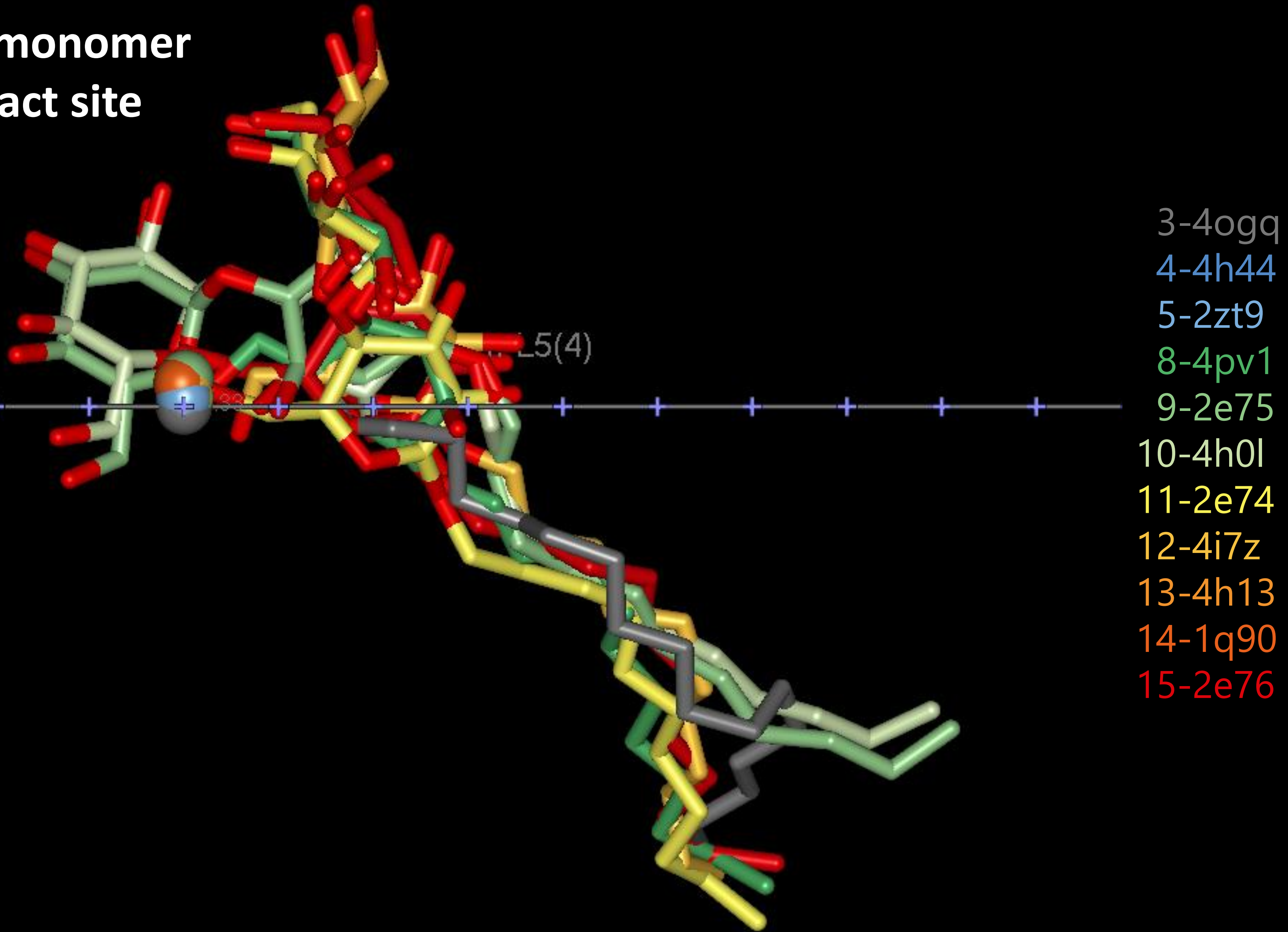

n-L5(3-5) sites - closer n-side view

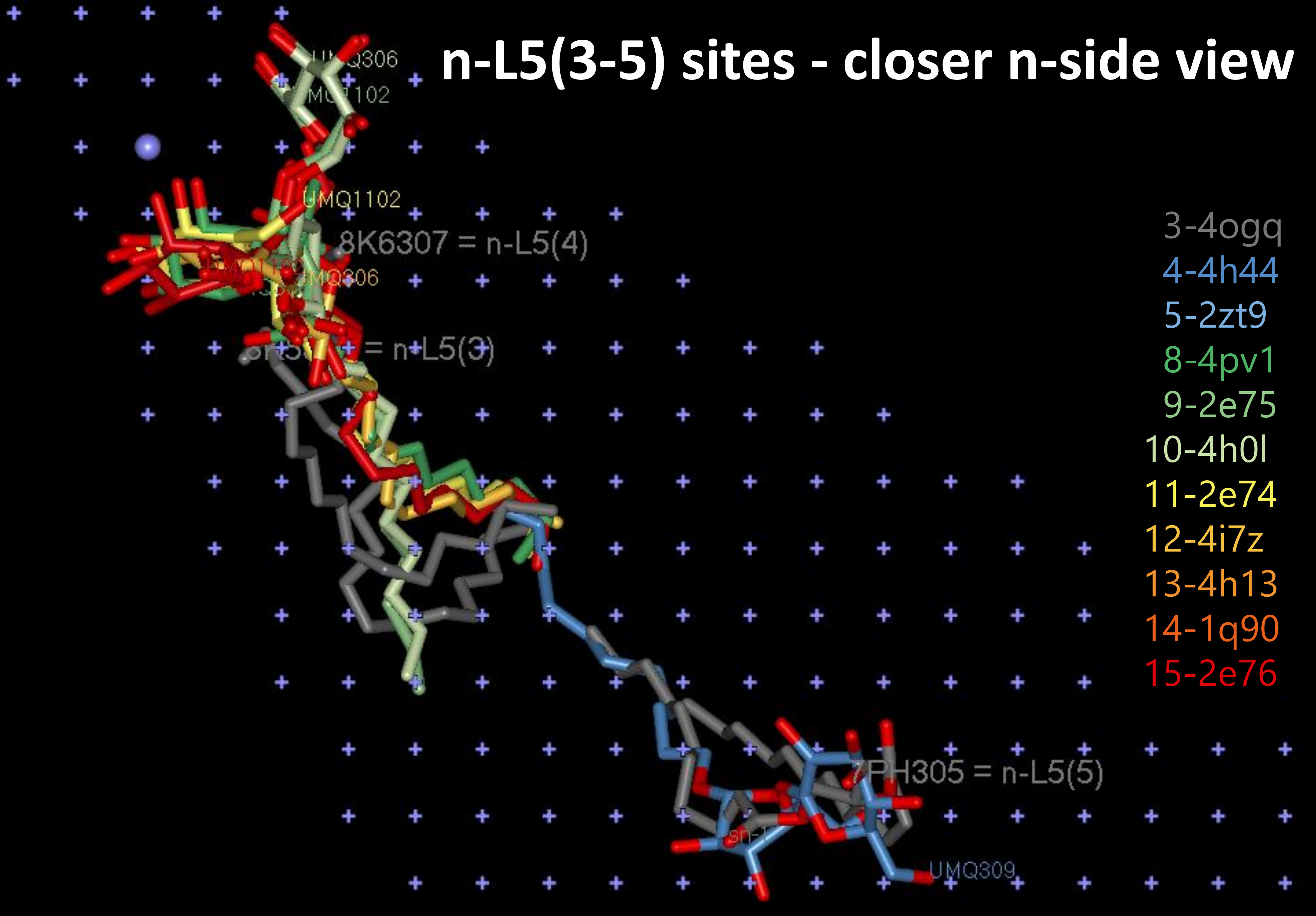

n-L5(3) and n-L5(5) with n-L5(4)

Front view

Spin 0°

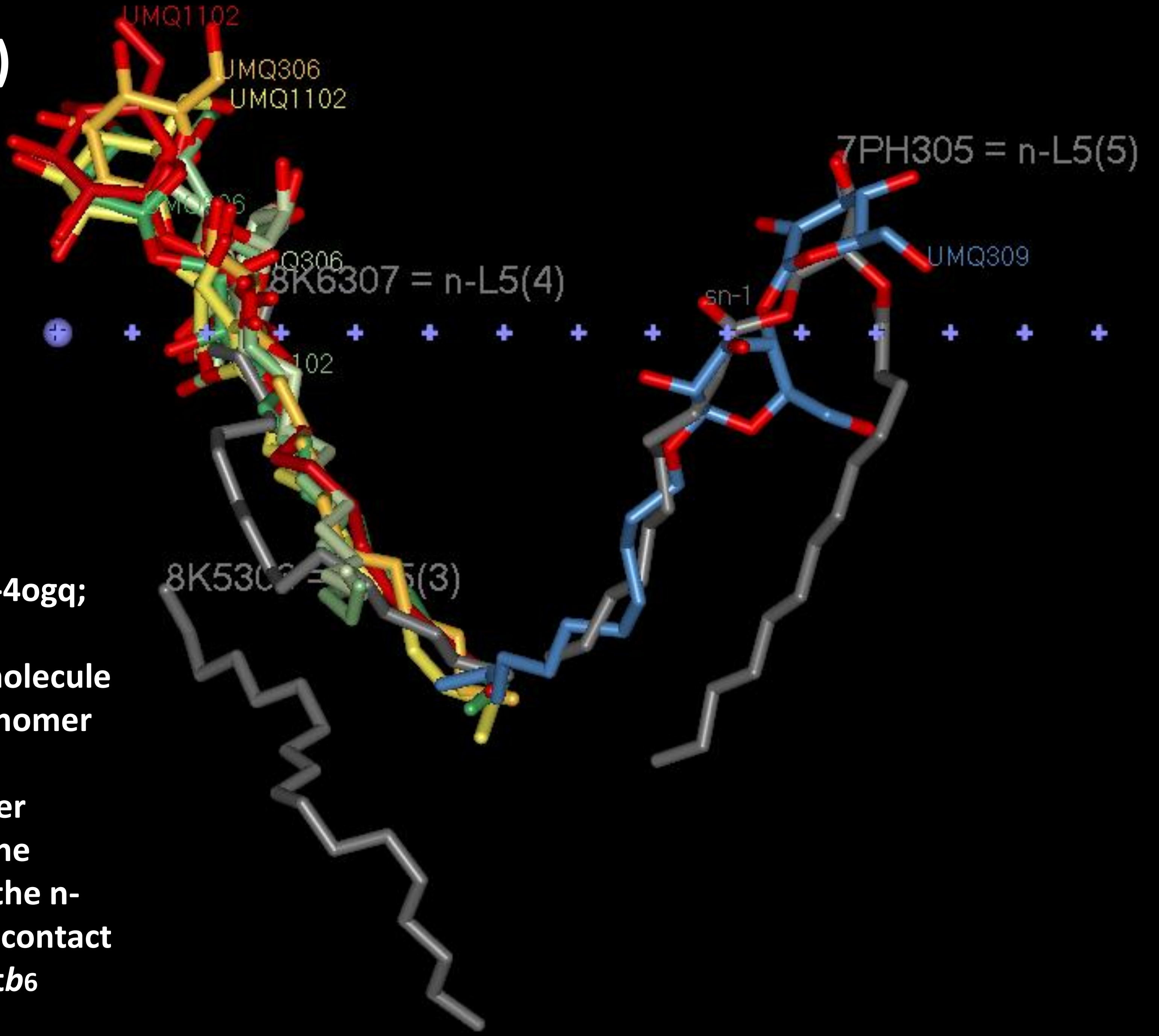

3-4ogq
4-4h44
5-2zt9
8-4pv1
9-2e75
10-4h0l
11-2e74
12-4i7z
13-4h13
14-1q90
15-2e76

- n-L5(3) is visible only in 3-4ogq;
- n-L5(5) is occupied by a molecule from the other cyt*b6f* monomer in 3-4ogq and 4-4h44;
- n-L5(5) is an intermonomer connecting site via both the chain-chain contact with the n-L5(4) detergent and head contact with the N-terminal of cyt*b6* subunit.

n-L5(3-5) front view

Spin 90°

- n-L5(5) is occupied only in structures 4ogq and 4h44 which have small p-gate width (see Table S2).
- A novelty reported here for the n-L5(5) site is that it corresponds to an equivalent site in cyt*bc*1 complex;
- The release of n-L5(5) site in structures with bigger dp-gate width is in accord with the idea that relative to cyt*bc*1, the specific conformational changes performed by cyt*b6f* during state transitions require higher flexibility of the complex at its n-side.

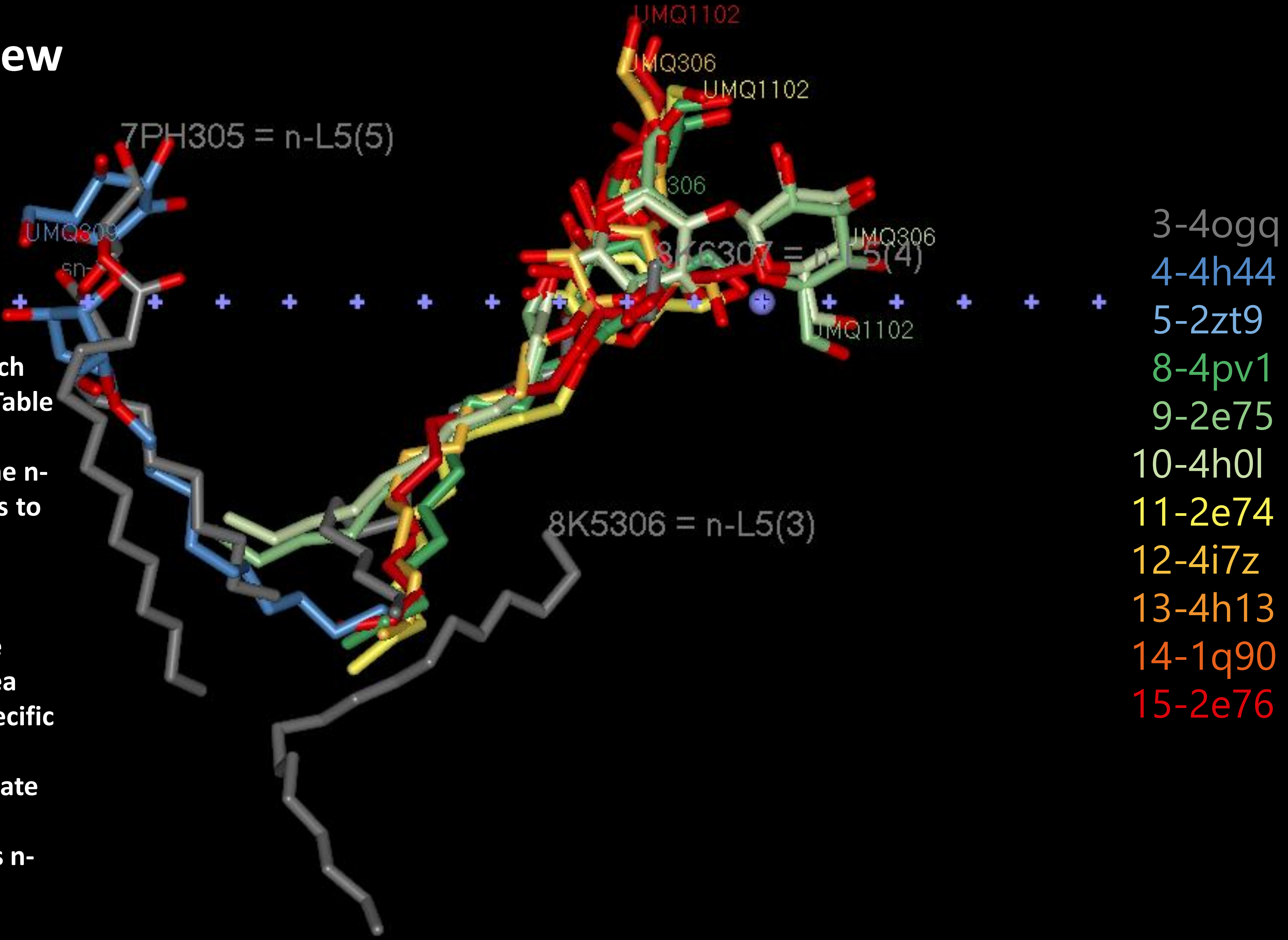

p-L5(4) site front view

Spin 270°

- p-L5(4) is close to the phytyl of Chla and may interact with it as seen in 4-4h44 and 13-4h13.

★ p-L5(4) has the same five structure-occupants as the p-L5(2). They are visible in one and the same five structures (3, 4, 8, 12 and 13)

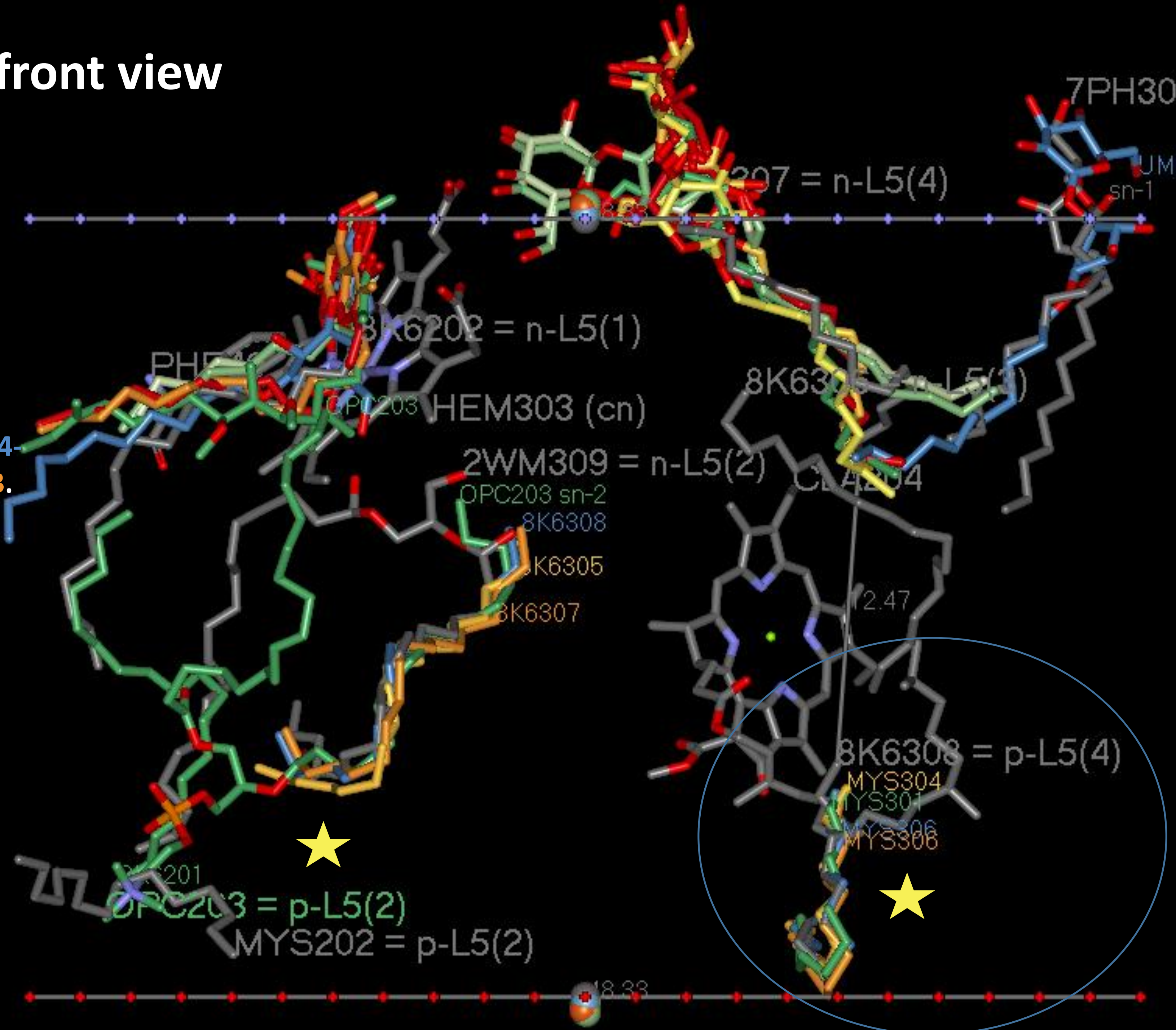

3-4ogq
4-4h44
5-2zt9
8-4pv1
9-2e75
10-4h0l
11-2e74
12-4i7z
13-4h13
14-1q90
15-2e76

L5 sites group and the added p-L4(1) and n-L3(1)

p-side view

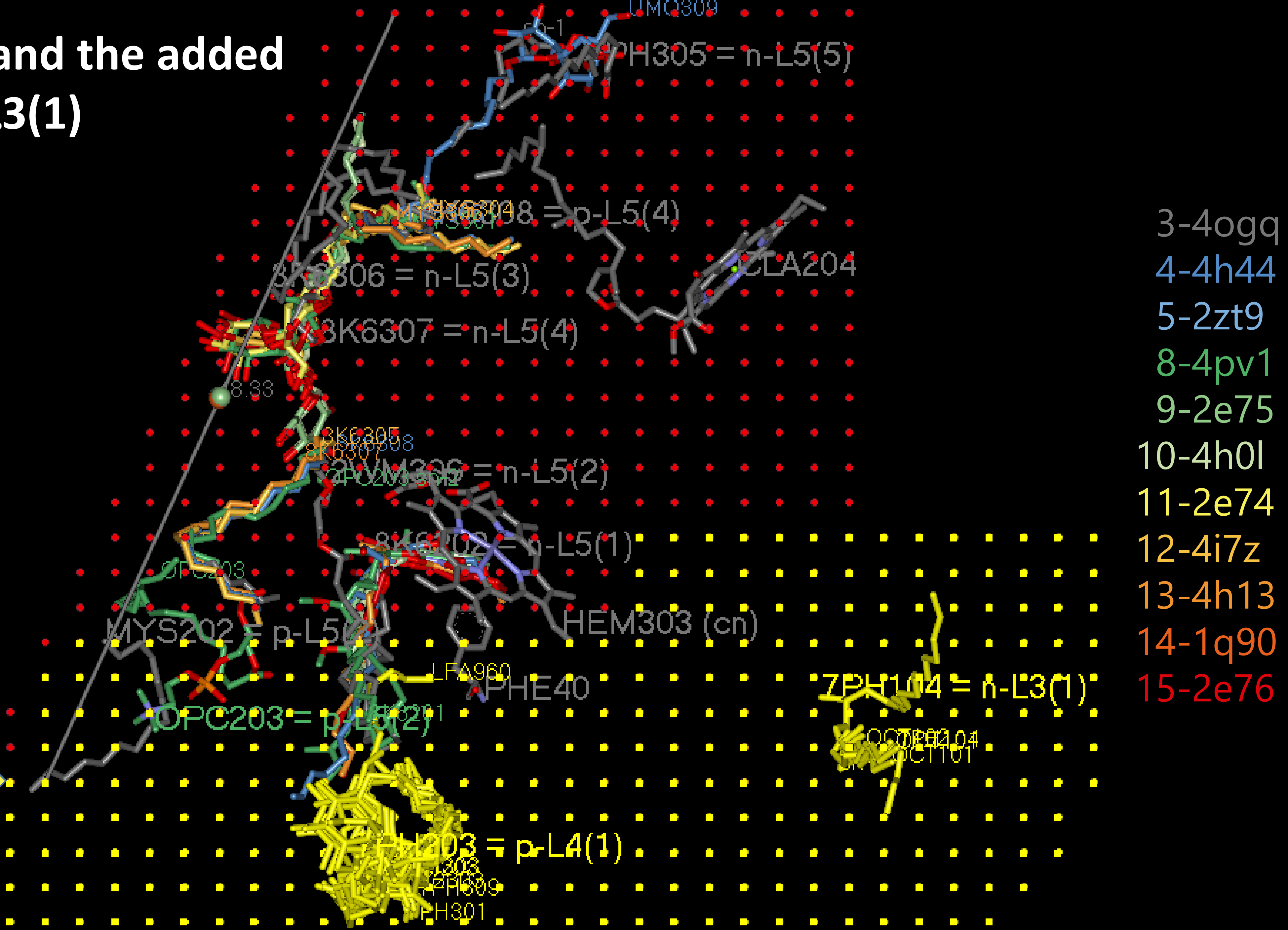

p-L4(1) and n-L3(1) are added to L5 since the same five structures as for n/p-L5(2) and p-L5(4) have the same occupants

L5-sites group and the added p-L4(1) and n-L3(1)

Front view

Spin 50º

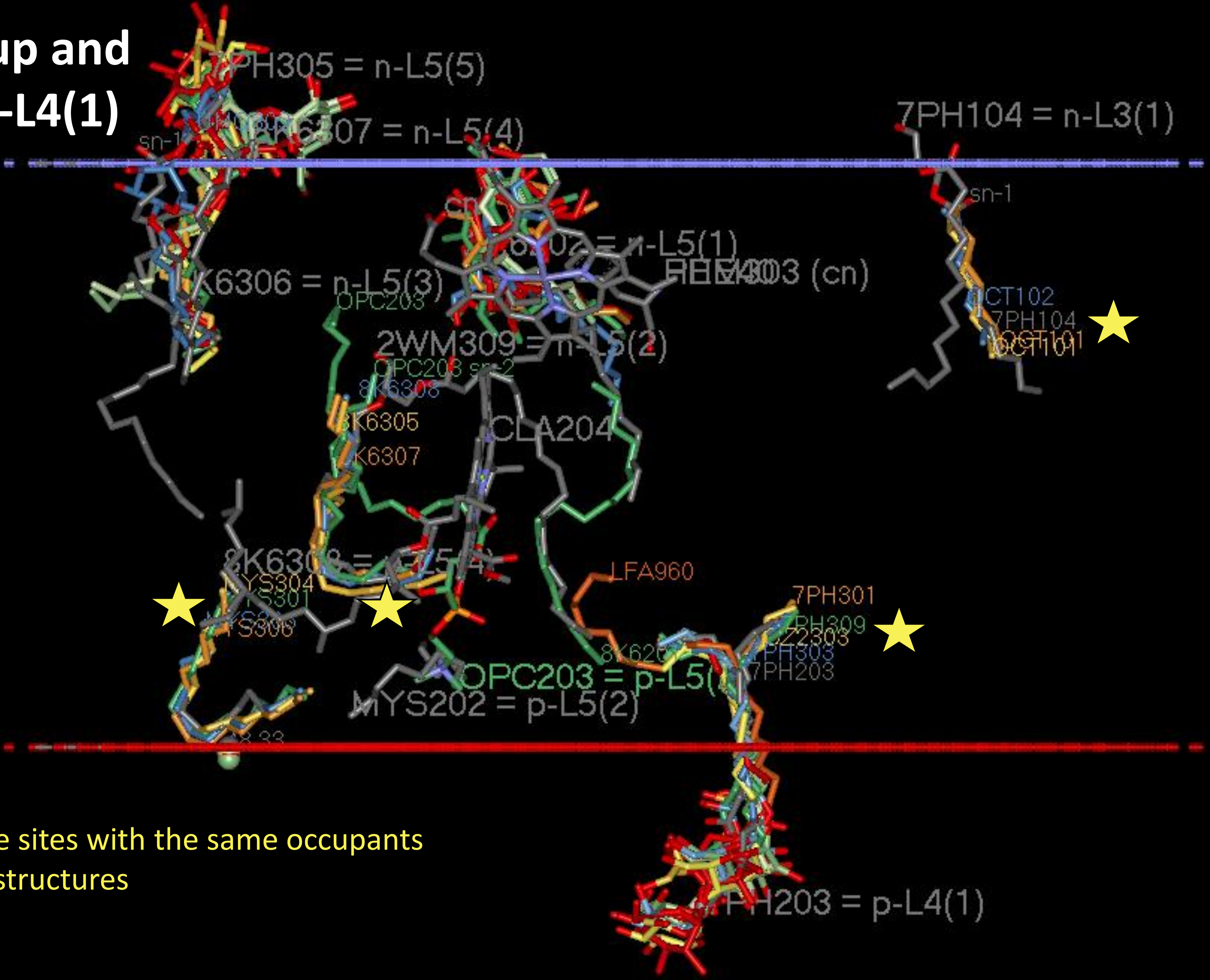

3-4ogq
4-4h44
5-2zt9
8-4pv1
9-2e75
10-4h0l
11-2e74
12-4i7z
13-4h13
14-1q90
15-2e76

★ indicates the sites with the same occupants in five-four structures

Cyt*b*6*f* lipid binding sites and their dynamics during induction of transition to State 1

Starting from the 4ogq structure, the induction of the transition to state 1 is modeled by the structure 1vf5 and then the structure 2d2c (see their addition in the legend in right; -s and –b mean small and big p-gate and dn-distance, respectively). Relative to the 4ogq structure, these structures have decreasing hydrophobic thickness (from 30.6 Å to 29.8 Å and 28.6 Å), average p-gate width and n-side distance (see Table S2). These structures are asymmetric dimers, each containing only two lipids per monomer. The stronger changes in the hydrophobic thickness and the other two distances for these two structures compared, to all others during the induction of a transition to state 1 are paralleled by significant conformational changes of the Phe124 from the *fg*-loop region. The Phe124 is progressively rotated from a down- to an up position relative to the n-side membrane interface plane. It is concomitantly moved from a protein-surface-exposed (membrane-buried) to a protein-buried position. The protein-buried movement is directed towards the Leu106 of the core helix B of cyt*b*6 subunit and the residue Ile211 from the C-terminal region of cyt*b*6 subunit. The Phe/Tyr124(CA) – Leu106(O) and Phe/Tyr124(CE2) – Ile211(CD1) distances are almost the same in all structures except these two. These pair distances decreased from around 8.7 Å and 20 Å to 4.5 Å and 3.4 Å in structure 1-2d2c, respectively. The Phe124 pose and the Phe40-Phe124 distance become very similar to those in the analogous cyt*bc*1 structures [Vladkova 2016]. The changes in lipids within these two structures to adapt to the conformational changes in the protein are shown below. First are the n-L4(1-3) sites and then the n/p-L5(1-2) sites.

1-2d2c-s
2-1vf5-s
3-4ogq
4-4h44
5-2zt9
6-1vf5-b
7-2d2c-b
8-4pv1
9-2e75
10-4h0l
11-2e74
12-4i7z
13-4h13
14-1q90
15-2e76

n-L4(1)-site (SQDG) in all 15 monomeric cyt*b₆f* structures

p-side view

- n-L4(1) site is occupied in all 15 monomeric structures. It is a SQDG lipid in almost all structures (11), except in the asymmetric dimers 1vf5 and 2d2c, wherein it is a DOPC lipid.
- There is a clear shift of their chains to the lipid-lipid dimer interface, closer to the chains of the two detergents at n-L4(2) and n-L4(3) positions (see above).

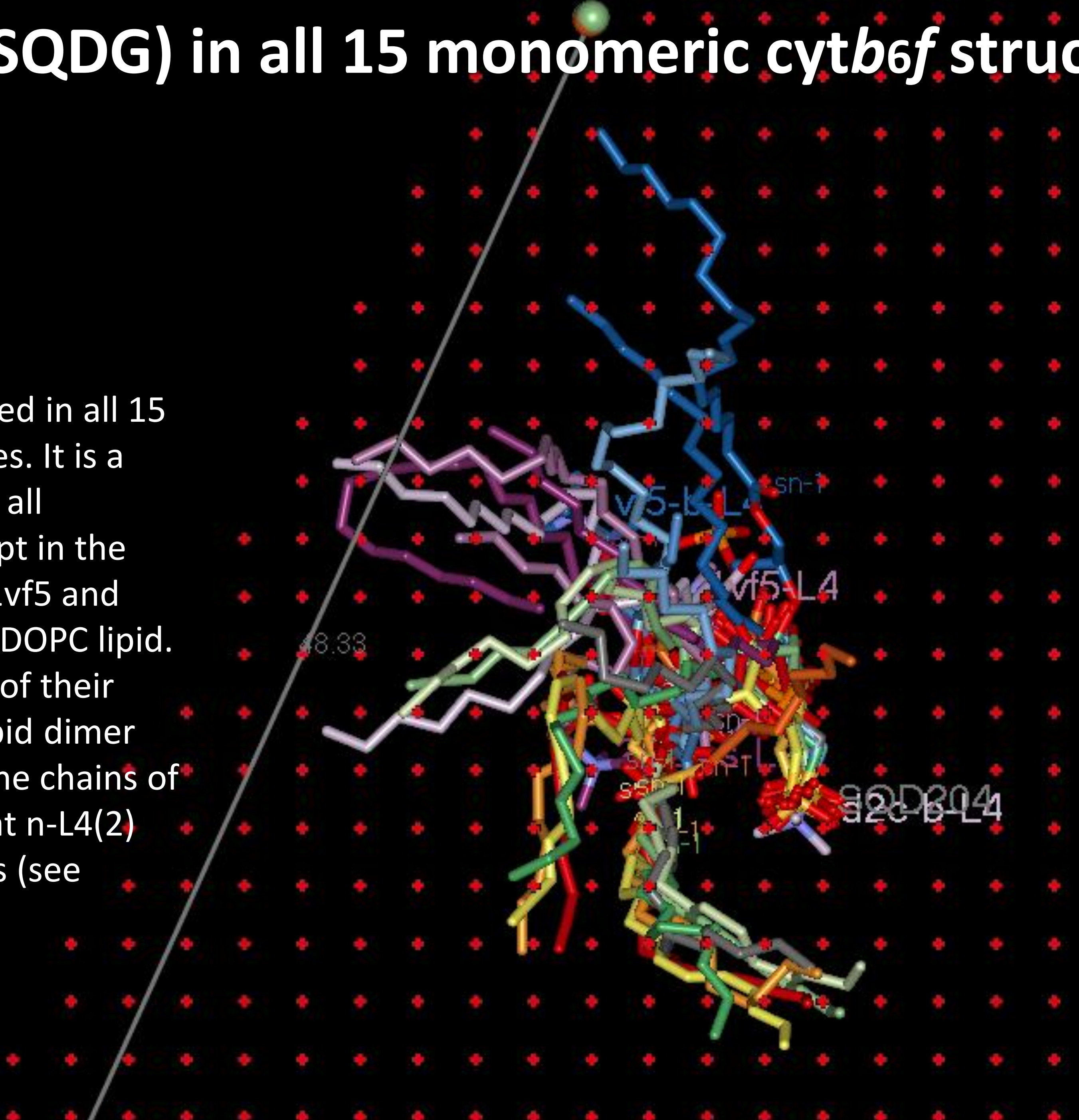

1-2d2c-s
2-1vf5-s
3-4ogq
4-4h44
5-2zt9
6-1vf5-b
7-2d2c-b
8-4pv1
9-2e75
10-4h0l
11-2e74
12-4i7z
13-4h13
14-1q90
15-2e76

n-L4(1)-site (SQDG) in all 15 monomeric cyt*b6f* structures

n-side view

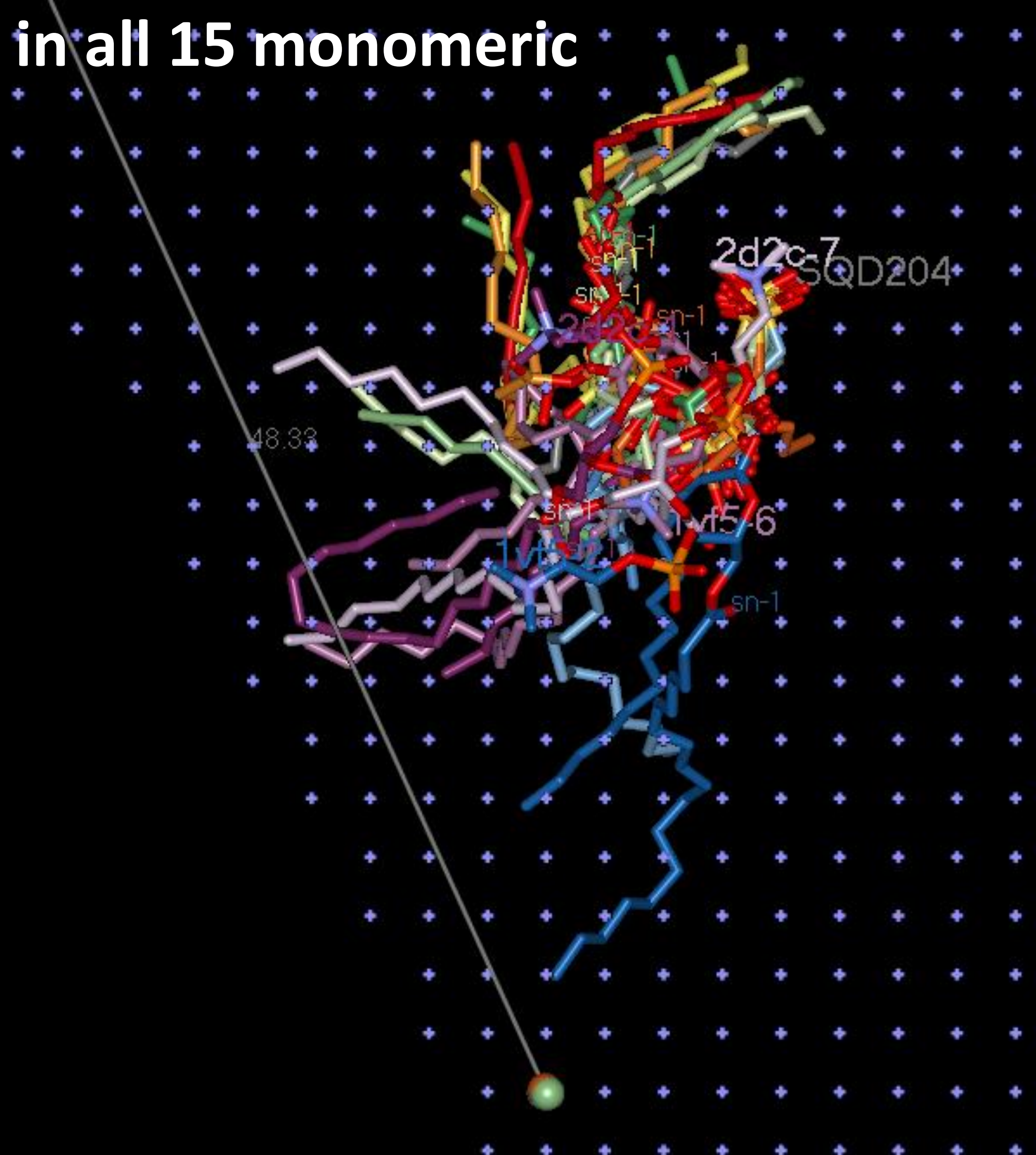

1-2d2c-s
2-1vf5-s
3-4ogq
4-4h44
5-2zt9
6-1vf5-b
7-2d2c-b
8-4pv1
9-2e75
10-4h0l
11-2e74
12-4i7z
13-4h13
14-1q90
15-2e76

n-L4(1)-site (SQDG) in all 15 monomeric cyt*b6f* structures

Front view

Spin 0º

- **There is also displacement and bigger head group rotation for the DOPC in 2d2c and 1vf5 as compared to the head group mobility of SQDG in the other 11 structures.**

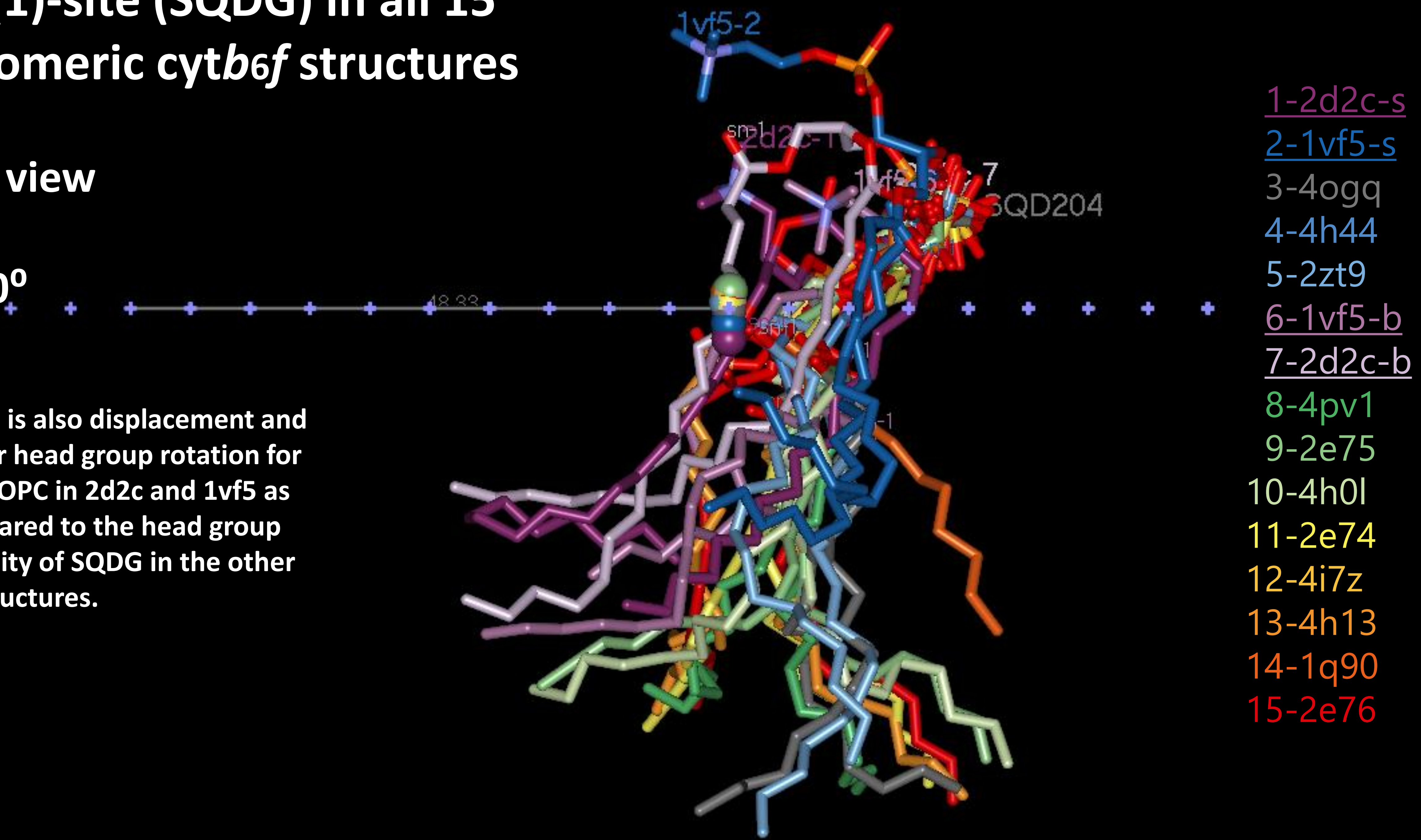

n-L4(1)-site (SQDG) in all 15 monomeric cytb_6f structures

Front view

Spin 90°

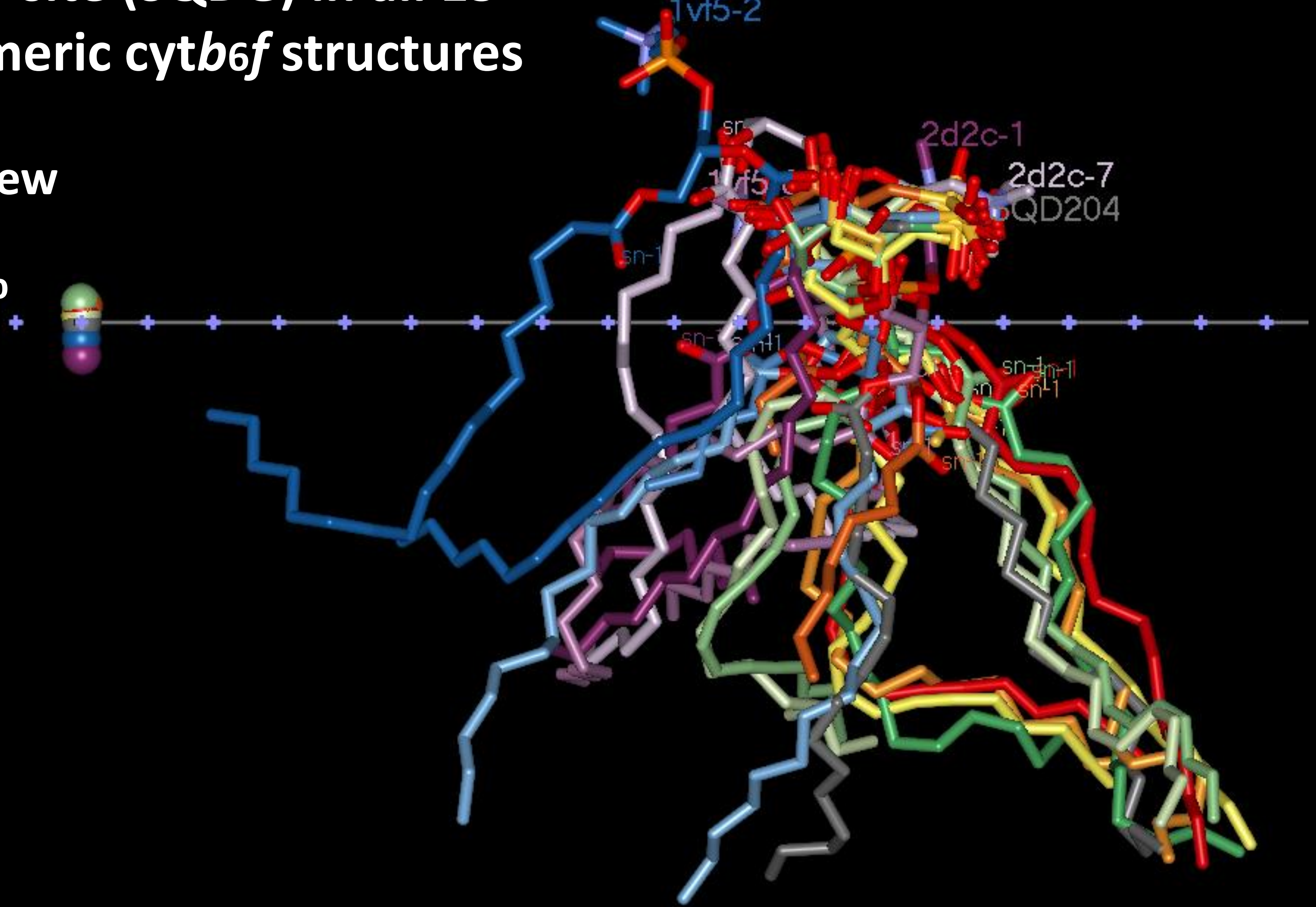

1-2d2c-s
2-1vf5-s
3-4ogq
4-4h44
5-2zt9
6-1vf5-b
7-2d2c-b
8-4pv1
9-2e75
10-4h0l
11-2e74
12-4i7z
13-4h13
14-1q90
15-2e76

n-L4(1-3) sites in the cyt*b6f* structures 3, 4, 5 and 8

p-side view

For a better viewing the location of 1vf5 and 2d2c lipids in the region of the n-L4(1-3) sites, only the four structures 3, 4, 5 and 8 (see the legend) are used for comparison. These four structures (see the legend) have the closest values for the p-side lateral gate (p-gate) to those for the structures 1vf5 and 2d2c (Table S2).

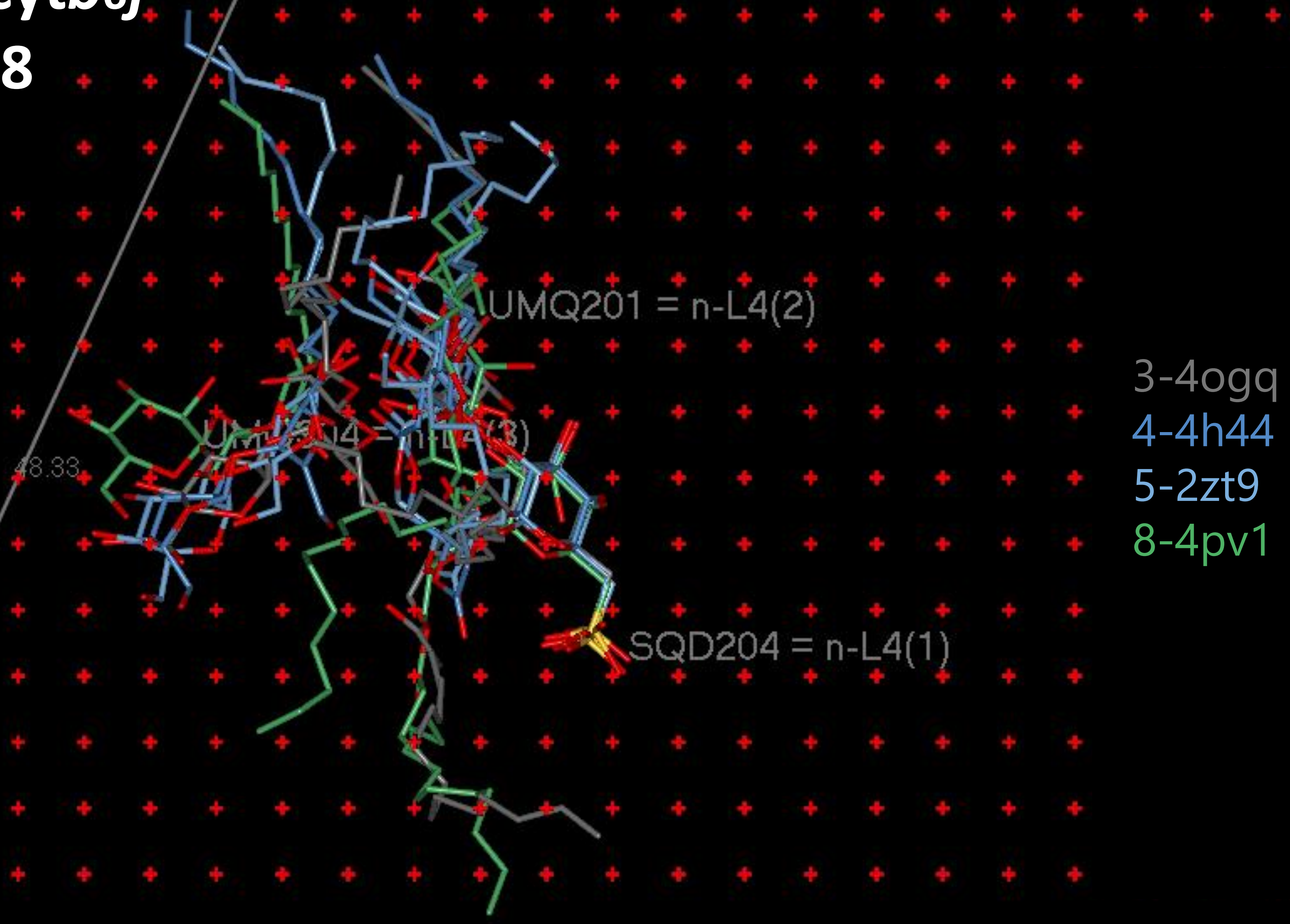

n-L4(1-3) sites in the cyt*b6f* structures 3, 4, 5 and 8

n-side view

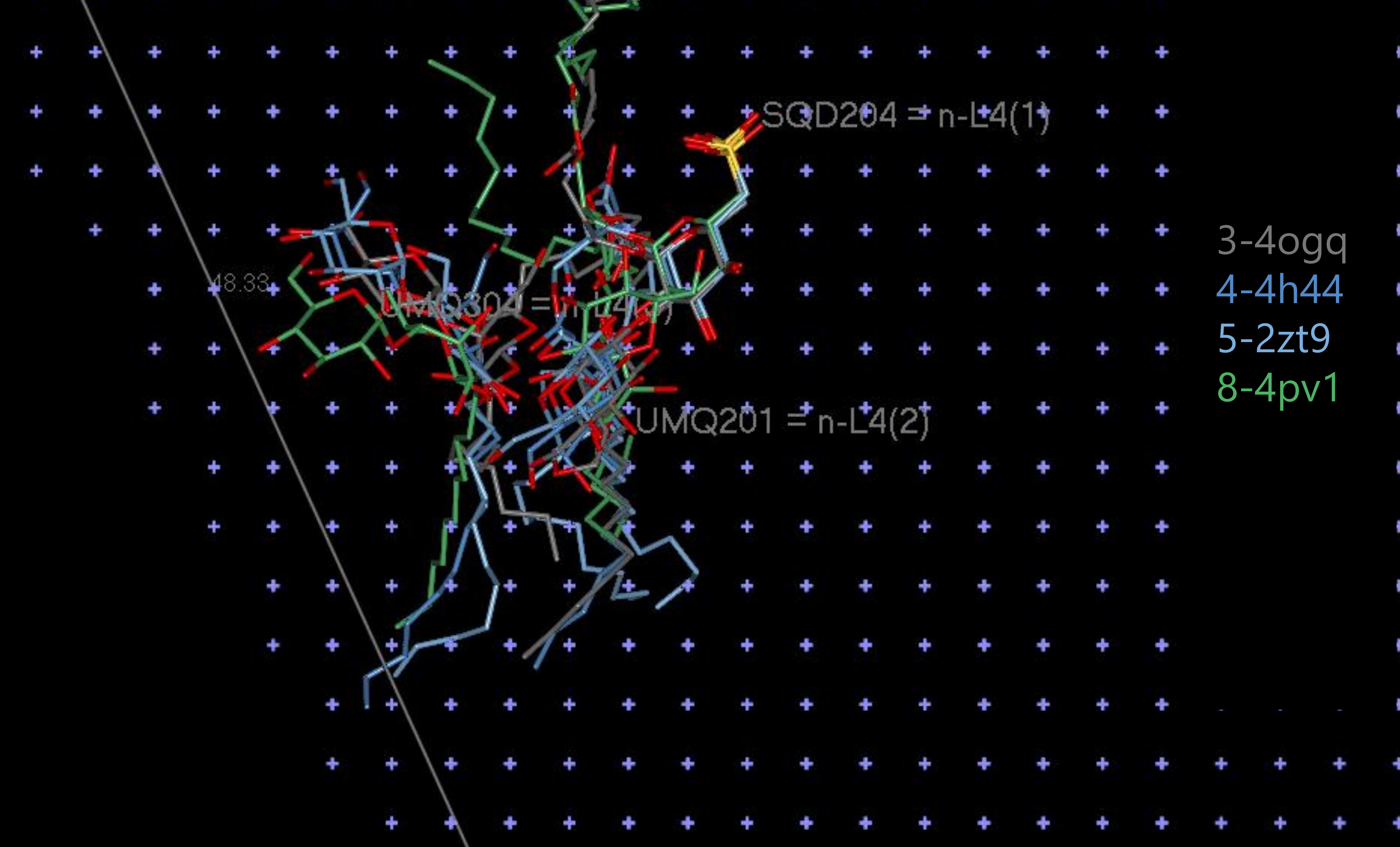

n-L4(1-3) sites in structures 3, 4, 5 and 8 with the added 1vf5

n-side view

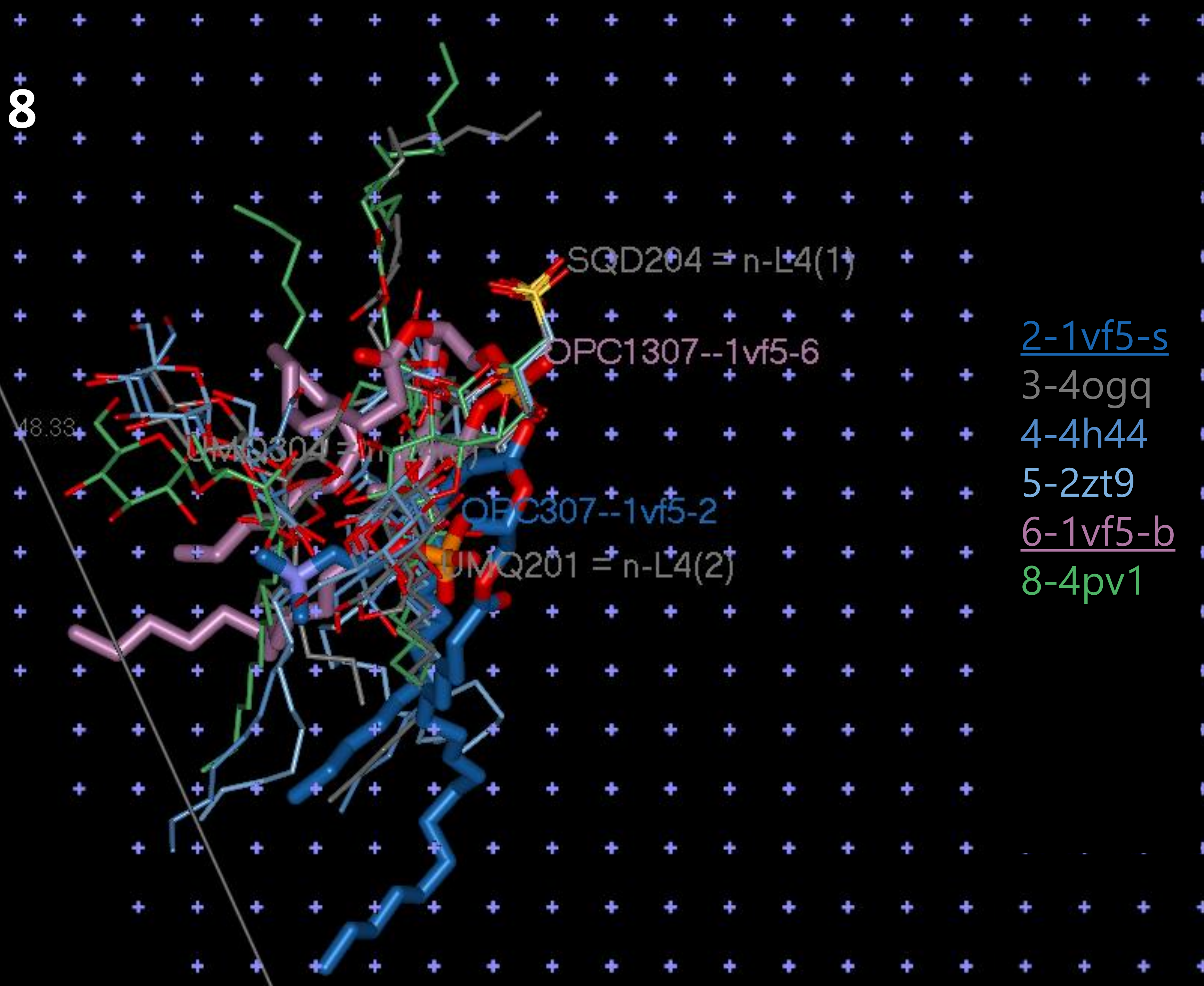

n-L4(1-3) sites in structures 3, 4, 5 and 8 with the added 2d2c

n-side view

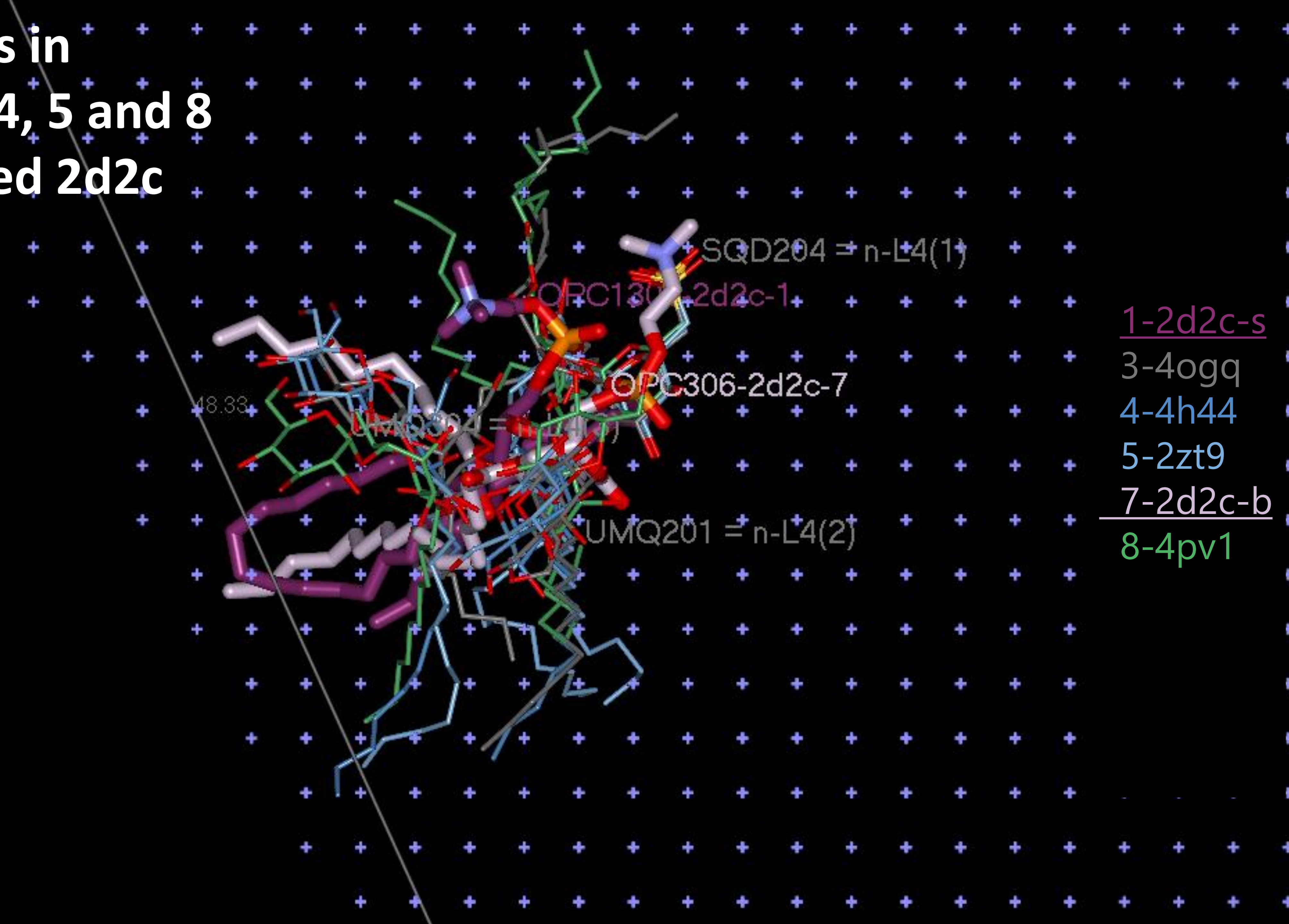

n-L4(1-3) sites in structures 3, 4, 5 and 8 with the added 1vf5 and 2d2c

n-side view

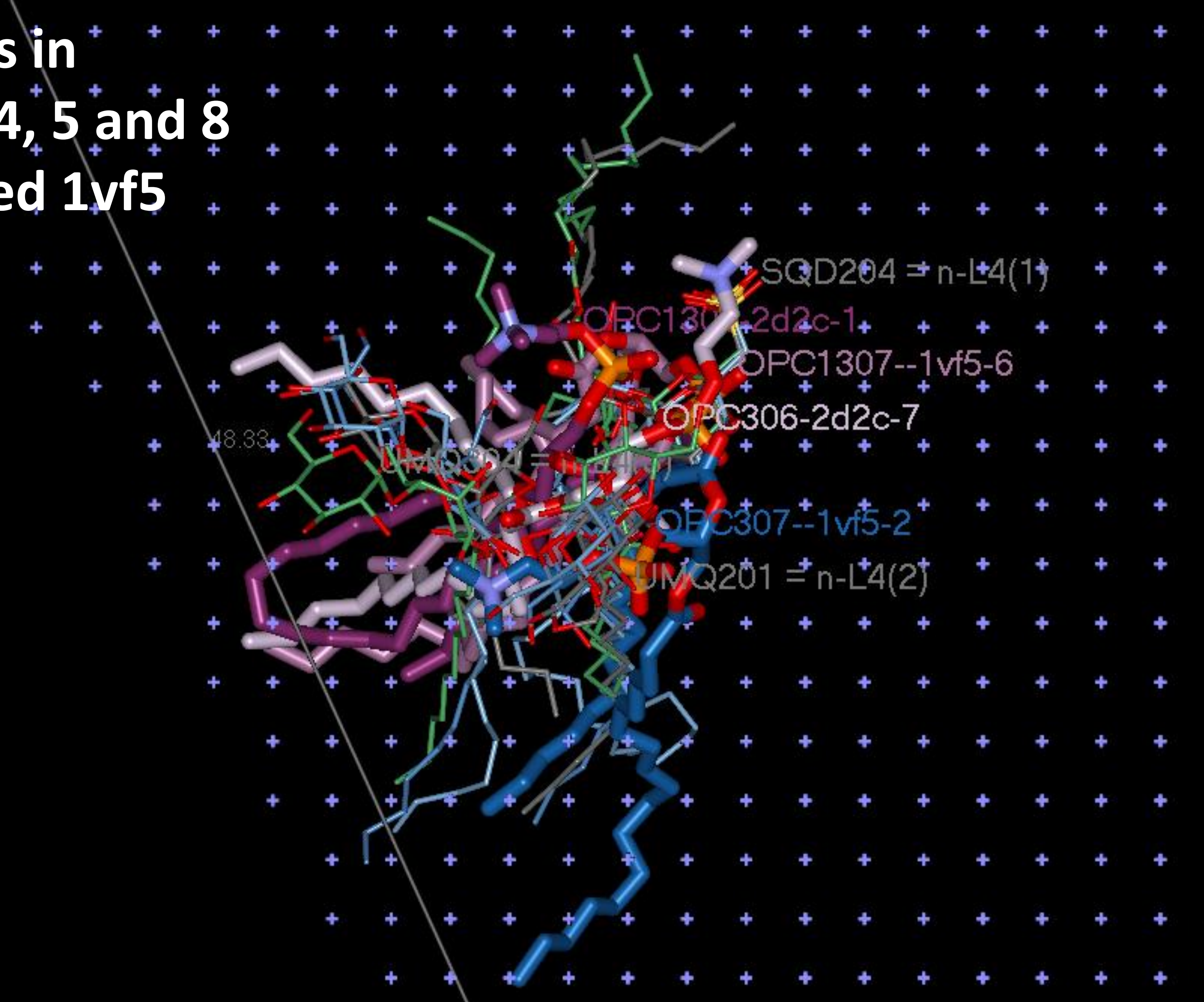

1-2d2c-s
2-1vf5-s
3-4ogq
4-4h44
5-2zt9
6-1vf5-b
7-2d2c-b
8-4pv1

n-L4(1-3) sites in structures 3, 4, 5 and 8

Front view

Spin 0º

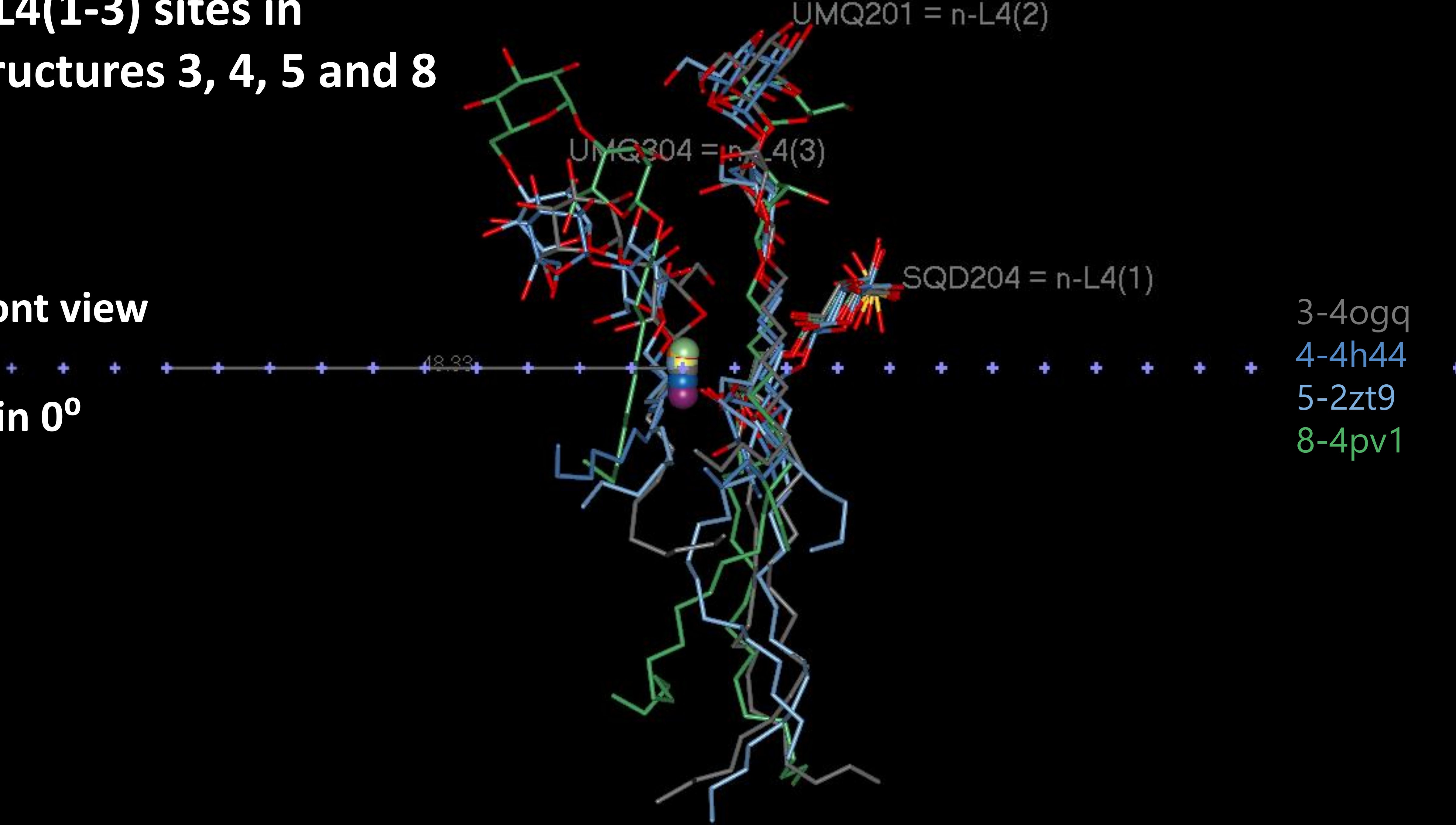

3-4ogq
4-4h44
5-2zt9
8-4pv1

n-L4(1-3) sites in structures 3, 4, 5 and 8 with the added 1vf5

Front view

Spin 0º

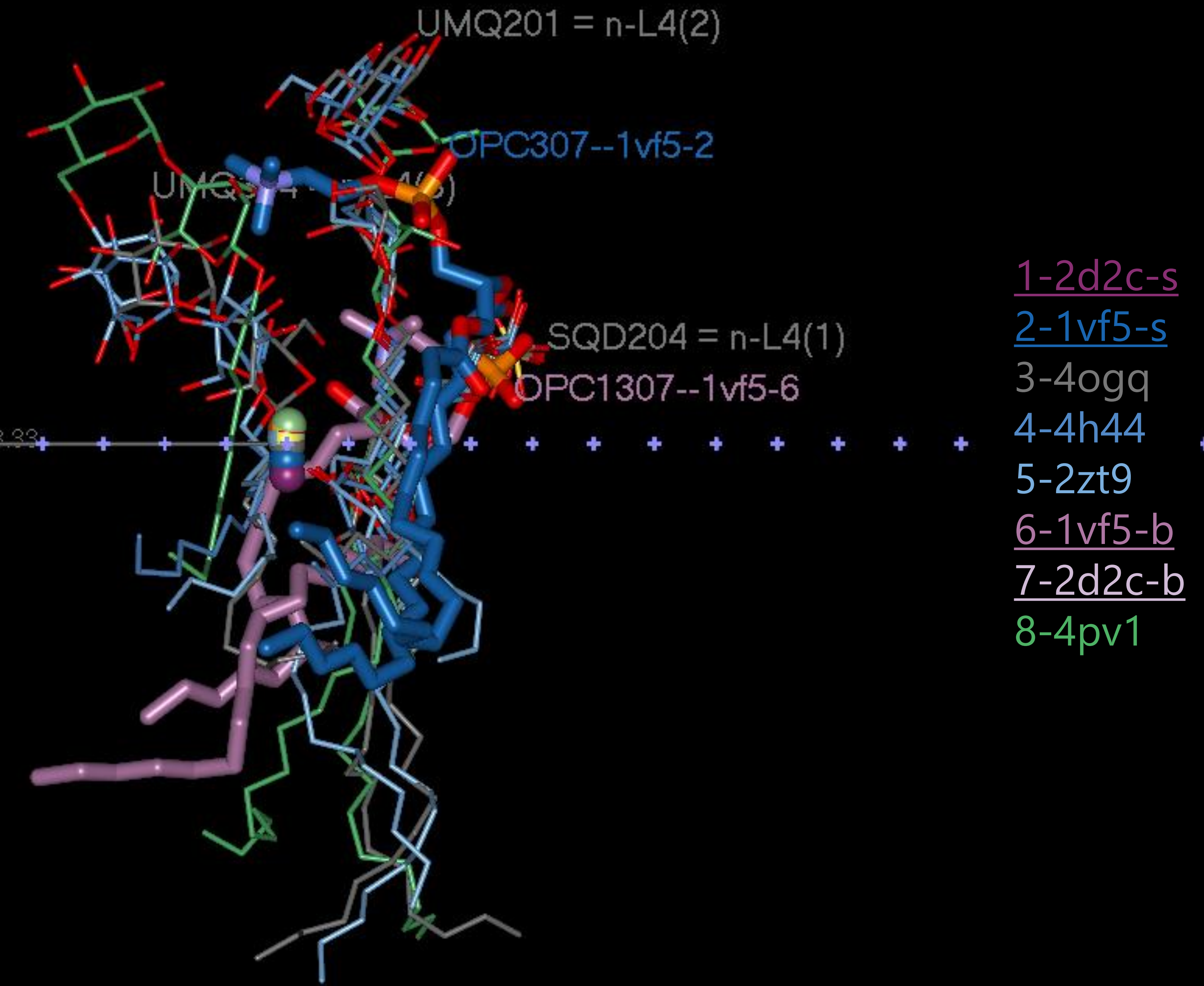

n-L4(1-3) sites in structures 3, 4, 5 and 8 with the added 2d2c

Front view

Spin 0º

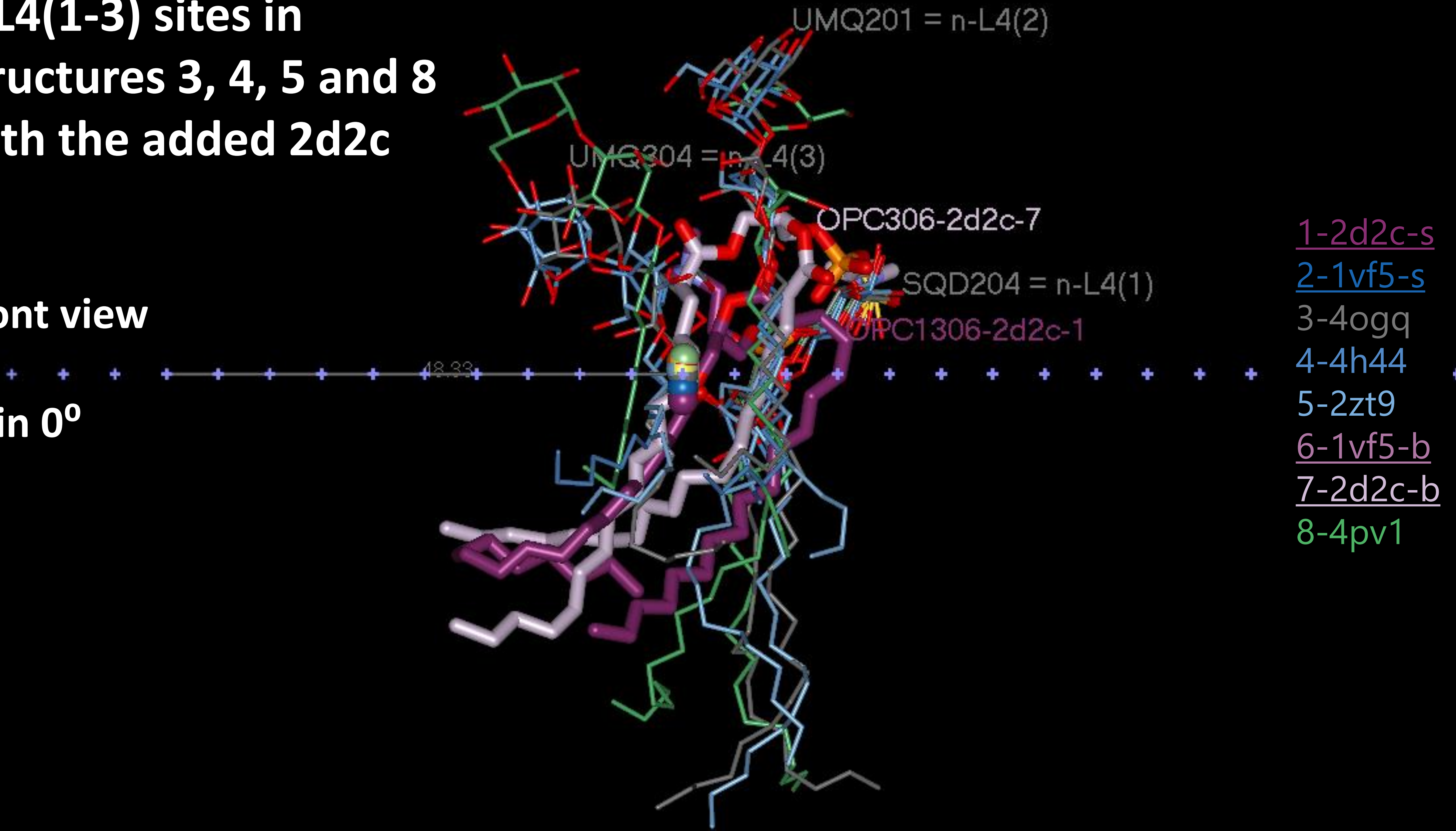

n-L4(1-3) sites in structures 3, 4, 5 and 8 with the added 1vf5 and 2d2c

Front view

Spin 0º

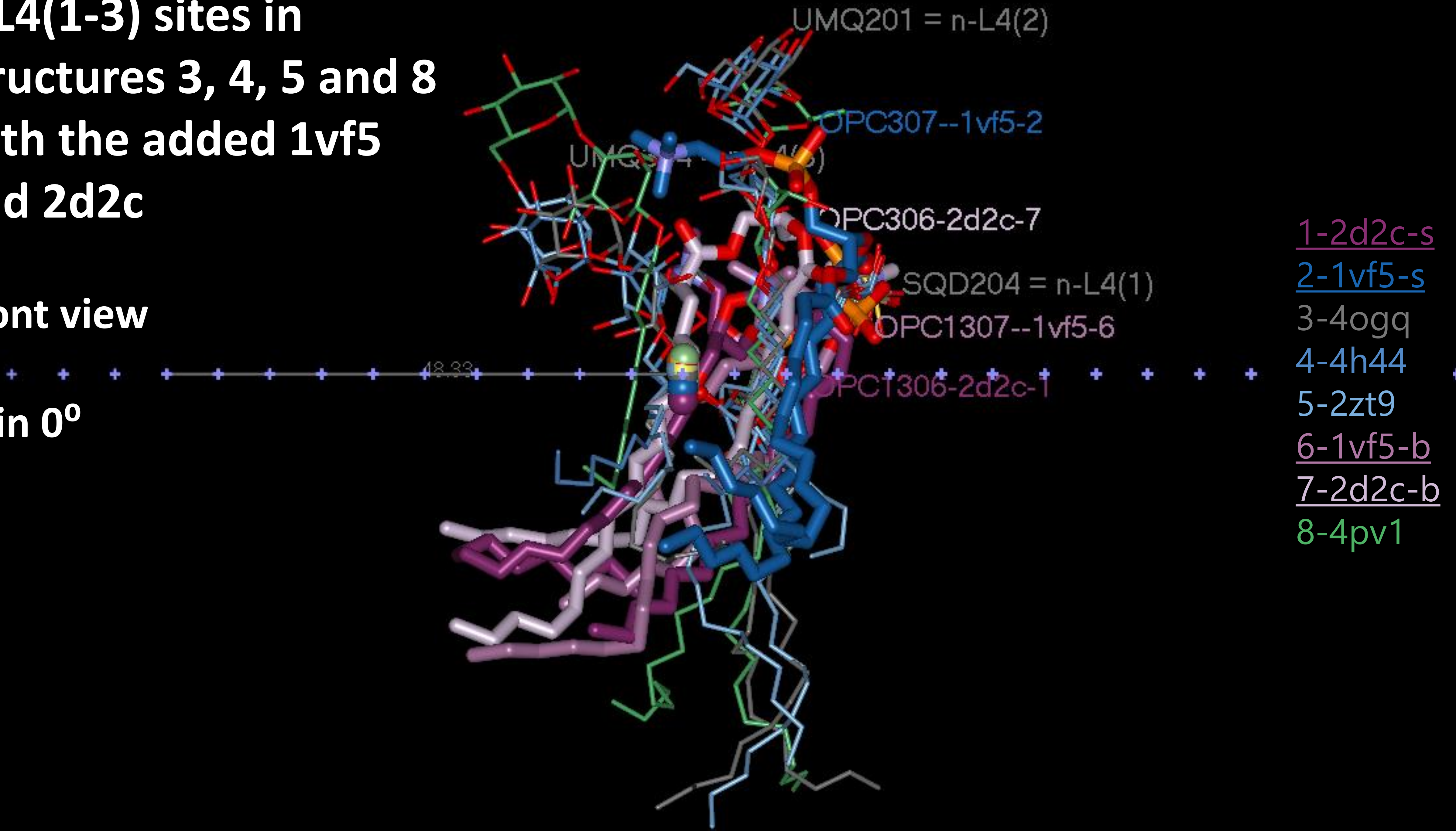

n-L4(1-3) sites in structures 3, 4, 5 and 8

Front view

Spin 90º

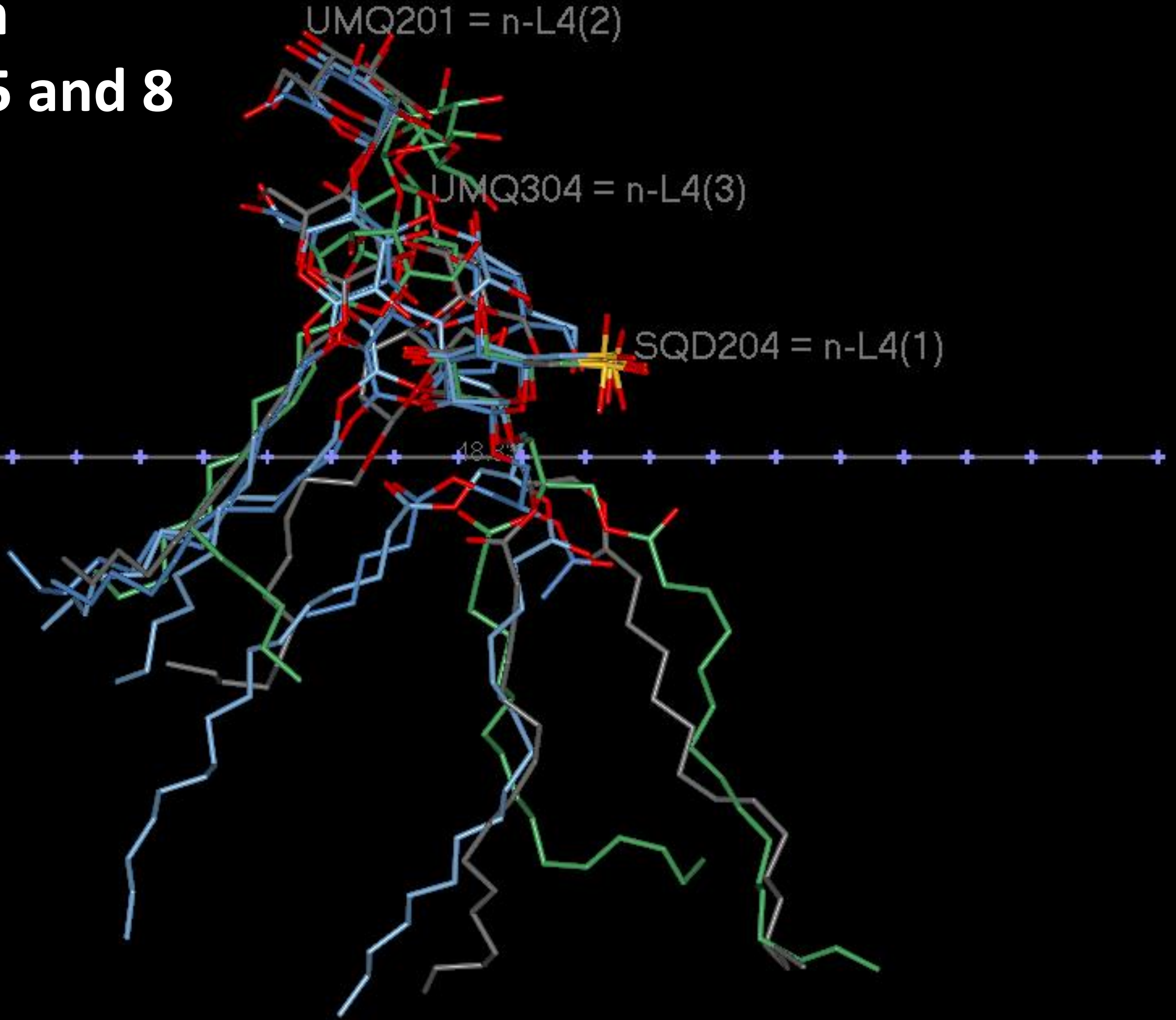

1-2d2c-s
2-1vf5-s
3-4ogq
4-4h44
5-2zt9
6-1vf5-b
7-2d2c-b
8-4pv1

n-L4(1-3) site in structures 3, 4, 5 and 8 with the added 1vf5

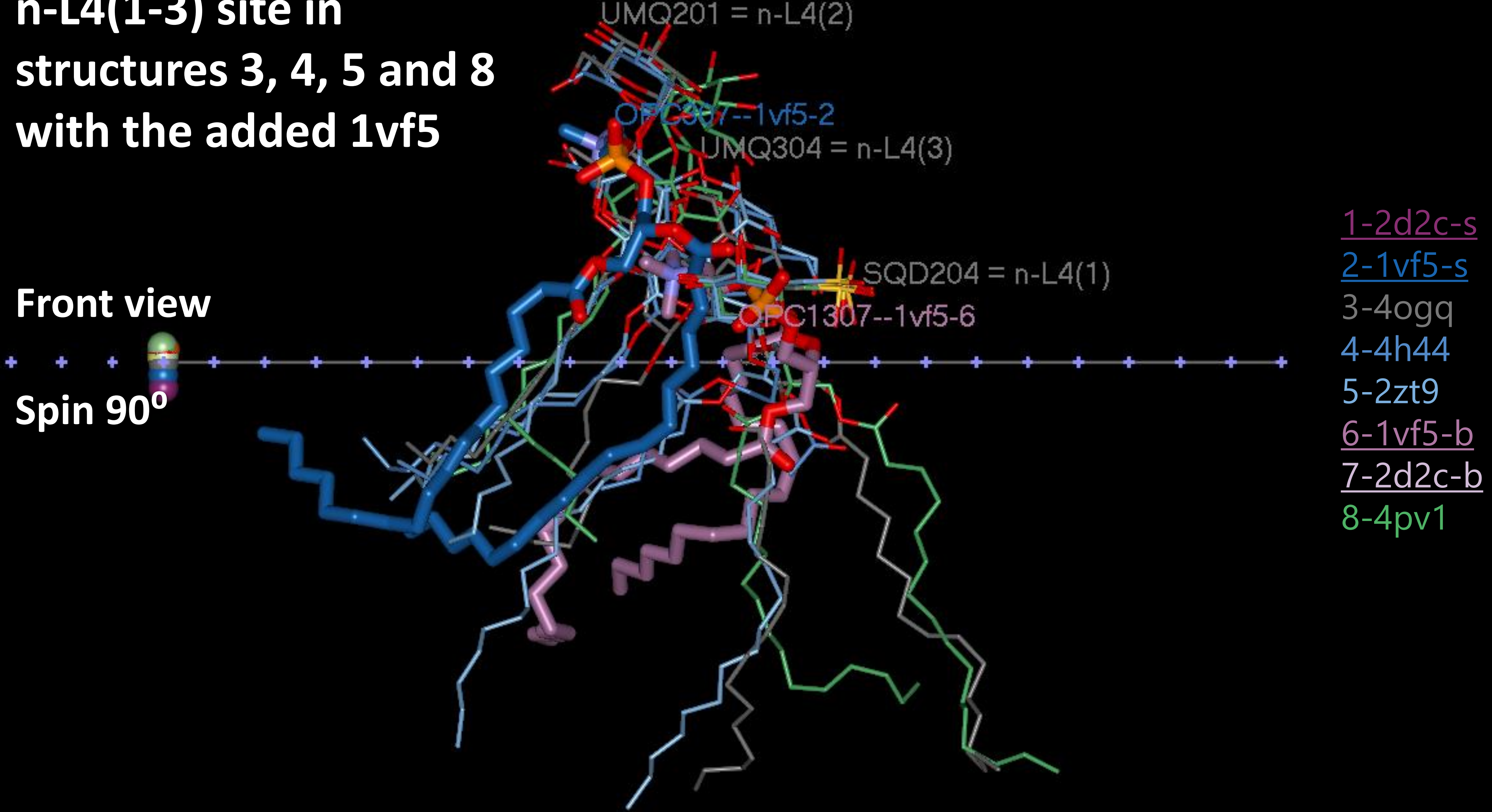

n-L4(1-3) sites in structures 3, 4, 5 and 8 with the added 2d2c

Front view

Spin 90°

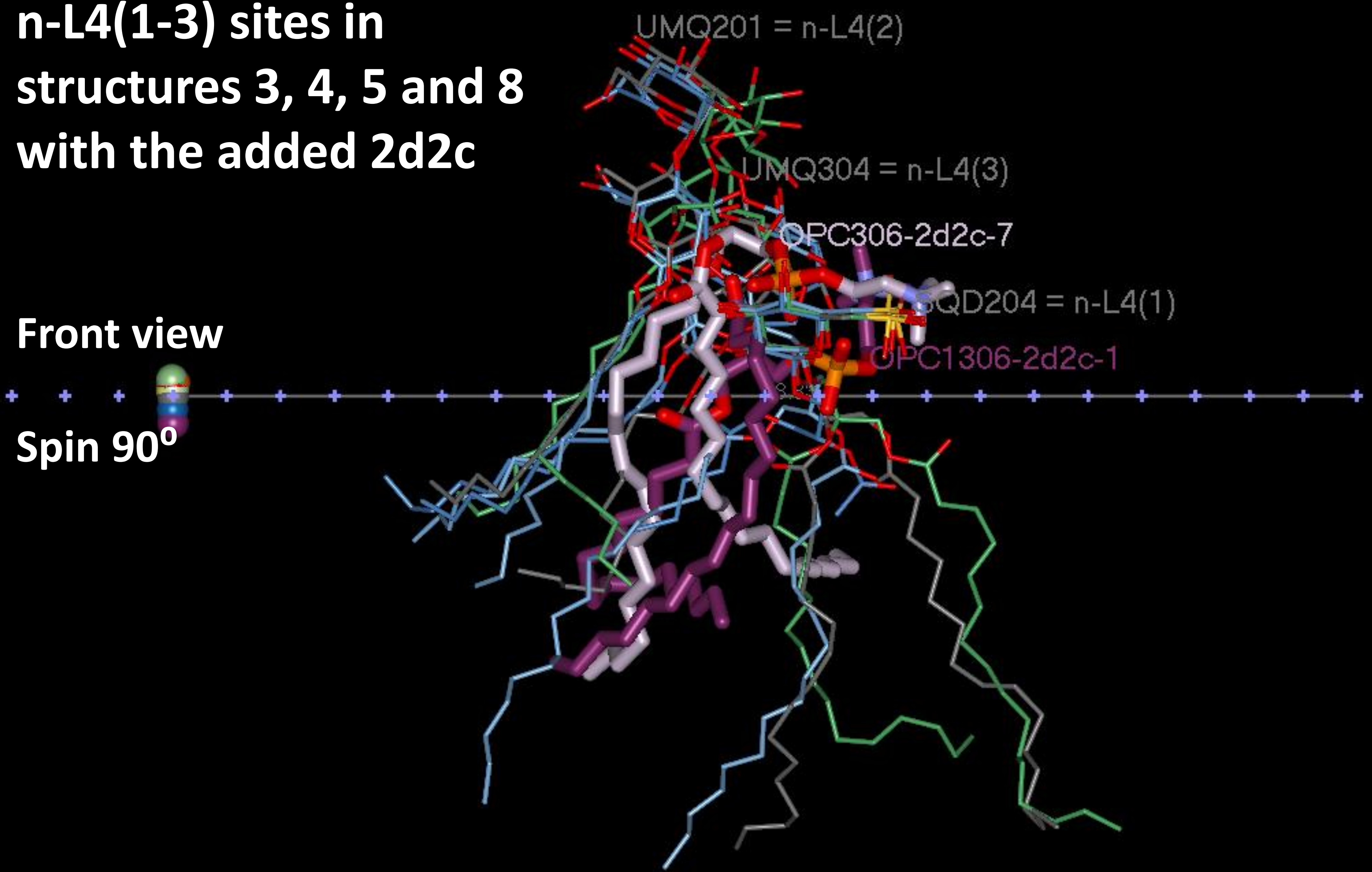

1-2d2c-s
2-1vf5-s
3-4ogq
4-4h44
5-2zt9
6-1vf5-b
7-2d2c-b
8-4pv1

n-L4(1-3) sites in structures 3, 4, 5 and 8 with the added 1vf5 and 2d2c

Front view

Spin 90º

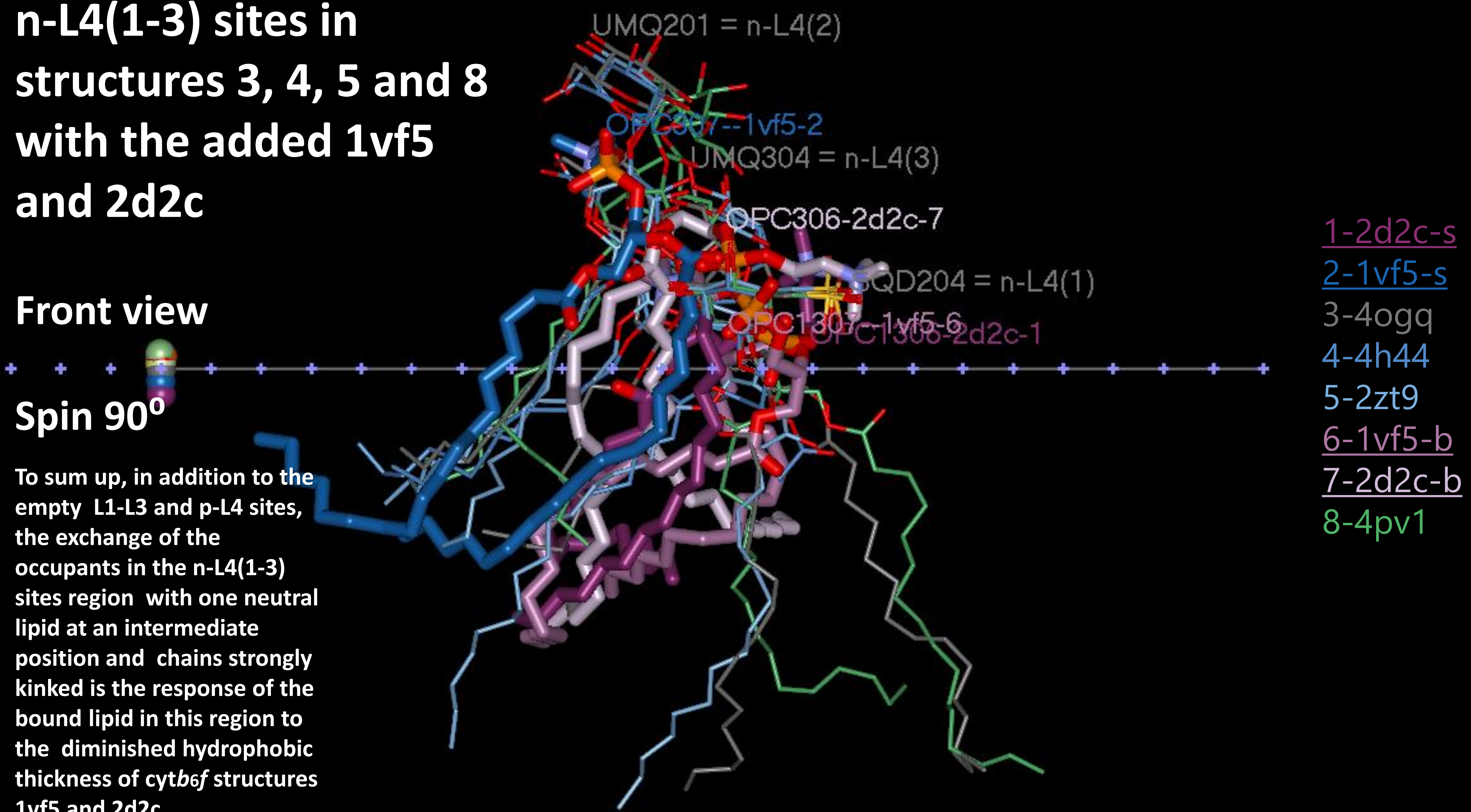

To sum up, in addition to the empty L1-L3 and p-L4 sites, the exchange of the occupants in the n-L4(1-3) sites region with one neutral lipid at an intermediate position and chains strongly kinked is the response of the bound lipid in this region to the diminished hydrophobic thickness of cyt*b*6*f* structures 1vf5 and 2d2c.

n/p-L5(1-2) sites in structures 3, 4 and 8

p-side view

These sites are empty in 5-2zt9. In the other three structures:
The n-L5(1) site is occupied by an 18-C long chain (3-4ogq), a detergent (4-4h44) and SMA inhibitor (8-4pv1). In the rest of the 11 structures, n-L5(1) is occupied by Qn-site inhibitors as indicated in Table 2.
The core lipid site n/p-L5(2) is occupied by 2WM309 (DAG) in 4ogq, by OPC208 in 4pv1 and by 8K6 (18-C long hydrocarbon chain) in three other structures (4-4h44, 12-4i7z and 13-4h13). Notably, the *sn*-2 chain of the DAG and OPC coincide with the 18-C long chain. This site is empty in the rest of 11 structures.

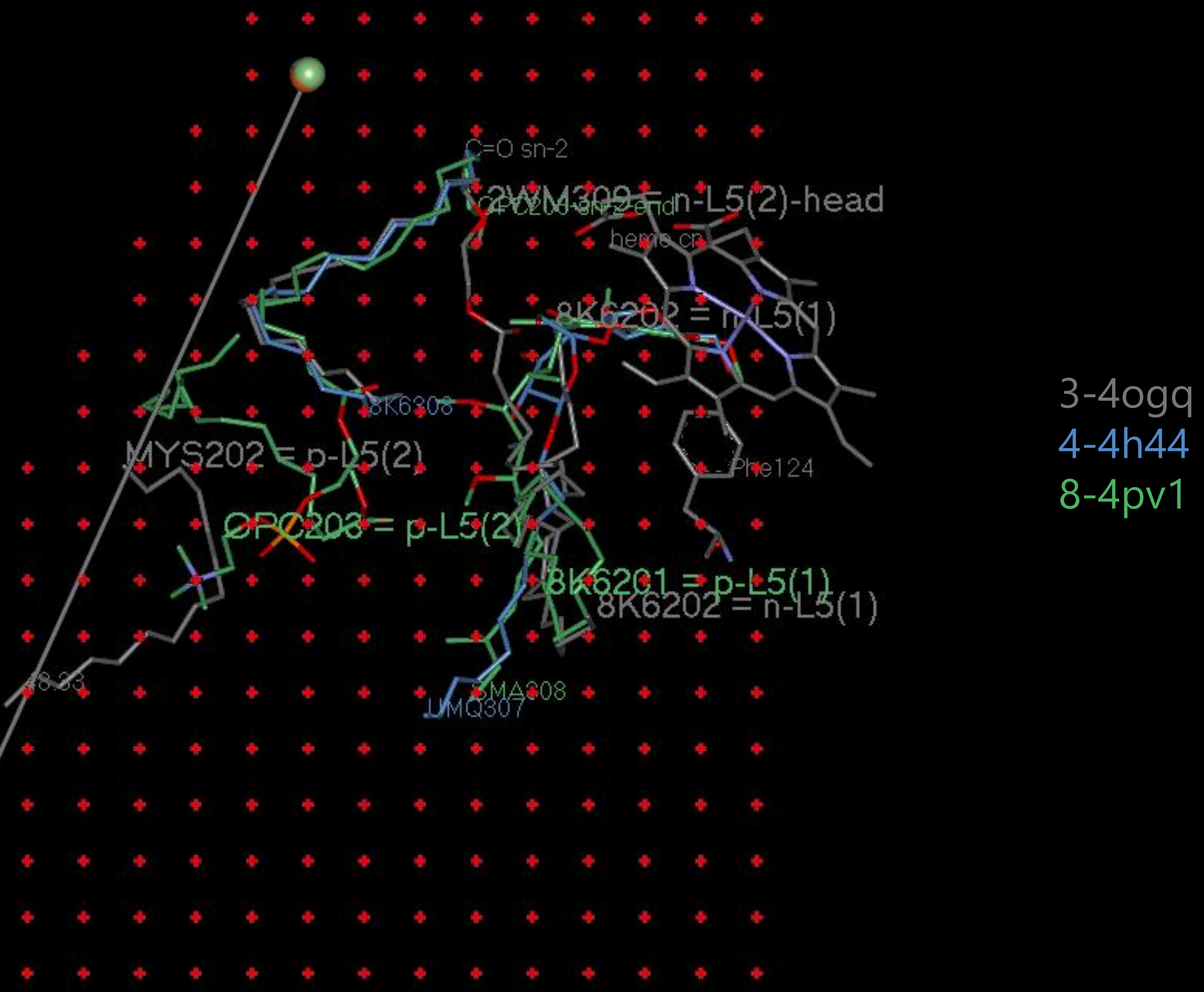

n/p-L5(1-2) sites in structures 3, 4 and 8 with the added 1vf5

p-side view

- n/p-L5(1-2) of structures 3, 4 and 8 are shown in the thinnest stick style
- The plastoquinone (PQ) molecules (n-L5(1)) of 1vf5 are shown in a thicker stick style;
- The DOPC lipids of 1vf5 are shown in the thickest stick style. The same style is applied for the DOPC lipids of 2d2c (next slides).
- As above, only the heme c_n and Phe40 of 4ogq are shown.
- Note that PQ occupies the n-L5(1) site, while DOPC is closer to n/p-L5(2) site

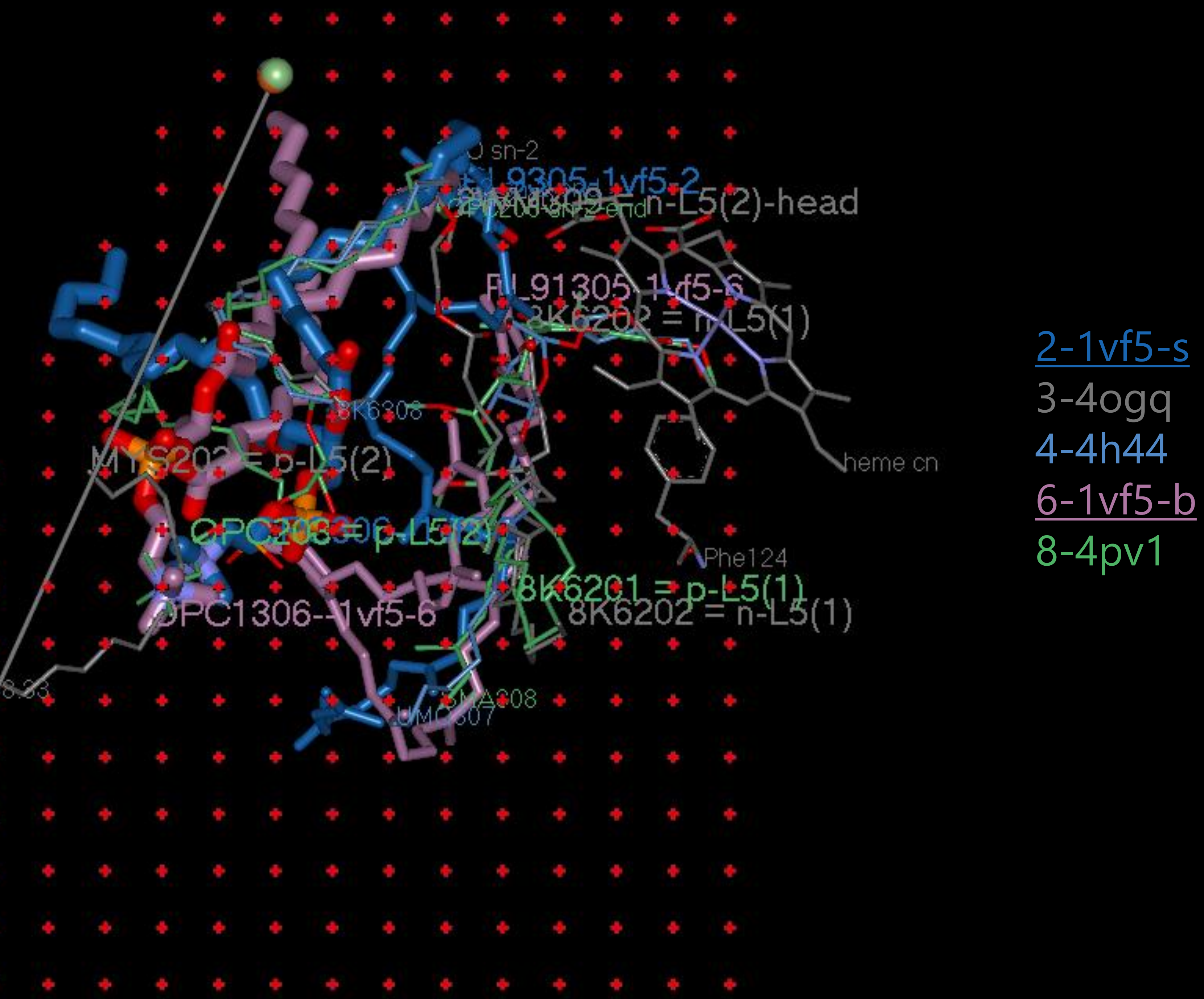

n/p-L5(1-2) sites in structures 3, 4 and 8 with the added 2d2c

p-side view

- **2d2c does not have bound PQ at the n-L5(1) site.**
- **Note that DOPC are much closer to the n-L5(1) site than to the n/p-L5(2) sites (also seen in the Front view slides below)**

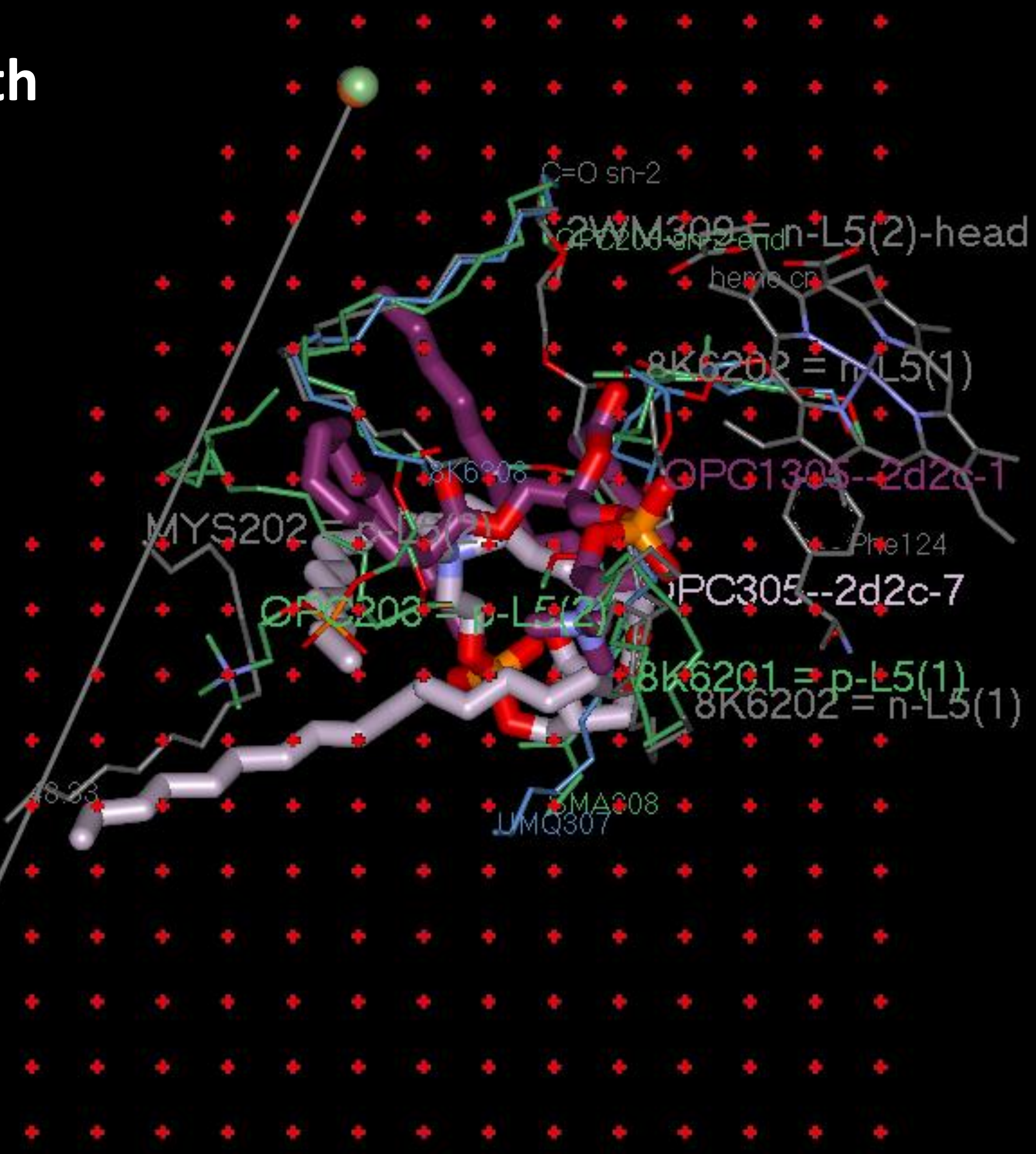

1-2d2c-s
3-4ogq
4-4h44
7-2d2c-b
8-4pv1

n/p-L5(1-2) sites in structures 3, 4 and 8 with the added 1vf5 and 2d2c

p-side view

- **Clearly, DOPC in 1vf5 and 2d2c have different positions**

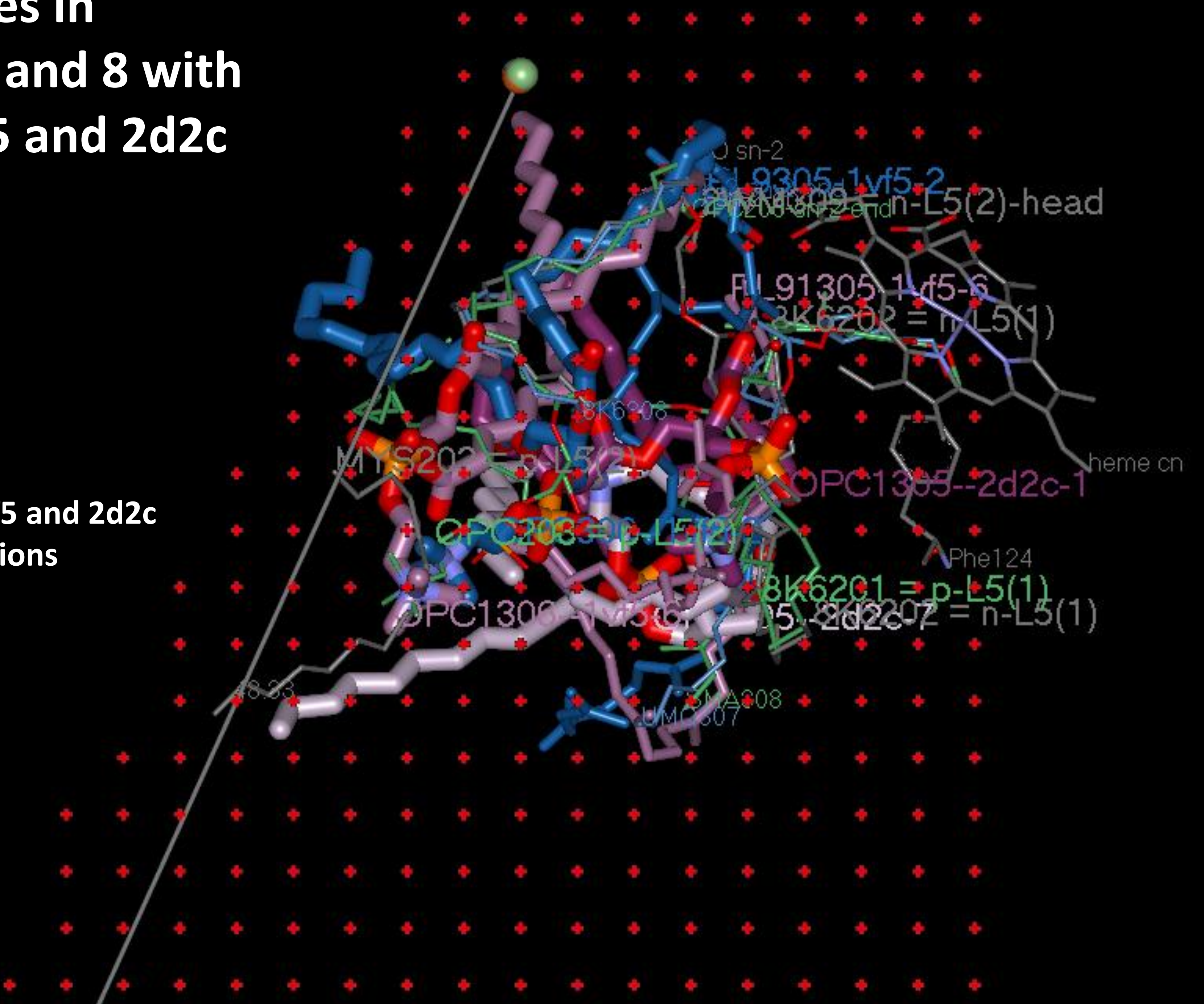

1-2d2c-s
2-1vf5-s
3-4ogq
4-4h44
6-1vf5-b
7-2d2c-b
8-4pv1

n/p-L5(1-2) sites in structures 3, 4 and 8

n-side view

- The same is seen in this n-side view series

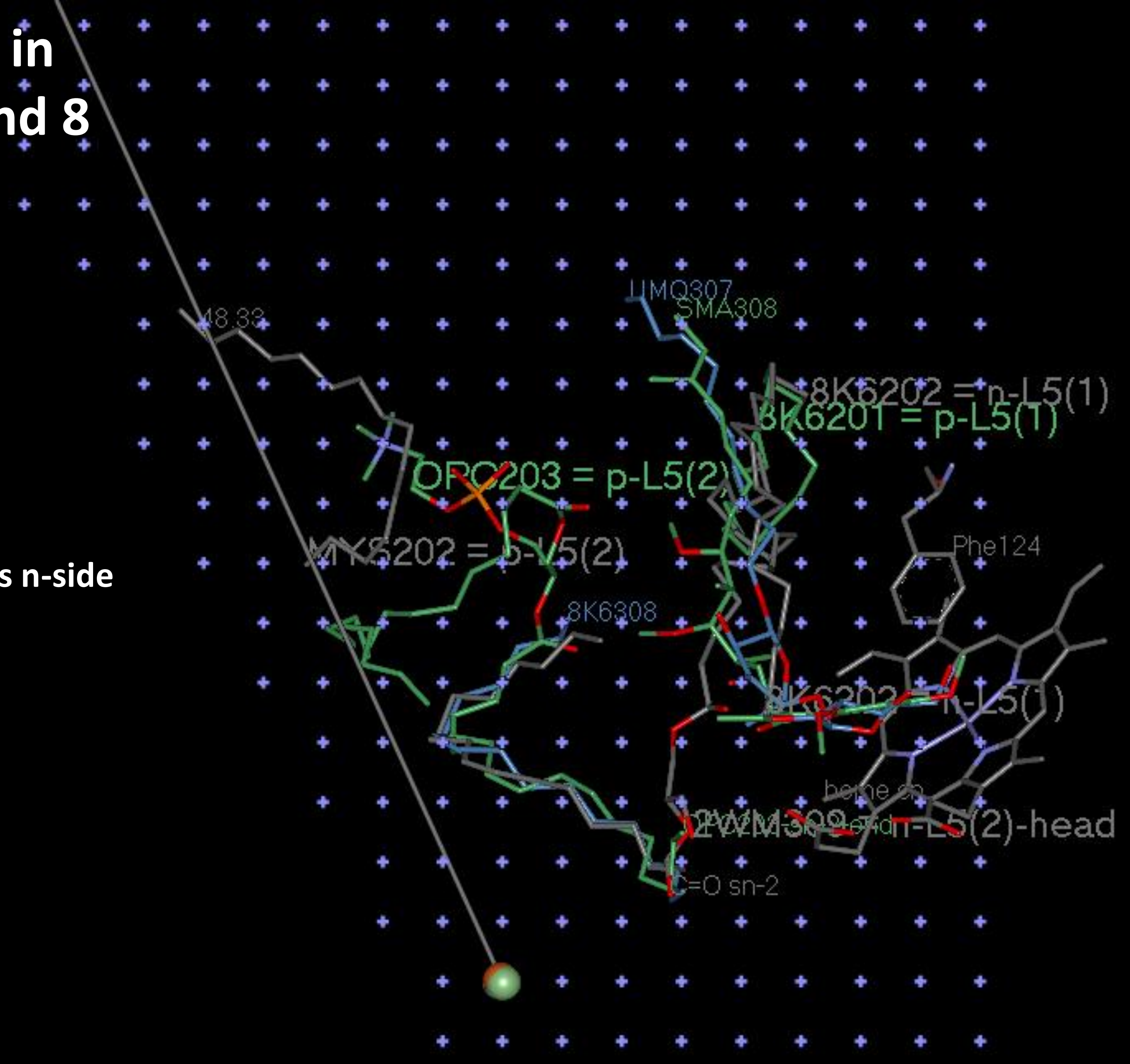

3-4ogq
4-4h44
8-4pv1

n/p-L5(1-2) sites in structures 3, 4 and 8 with the added 1vf5

n-side view

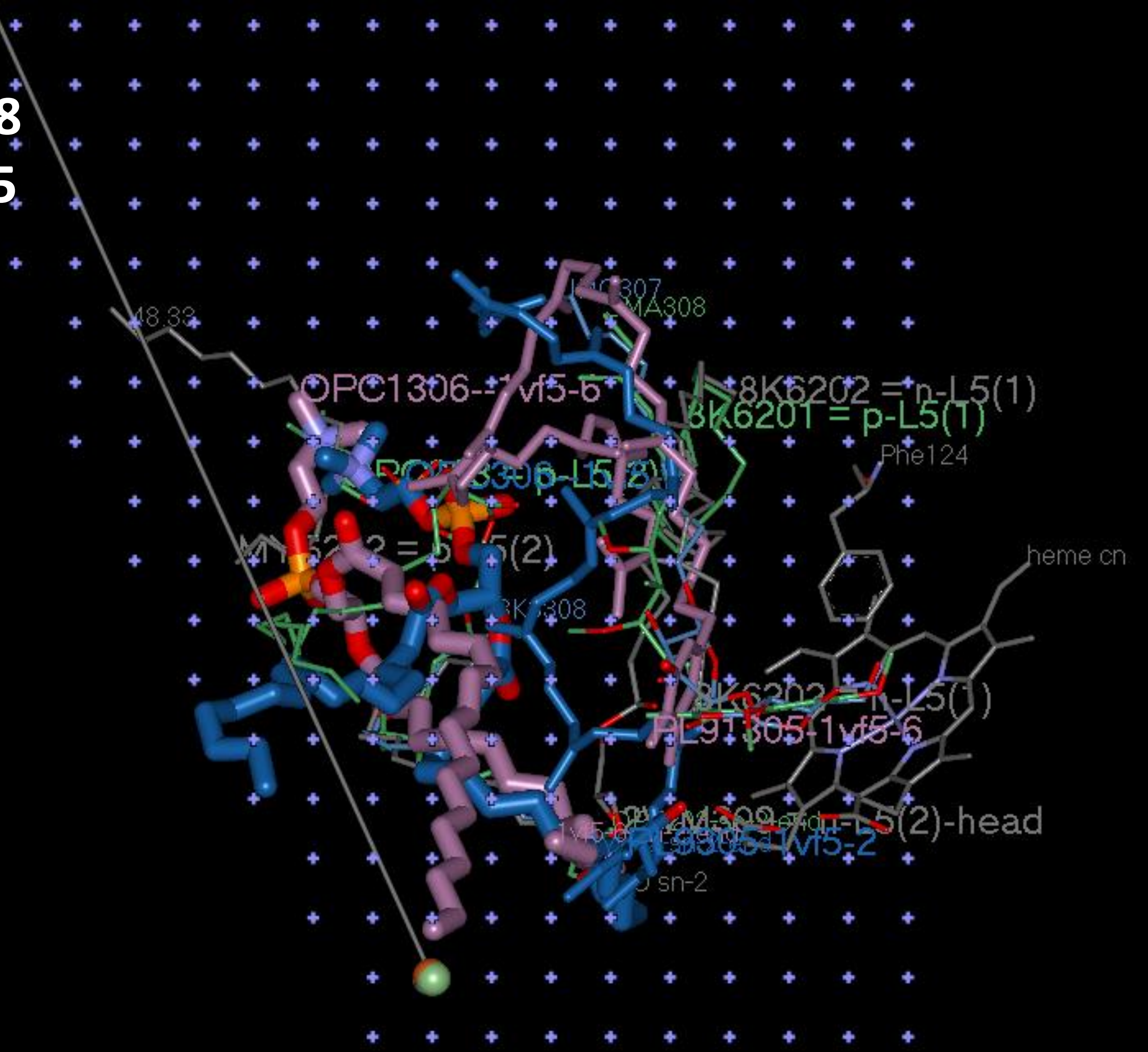

2-1vf5-s
3-4ogq
4-4h44
6-1vf5-b
8-4pv1

n/p-L5(1-2) sites in structures 3, 4 and 8 with the added 2d2c

n-side view

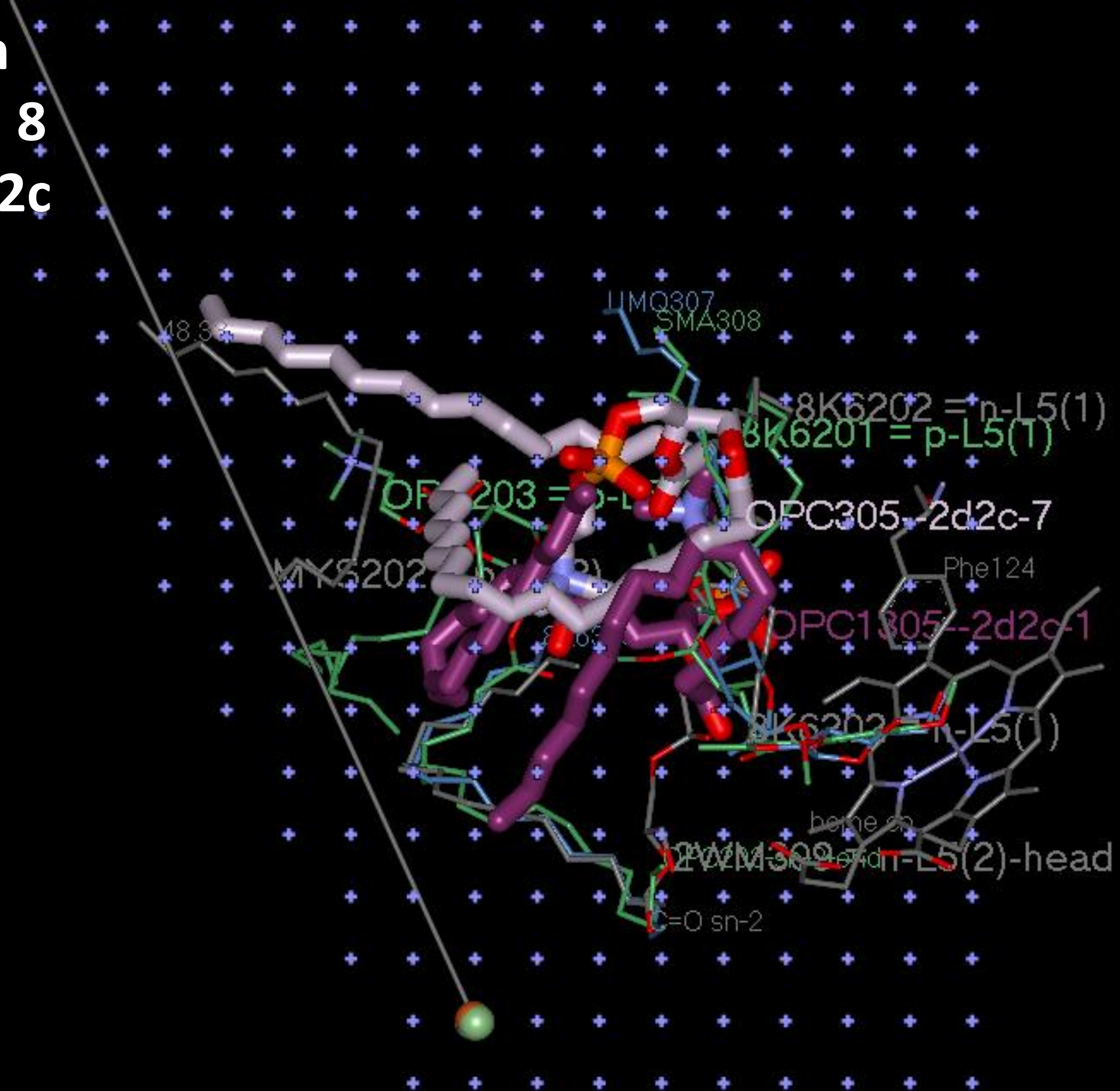

1-2d2c-s
3-4ogq
4-4h44
7-2d2c-b
8-4pv1

n/p-L5(1-2) sites in structures 3, 4 and 8 with the added 1vf5 and 2d2c

n-side view

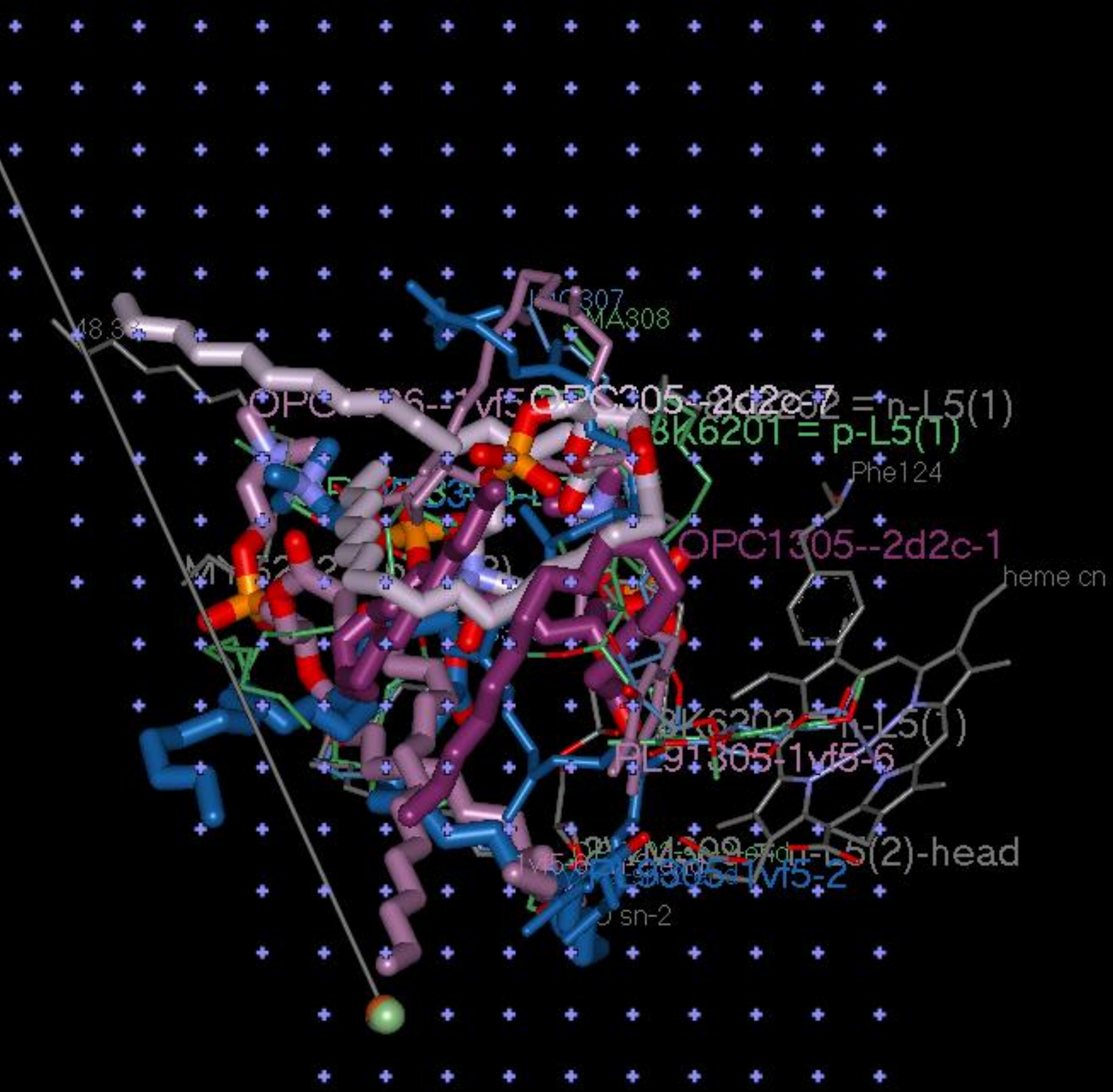

1-2d2c-s
2-1vf5-s
3-4ogq
4-4h44
6-1vf5-b
7-2d2c-b
8-4pv1

n/p-L5(1-2) sites in structures 3, 4 and 8

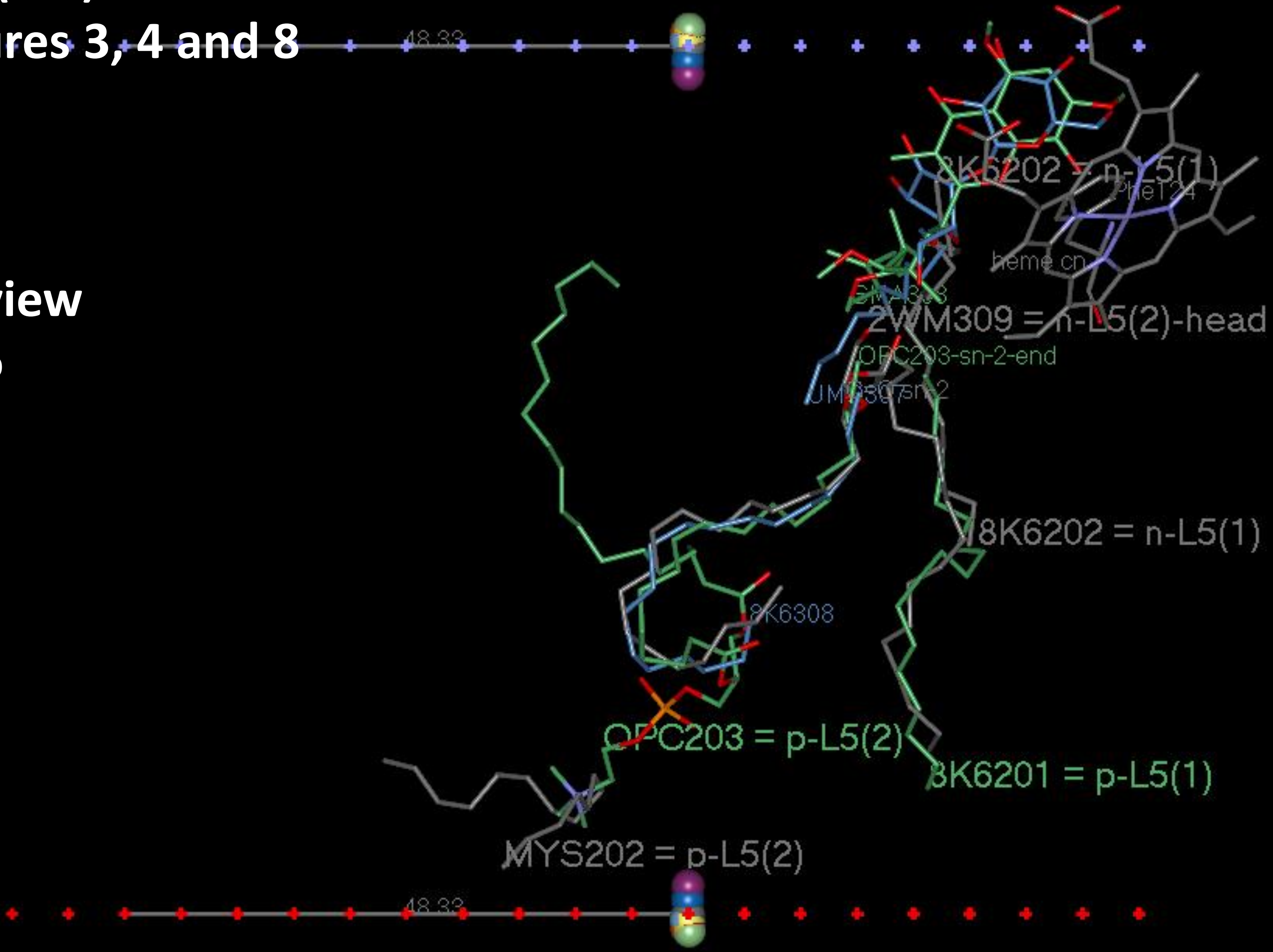

Front view

Spin 0º

3-4ogq
4-4h44
5-2zt9
8-4pv1

n/p-L5(1-2) sites in structures 3, 4 and 8 with the added 1vf5

Front view

Spin 0°

- **Note the extended transmembrane position of the two DOPC lipids with head groups and sn-2 chains coinciding with those of DOPC in 4pv1 structure – the p-L5(2) site**

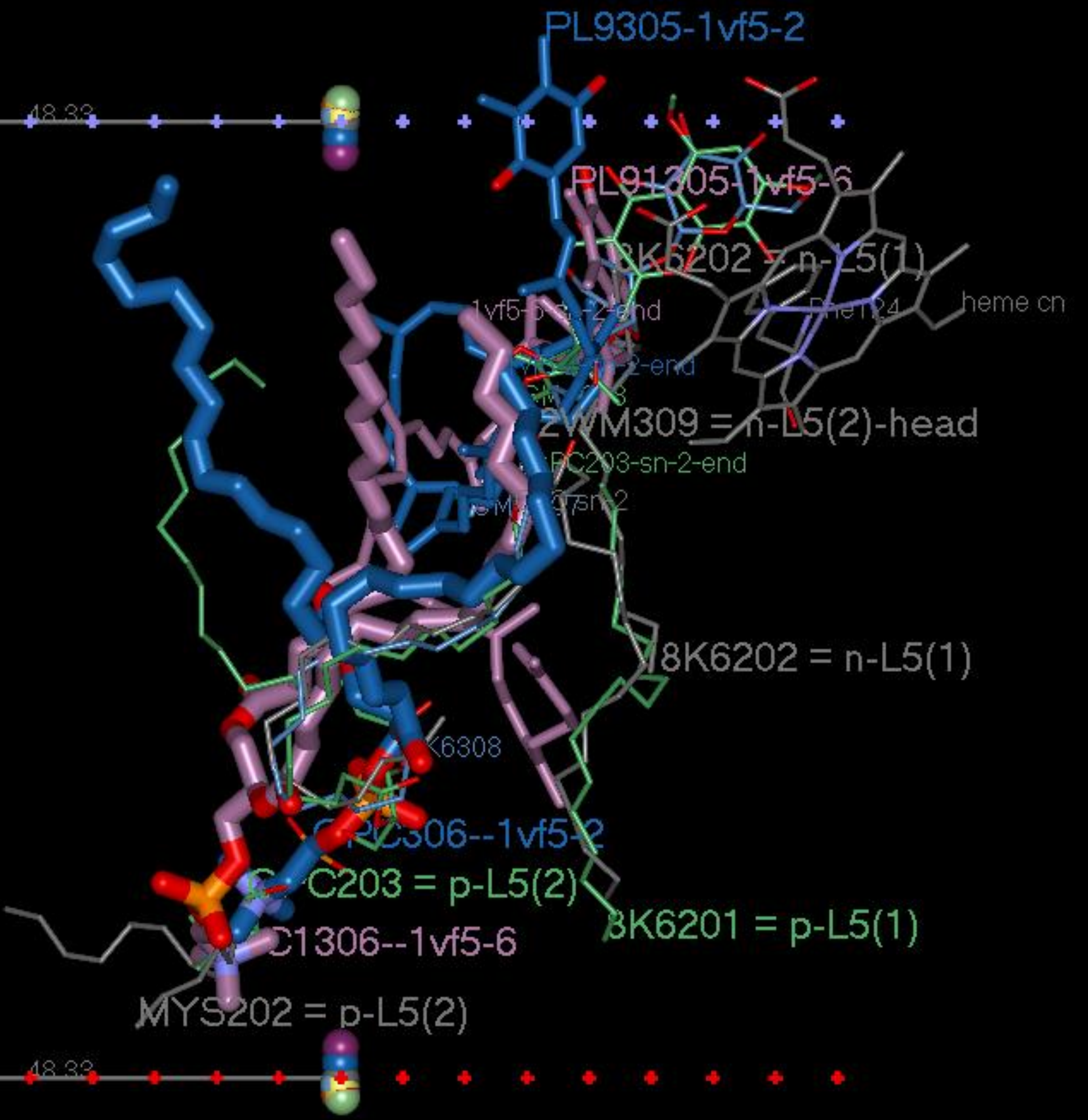

2-1vf5-s
3-4ogq
4-4h44
6-1vf5-b
8-4pv1

n/p-L5(1-2) sites in structures 3, 4 and 8 with the added 2d2c

Front view

Spin 0°

- In contrast, the two DOPC lipids in 2d2c are collected in the n-side hydrophobic half of the lipid bilayer. They are in an intermediate position between the n-L5(1) and n-L5(2).

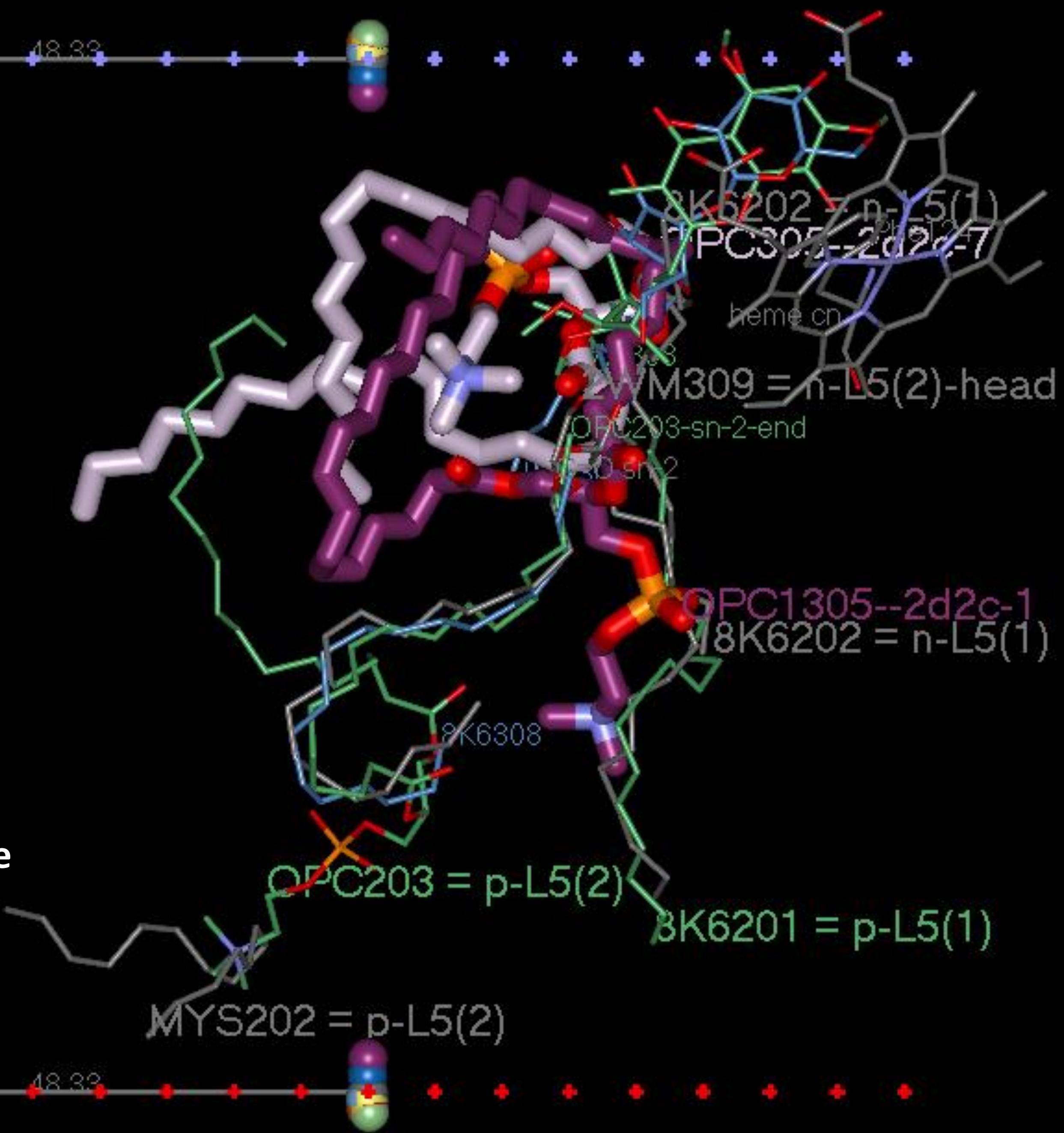

1-2d2c-s
3-4ogq
4-4h44
7-2d2c-b
8-4pv1

n/p-L5(1-2) sites in structures 3, 4 and 8 with the added 1vf5 and 2d2c

Front view

Spin 0º

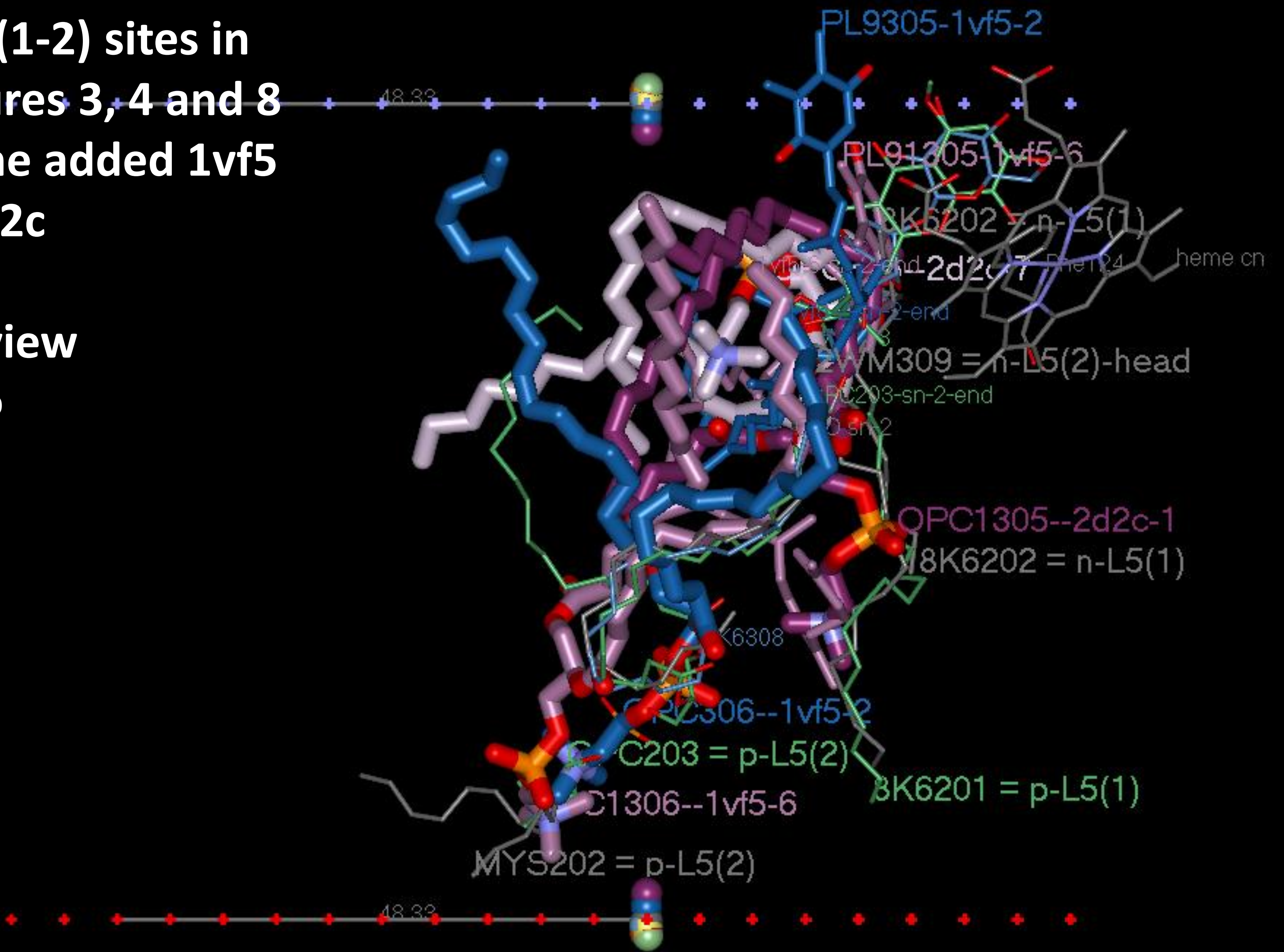

1-2d2c-s
2-1vf5-s
3-4ogq
4-4h44
6-1vf5-b
7-2d2c-b
8-4pv1

n/p-L5(1-2) sites in structures 3, 4 and 8

Front view

Spin 90º

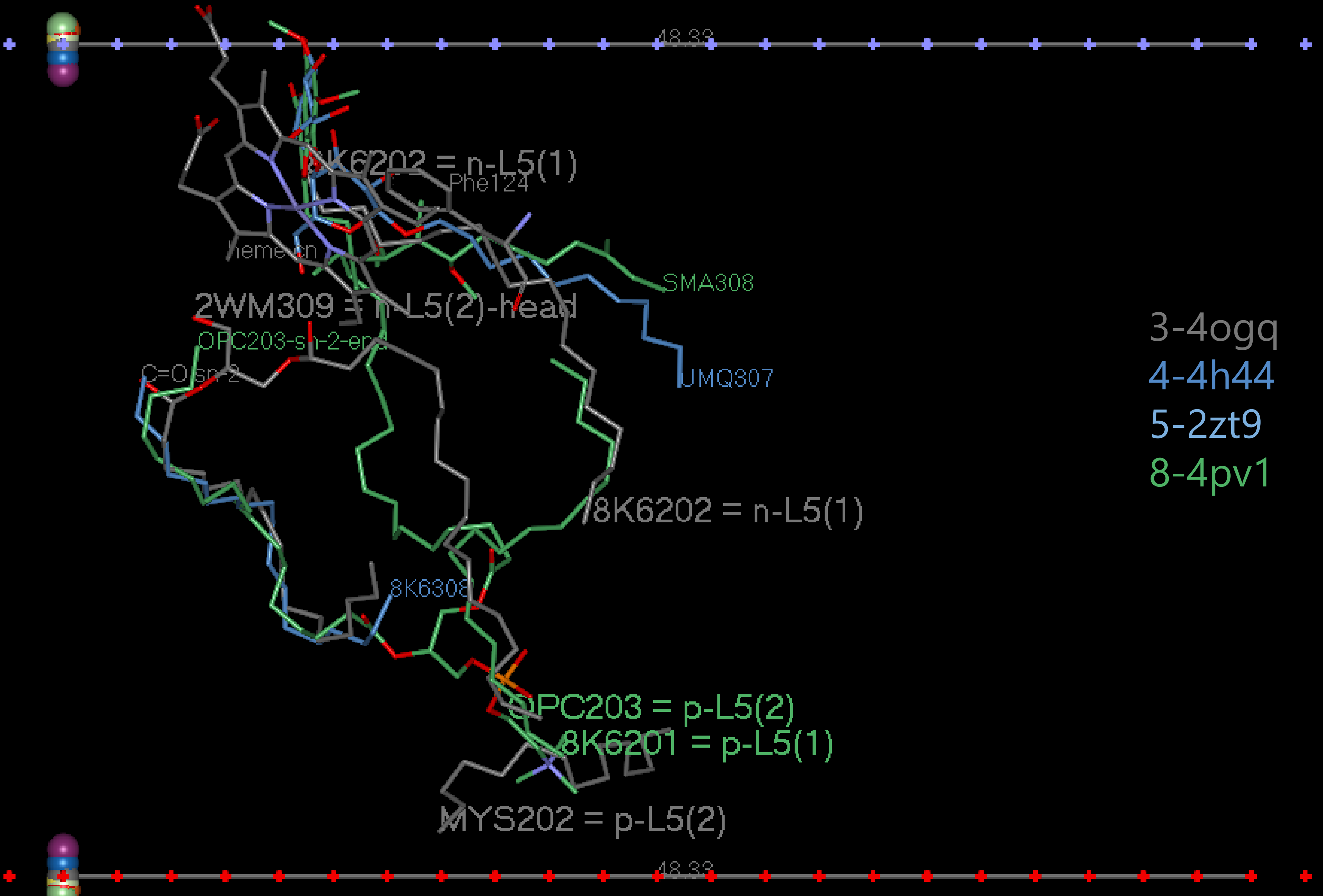

n/p-L5(1-2) sites in structures 3, 4 and 8 with the added 1vf5

Front view

Spin 90º

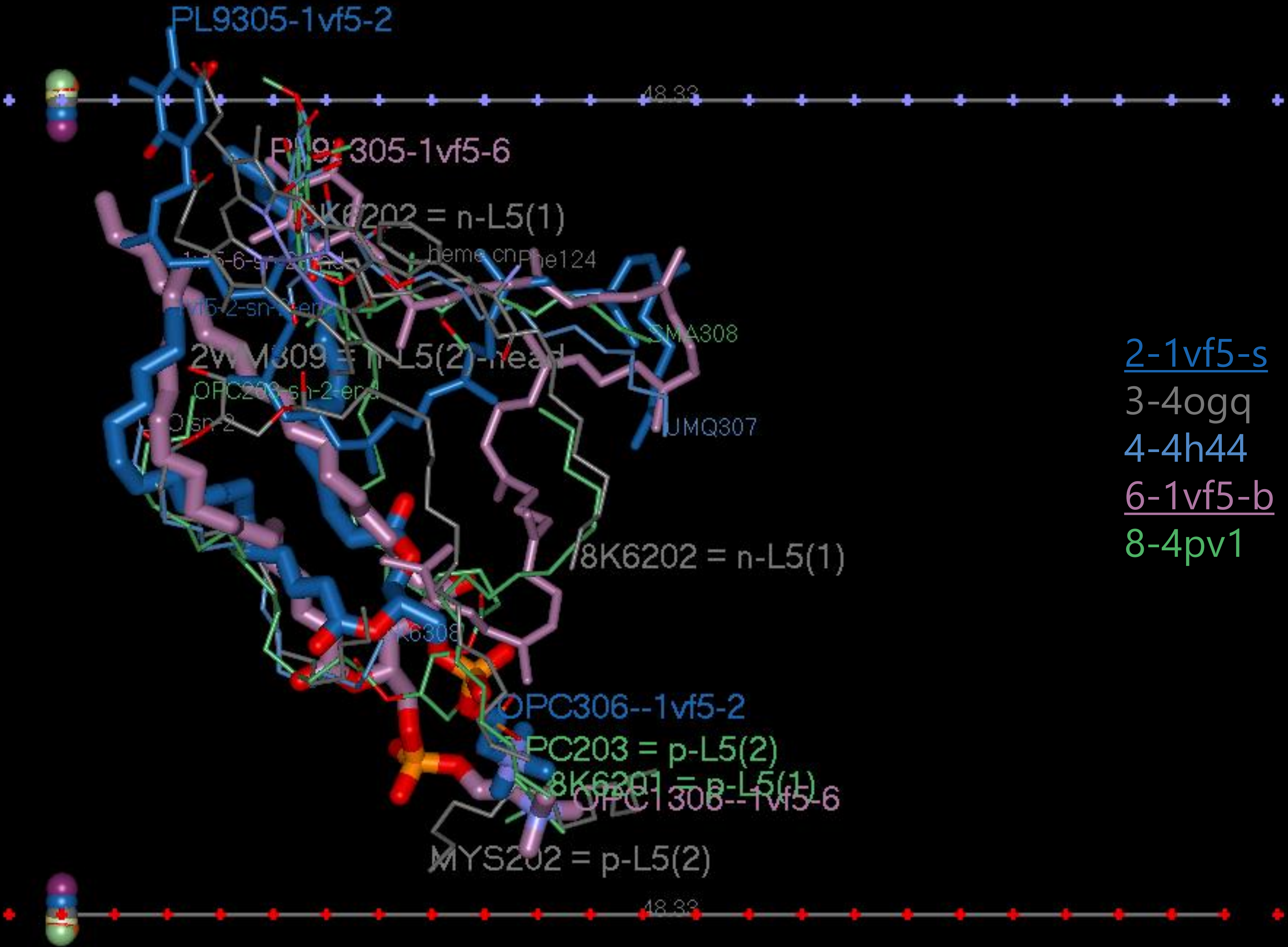

2-1vf5-s
3-4ogq
4-4h44
6-1vf5-b
8-4pv1

n/p-L5(1-2) sites in structures 3, 4 and 8 with the added 2d2c

Front view

Spin 90º

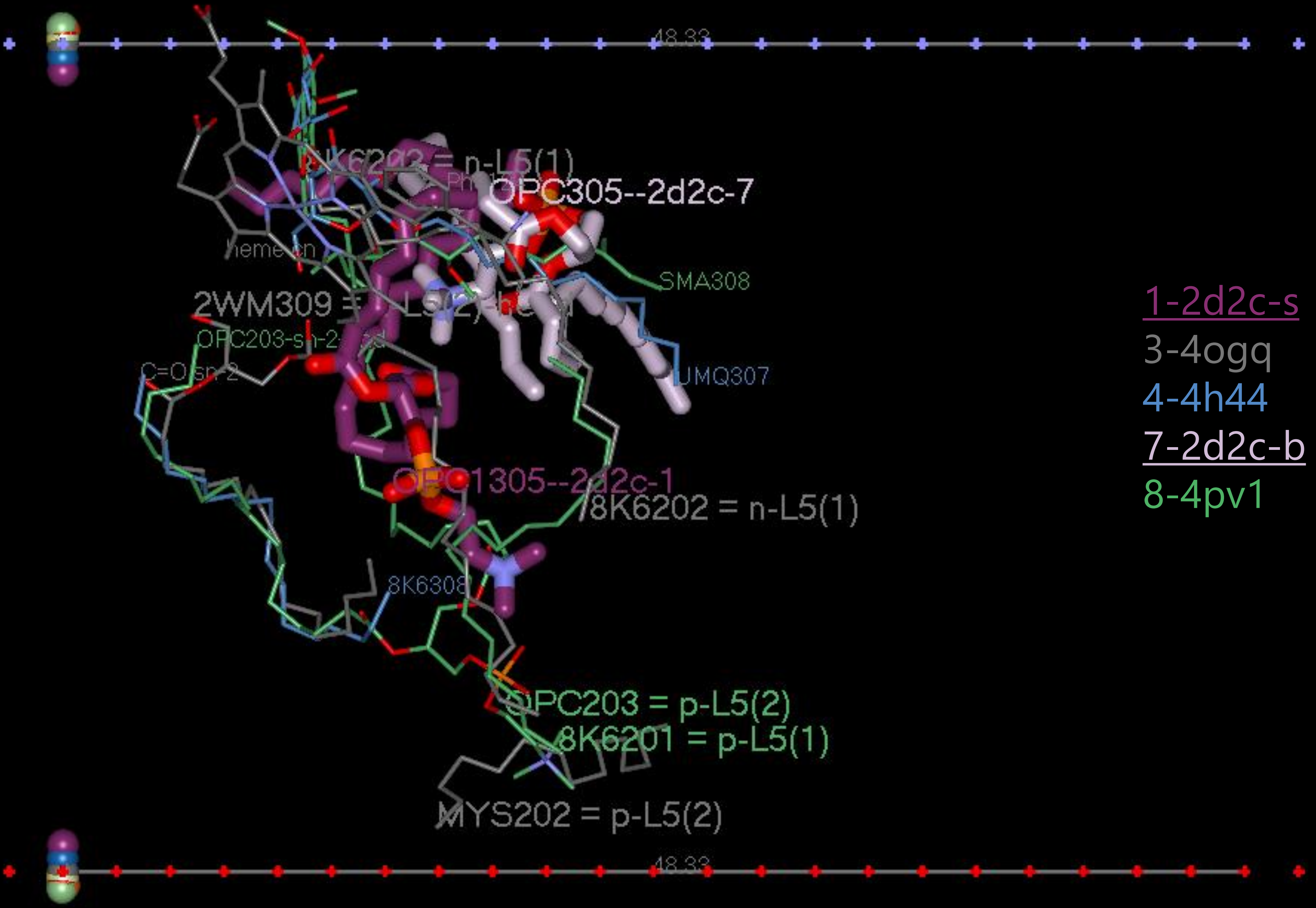

n/p-L5(1-2) sites in structures 3, 4 and 8 with the added 1vf5 and 2d2c

Front view

Spin 90º

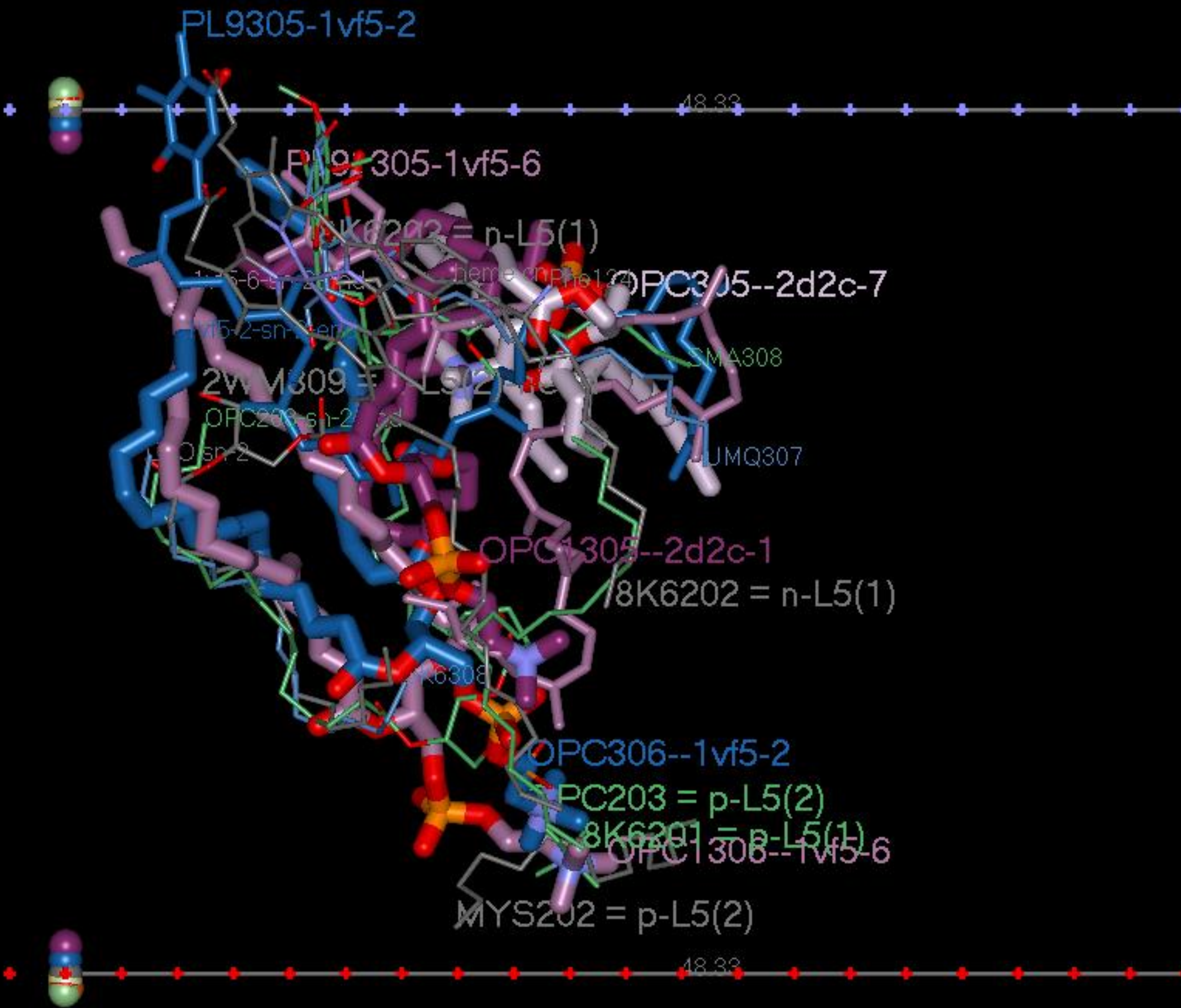

1-2d2c-s
2-1vf5-s
3-4ogq
4-4h44
6-1vf5-b
7-2d2c-b
8-4pv1
